# Gene expression profile analysis of Manila clam (*Ruditapes philippinarum*) hemocytes after a *Vibrio alginolyticus* challenge using an immune-enriched oligo-microarray

**DOI:** 10.1186/1471-2164-15-267

**Published:** 2014-04-07

**Authors:** Rebeca Moreira, Massimo Milan, Pablo Balseiro, Alejandro Romero, Massimiliano Babbucci, Antonio Figueras, Luca Bargelloni, Beatriz Novoa

**Affiliations:** 1 Instituto de Investigaciones Marinas (IIM), Consejo Superior de Investigaciones Científicas (CSIC), Eduardo Cabello 6, 36208 Vigo, Spain; 2 Department of Comparative Biomedicine and Food Science (BCA), University of Padova, Viale dell’Università 16, 35020 Legnaro, Italy

**Keywords:** *Ruditapes philippinarum*, *Vibrio alginolyticus*, Hemocytes, Oligo-microarray, Gene ontology, Blast2GO, Immune response

## Abstract

**Background:**

The Manila clam (*Ruditapes philippinarum*) is a cultured bivalve with worldwide commercial importance, and diseases cause high economic losses. For this reason, interest in the immune genes in this species has recently increased. The present work describes the construction of the first *R. philippinarum* microarray containing immune-related hemocyte sequences and its application to study the gene transcription profiles of hemocytes from clams infected with *V. alginolyticus* through a time course.

**Results:**

The complete set of sequences from *R. philippinarum* available in the public databases and the hemocyte sequences enriched in immune transcripts were assembled successfully. A total of 12,156 annotated sequences were used to construct the 8 ×15 k oligo-microarray. The microarray experiments yielded a total of 579 differentially expressed transcripts. Using the gene expression results, the associated Gene Ontology terms and the enrichment analysis, we found different response mechanisms throughout the experiment. Genes related to signaling, transcription and apoptosis, such as IL-17D, NF-κB or calmodulin, were typically expressed as early as 3 hours post-challenge (hpc), while characteristic immune genes, such as PGRPs, FREPs and defense proteins appeared later at 8 hpc. This immune-triggering response could have affected a high number of processes that seemed to be activated 24 hpc to overcome the *Vibrio* challenge, including the expression of many cytoskeleton molecules, which is indicative of the active movement of hemocytes. In fact functional studies showed an increment in apoptosis, necrosis or cell migration after the infection. Finally, 72 hpc, activity returned to normal levels, and more than 50% of the genes were downregulated in a negative feedback of all of the previously active processes.

**Conclusions:**

Using a new version of the *R. philippinarum* oligo-microarray, a putative timing for the response against a *Vibrio* infection was established. The key point to overcome the challenge seemed to be 8 hours after the challenge, when we detected immune functions that could lead to the destruction of the pathogen and the activation of a wide variety of processes related to homeostasis and defense. These results highlight the importance of a fast response in bivalves and the effectiveness of their innate immune system.

## Background

The Manila clam (*Ruditapes philippinarum*) is a cultured bivalve species with high worldwide commercial importance and value. The culture of these clams has been increasing in recent years, especially in Europe and Asia. Therefore, diseases can result in significant economic losses. The majority of diseases in clams are associated with *Vibrio*[1,2] and *Perkinsus*[3,4] species. Diseases can affect not only the development and survivorship of these organisms [5,6] but also the quality and price of the product. Therefore, there has been a growing interest in the study of the molecular biology of the defense mechanisms in bivalves in the last decade. Although molluscs lack a specific immune system, the innate response involving circulating hemocytes and a large variety of molecular effectors seems to be an efficient defense method for responding to external aggressions by detecting the molecular signatures of an infection [7-11]. Hemocytes are the main line of cellular defense against invading pathogens in molluscs [12], and the immune genes expressed in bivalve hemocytes are of great interest to researchers [13-19]. In recent years, a serious effort has also been made to increase the number of *R. philippinarum* sequences in public databases. Before 2011, less than 6,000 nucleotide sequences were available for this species. Our two groups released 457,717 and 975,190 raw reads from adult/larval tissues and hemocytes, respectively [20,21]. Concurrently, studies by Ghiselli *et al*. on gonad tissue yielded approximately 90 million raw sequences [22].

Oligo-microarrays are a sensitive and reproducible high-throughput technology for analyzing the gene expression of thousands of genes simultaneously [23]. This platform has been used by scientists to study the expression profile in many species from yeast to human [24,25]. Microarrays have also been applied to bivalves for different purposes [26-28]. To our knowledge, a toxicogenomics study in the gill and the digestive gland [20] and a recent study of the response to brown ring disease, [29] are the only works to date to use an oligo-microarray in *R. philippinarum*.

The main objective of this work was to analyze the response of the Manila clam against strain TA15 of *V. alginolyticus* through a time course. *V. alginolyticus* was previously reported to produce important mortality in clam larvae [6]. For this study, a new version of Manila clam DNA microarray including the sequences of thousands of hemocyte-exclusive genes [21] was designed and developed.

## Results and discussion

### Assembly and annotation

A summary of the sequence origin, assembly and annotation results is shown in Table 1. From the total 1,438,665 sequences from *R. philippinarum,* the Newbler software package (GS *De Novo* Assembler v2.6, Roche) was able to assemble 88.24% of the raw sequences, and 11.76% of the sequences (169,223) remained as singletons. The assembly resulted in 26,708 isotigs grouped into 15,175 isogroups and 156 contigs.

**Table 1:** Description of the microarray design process

	**n° sequences**
**Sequence origin**	
Sanger (Milan *et al*. [20])	5,758
454 tissues (Milan *et al*. [20])	457,717
454 hemocytes (Moreira *et al*. [21])	975,190
subTOTAL	1,438,665
NCBI	2,050
TOTAL	1,440,715
**Assembly**	
Singletons	169,223
Singletons phred Q > 20	16,495
Isotigs	26,708
Isogroups	15,175
Contigs	156
**Annotation**	
Singletons phred Q > 20 and NCBI	5,914
Contigs and longest isotig of each isogroup	6,242
Frame +	7,827
Frame -	2,761
Ambiguous frame	1,568
TOTAL *R. philippinarum* successfully designed probes	13,671

The longest isotig of each isogroup, the contigs, the singletons with more than 200 bp of continuous sequence with a Phred Q > 20 (16,495) and the *R. philippinarum* ESTs in the NCBI database (2,050) were then considered for the annotation. The putative identities of these sequences were obtained by running BlastX and BlastN similarity searches in 48 different protein and nucleotide databases. Additional file 1: Table S1 shows the percentage value of annotation success of each database. The protein databases showed a higher average percentage of matches (18.84%) than the nucleotide ones (5.3%), presumably due to the degeneration of the genetic code. Furthermore, the species databases yielded much lesser annotation percentage than the general ones (NCBI) with the exception of certain databases, probably because of the huge amount of sequences available, in the case of *H. sapiens,* or the higher phylogenetic similarity, with *S. purpuratus* and *L. gigantea*. Thus, we found it is worth the use of general protein databases and the specific ones previously named since only 1.5% of the contigs/isotigs and the 4.1% of the singletons were annotated with the remaining specific databases. A total of 12,156 successfully annotated transcripts were considered for the *R. philippinarum* DNA microarray design (see Methods). As most sequence reads were obtained from a non-directional cDNA library, sense strand orientation was inferred putatively from the homologous protein sequences of other species. One probe for annotated transcripts with known orientation was designed to construct a high-density oligo-DNA microarray, while two probes with both orientations were designed for contigs with ambiguous orientation (see Table 1). A total of 13,671 probes representing 12,108 unique transcripts were created using the Agilent eArray interface (https://earray.chem.agilent.com/earray/).

### Microarray hybridization, robustness and validation

A total of 36 microarray experiments were performed. The upper and lower fluorescence values were erased from the raw data (20 - 90^th^ percentile) in all of the experiments, and only robust fluorescence values were used to analyze the expression and function of the results.

**Figure 1:**
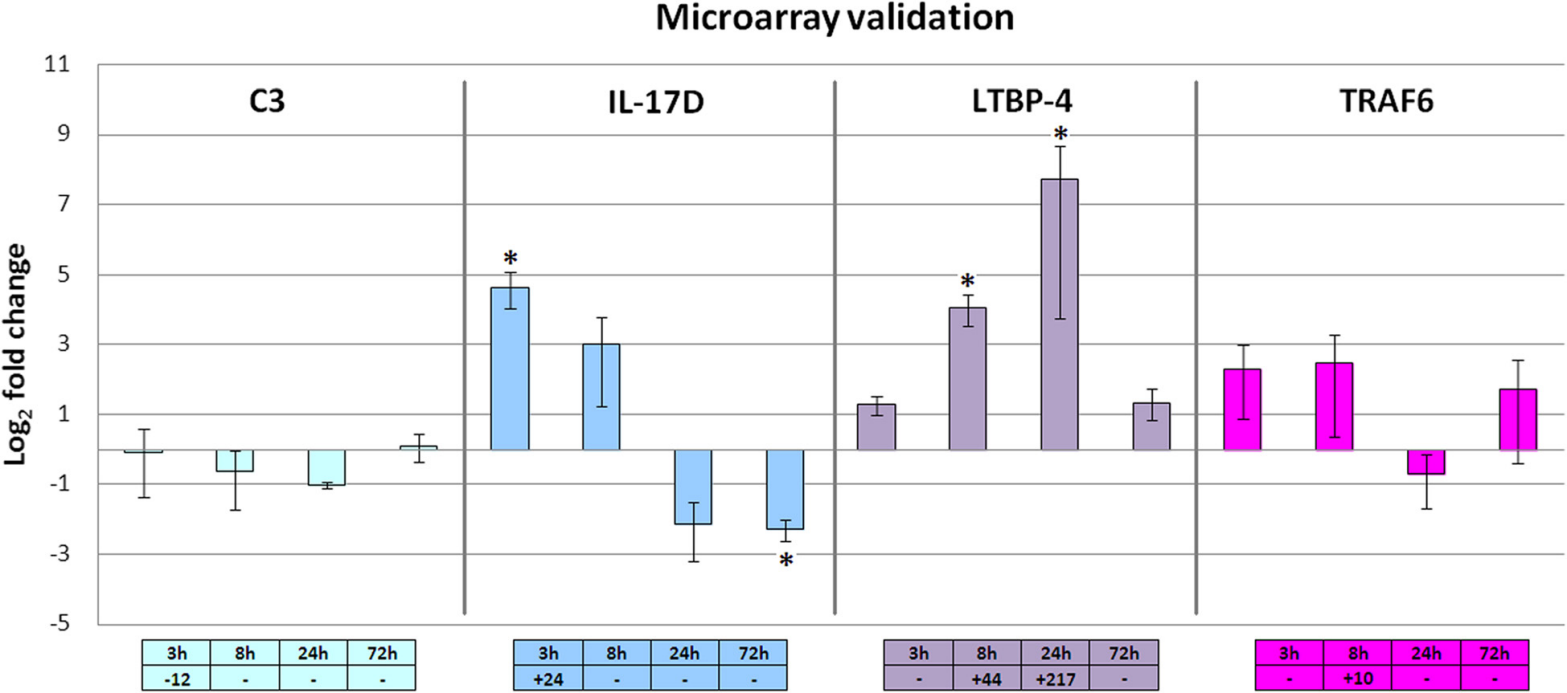
**Validation of the microarray.** qPCR expression of selected genes: C3 (Complement component C3), IL-17D (Interleukin 17 D), LTBP-4 (Latent Transforming Growth Factor-β Binding Protein 4) and TRAF6 (tumor necrosis factor receptor-associated factor 6). Data are log2 transformed to facilitate the illustration of downregulated genes. All qPCR reactions were performed as technical triplicates and the expression level of analyzed genes was normalized using the 18S rRNA. Fold change units were then calculated by dividing the normalized expression values of hemocytes from infected clams by the normalized expression values of the controls. Each bar represents the mean and standard error of the biological replicates. Asterisks highlight the significant fold changes (t-test, p < 0.05). Microarray results (t-test, p < 0.01) are indicated in the boxes below the graph.

Quantitative PCR (qPCR) is commonly used to confirm the results obtained from microarray analysis, and although microarray and qPCR data could disagree, it is known that qPCR using SYBR Green is useful to validate Agilent inkjet-printed 60-mer oligo-microarrays [30]. Specific primers were designed to perform a qPCR for four selected genes with cDNAs synthesized from the same RNAs used for the microarray hybridization. The expression patterns identified for these genes by the array and by qPCR showed similar profiles (Figure 1). Significant differences in expression compared with the controls (t-test, p < 0.05) were observed in the qPCR results and matched the microarray results in most of the cases (IL-17D 3 h, LTBP-4 8 h and 24 h) but showed some new significant results in one case (IL-17D 72 h). The direction of the regulation was always comparable in both techniques.

The four genes selected for qPCR were chosen based on their relevance in the immune system. The complement component C3 is the central component of the complement system, whose functions are to distinguish and eliminate pathogens and trigger an inflammatory response [31]. The complement system has been characterized in the carpet shell clam *R. decussatus* and was found to be inhibited early after a *Vibrio* challenge, which concurs with our results [32]. The interleukin (IL) 17D belongs to a particular family with no sequence similarity to any other known cytokines. In humans, IL-17D regulates cytokine production in endothelial cells and has an inhibitory effect on hematopoiesis [33]. Previous works in vertebrates and invertebrates have analyzed IL-17 expression after a bacterial challenge [34,35]. These studies showed a general increase in IL-17 expression until a maximum level was reached between 6 and 24 hours post-challenge in both vertebrates and invertebrates, with the exception of the turbot intestine. In this fish species, the expression profile of IL-17 is more similar to our results, with an initial increase and subsequent decrease and inhibition. The latent-transforming growth factor beta-binding protein 4 (LTBP-4) assists in TGF-β folding, secretion and activation by mediating its matrix targeting [36] and is also involved in cell adhesion and migration [37]. The TNF receptor-associated factor 6 (TRAF6) is a key signaling adaptor molecule common to the TNFR superfamily and the IL-1R/TLR family that leads to the activation of the nuclear factor kappa-B (NF-κB) and AP-1 transcription factors. TRAF6 has been identified in other bivalves, such as the Zhikong scallop [38], and the expression of TRAF6 in hemocytes after a bacterial challenge (peptidoglycan) *in vitro* indicates an initial significant inhibition of TRAF6 transcription (3 h) followed by a subsequent recovery of the basal levels (6 h). This result does not coincide with our results in either expression or in timing, and this discrepancy could be a result of the different experimental designs. Nevertheless a late inhibition (24 h) and recuperation of the basal expression (72 h) was observed in *R. philippinarum*.

### Gene expression profile after TA15 stimulation

To select genes that were significantly regulated by the *V. alginolyticus* challenge, we performed a t-test (p < 0.01) to find genes that were significantly different between the control and infected samples and an ANOVA (p < 0.05) to analyze the effect of the challenge in the whole dataset.

**Figure 2:**
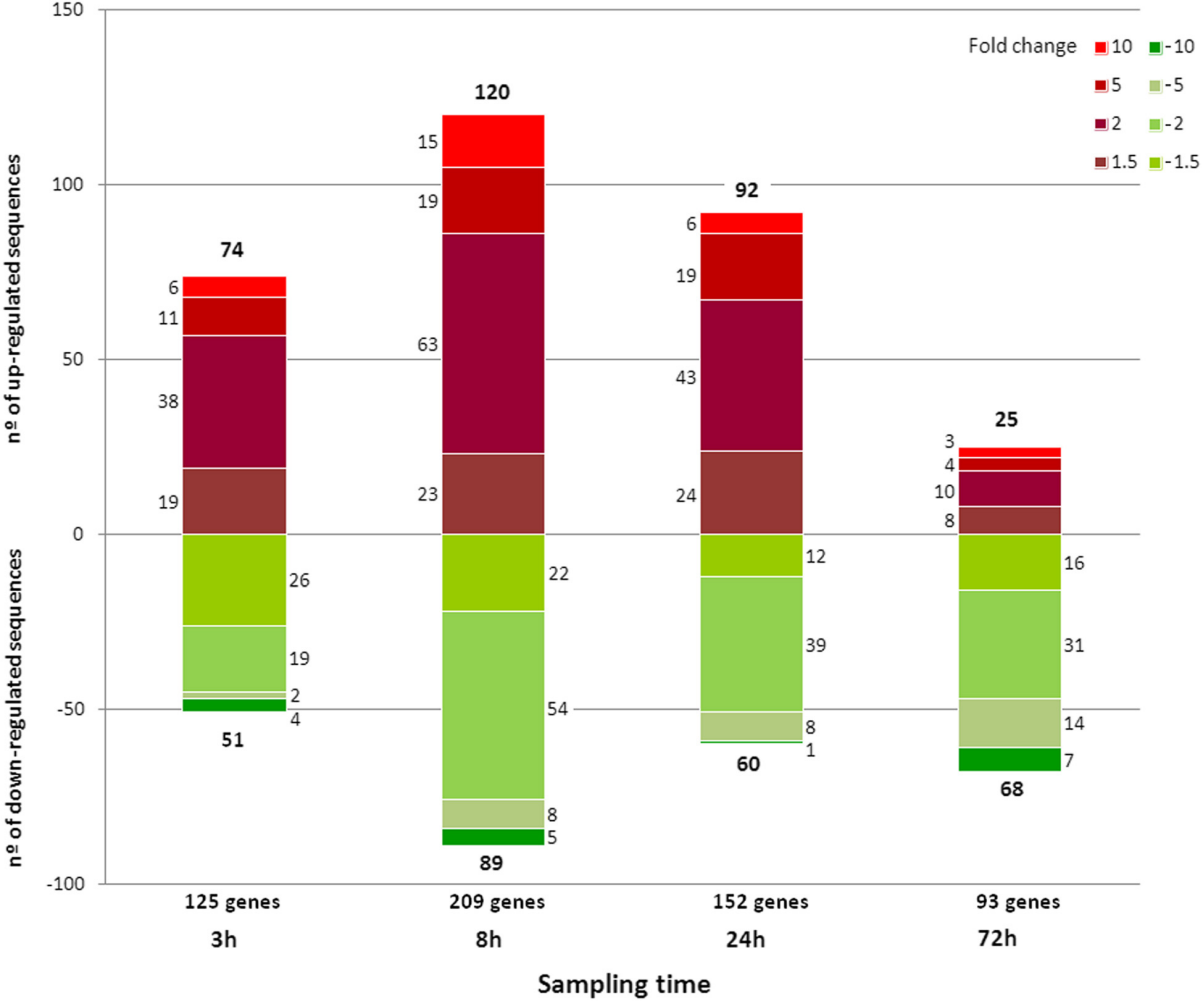
**Distribution of regulated genes through time.** Statistically significant gene modulation is subdivided according to intensity (fold change) and sense (up and down-regulation).

The t-test yielded the identification of 579 differentially expressed genes between the control groups and the *Vibrio*-infected groups. The highest number of differentially expressed genes (209) was found 8 hours after the challenge, while 125, 152 and 93 significantly differentially expressed genes were found 3, 24 and 72 hours after challenge, respectively. The expression pattern is an increase in the number of differentially expressed genes to 8 hours and a progressive decrease until reaching the minimum at 72 hours after the challenge. Figure 2 summarizes the results of the t-test and describes the number of regulated genes in fold change groups.

**Figure 3:**
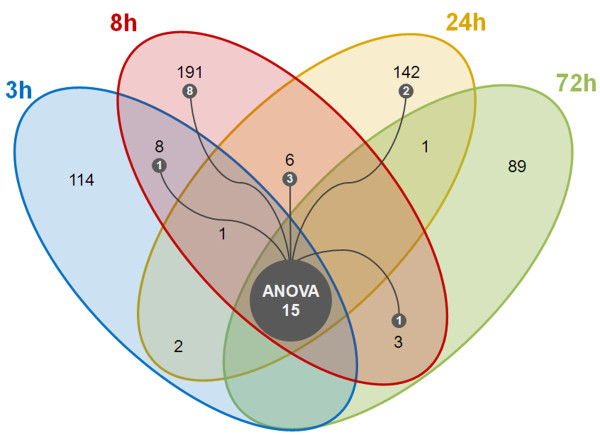
Venn diagram showing the common regulated genes among the four sampling times.

A Venn diagram illustrates the expression of the exclusive and common genes between the different sampling times in response to *V. alginolyticus* (Figure 3). This Venn diagram shows that the general transcription pattern of these genes is on/off, which implies that the majority of the genes were regulated at a single point (on) and only transcribed at basal levels the rest of the time (off). The genes expressed at several time points were scarce (21 out of 579, 3.63%), and only the ras-related c3 botulinum toxin substrate 3 (RAC3), a small GTPase of the Rho family implicated in cell differentiation, migration and apoptosis [39], was expressed in three consecutive sampling points (3, 8 and 24 hours post-challenge). In Figure 3, the coincident genes detected by the ANOVA and the t-test are also shown. The ANOVA analysis resulted in 15 regulated genes, and interestingly, 13 of these genes were in the group of 8 hours post-challenge and 8 genes were exclusive to this sampling point (for a description of these genes, see the ANOVA section).

**Figure 4:**
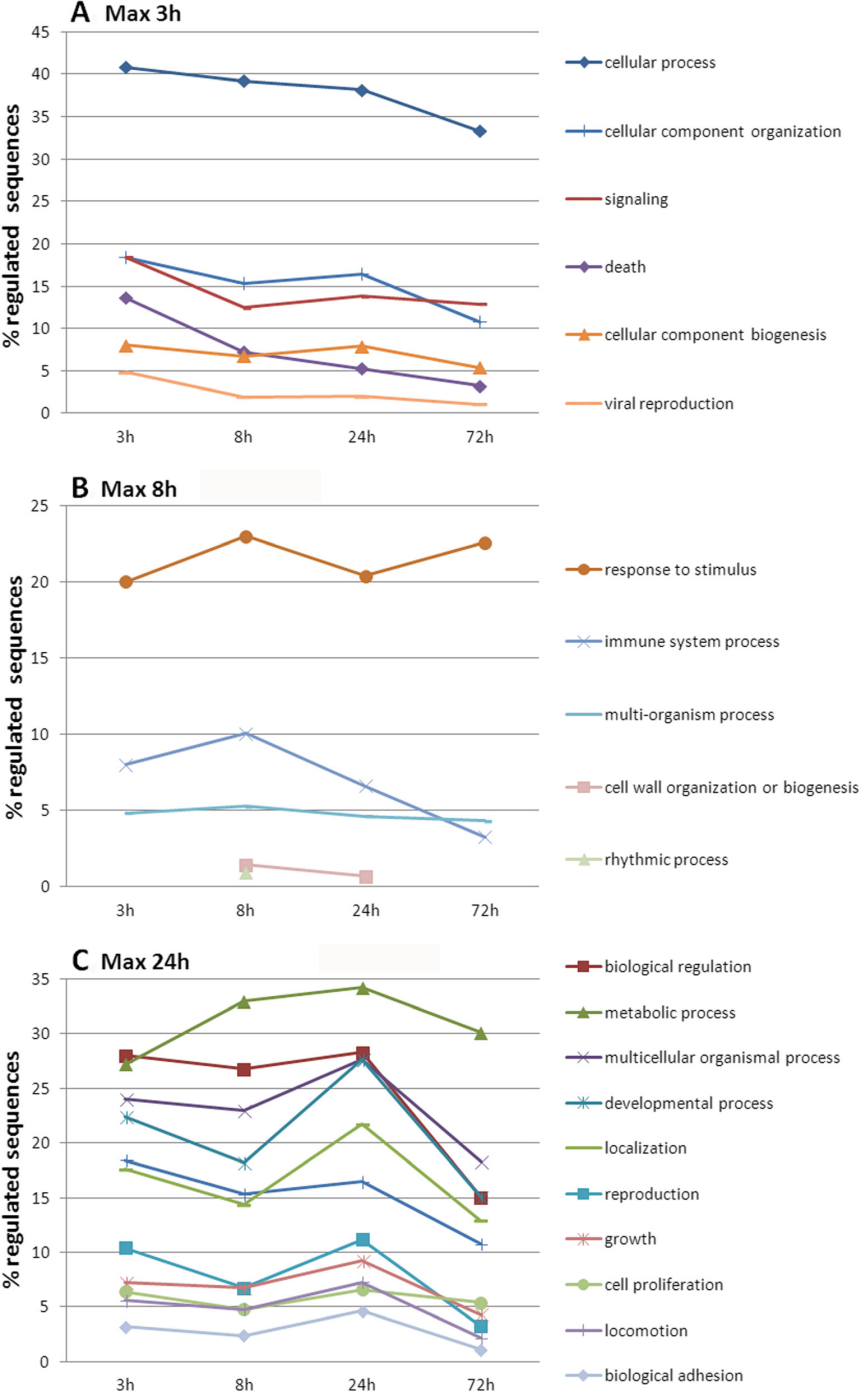
**Biological process GO terms change in regulated genes through time.** Numbers in abscissa refer to the percentage of GO term hits of the total of regulated sequences. GO terms with maximum representation in each sampling point are separately represented. **A**: 3 h, **B**: 8 h and **C**: 24 h. No GO processes exhibited a maximum representation 72 hours post-challenge.

The sequences of the regulated genes in each sampling time (t-test, p < 0.01) were analyzed in a Gene Ontology (GO) approach to cluster them into groups depending on the biological process in which the genes were involved and the time of maximum expression (Figure 4). Moreover, Table 2 shows the top 25 expressed genes in each sampling point. With this information, it is possible to infer the timing of the *R. philippinarum* response to a *V. alginolyticus* challenge. We have to take into account that the cumulative mortality rate at the end of the experiment, 72 hours, was 44%. The high mortality assures an infection but at the same time it is not too high allowing the clams to be able to fight against *Vibrio*.

**Table 2:** Top 25 expressed genes along the time course

**Sequence description**	**3 hpc FC**	**Sequence description**	**24 hpc FC**
Dynein light chain flagellar outer arm	48.91	Mariner mos1 transposase	13.2
Interleukin-17D	23.7	pdz and lim domain 5	6.31
Ras-related c3 botulinum toxin substrate 3	11.05	Zygote arrest protein 1	5.62
Barrier-to-autointegration factor	10.81	Monomeric sarcosine oxidase	5.57
Prothrombin	9.93	Cytochrome c-type heme lyase	5.45
Perlucin	7.94	Titin	5.27
Cubilin	6.23	Twitchin	4.77
Ets-related transcription factor elf-5	6.19	Pin2 terf1-interacting telomerase inhibitor 1	4.43
Pre-mrna-splicing factor cwc24	5.28	Leukocyte receptor cluster member 1 homolog	4.34
Adp-ribosyl cyclase	5	SART-3	4.19
Insoluble matrix shell protein 5	4.81	Myosin heavy striated muscle	4.03
MAPKKK1	3.92	Galaxin	3.45
Heat shock protein 70	3.91	Thap domain-containing protein 9	3.23
40s ribosomal protein s12	3.59	Calponin 3	3.09
HMG box transcription factor Sox17	3.57	Microtubule-associated protein futsch	2.94
LTBP-4	3.56	Nuclear speckle splicing regulatory protein 1	2.94
Histone acetyltransferase kat6a	3.54	Staphylococcal nuclease dom-cont protein 1	2.92
Calmodulin	3.46	Peroxisomal sarcosine oxidase	**−6.9**
Nucleolar complex protein 4 homolog	3.33	Mosc domain-containing protein mitochondrial	**−4.03**
Phosphoinositide 3-kinase adapter protein 1	3.27	Aldose 1-epimerase	**−3.88**
Cytochrome p450 2d6	3.21	Nuclease harbi1	**−3.8**
Electron transfer flavoprotein	**−67.28**	Lysocardiolipin acyltransferase 1	**−3.34**
Complement component c3	**−12.03**	Nuclear pore complex protein nup107	**−3.17**
Chaperonin	**−6.62**	Hamartin	**−3.09**
Collagen alpha-1 chain precursor	**−3.17**	Selectin	**−2.95**
**Sequence description**	**8 hpc FC**	**Sequence description**	**72 hpc FC**
Ganglioside gm2 activator	25.86	Mantle gene 6	52.66
Udp-glucose 4-epimerase	17.86	Serum amyloid a-3 protein precursor	19.53
Probable cubilin precursor	13.74	Elongation factor 1-gamma	7.95
Ets domain-containing protein elk-3	10.09	Biotin carboxylase	7.14
tnf receptor-associated factor 6	9.69	Kazal-type proteinase inhibitor protein	6.96
Nuclear factor nf-kappa-b p105 subunit	9.49	Abc transporter related protein	5.55
n-acylethanolamine-hydrolyzing acid amidase	9.44	Fizzy-related protein homolog	3.36
Pathogenesis-related protein 5	8.81	Nadh dehydrogenase 1 alpha subcomplex	2.47
Transmembrane protein 205	7.14	Sarcoplasmic calcium-binding protein	**−25.5**
Peptidyl-prolyl cis-trans isomerase b	6.64	High-affinity Na-dependent carnitine cotransporter	**−17.69**
Pro-neuregulin-2 membrane-bound isoform	6.42	Aragonite-binding protein	**−12.9**
Defense protein	5.56	Cytidine deaminase	**−12.29**
Peptidoglycan-recognition protein	4.94	Cat eye syndrome critical region protein 5	**−9.37**
Ficolin-2	4.85	Aldehyde cytosolic 2	**−8.3**
Cerebellin-3	**−46.98**	Lysozyme	**−7.67**
17-beta-hydroxysteroid dehydrogenase 14	**−18.9**	Glutamine synthetase/glutamate decarboxylase	**−6.71**
Complement c1q TNF-related protein 6	**−12.62**	Lambda-crystallin	**−5.72**
Ureidoglycolate hydrolase	**−12.39**	Organic cation transporter 1	**−5.17**
Sodium-dependent serotonine	**−12.02**	Dna-directed rna polymerase ii subunit rpb3	**−4.52**
Cytochrome p450 2u1	**−9.19**	Monocarboxylate transporter 5	**−4.14**
Organic cation transporter protein	**−7.44**	Cholinesterase	**−3.96**
Chitotriosidase	**−7.41**	Muscle lim protein 1	**−3.92**
Protein acn9 mitochondrial precursor	**−6.89**	Probable 4-hydroxy-2-oxoglutarate mitochondrial	**−3.46**
C1q domain containing protein	**−6.84**	Protein nlrc3-like	**−3.19**
N-acetylneuraminate lyase	**−5.53**	Beta-microseminoprotein	**−3.03**

**In bold**, downregulated transcripts.

In the first sampling point, 3 hours post-challenge, the results of the GO term analysis (Figure 4A) showed that most of the genes that were differentially expressed belonged to the cellular process category that involves many different functions. More specifically, one of the principal processes with a maximum expression at this sampling point was related to cellular component organization and biogenesis, leading to the assembly, disassembly or arrangement of the constituent macromolecules of the cell. Signaling and death processes were also present to trigger the response to the pathogen and eliminate compromised cells after an infection. Following the same line, the main functions of the top 25 hemocyte genes in the first sampling time were in accordance with the GO results. The most significantly upregulated gene 3 hpc (Table 2) is related to the cytoskeleton (dynein light chain) and could be involved in cellular spreading, chemotaxis and adhesion (RAC3, perlucin-like protein). However, the signaling and transcription processes (LTBP-4, phosphoinositide 3-kinase adapter protein 1, adp-ribosyl cyclase, HMG box transcription factor Sox17) could lead to a rapid triggering of several mechanisms, including the calcium-dependent processes that can lead to apoptosis via calmodulin activation [40]. Another upregulated gene was prothrombin, which is related to coagulation, but strikingly, there was an absence of the inflammatory response at the transcription level, as demonstrated by the strong inhibition of the complement factor C3.

In Figure 4B, 8 hours after challenge, the most represented functions are those related to response to stimulus and immune system. These processes initiate the response to the pathogen after the initial triggering of the signaling pathways. The GO term multi-organism process, with genes such as TRAF6, is also closely related because an interaction between clams and *V. alginolyticus* is implied. These results coincide with the information obtained in the top-list where genes involved in defense and wound healing (pathogenesis-related protein 5, defense protein precursor and pro-neuregulin-2 membrane bound isoform) or metabolism (udp-glucose 4-epimerase) are included. Several processes were maintained from 3 hours to 8 hours, such as transcription (NF-κB p105 subunit) or recognition (peptidoglycan-recognition protein sb1 precursor (PGRP) and fibrinogen-related protein (FREP)). The expression of FREPs agrees with results reported in the mussel after a bacterial infection in which the maximum expression was obtained after 6 hours post-infection [19]. This result means that while the signaling is maintained because of the pathogen adhesion and recognition, the immune response seems to be initiated. The three immune-related molecules with C1q domains in the list were found to be downregulated. This effect could occur because C1q up-regulation is a very fast mechanism in molluscs as suggested by Gestal *et al*. [41].

The major diversity of the processes found by the GO terms occurred one day after the *Vibrio* challenge (Figure 4C). General functions such as biological regulation or metabolism seemed to peak at this time point. Other processes such as development, localization (the process in which a cell, a macromolecule or an organelle is transported to and/or maintained in a specific location), reproduction, growth, cell proliferation, locomotion and biological adhesion were also important 24 hpc. These specific processes are closely related and could indicate the resolution of the challenge after the expression of the immune genes that were triggered at 8 hours. Locomotion and biological adhesion appeared together in the time course because these two processes are intimately related and lead to taxis and migration. In zebrafish, it is known that the chemotaxis of immune cells begins quickly and lasts for several days after an inflammatory stimulus (tailfin transection). Neutrophils are rapidly recruited with a maximum cell count at 6 h post injury, and the recruitment of macrophages progressively increases until at least 48 h after injury [42]. The temporal response of *R. philippinarum* hemocytes seems to be intermediate between these two vertebrate cell types. However, the cytoskeleton stays active, which suggests that locomotion is a very dynamic process with a high percentage of the top-regulated genes (Table 2, 24 hpc) such as titin, twitchin or calponin-3. In non muscular cells, as the hemocytes, titin has been shown to be related to chromosome condensation and segregation during mitosis [43]. This activity could be indicative of hemocyte proliferation and other upregulated processes, such as development. Furthermore, other functions are upregulated, such as transcription (staphylococcal nuclease domain-containing protein 1), or downregulated, such as metabolism or transport (aldose 1-epimerase, nuclear pore complex protein nup107).

Three days after the challenge, many genes were downregulated, suggesting negative feedback of the majority of the activated genes (Table 2, 72 hpc). Figure 4 also showed that the GO analysis could not find any processes with a maximum representation 72 hours post-challenge. Metabolism, adhesion, cytoskeleton modulation, transcription and defense (lysozyme) returned to basal levels, which was also illustrated by the general expression pattern shown in Figure 2. Curiously, the most upregulated gene, mantle gene 6, is related to the biomineralization of the shell [44]. This result could suggest that after the infection is controlled, the clams refocus the genetic machinery to other matters, such as shell repair. This result agrees with the GO analysis, and all of the biological functions began to stabilize three days after the bacterial challenge.

Because the defense against pathogens in molluscs relies on the innate immune system the response is expected to be fast, to overcome the infections as soon as possible. The contrary often means that the organisms will die. In fact, the critical point of the experiment seemed to be 8 hours after the challenge, when many immune genes were upregulated. Regulation of the apoptotic processes in hemocytes was also triggered early after the challenge and barely no genes related to apoptosis were found 3 days after the challenge. On the other hand, some effector genes such as C3, C1q, defense protein, and lysozyme seemed to exert their function before 24 hpc; cathepsins, proteases and protease inhibitors were up or downregulated throughout all the challenge pointing to complex interactions between bacteria and clams. These findings suggest that the survival or death of the animals is decided very early after the challenge, suggesting the importance of a fast triggering of the defense mechanisms.

### Enrichment analysis

**Figure 5:**
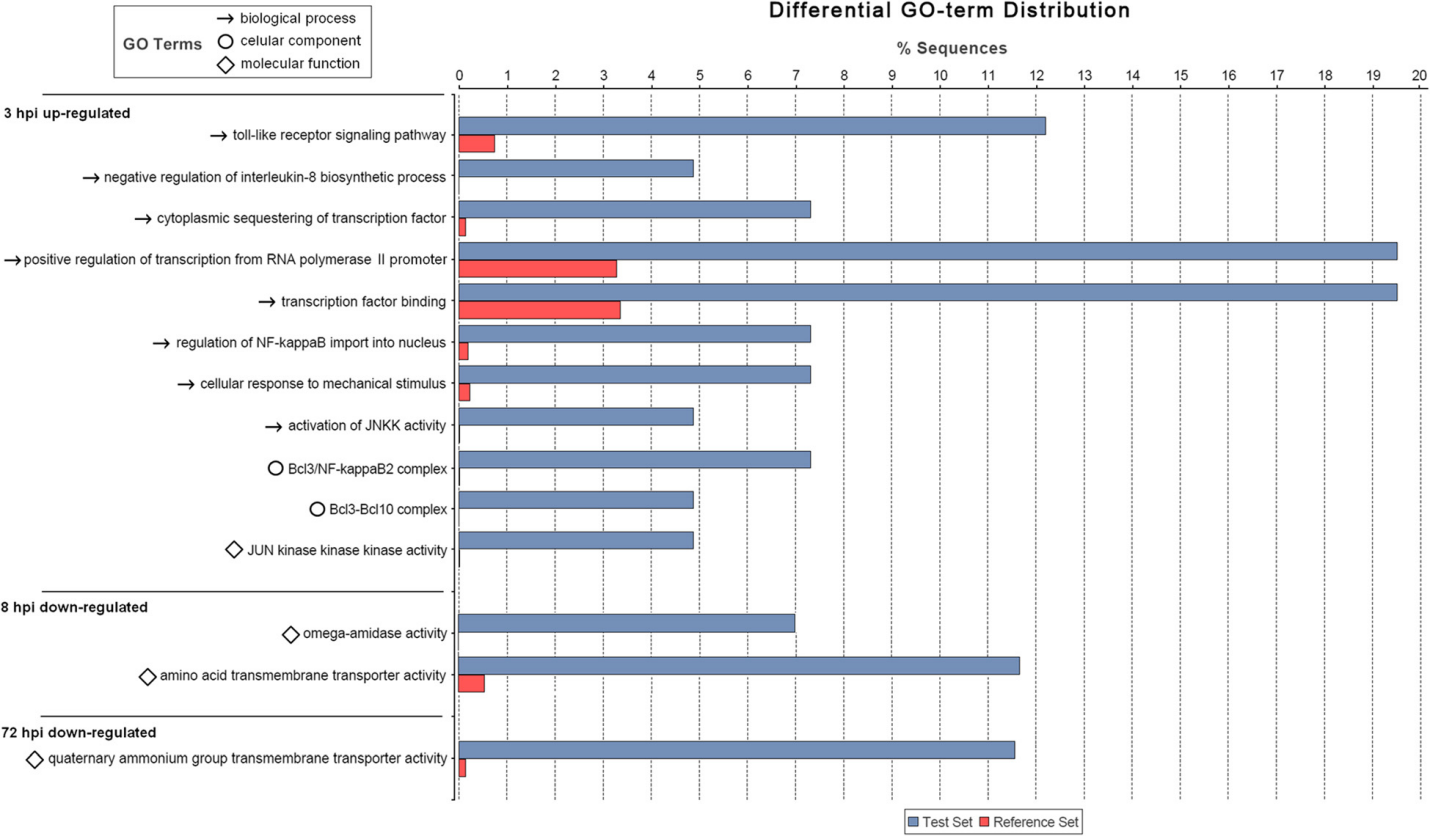
**Enrichment analysis results.** Differential GO term distribution between the test set (significantly expressed genes for each sampling point and regulation, up/down) and the reference set (all of the sequences present in the microarray). Only significant analyses were represented. hpc, hours post-challenge.

A Fisher’s exact test was performed to detect any significant enrichment of GO terms in each sampling point and regulation in comparison with the reference set of sequences present in the microarray. Only three analyses were enriched in specific GO terms compared with the entire microarray output: 3 hours upregulated (45 enriched GO terms), 8 hours downregulated (3 terms) and 72 hours downregulated (2 terms). After using the blast2GO tool to reduce the GO enriched terms, the most specific terms (the highest level GO terms of a parental line) were presented in Figure 5. It can be observed that the first significantly enriched processes of the upregulated genes were exclusively related to immune signaling and transcription, such as the toll-like receptor pathway or the regulation of NF-κB import to nucleus. An interesting result was the negative regulation of the IL-8 biosynthetic process, which is an indicator of an inhibition of the pro-inflammatory response. These results confirmed the previous findings of the GO annotation and the top-expressed genes, which showed an initial activation of the signaling and transcriptional processes and a strong inhibition of pro-inflammatory molecules such as C3, which seemed to exert their function much earlier than 3 hours after the stimulus, a trait that has been previously observed in bivalves [32].

The enrichment results at 8 hours and 72 hours post-challenge show that only downregulated processes related to metabolism and transport were found, but no specific enrichment of immunity genes was observed. These results imply the importance of an early response and an effective innate immune system in these bivalves.

### ANOVA analysis: effect of the challenge in all of the experimental data

An ANOVA was performed to globally analyze the information of the microarray experiments after the challenge. Although a vast majority of the genes were regulated at a single sampling point, the ANOVA results showed a total of 15 genes that were significantly regulated throughout the time course. Table 3 summarizes a description of the 10 successfully annotated genes with their respective fold changes. The expression profile of the ten genes coincides with the observed pattern in Figure 1: the maximum fold change is achieved 8 hours after the bacterial challenge (except for LTBP4, whose maximum expression comes out at 24 hours). This finding suggests that the highest modulation of expression happens at 8 hpc. Thus, that time point after the challenge could represent the actual time point in which the fate of the surviving and dying animals is set.

**Table 3:** **Differentially expressed genes detected by ANOVA and regulation after *V. alginolyticus *** **challenge**

	**3 h FC**	**8 h FC**	**24 h FC**	**72 h FC**
**Immune effectors**				
Latent-transforming growth factor beta-binding protein 4	3.55	43.50	217.29	3.49
Heat shock 70 kda protein	1.31	5.30	2.18	−1.39
Phosphoinositide 3-kinase adapter protein 1	3.27	5.56	2.06	−1.13
Tnf receptor-associated factor 6	2.47	9.69	2.36	−1.27
**Recognition/adhesion**				
Perlucin-like protein	7.94	39.44	29.44	19.91
Protein lin-7 homolog c	−1.29	−2.37	−2.26	−1.25
**Transcription**				
Nuclear receptor dax-1	2.18	4.02	2.85	1.06
Ets-related transcription factor elf-5	6.19	26.84	10.00	1.35
Nuclear factor nf-kappa-b p50 subunit	1.74	9.48	1.89	−1.33
A20-binding inhibitor of nf-kappa-b activation 2	1.98	3.37	1.77	−1.13

These ten genes have important defense, protective and homeostasis functions, including cell death and apoptosis, that are key events to decide survivorship of the challenged clams: The phosphoinositide 3-kinase adapter protein 1 (PIK3AP1 or BCAP) links Toll-like receptor signaling and PI3K activation, preventing excessive inflammatory cytokine production and contributing to B-cell development in humans [45,46], this could mean that as the same time that defense cells produce an immune response, this is being controlled to avoid self-damage by an uncontrolled inflammatory reaction. The nuclear receptor dax-1 (NR0B1) is a co-regulatory inhibitor of the transcriptional activity of other nuclear receptors. NR0B1 is also related to the development and maintenance of stem cell pluripotency and lipogenesis and gluconeogenesis [47], important processes to maintain the energy homeostasis necessary to overcome an infection and regenerate damaged tissues. LTBP-4, described in the validation of the microarray, modulates TGF-β activity and is related to cell adhesion and migration. The perlucin-like protein is a constitutive and regulating C-type lectin that promotes calcium carbonate precipitation and crystallization. Perlucin contains a carbohydrate recognition domain, and because this protein is expressed both in the mantle and in the gills and the digestive tract, it has been suggested that perlucin could play a role in non-self antigen recognition, similarly to other C-type lectins to trigger the immune response [48]. The upregulation of heat shock proteins (HSPs) represents an important mechanism in the stress response and their functions are closely linked to the innate immune system. Although the primary role of HSP70 is to function as a molecular chaperone, it inhibits the mitochondrial apoptosis pathway as well [49], a signal of the changes being produced after the *Vibrio* challenge and the survival efforts of hemocytes to overcome the infection. TRAF6, which has been described previously, is a signaling molecule that leads to the activation of the NF-κB and AP-1 transcription factors implicated in the regulation processes such as cell proliferation and survival, growth, differentiation, apoptosis, cell migration or metabolism; all of them closely related to immunity and response to a pathogen. The protein lin-7 homolog c establishes and maintains the distribution of channels and receptors at the plasma membrane of epithelial cells [50] and is likely to be involved in the formation of the cadherin-independent tight junction-like structure in epithelial cells and in the synapses in mammals [51], suggesting a role in establishment of the hemocytes adhesion to other cells and maybe intercellular signal transductions after the *Vibrio* challenge. The last three genes are a group related to transcription: the ets-related transcription factor elf-5, a transcriptional activator restricted to epithelial cells and involved in the keratinocyte differentiation in mammals [52], the NF-κB p50 subunit and the a20-binding inhibitor of NF-κB activation 2, two molecules that are implicated in the NF-κB signaling pathway, which controls many inflammatory and immune responses, cell proliferation, apoptosis and metabolism [53,54]. These molecules are closely involved in defense against pathogens, from recognition to effector functions in the immune system. Additionally, cell proliferation, survival or death were very represented processes, showing that apoptotic/antiapoptotic molecules are highly expressed after a *Vibrio* challenge, indicative of the active fight of the hemocytes.

### Functional immune response of hemocytes after bacterial infection

**Figure 6:**
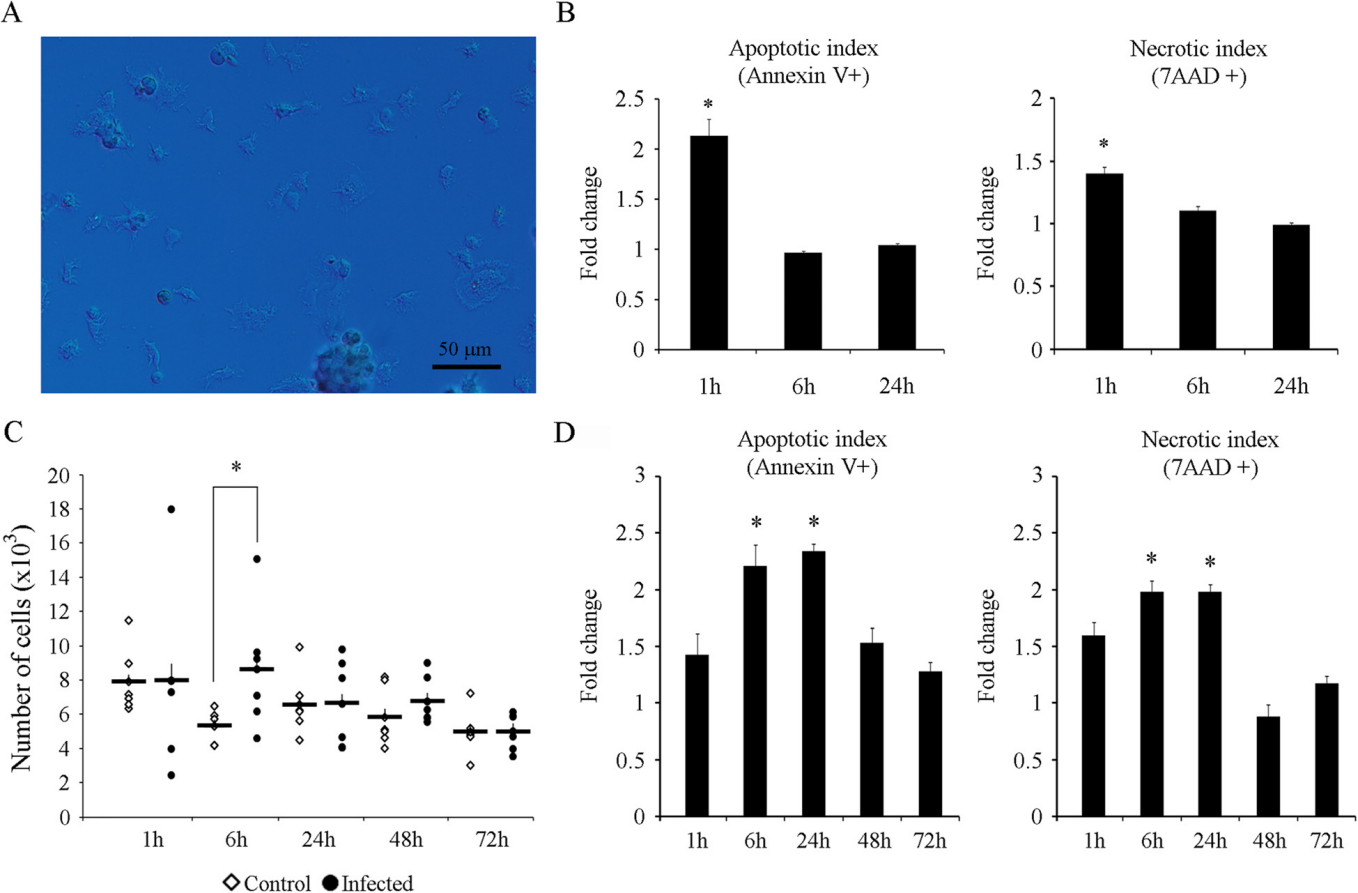
**Evaluation of the immune response after bacterial infection. (A)** The cellular components of the hemolymph are granulocytes and hyalinocytes. Photographs were taken using Nomarski DIC objective. **(B)** Apoptotic and necrotic levels induced by *V. alginolyticus* in primary cultures of hemocytes (n = 6). **(C)***In vivo* modulation of the cellular concentration in hemolymph after bacterial infection. A significant increment in the number of cells was observed 6 h after infection. Graph represents the measurement of 6 independent animals as well as the mean + SEM. **(D)** Apoptotic and necrotic levels *in vivo* induced by *V. alginolyticus* at different times post infection. In all graphs, bars represent the mean + SEM of 6 independent measurements. Data were analyzed using the Student’s t-test. (*) significant differences at p < 0.05.

To finally evaluate if the changes in gene expression corresponded to functional parameters, the immune response triggered by the *V. alginolyticus* infection was evaluated. In primary cultures of clam hemocytes (granulocytes and hyalinocytes) (Figure 6A), the *in vitro* infection with *V. alginolyticus* induced apoptotic and necrotic cell death 1 h after infection (Figure 1B). This functional result agrees with the GO analysis showing that most significantly upregulated genes at the beginning of the infection could be involved in chemotaxis, adhesion and therefore they lead to a rapid triggering of several mechanisms, such as apoptosis.

Results obtained after the intramuscular injection of *V. alginolyticus* also supported the previous findings of the GO annotation and the top-expressed genes. This bacterial infection induced significant changes in the cell concentration in hemolymph extracted from the site of infection. This parameter is an indirect indicator of cell migration to the site of infection. As soon as 6 h after infection the cell concentration registered in infected animals was significantly higher than that registered in controls. Cell migration seemed to be a quickly response since no significant variations were registered at 24, 48 and 72 h (Figure 6C). *V. alginolyticus* was also able to induce cell death after *in vivo* infections, although the timing for induction was different to that observed in cell cultures. A significant increment in the number of necrotic and apoptotic cells was registered at 6 and 24 h post-infection (Figure 6D).

## Conclusions

The current work presents the second version of the *R. philippinarum* oligo-microarray enriched with immune sequences from hemocytes. We have analyzed the response of the Manila clam, *R. philippinarum*, after a *V. alginolyticus* challenge, strain TA15, and found almost 600 differentially expressed genes out of 13,671 probes. After the expression, GO term analyses and functional immune studies, we were able to establish a putative timing for a *Vibrio* infection in clams: there was an early response (3 hours) of hemocytes against the *Vibrio* challenge in which the main functions of hemocytes were mainly related to cellular component organization, biogenesis, cell migration, signaling and death. These functions were a fast response to the pathogen with related genes such as IL-17 or calmodulin. Eight hours seemed to be a key point in time to overcome the bacterial challenge, as the most important functions were related to response to stimulus, the immune system process and the multi-organism process to directly initiate the defense. Genes such as PGRPs, FREPs and defense proteins were present at this sampling point. After one day, there was a modulation of a great number of functions, from general processes such as metabolism to more specific functions such as development, localization and locomotion. Many cytoskeleton genes were found between the top-expressed genes, which is indicative of a possible chemotactic response in hemocytes. Finally, after 72 h, the number of regulated genes decreased substantially, indicating a stabilization of the status of the clam and the end of the cellular response to the challenge. We have to take into account that the modulation of the genes can be the result of an effective immune response but also a symptom of a future death. In fact, several apoptotic and anti-apoptotic genes were regulated. This highlights that the obtained transcriptomic profile is a reflection of a complex interaction between the host immune system and the pathogen that deserves further studies.

In summary, bivalves are able to respond quickly against an infection with an orchestrated modulation of different genes. The immune-enriched oligo-microarray for *R. philippinarum* has proven to be useful in hemocyte expression analysis, and this tool has yielded interesting results.

## Methods

### Sequence assembly, annotation and microarray design

A total of 1,438,665 sequences from *R. philippinarum* were collected from different origins: 454 sequencing and Sanger sequencing [20,21] and available ESTs in the NCBI database. Table 1 details the number of sequences by origin, assembly and annotation criteria. After removing low-quality sequences and filtering for adaptors and primers, the cured sequences were assembled using the Newbler software package (GS *De Novo* Assembler v2.6, Roche). BlastX and BlastN algorithms [55] (http://blast.ncbi.nlm.nih.gov/Blast.cgi) were used to annotate the selected sequences. Alignments with an e-value threshold of 10^−3^ and 10^−5^ were considered significant for protein and nucleotide databases, respectively. The reference databases that were used are NCBI SwissProt, NCBI Metazoan RefSeq, NCBI nonredundant and ENSEMBL databases, and the dedicated databases for *B. glabrata* (http://www.snaildb.org/) and *L. gigantea* (http://genome.jgi-psf.org/Lotgi1/Lotgi1.download.html).

Probe design was carried out using the Agilent eArray interface (https://earray.chem.agilent.com/earray/), which applies proprietary prediction algorithms to design 60-mer oligoprobes.

Microarrays were synthesized *in situ* using the Agilent ink-jet technology with an 8 × 15 K format. Each array included default positive and negative controls. Probe sequences and other details on the microarray platform can be found in the GEO database (http://www.ncbi.nlm.nih.gov/geo/) [56] under accession number GPL16450. The GEO accession for the series data (microarray gene expression data) is GSE43274.

### Animal sampling and RNA isolation

*R. philippinarum* clams were obtained from a commercial shellfish farm (Vigo, Galicia, Spain). Clams were maintained in open circuit filtered sea water tanks at 15°C with aeration and were fed daily with *Phaeodactylum tricornutum* and *Isochrysis galbana.* Prior to the experiments, the clams were acclimatized to the aquarium conditions for one week.

Clams (n = 100) were notched in the shell and injected in the muscle with 100 μl of 10^8^ CFU/ml of *Vibrio alginolyticus*, strain TA15. The inoculation dose was chosen according to previous *Vibrio* challenges in *R. philippinarum*[5,18,57]. Controls (n = 100) were injected with 100 μl of PBS. After challenge, the clams were returned to the tanks and maintained at 15°C until sampling at 3, 8, 24, and 72 hours after challenge. The cumulative mortality rate at the end of the experiment, 72 hours, was 44%. The controls showed an end point mortality of 10%.

Hemolymph (1 ml) was withdrawn from the adductor muscle of the clams with a 0.5 mm diameter (25 G) disposable needle. Hemolymph from four individuals was pooled, and five biological replicates were taken at each sampling point. The hemolymph was centrifuged at 3000 g for 10 minutes at 4°C. The pellet was resuspended in 250 μl of TRIzol (Invitrogen). Total RNA isolation was conducted following the manufacturer’s specifications in combination with the RNeasy Mini kit (Qiagen) for RNA purification after DNase I treatment. Next, the concentration and purity of the RNA were measured using a *NanoDrop ND1000* spectrophotometer. Finally, RNA integrity was tested on an Agilent 2100 Bioanalyzer (Agilent Technologies). Only the samples with high RNA quality were used for labeling and hybridization, 3 to 5 biological replicates were used for each condition and time.

### Cy3 labeling

Sample labeling and hybridization were performed according to the Agilent One-Color Microarray-Based Gene Expression Analysis protocol. Briefly, 100 ng of RNA from each RNA sample was amplified and labeled with Cy3 using the Low Input Quick Amp labeling kit (Agilent Technologies), according to the manufacturer’s instructions. Each sample included a mixture of 10 different viral poly-acetylated RNAs (Agilent Spike-In Mix) before amplification and labeling so the microarray analysis work-flow could be assessed. Qiagen RNeasy mini spin columns were used to purify amplified RNA. Finally, amplification and dye incorporation rates were verified using a *NanoDrop ND1000* spectrophotometer. These values should be between 200 and 500 ng/μL (RNA concentration) and between 20 and 50 pmol/μg aRNA (dye incorporation).

### Microarray hybridization and analysis

Cy3 labeled RNA (600 ng) was fragmented with 5 μl of 10× Blocking Agent and 1 μl of 25× Fragmentation Buffer at 60°C for 30 min. Finally, 55 μl of 2× GE Hybridization buffer was added to dilute the fragmented RNA. The eight spaces of the gasket slide were filled with 40 μl of the correspondent hybridization solution and then assembled on the microarray slide (each slide contained eight arrays). Slides were incubated for 17 h at 65°C in an Agilent Hybridization Oven, subsequently removed from the hybridization chamber, quickly submerged in GE Wash Buffer 1 to disassemble the slides and then washed in GE Wash Buffer 1 for 1 minute followed by one additional wash in pre-warmed (37°C) GE Wash Buffer 2.

Hybridized slides were scanned at 5 μm resolution using an Agilent G2565BA DNA microarray scanner. Default settings were modified to scan the same slide at two different sensitivity levels (XDR Hi 100% and XDR Lo 10%). The two linked images generated were analyzed together, the data were extracted, and the background was subtracted using the standard procedures in the Agilent Feature Extraction Software version 9.5.1. The software returned a series of spot quality measures to evaluate the goodness and the reliability of the spot intensity estimates. After ensuring that all of the microarrays passed the quality tests (spatial distribution of median signals for row and column, distribution of the spot signal, spatial distribution of all outliers of the array, coefficient of variation of the technical replicates of the SpikeIn and the regression correlation between signal and concentration) (Additional file 2: 1–36), control features (positive, negative, etc.), except for SpikeIn Viral RNAs, were excluded from subsequent analyses. SpikeIn control intensities were used as internal controls and were expected to be uniform across the experiments of a given dataset.

The GeneSpring software (Agilent) was used to normalize and analyze the microarray fluorescence data. To identify regulated genes, two statistical analyses in filtered raw data (20 - 90^th^ Percentile) were carried out: a t-test (p < 0.01) and a two-way ANOVA (p < 0.05) with a Benjamini-Hochberg multiple testing correction. The t-test was used to find the genes that were significantly different between the controls and the infected samples at each sampling point. Genes with a fold change between ±1.5 were not used for further investigation. ANOVA analysis was performed to analyze the whole dataset taking into account the challenge along all the sampling times.

### GO terms and enrichment analysis

After statistical analysis, blast2GO software [58] was used to assign GO terms [59] to the significantly expressed genes (t-test, p < 0.01) through the time course. Default values (annotation cutoff = 55, GOweight = 5) in blast2GO were used to perform the analysis and biological process ontology level 2 was selected.

The enrichment analyses were made with the total microarray information as the reference set and each sampling time (3 h, 8 h, 24 h, 72 h) and regulation (up or down) as the test sets. Then, Fisher’s exact test was run with default values: a one-tailed test without removing double IDs and 0.05 false discovery rate (FDR) cut-off. To construct Figure 5, the blast2GO option to show only the most specific terms (0.05 FDR cut-off) was performed.

### Validation of the microarray

Specific PCR primers were designed from the sequences of the selected probes (Table 4) using the *Primer3* program [60] according to qPCR restrictions. *Oligo Analyzer 1.0.2* was used to check for dimer and hairpin formation. The efficiency of each primer pair was then analyzed with seven serial five-fold dilutions of cDNA of *R. philippinarum* and was calculated from the slope of the regression line of the quantification cycle versus the relative concentration of cDNA [61]. A melting curve analysis was also performed to verify that only specific amplification occurred and that no primer dimers were amplified. If these conditions were not satisfied, new primer pairs were designed.

**Table 4:** Primers designed for the microarray validation

**Primer name**	**Sequence 5′ → 3′**	**Tm**	**Product size**	**Ct slope**	**Acc. n°/Probe name**
Clam 18S F	CCGAACATCTAAGGGCATCA	60.12	169 bp	−3.0	EF426293.1
Clam 18S R	AGTTGGTGGAGCGATTTGTC	60.99			
C3 F	CCCAGGTGCCAAAGAACA	55.56	162 bp	−3.5	S_isotig17547_isogroup06014
C3 R	GCGGGGTACACATACTCGTC	60.00			
IL-17D F	CTCAAAAAGACTCACAGGGAATG	60.15	186 bp	−3.8	P_isotig09595_isogroup01099
IL-17D R	CTGGCAATGATGTACTGTCGTAA	60.07			
LTBP-4 F	TAATCATTGCCGCCTTATCG	60.92	188 bp	−3.6	S_isotig21547_isogroup10014
LTBP-4 R	GCGACCTGAATCAAATTCGT	60.08			
TRAF6 F	GCCAACATAGTAGCTCAGGAACA	60.68	148 bp	−3.4	P_isotig17929_isogroup06396
TRAF6 R	TTCCAATATAGCTTACAACATCAACA	59.08			

The cDNA synthesis was performed on 1 μg of total RNA using SuperScript™ III Reverse Transcriptase (Invitrogen) following the manufacturer’s protocol. The same RNAs used for hybridization of the microarrays were used for the retrotranscription.

Real-time quantitative PCR was performed in the *7300 Real Time PCR System* (Applied Biosystems). One microlitre of fivefold-diluted cDNA template was mixed with 0.5 μl of each primer (10 μM) and 12.5 μl of SYBR Green PCR master mix (Applied Biosystems) in a final volume of 25 μl. The standard cycling conditions were 95°C for 10 min, followed by 40 cycles of 95°C for 15 seconds and 60°C for 30 seconds. All reactions were performed as technical triplicates, and an analysis of melting curves was performed in each reaction. The relative expression levels of the genes were normalized using 18S as a reference gene, which was constitutively expressed and not affected by the Vibrio challenge, following the Pfaffl method [61].

### Functional immune response of hemocytes after bacterial infection

The immune response triggered after *V. alginolyticus* infection was evaluated through *in vitro* as well as *in vivo* experiments. Flow cytometry was used to evaluate different immune parameters such as cell migration and bacterial-induced apoptosis.

For *in vitro* experiments hemolymph from 12 animals were diluted 1:1 in ice-cold filtered sea water to prevent aggregation and dispensed (200 μl) into 24 wells plates. After 30 min of incubation at 15°C in the dark for cell adhesion, cells were infected with 200 μl of the TA15 strain *V. alginolyticus* (10^8^ CFUs/ml) and maintained at 15°C. Induction of apoptosis was evaluated 1 h, 6 h and 24 h after infection in six different cell cultures. Supernatants were removed and 500 μl of ice-cold binding buffer (10 mM Hepes/NaOH, pH 7.4, 140 mM NaCl, 2.5 mM CaCl_2_) containing 5 μl Annexin V-FITC (BD Biosciences) and 10 μl of 7-amino-actinomycin D (7-AAD, BD Biosciences) were added. Cells were incubated for 15 min in the dark and analyzed in a FACSCalibur flow cytometer using Cell Quest software (BD Biosciences). The apoptotic process was evaluated using the FITC and 7-AAD content detected in the FL-1 (530 nm) and FL-3 (650 nm) channels, respectively. Fold units were calculated by dividing the percentage of FL-1 positive hemocytes obtained after infection by the values recorded in the control group. In all experiments data were analyzed using the Student’s t-test (p < 0.05). Fresh hemocytes were visualized using an Eclipse 80i light microscopy (Nikon) with Nomarski DIC prism to enhance the contrast.

For *in vivo* experiments 40 clams were intramuscular injected with 100 μl of the TA15 strain *V. alginolyticus* (4×10^10^ CFUs/ml). Control animals were injected with the same volume of filtered sea water. Animals were maintained in closed-circuit aquarium at 15°C. Hemolymph was extracted from 6 infected and 6 control animals at 1, 6, 24, 48 and 72 h after infection. Samples were immediately diluted 1:1 in ice-cold binding buffer and stained with Annexin V-FITC (BD Biosciences) and 7-amino-actinomycin D (7-AAD, BD Biosciences). Cells were incubated for 15 min in the dark and analyzed by flow cytometry as previously described. Cell migration to the site of infection was estimated by measuring the cell concentration in hemolymph extracted from the infected muscle.

### Availability of supporting data

Microarray data are deposited in the public functional genomics data repository GEO (Gene Expression Omnibus):

– Microarray platform:

https://www.ncbi.nlm.nih.gov/geo/query/acc.cgi?acc=GPL16450

– Microarray gene expression data:

https://www.ncbi.nlm.nih.gov/geo/query/acc.cgi?acc=GSE43274

## Competing interests

The authors declare that they have no competing interests.

## Authors’ contributions

BN and AF conceived and designed the experiments. RM prepared the samples. LB, MM, MB and RM made the assembly and annotation of the sequences and designed the microarray platform. RM and MM hybridized the microarrays. AR performed functional immune assays. BN, AF, PB, AR and RM analyzed the data. RM wrote the paper. All authors read and approved the manuscript.

## Supplementary Material

Additional file 1 **Description of the reference databases used to annotate *R. philippinarum* sequences.** Click here for file

Additional file 2 Quality control of each microarray experiment provided by the Agilent Feature Extraction Software. Click here for file
