# Supplementary material for: Gene expression profile analysis of Manila clam (Ruditapes philippinarum) hemocytes after a Vibrio alginolyticus challenge using an immune-enriched oligo-microarray
Source: BMC Genomics. 2014 Apr 7;15:267. doi: 10.1186/1471-2164-15-267 (PMC4234419; doi:10.1186/1471-2164-15-267)
Supplement: Additional file 2 — Quality control of each microarray experiment provided by the Agilent Feature Extraction Software. [file 1471-2164-15-267-S2.pdf]

QC Report - Agilent Technologies : 1 Color Gene Expression

Date	Friday, September 02, 2011 - 10:55	Grid	035923_D_F_20110809
Image	UniversityPadova_253592310001_S01_H [1_1]	BG Method	No Background
Protocol	GE1-v5_95_Feb07 (Read Only)	Background Detrend	On(FeatNCRange, LoPass)
User Name	Administrator	Multiplicative Detrend	True
FE Version	9.5.1.1	Additive Error	1(Green)
		Saturation Value	595942 (g)

Spot Finding of the Four Corners of the Array

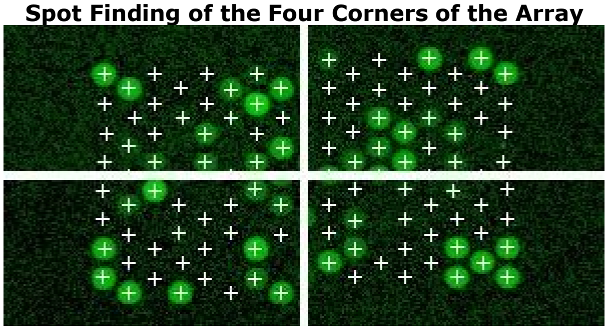

Grid Normal

	Feature	Local Background
	Green	Green
Non Uniform	0	34
Population	22	250

Spatial Distribution of All Outliers on the Array

192 rows x 82 columns

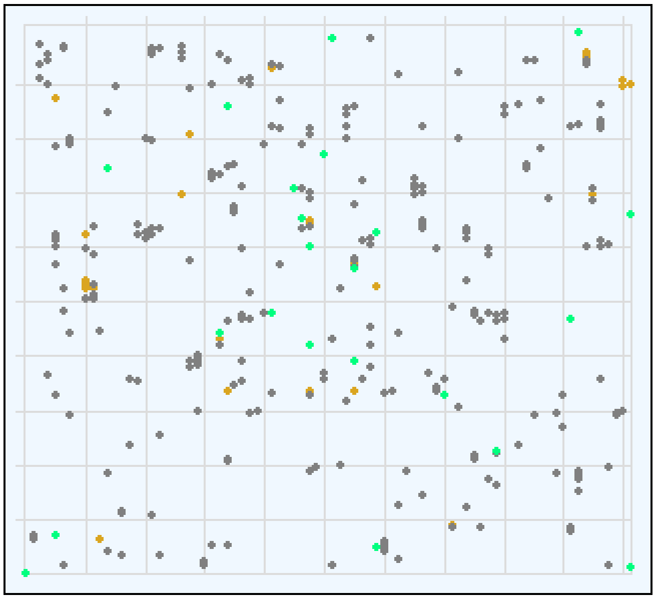

FeatureNonUnif (Green) = 0(0.00%)

GeneNonUnif (Green) = 0 (0.000 %)

BG NonUniform BG Population

Green FeaturePopulation Green Feature NonUniform

Negative Control Stats

	Green
Average Net Signals	10.62

Net Signal Statistics

Agilent SpikeIns:

	Green
# Saturated Features	0
99% of Sig. Distrib.	183054
50% of Sig. Distrib.	506
1% of Sig. Distrib.	11

Non-Control probes:

	Green
# Saturated Features	0
99% of Sig. Distrib.	33064
50% of Sig. Distrib.	24
1% of Sig. Distrib.	9

Histogram of Signals Plot

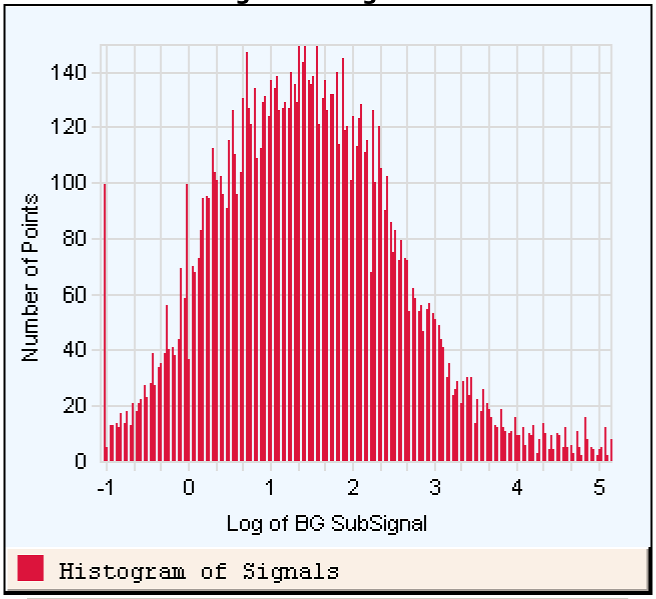

Features (NonCtrl) with BGSubSignal < 0: 2331 (Green)

StdDev Net Signals	1.34
Average BG Sub Signal	-1.41
StdDev BG Sub Signal	1.31

Local Bkg (inliers)

	Green
Number	13929
Avg	42.98
SD	1.81

Foreground Surface Fit

	Green
RMS_Fit	0.45
RMS_Resid	1.45
Avg_Fit	40.68

Multiplicative Surface Fit

	Green
RMS_Fit	0.11

Reproducibility: %CV for Replicated Probes

	Median %CV Signal (inliers)	
	Non-Control probes	Agilent SpikeIns
	Green	Green
BGSubSignal	-1.00	12.07
ProcessedSignal	-1.00	5.39

Agilent SpikeIns Signal Statistics

Probe Name	Log (Relative Conc.)	Median (Log Proc. Sig.)	% CV	StdDev
(+)E1A_r60_3	0.30	0.19	47.09	0.13
(+)E1A_r60_a104	1.30	0.48	42.87	0.19
(+)E1A_r60_a107	2.30	1.20	23.03	0.12
(+)E1A_r60_a135	3.30	2.11	9.30	0.04
(+)E1A_r60_a20	3.83	2.58	5.18	0.02
(+)E1A_r60_a22	4.30	3.05	4.35	0.02
(+)E1A_r60_a97	4.82	3.74	5.39	0.02
(+)E1A_r60_n11	5.30	4.28	4.50	0.02
(+)E1A_r60_n9	5.82	4.62	8.43	0.04
(+)E1A_r60_1	6.30	5.24	6.00	0.03

Agilent SpikeIns: %CV of Avg. Processed Signal Plot

Spatial Distribution of Median Signals for each Row

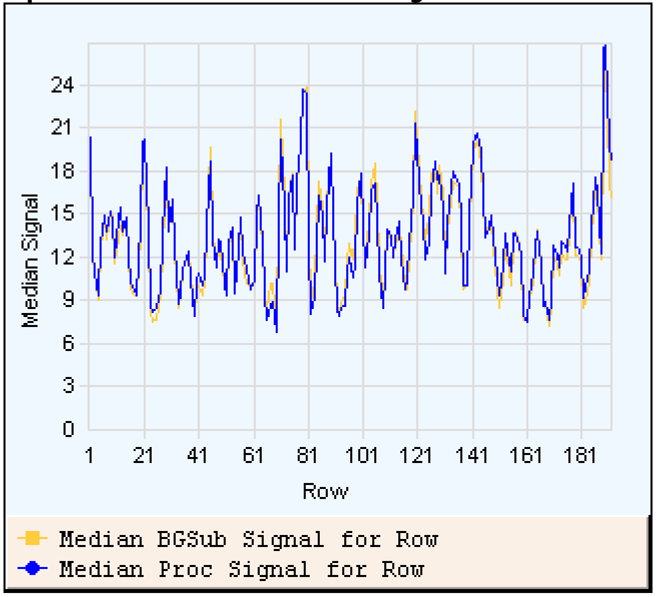

Spatial Distribution of Median Signals for each Column

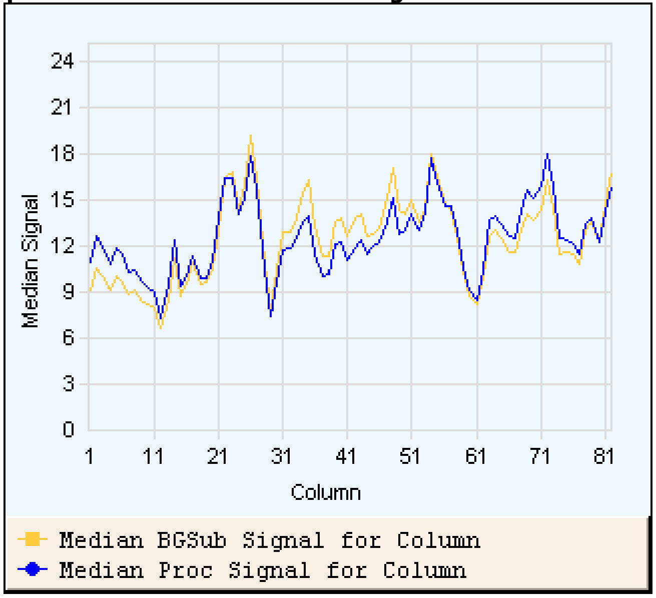

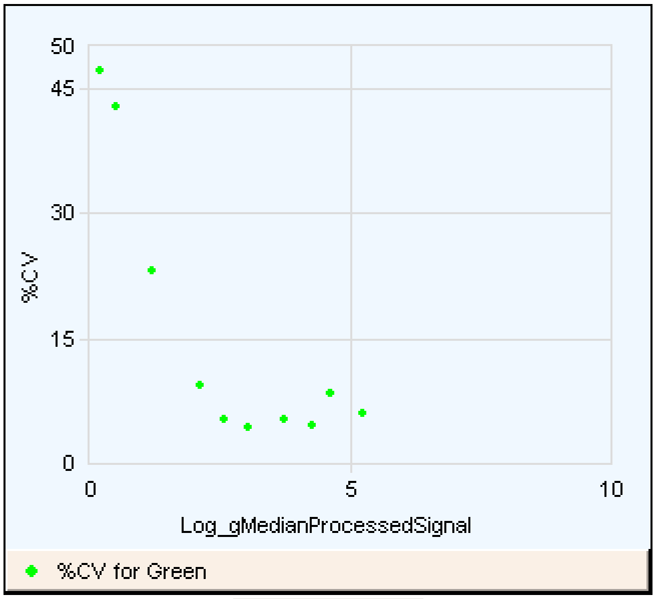

Median %CV:5.39

Agilent SpikeIns: Log(Signal) vs. Log(Relative concentration) Plot

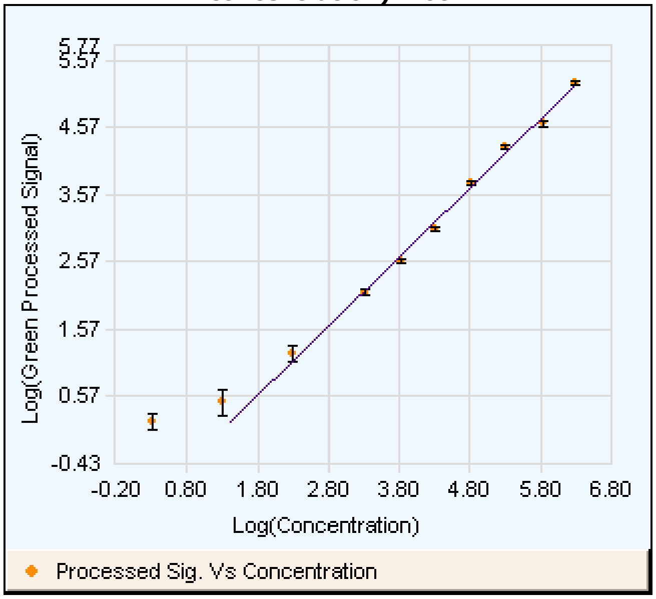

Agilent Spike-In Concentration-Response Statistics

Linear Range Statistics:

Low Signal	0.20
High Signal	5.72
Low Relative Concentration	1.40
High Relative Concentration	6.82
Slope	1.02
R^2 Value	1.00

Signal Detection Limit Statistics

Saturation Point	5.78
Low Threshold	-0.01
Low Threshold Error	0.23
Spike-In Detection Limit	0.65

QC Report - Agilent Technologies : 1 Color Gene Expression

Date	Tuesday, September 20, 2011 - 11:05	Grid	035923_D_F_20110809
Image	UniversityPadova_253592310003_S01_H [2_3]	BG Method	No Background
Protocol	GE1-v5_95_Feb07 (Read Only)	Background Detrend	On(FeatNCRange, LoPass)
User Name	Administrator	Multiplicative Detrend	True
FE Version	9.5.1.1	Additive Error	3(Green)
		Saturation Value	588400 (g)

Spot Finding of the Four Corners of the Array

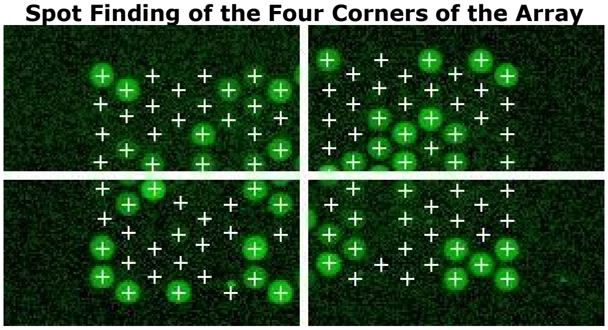

Grid Normal

	Feature	Local Background
	Green	Green
Non Uniform	2	43
Population	12	267

Spatial Distribution of All Outliers on the Array

192 rows x 82 columns

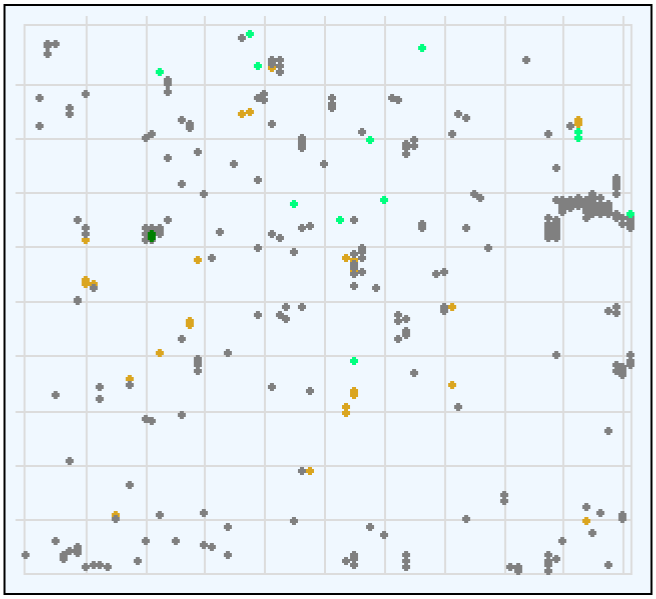

FeatureNonUnif (Green) = 2(0.01%)

GeneNonUnif (Green) = 2 (0.015 %)

BG NonUniform BG Population

Green FeaturePopulation Green Feature NonUniform

Negative Control Stats

	Green
Average Net Signals	15.81

Net Signal Statistics

Agilent SpikeIns:

	Green
# Saturated Features	0
99% of Sig. Distrib.	257155
50% of Sig. Distrib.	637
1% of Sig. Distrib.	12

Non-Control probes:

	Green
# Saturated Features	0
99% of Sig. Distrib.	52283
50% of Sig. Distrib.	41
1% of Sig. Distrib.	12

Histogram of Signals Plot

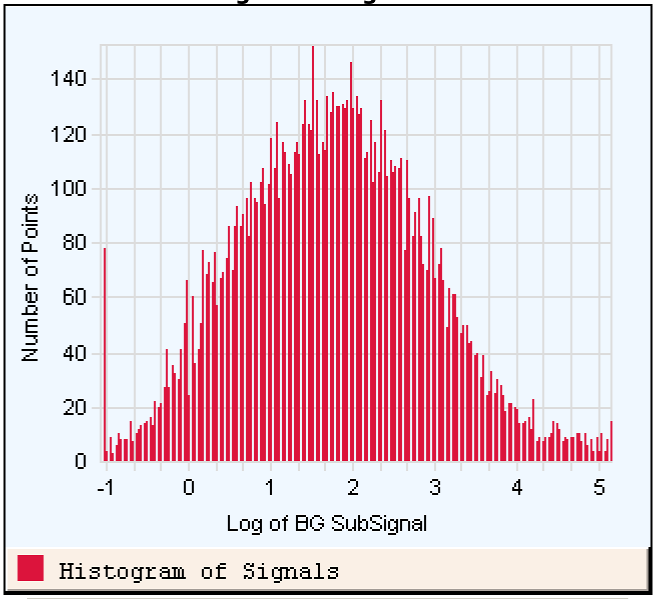

Features (NonCtrl) with BGSubSignal < 0: 2525 (Green)

StdDev Net Signals	3.05
Average BG Sub Signal	-2.08
StdDev BG Sub Signal	1.94

Local Bkg (inliers)

	Green
Number	13912
Avg	43.87
SD	2.27

Foreground Surface Fit

	Green
RMS_Fit	2.43
RMS_Resid	2.64
Avg_Fit	47.13

Multiplicative Surface Fit

	Green
RMS_Fit	0.11

Reproducibility: %CV for Replicated Probes

	Median %CV Signal (inliers)	
	Non-Control probes	Agilent SpikeIns
	Green	Green
BGSubSignal	-1.00	12.03
ProcessedSignal	-1.00	3.56

Agilent SpikeIns Signal Statistics

Probe Name	Log (Relative Conc.)	Median (Log Proc. Sig.)	% CV	StdDev
(+)E1A_r60_3	0.30	0.43	10.28	0.04
(+)E1A_r60_a104	1.30	0.66	95.92	0.28
(+)E1A_r60_a107	2.30	1.36	15.66	0.07
(+)E1A_r60_a135	3.30	2.29	3.65	0.02
(+)E1A_r60_a20	3.83	2.71	3.64	0.02
(+)E1A_r60_a22	4.30	3.13	3.56	0.02
(+)E1A_r60_a97	4.82	3.92	3.96	0.02
(+)E1A_r60_n11	5.30	4.45	2.63	0.01
(+)E1A_r60_n9	5.82	4.84	2.37	0.01
(+)E1A_r60_1	6.30	5.37	3.52	0.02

Agilent SpikeIns: %CV of Avg. Processed Signal Plot

Spatial Distribution of Median Signals for each Row

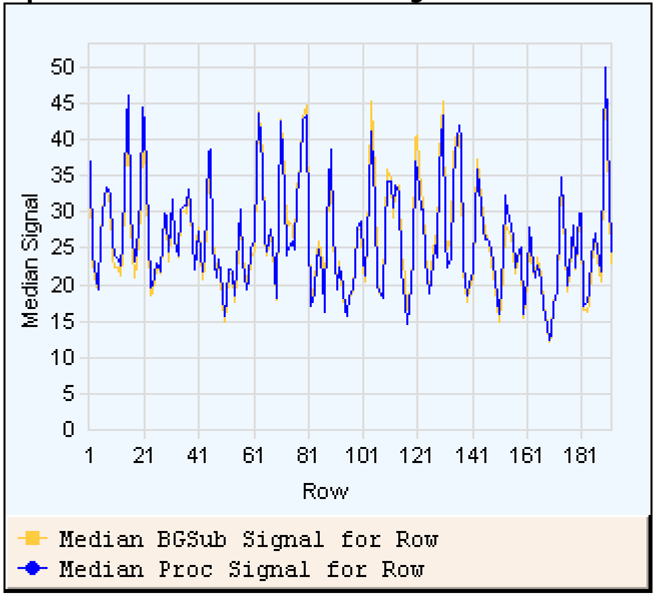

Spatial Distribution of Median Signals for each Column

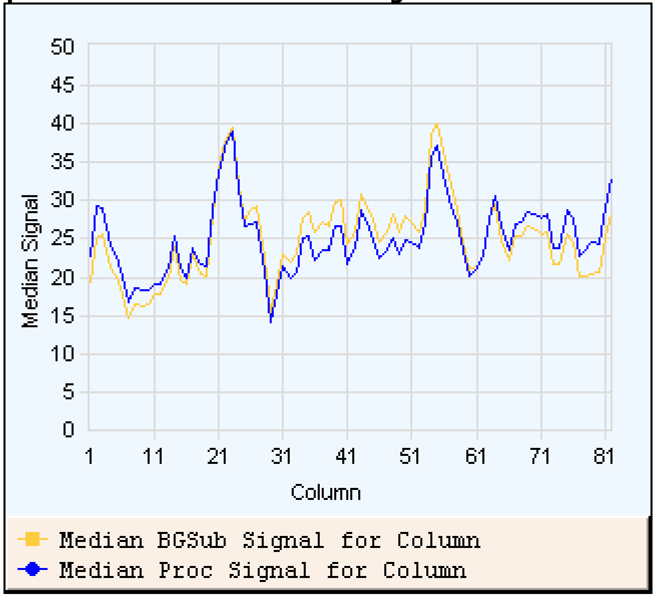

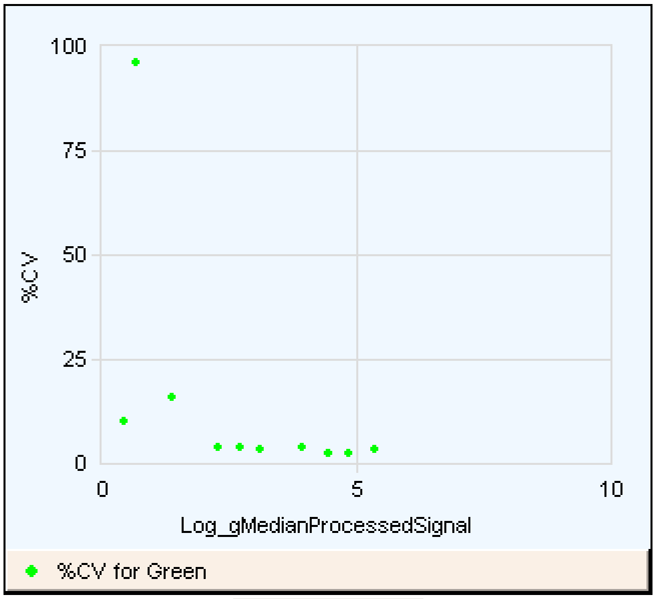

Median %CV:3.56

Agilent SpikeIns: Log(Signal) vs. Log(Relative concentration) Plot

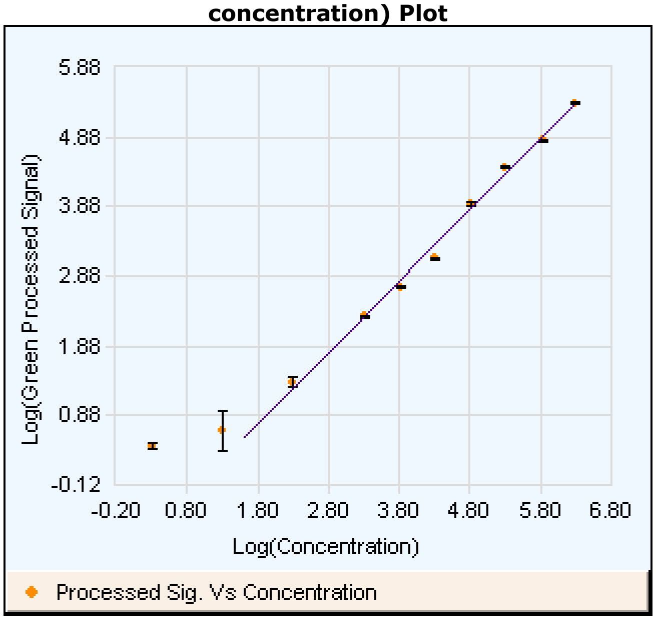

Agilent Spike-In Concentration-Response Statistics

Linear Range Statistics:

Low Signal	0.56
High Signal	5.66
Low Relative Concentration	1.61
High Relative Concentration	6.60
Slope	1.02
R^2 Value	0.99

Signal Detection Limit Statistics

Saturation Point	5.77
Low Threshold	0.30
Low Threshold Error	0.29
Spike-In Detection Limit	1.13

QC Report - Agilent Technologies : 1 Color Gene Expression

Date	Tuesday, September 20, 2011 - 11:05	Grid	035923_D_F_20110809
Image	UniversityPadova_253592310003_S01_H [2_4]	BG Method	No Background
Protocol	GE1-v5_95_Feb07 (Read Only)	Background Detrend	On(FeatNCRange, LoPass)
User Name	Administrator	Multiplicative Detrend	True
FE Version	9.5.1.1	Additive Error	3(Green)
		Saturation Value	588550 (g)

Spot Finding of the Four Corners of the Array

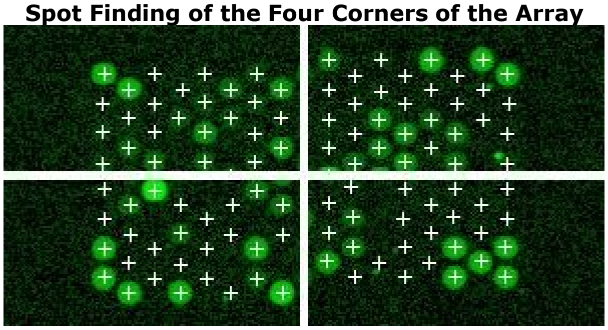

Grid Normal

	Feature	Local Background
	Green	Green
Non Uniform	4	40
Population	14	203

Spatial Distribution of All Outliers on the Array

192 rows x 82 columns

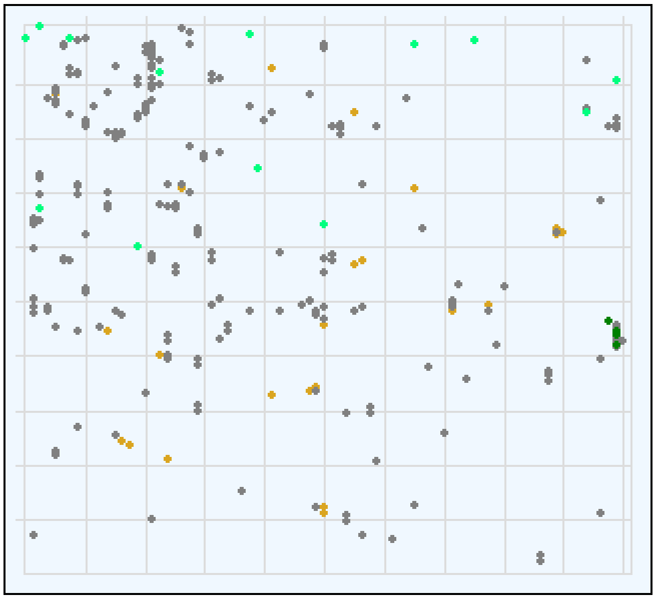

FeatureNonUnif (Green) = 4(0.03%)

GeneNonUnif (Green) = 3 (0.022 %)

BG NonUniform
BG Population
Green FeaturePopulation
Green Feature NonUniform

Negative Control Stats

	Green
Average Net Signals	15.26

Net Signal Statistics

Agilent SpikeIns:

	Green
# Saturated Features	0
99% of Sig. Distrib.	264431
50% of Sig. Distrib.	697
1% of Sig. Distrib.	14

Non-Control probes:

	Green
# Saturated Features	0
99% of Sig. Distrib.	30591
50% of Sig. Distrib.	27
1% of Sig. Distrib.	12

Histogram of Signals Plot

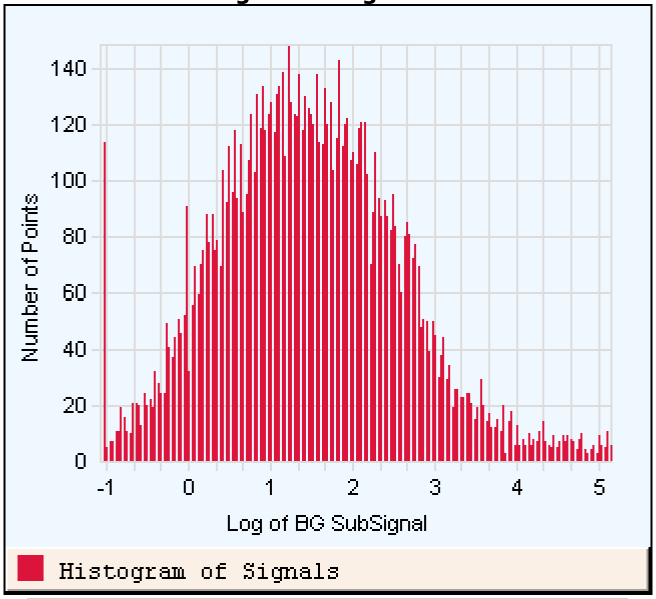

Features (NonCtrl) with BGSubSignal < 0: 3136 (Green)

StdDev Net Signals	2.63
Average BG Sub Signal	-1.73
StdDev BG Sub Signal	1.71

Local Bkg (inliers)

	Green
Number	13981
Avg	41.75
SD	2.00

Foreground Surface Fit

	Green
RMS_Fit	2.04
RMS_Resid	2.69
Avg_Fit	46.45

Multiplicative Surface Fit

	Green
RMS_Fit	0.10

Reproducibility: %CV for Replicated Probes

	Median %CV Signal (inliers)	
	Non-Control probes	Agilent SpikeIns
	Green	Green
BGSubSignal	-1.00	11.05
ProcessedSignal	-1.00	3.64

Agilent SpikeIns Signal Statistics

Probe Name	Log (Relative Conc.)	Median (Log Proc. Sig.)	% CV	StdDev
(+)E1A_r60_3	0.30	0.44	8.18	0.04
(+)E1A_r60_a104	1.30	0.71	55.20	0.17
(+)E1A_r60_a107	2.30	1.37	30.19	0.11
(+)E1A_r60_a135	3.30	2.30	4.94	0.02
(+)E1A_r60_a20	3.83	2.75	3.75	0.02
(+)E1A_r60_a22	4.30	3.20	2.54	0.01
(+)E1A_r60_a97	4.82	3.93	3.66	0.02
(+)E1A_r60_n11	5.30	4.48	3.12	0.01
(+)E1A_r60_n9	5.82	4.85	3.64	0.02
(+)E1A_r60_1	6.30	5.38	3.24	0.01

Agilent SpikeIns: %CV of Avg. Processed Signal Plot

Spatial Distribution of Median Signals for each Row

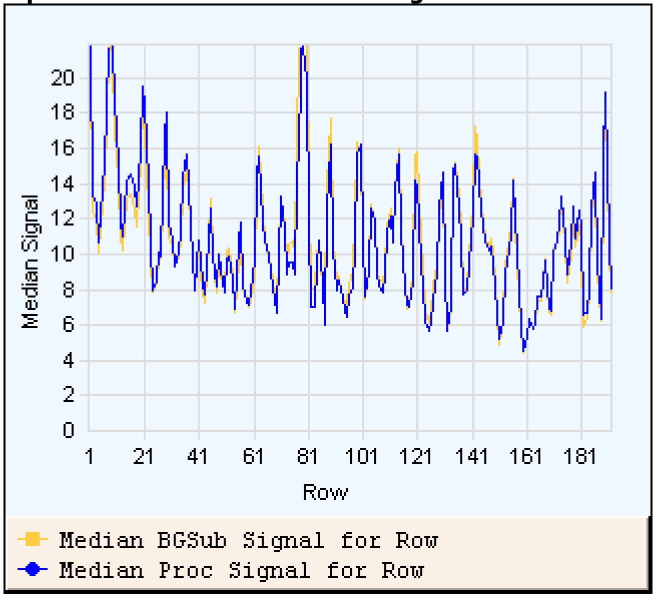

Spatial Distribution of Median Signals for each Column

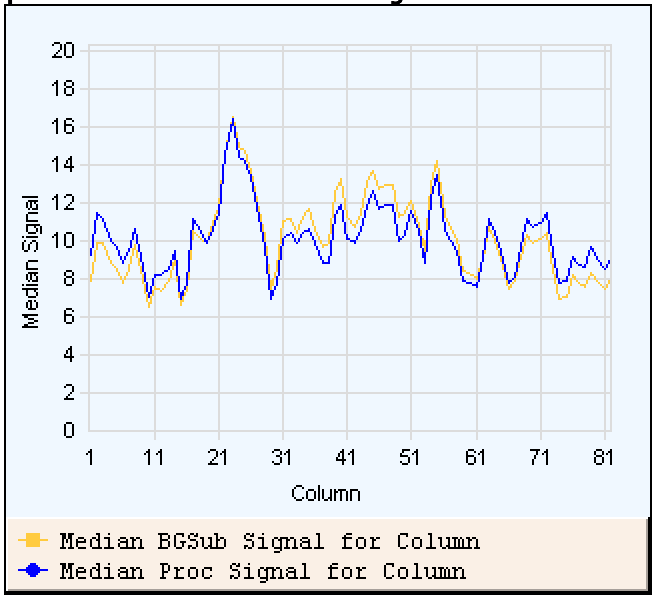

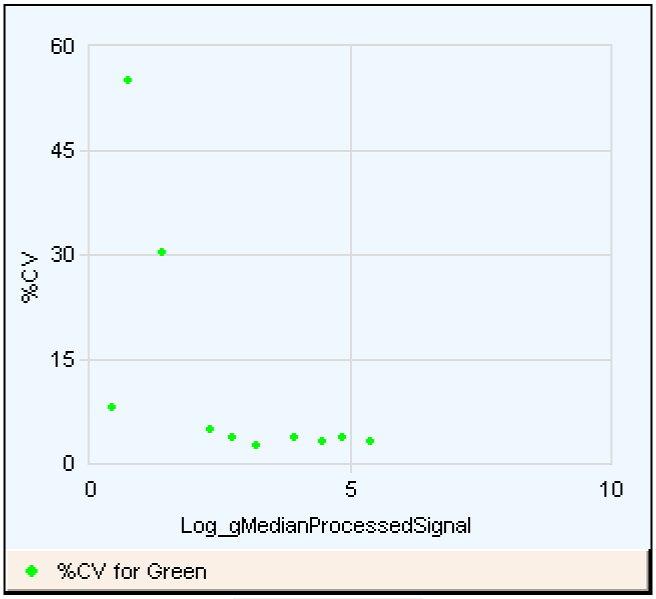

Median %CV:3.64

Agilent SpikeIns: Log(Signal) vs. Log(Relative concentration) Plot

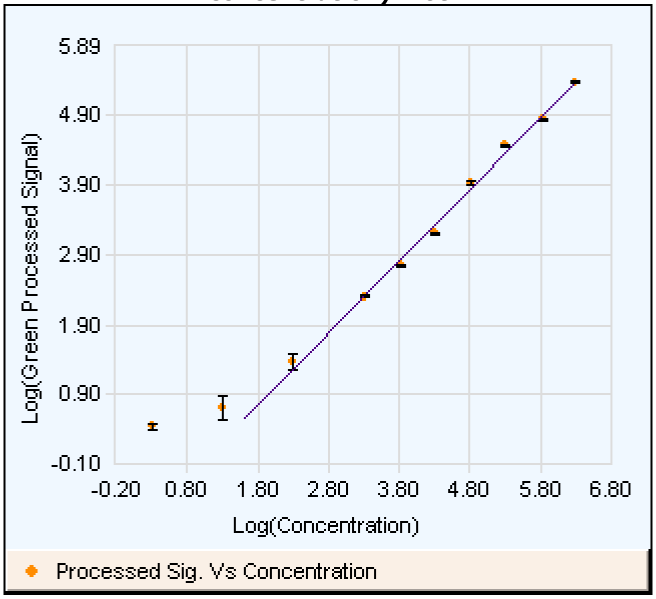

Agilent Spike-In Concentration-Response Statistics

Linear Range Statistics:

Low Signal	0.57
High Signal	5.65
Low Relative Concentration	1.60
High Relative Concentration	6.57
Slope	1.02
R^2 Value	1.00

Signal Detection Limit Statistics

Saturation Point	5.77
Low Threshold	0.32
Low Threshold Error	0.17
Spike-In Detection Limit	0.96

QC Report - Agilent Technologies : 1 Color Gene Expression

Date	Friday, September 02, 2011 - 10:55	Grid	035923_D_F_20110809
Image	UniversityPadova_253592310001_S01_H [1_3]	BG Method	No Background
Protocol	GE1-v5_95_Feb07 (Read Only)	Background Detrend	On(FeatNCRange, LoPass)
User Name	Administrator	Multiplicative Detrend	True
FE Version	9.5.1.1	Additive Error	2(Green)
		Saturation Value	597103 (g)

Spot Finding of the Four Corners of the Array

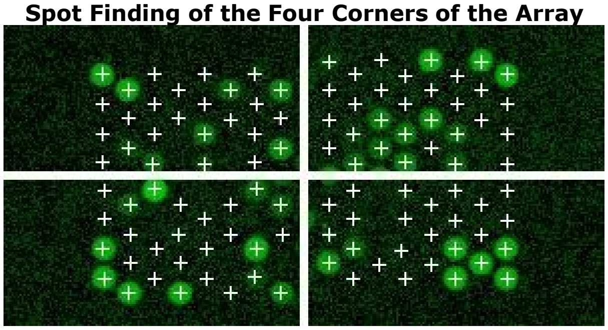

Grid Normal

	Feature	Local Background
	Green	Green
Non Uniform	0	32
Population	10	177

Spatial Distribution of All Outliers on the Array

192 rows x 82 columns

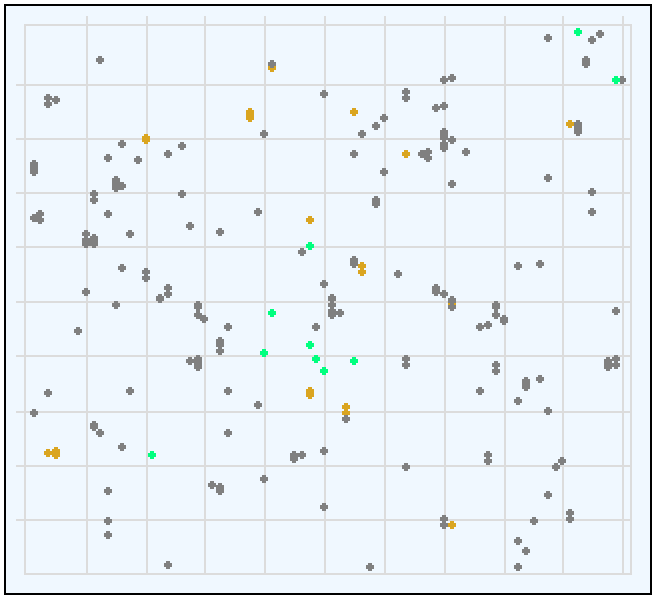

FeatureNonUnif (Green) = 0(0.00%)

GeneNonUnif (Green) = 0 (0.000 %)

BG NonUniform, BG Population, Green FeaturePopulation, Green Feature NonUniform

Negative Control Stats

	Green
Average Net Signals	11.19

Net Signal Statistics

Agilent SpikeIns:

	Green
# Saturated Features	0
99% of Sig. Distrib.	229776
50% of Sig. Distrib.	668
1% of Sig. Distrib.	10

Non-Control probes:

	Green
# Saturated Features	0
99% of Sig. Distrib.	24415
50% of Sig. Distrib.	18
1% of Sig. Distrib.	9

Histogram of Signals Plot

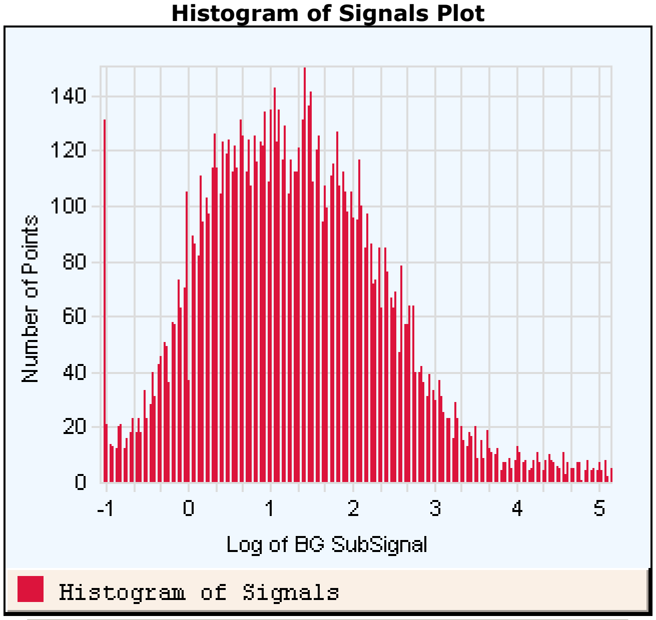

Features (NonCtrl) with BGSubSignal < 0: 3171 (Green)

StdDev Net Signals	1.50
Average BG Sub Signal	-1.38
StdDev BG Sub Signal	1.52

Local Bkg (inliers)

	Green
Number	14009
Avg	42.41
SD	1.86

Foreground Surface Fit

	Green
RMS_Fit	0.53
RMS_Resid	1.59
Avg_Fit	41.19

Multiplicative Surface Fit

	Green
RMS_Fit	0.11

Reproducibility: %CV for Replicated Probes

	Median %CV Signal (inliers)	
	Non-Control probes	Agilent SpikeIns
	Green	Green
BGSubSignal	-1.00	12.28
ProcessedSignal	-1.00	4.38

Agilent SpikeIns Signal Statistics

Probe Name	Log (Relative Conc.)	Median (Log Proc. Sig.)	% CV	StdDev
(+)E1A_r60_3	0.30	0.24	27.34	0.10
(+)E1A_r60_a104	1.30	0.62	30.97	0.15
(+)E1A_r60_a107	2.30	1.31	15.29	0.07
(+)E1A_r60_a135	3.30	2.23	4.52	0.02
(+)E1A_r60_a20	3.83	2.69	5.00	0.02
(+)E1A_r60_a22	4.30	3.13	3.98	0.02
(+)E1A_r60_a97	4.82	3.83	4.02	0.02
(+)E1A_r60_n11	5.30	4.39	3.96	0.02
(+)E1A_r60_n9	5.82	4.76	4.38	0.02
(+)E1A_r60_1	6.30	5.32	5.47	0.02

Agilent SpikeIns: %CV of Avg. Processed Signal Plot

Spatial Distribution of Median Signals for each Row

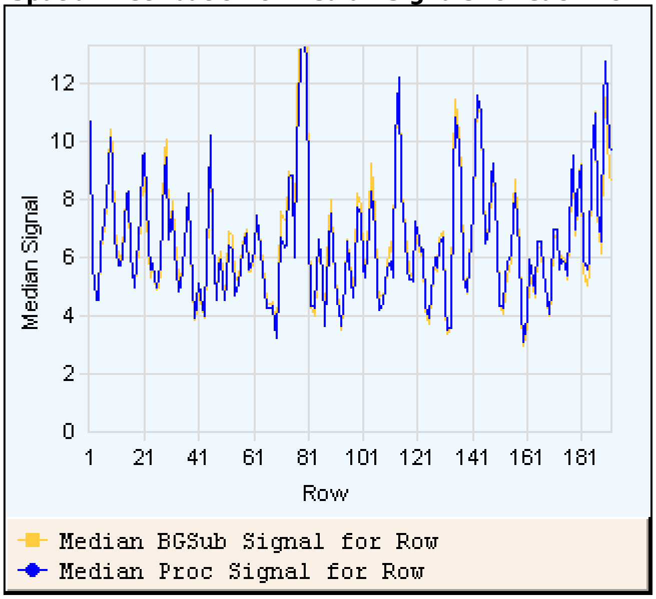

Spatial Distribution of Median Signals for each Column

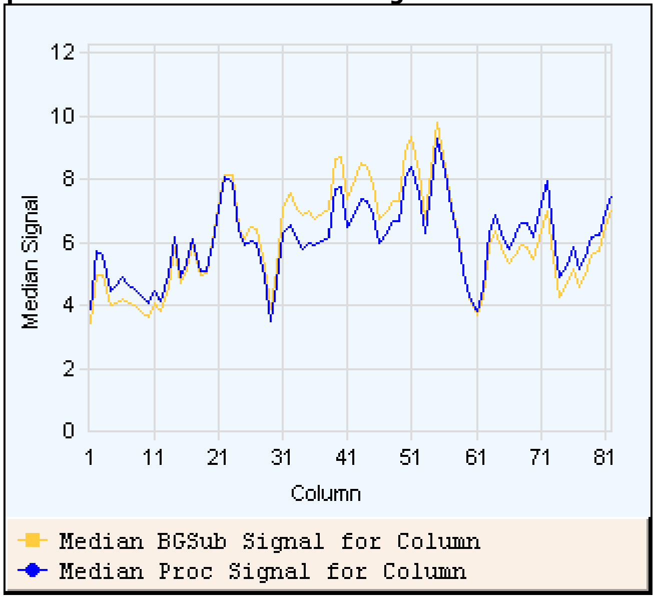

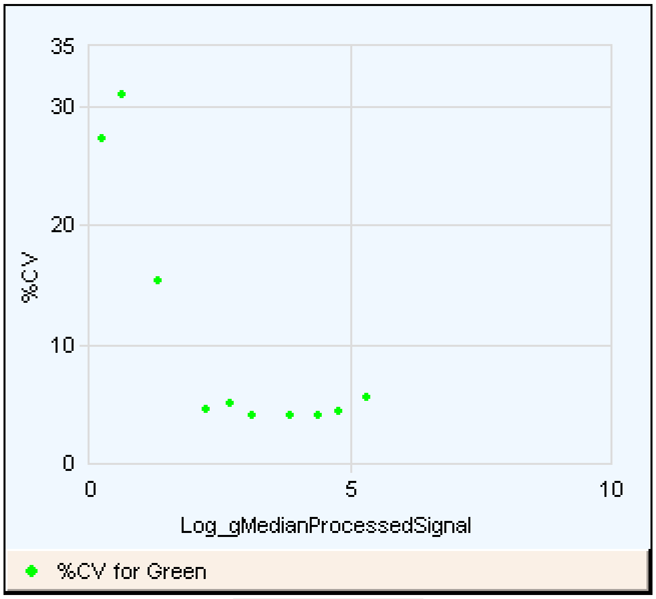

Median %CV:4.38

Agilent SpikeIns: Log(Signal) vs. Log(Relative concentration) Plot

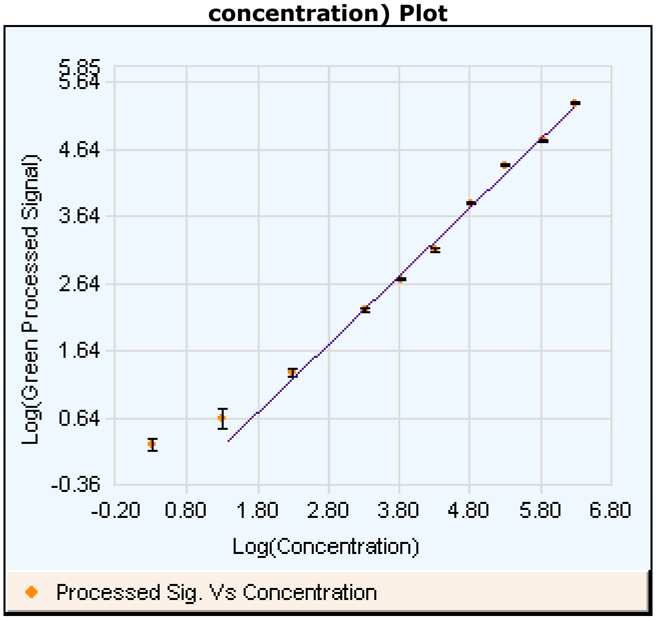

Agilent Spike-In Concentration-Response Statistics

Linear Range Statistics:

Low Signal	0.30
High Signal	5.70
Low Relative Concentration	1.40
High Relative Concentration	6.70
Slope	1.02
R^2 Value	1.00

Signal Detection Limit Statistics

Saturation Point	5.78
Low Threshold	0.09
Low Threshold Error	0.18
Spike-In Detection Limit	0.76

QC Report - Agilent Technologies : 1 Color Gene Expression

Date	Tuesday, September 20, 2011 - 11:09	Grid	035923_D_F_20110809
Image	UniversityPadova_253592310004_S01_H [1_1]	BG Method	No Background
Protocol	GE1-v5_95_Feb07 (Read Only)	Background Detrend	On(FeatNCRange, LoPass)
User Name	Administrator	Multiplicative Detrend	True
FE Version	9.5.1.1	Additive Error	2(Green)
		Saturation Value	585688 (g)

Spot Finding of the Four Corners of the Array

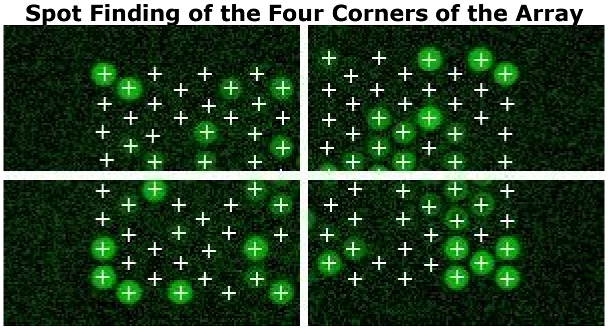

Grid Normal

	Feature Green	Local Background Green
Non Uniform	0	8
Population	17	228

Spatial Distribution of All Outliers on the Array

192 rows x 82 columns

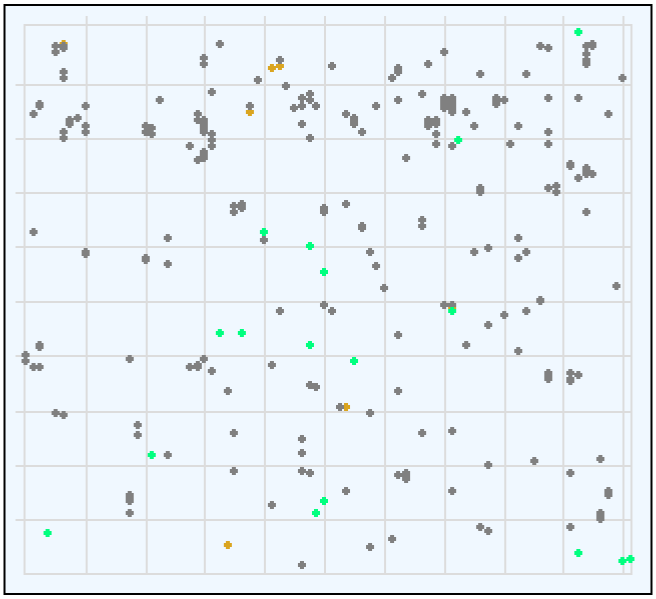

FeatureNonUnif (Green) = 0(0.00%)

GeneNonUnif (Green) = 0 (0.000 %)

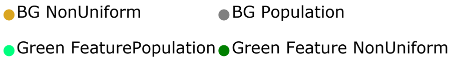

Negative Control Stats

	Green
Average Net Signals	16.32

Net Signal Statistics

Agilent SpikeIns:

	Green
# Saturated Features	0
99% of Sig. Distrib.	223860
50% of Sig. Distrib.	593
1% of Sig. Distrib.	16

Non-Control probes:

	Green
# Saturated Features	0
99% of Sig. Distrib.	22660
50% of Sig. Distrib.	25
1% of Sig. Distrib.	14

Histogram of Signals Plot

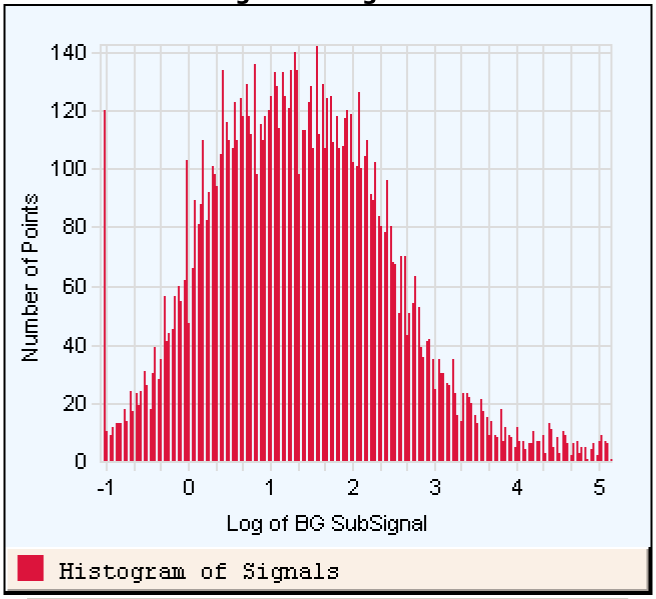

Features (NonCtrl) with BGSubSignal < 0: 3147 (Green)

StdDev Net Signals	1.65
Average BG Sub Signal	-1.63
StdDev BG Sub Signal	1.63

Local Bkg (inliers)

	Green
Number	13972
Avg	44.86
SD	1.96

Foreground Surface Fit

	Green
RMS_Fit	0.69
RMS_Resid	1.87
Avg_Fit	47.29

Multiplicative Surface Fit

	Green
RMS_Fit	0.12

Reproducibility: %CV for Replicated Probes

	Median %CV Signal (inliers)	
	Non-Control probes	Agilent SpikeIns
	Green	Green
BGSubSignal	-1.00	13.57
ProcessedSignal	-1.00	3.97

Agilent SpikeIns Signal Statistics

Probe Name	Log (Relative Conc.)	Median (Log Proc. Sig.)	% CV	StdDev
(+)E1A_r60_3	0.30	0.31	62.67	0.18
(+)E1A_r60_a104	1.30	0.41	36.06	0.15
(+)E1A_r60_a107	2.30	1.33	20.31	0.08
(+)E1A_r60_a135	3.30	2.25	6.66	0.03
(+)E1A_r60_a20	3.83	2.66	3.64	0.02
(+)E1A_r60_a22	4.30	3.14	3.03	0.01
(+)E1A_r60_a97	4.82	3.88	3.78	0.02
(+)E1A_r60_n11	5.30	4.43	3.97	0.02
(+)E1A_r60_n9	5.82	4.77	5.65	0.03
(+)E1A_r60_1	6.30	5.32	5.66	0.02

Agilent SpikeIns: %CV of Avg. Processed Signal Plot

Spatial Distribution of Median Signals for each Row

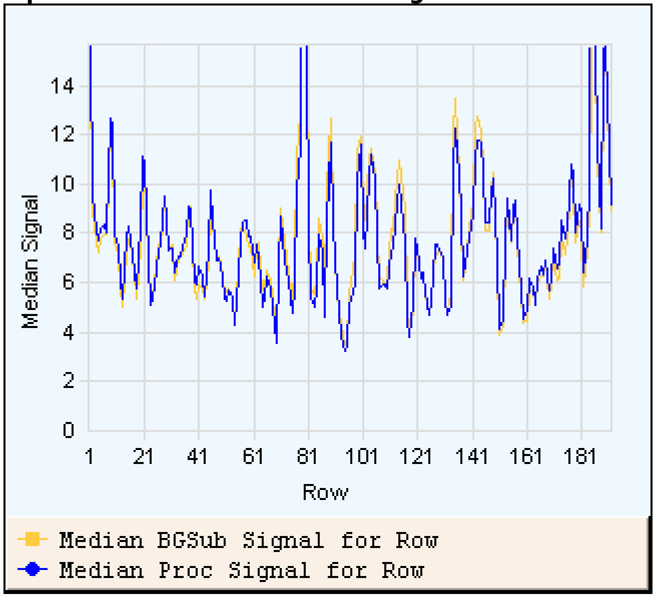

Spatial Distribution of Median Signals for each Column

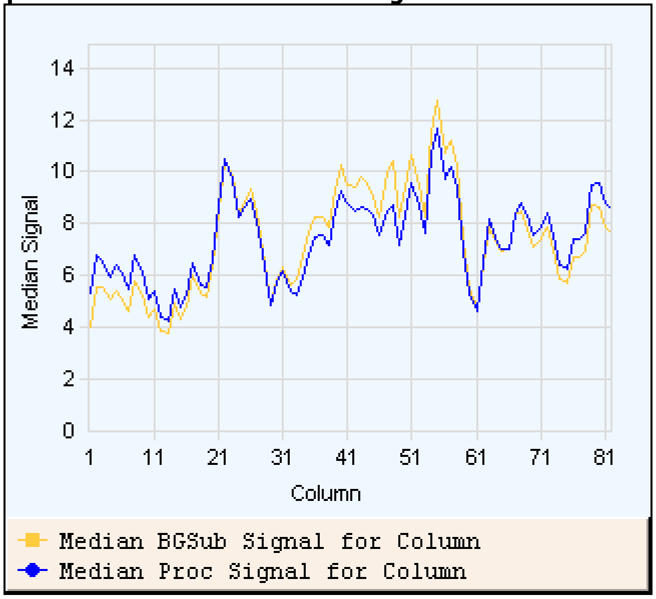

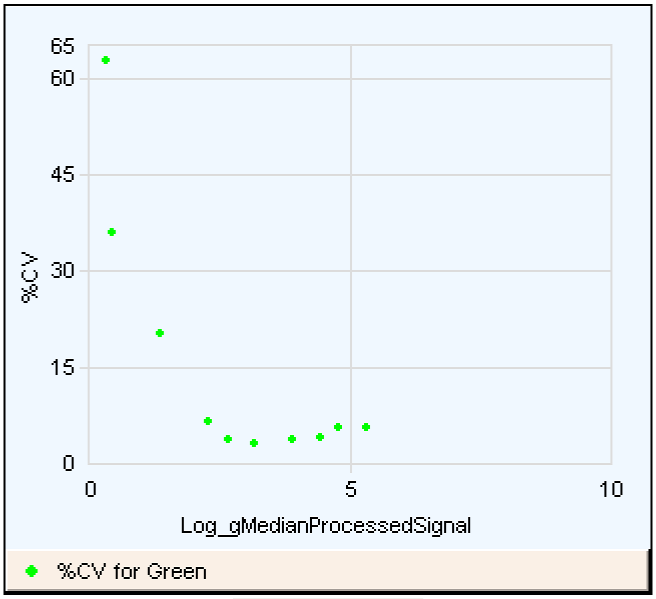

Median %CV:3.97

Agilent SpikeIns: Log(Signal) vs. Log(Relative concentration) Plot

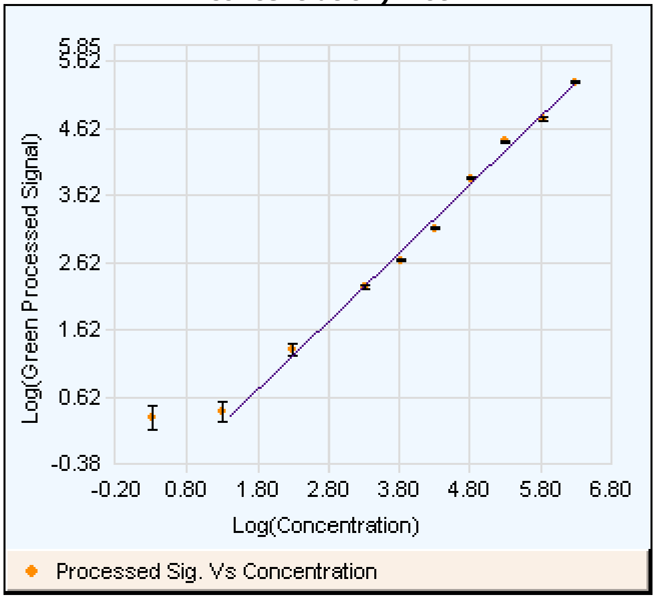

Agilent Spike-In Concentration-Response Statistics

Linear Range Statistics:

Low Signal	0.34
High Signal	5.65
Low Relative Concentration	1.42
High Relative Concentration	6.64
Slope	1.02
R^2 Value	0.99

Signal Detection Limit Statistics

Saturation Point	5.77
Low Threshold	0.06
Low Threshold Error	0.24
Spike-In Detection Limit	0.61

QC Report - Agilent Technologies : 1 Color Gene Expression

Date	Tuesday, September 20, 2011 - 11:09	Grid	035923_D_F_20110809
Image	UniversityPadova_253592310004_S01_H [1_2]	BG Method	No Background
Protocol	GE1-v5_95_Feb07 (Read Only)	Background Detrend	On(FeatNCRange, LoPass)
User Name	Administrator	Multiplicative Detrend	True
FE Version	9.5.1.1	Additive Error	2(Green)
		Saturation Value	582976 (g)

Spot Finding of the Four Corners of the Array

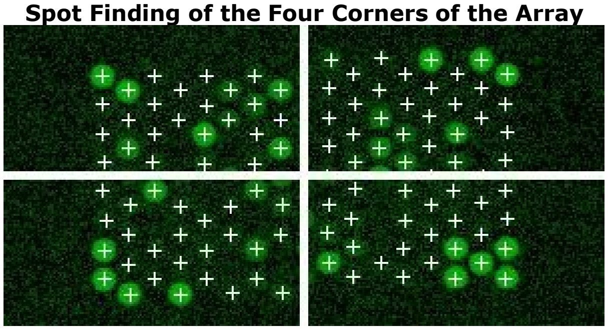

Grid Normal

	Feature	Local Background
	Green	Green
Non Uniform	0	13
Population	9	173

Spatial Distribution of All Outliers on the Array

192 rows x 82 columns

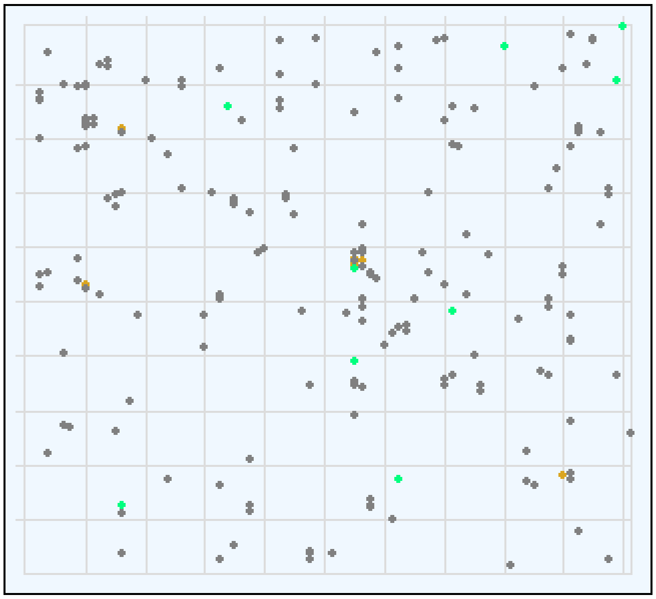

FeatureNonUnif (Green) = 0(0.00%)

GeneNonUnif (Green) = 0 (0.000 %)

BG NonUniform   BG Population

Green FeaturePopulation   Green Feature NonUniform

Negative Control Stats

	Green
Average Net Signals	19.81

Net Signal Statistics

Agilent SpikeIns:

	Green
# Saturated Features	0
99% of Sig. Distrib.	176080
50% of Sig. Distrib.	432
1% of Sig. Distrib.	19

Non-Control probes:

	Green
# Saturated Features	0
99% of Sig. Distrib.	9118
50% of Sig. Distrib.	26
1% of Sig. Distrib.	17

Histogram of Signals Plot

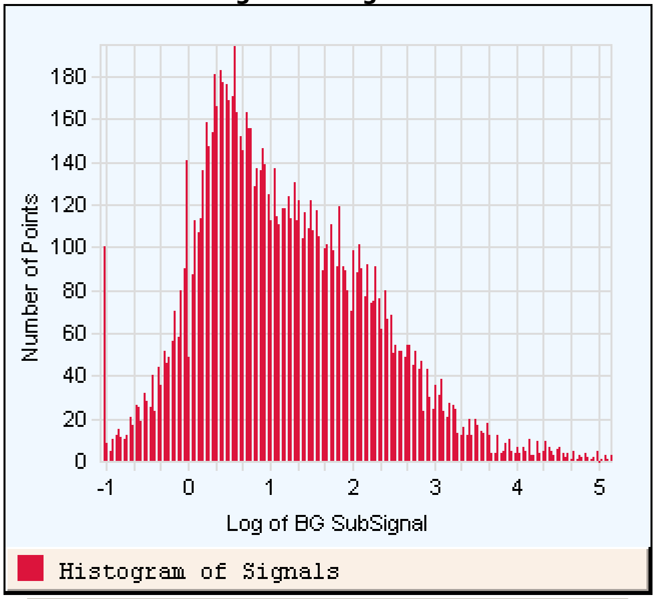

Features (NonCtrl) with BGSubSignal < 0: 2724 (Green)

StdDev Net Signals	1.83
Average BG Sub Signal	-1.68
StdDev BG Sub Signal	1.49

Local Bkg (inliers)

	Green
Number	14027
Avg	44.92
SD	1.79

Foreground Surface Fit

	Green
RMS_Fit	1.15
RMS_Resid	1.57
Avg_Fit	50.82

Multiplicative Surface Fit

	Green
RMS_Fit	0.11

Reproducibility: %CV for Replicated Probes

	Median %CV Signal (inliers)	
	Non-Control probes	Agilent SpikeIns
	Green	Green
BGSubSignal	-1.00	12.38
ProcessedSignal	-1.00	3.63

Agilent SpikeIns Signal Statistics

Probe Name	Log (Relative Conc.)	Median (Log Proc. Sig.)	% CV	StdDev
(+)E1A_r60_3	0.30	0.23	37.39	0.12
(+)E1A_r60_a104	1.30	0.61	48.53	0.22
(+)E1A_r60_a107	2.30	1.26	25.20	0.13
(+)E1A_r60_a135	3.30	2.11	5.28	0.02
(+)E1A_r60_a20	3.83	2.52	3.32	0.01
(+)E1A_r60_a22	4.30	2.97	3.48	0.02
(+)E1A_r60_a97	4.82	3.73	3.44	0.01
(+)E1A_r60_n11	5.30	4.27	4.50	0.02
(+)E1A_r60_n9	5.82	4.64	3.74	0.02
(+)E1A_r60_1	6.30	5.21	3.63	0.02

Agilent SpikeIns: %CV of Avg. Processed Signal Plot

Spatial Distribution of Median Signals for each Row

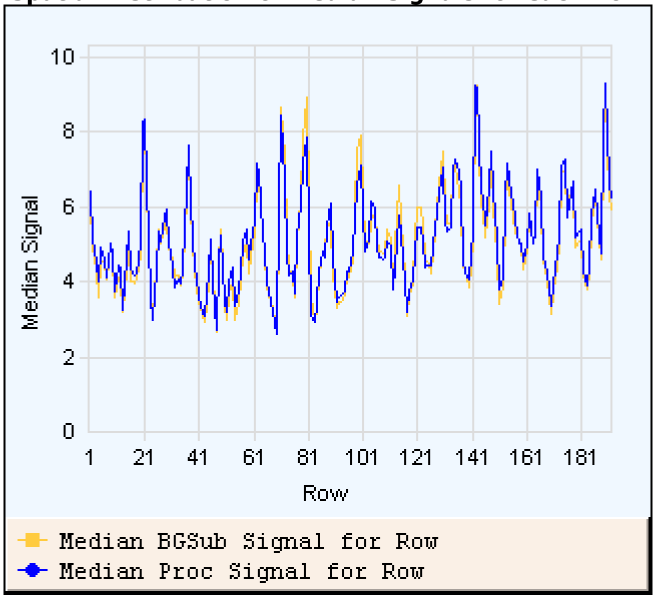

Spatial Distribution of Median Signals for each Column

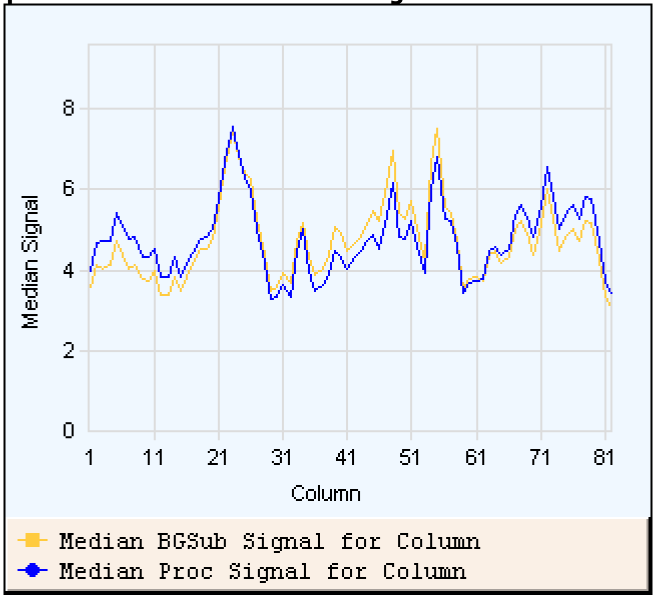

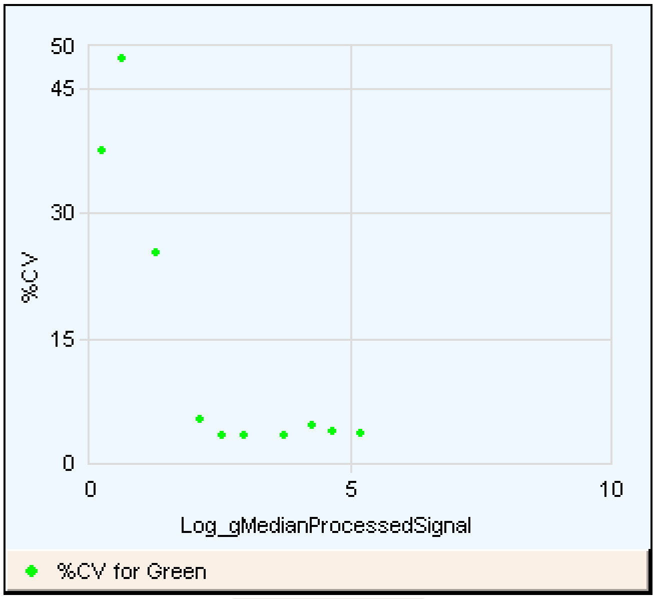

Median %CV:3.63

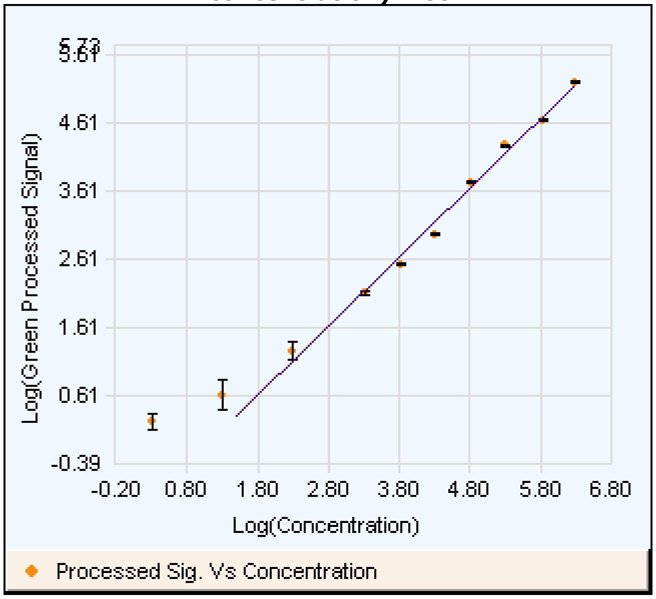

Agilent Spike-In Concentration-Response Statistics

Linear Range Statistics:

Low Signal	0.33
High Signal	5.71
Low Relative Concentration	1.52
High Relative Concentration	6.85
Slope	1.01
R^2 Value	0.99

Signal Detection Limit Statistics

Saturation Point	5.77
Low Threshold	0.12
Low Threshold Error	0.25
Spike-In Detection Limit	0.76

QC Report - Agilent Technologies : 1 Color Gene Expression

Date	Tuesday, September 20, 2011 - 11:09	Grid	035923_D_F_20110809
Image	UniversityPadova_253592310004_S01_H [1_3]	BG Method	No Background
Protocol	GE1-v5_95_Feb07 (Read Only)	Background Detrend	On(FeatNCRange, LoPass)
User Name	Administrator	Multiplicative Detrend	True
FE Version	9.5.1.1	Additive Error	2(Green)
		Saturation Value	584478 (g)

Spot Finding of the Four Corners of the Array

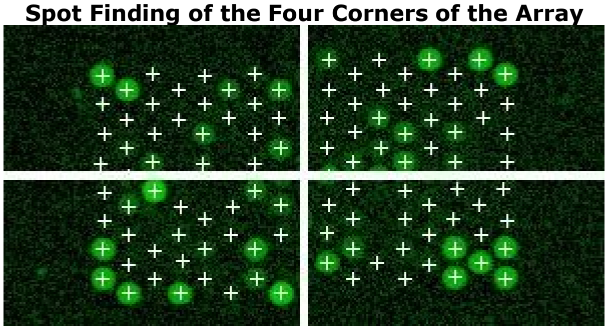

Grid Normal

	Feature	Local Background
	Green	Green
Non Uniform	1	13
Population	13	213

Spatial Distribution of All Outliers on the Array

192 rows x 82 columns

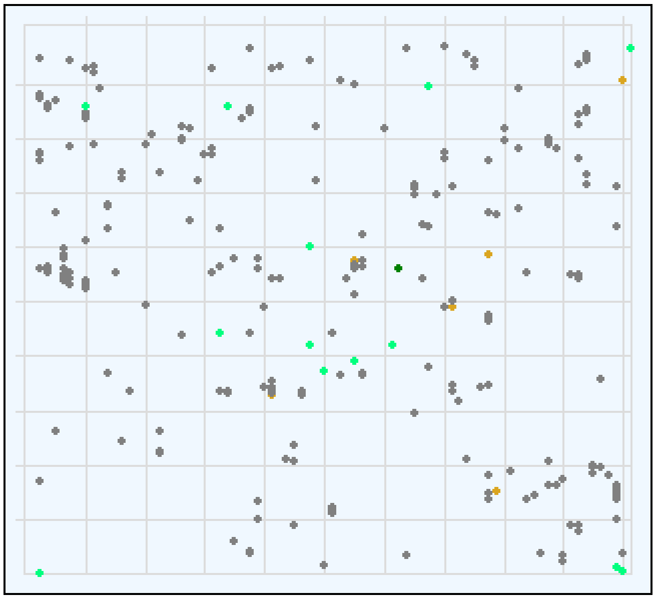

FeatureNonUnif (Green) = 1(0.01%)

GeneNonUnif (Green) = 1 (0.007 %)

BG NonUniform BG Population

Green FeaturePopulation Green Feature NonUniform

Negative Control Stats

	Green
Average Net Signals	16.38

Net Signal Statistics

Agilent SpikeIns:

	Green
# Saturated Features	0
99% of Sig. Distrib.	219556
50% of Sig. Distrib.	619
1% of Sig. Distrib.	14

Non-Control probes:

	Green
# Saturated Features	0
99% of Sig. Distrib.	18859
50% of Sig. Distrib.	22
1% of Sig. Distrib.	14

Histogram of Signals Plot

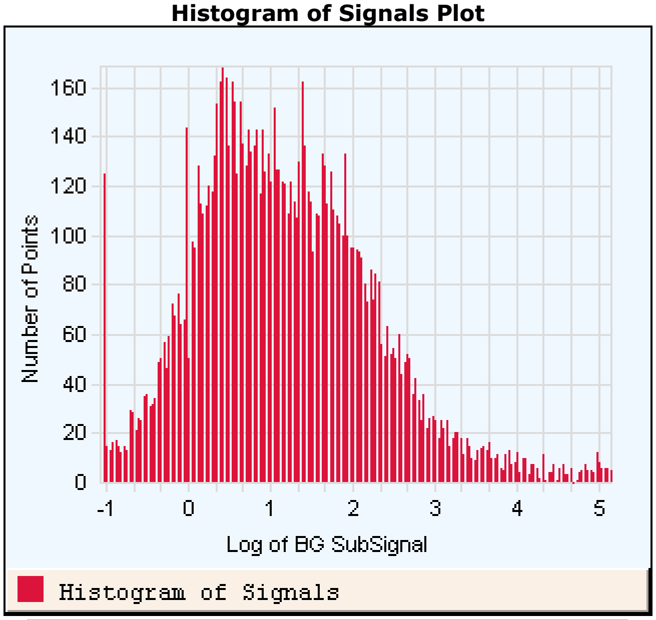

Features (NonCtrl) with BGSubSignal < 0: 2828 (Green)

StdDev Net Signals	1.65
Average BG Sub Signal	-0.95
StdDev BG Sub Signal	1.61

Local Bkg (inliers)

	Green
Number	13987
Avg	44.67
SD	1.90

Foreground Surface Fit

	Green
RMS_Fit	0.93
RMS_Resid	1.73
Avg_Fit	46.68

Multiplicative Surface Fit

	Green
RMS_Fit	0.10

Reproducibility: %CV for Replicated Probes

	Median %CV Signal (inliers)	
	Non-Control probes	Agilent SpikeIns
	Green	Green
BGSubSignal	-1.00	11.75
ProcessedSignal	-1.00	4.03

Agilent SpikeIns Signal Statistics

Probe Name	Log (Relative Conc.)	Median (Log Proc. Sig.)	% CV	StdDev
(+)E1A_r60_3	0.30	0.27	21.48	0.08
(+)E1A_r60_a104	1.30	0.57	46.28	0.20
(+)E1A_r60_a107	2.30	1.35	15.28	0.07
(+)E1A_r60_a135	3.30	2.24	5.08	0.02
(+)E1A_r60_a20	3.83	2.66	4.06	0.02
(+)E1A_r60_a22	4.30	3.10	3.33	0.01
(+)E1A_r60_a97	4.82	3.85	4.31	0.02
(+)E1A_r60_n11	5.30	4.39	3.19	0.01
(+)E1A_r60_n9	5.82	4.77	3.75	0.02
(+)E1A_r60_1	6.30	5.31	4.03	0.02

Agilent SpikeIns: %CV of Avg. Processed Signal Plot

Spatial Distribution of Median Signals for each Row

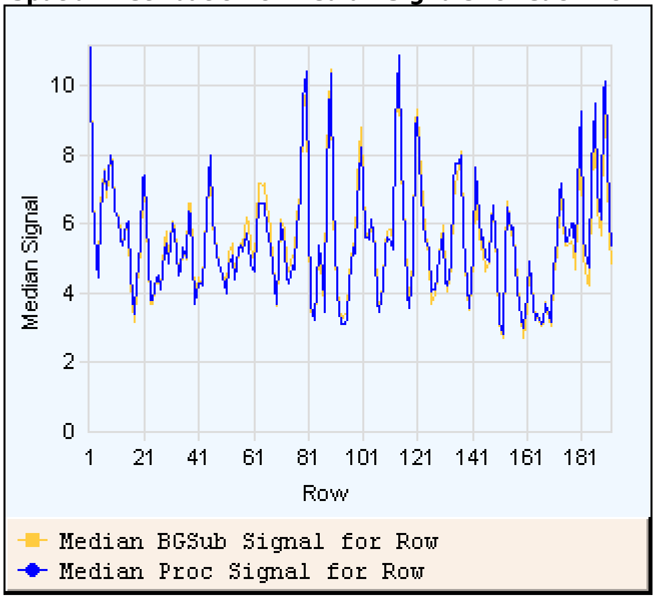

Spatial Distribution of Median Signals for each Column

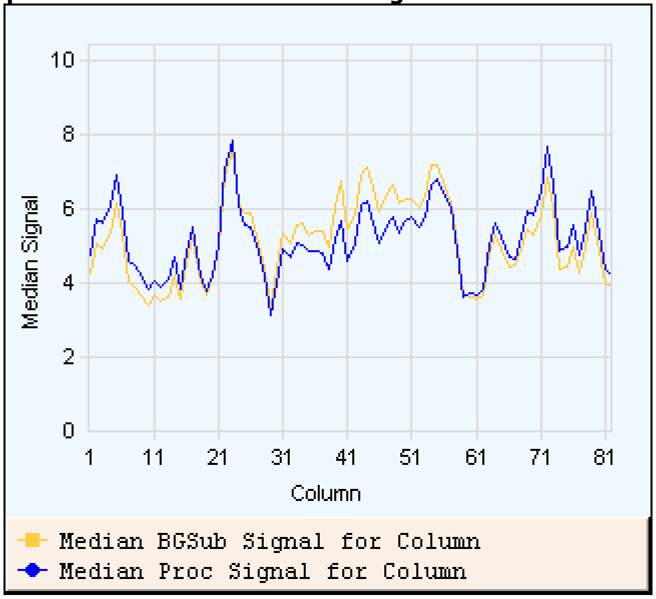

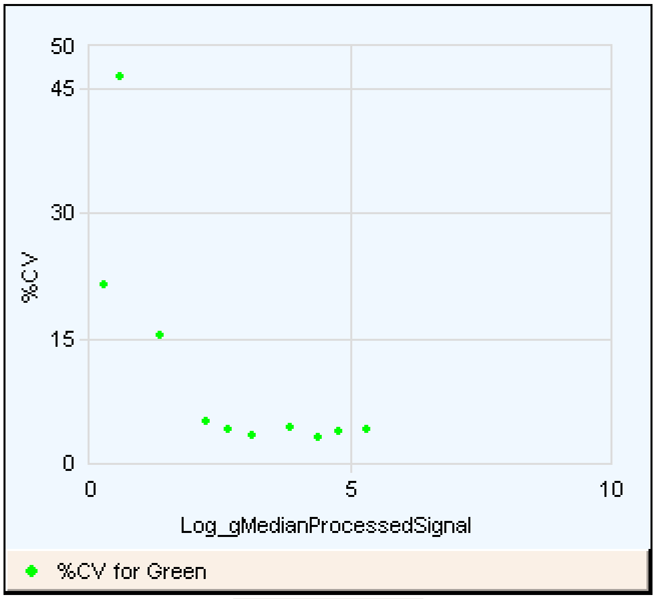

Median %CV:4.03

Agilent SpikeIns: Log(Signal) vs. Log(Relative concentration) Plot

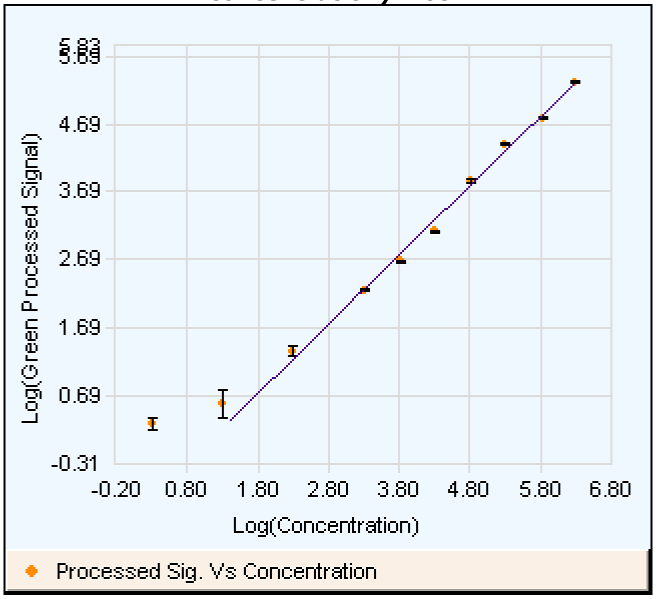

Agilent Spike-In Concentration-Response Statistics

Linear Range Statistics:

Low Signal	0.35
High Signal	5.68
Low Relative Concentration	1.42
High Relative Concentration	6.69
Slope	1.01
R^2 Value	0.99

Signal Detection Limit Statistics

Saturation Point	5.77
Low Threshold	0.11
Low Threshold Error	0.22
Spike-In Detection Limit	0.85

QC Report - Agilent Technologies : 1 Color Gene Expression

Date	Friday, September 23, 2011 - 11:02	Grid	035923_D_F_20110809
Image	UniversityPadova_253592310006_S01_H [1_1]	BG Method	No Background
Protocol	GE1-v5_95_Feb07 (Read Only)	Background Detrend	On(FeatNCRange, LoPass)
User Name	Administrator	Multiplicative Detrend	True
FE Version	9.5.1.1	Additive Error	2(Green)
		Saturation Value	587843 (g)

Spot Finding of the Four Corners of the Array

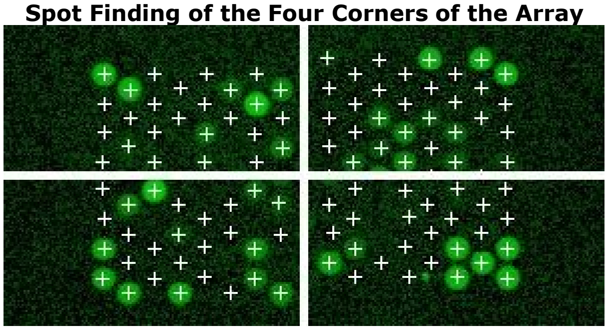

Grid Normal

	Feature	Local Background
	Green	Green
Non Uniform	7	38
Population	18	176

Spatial Distribution of All Outliers on the Array

192 rows x 82 columns

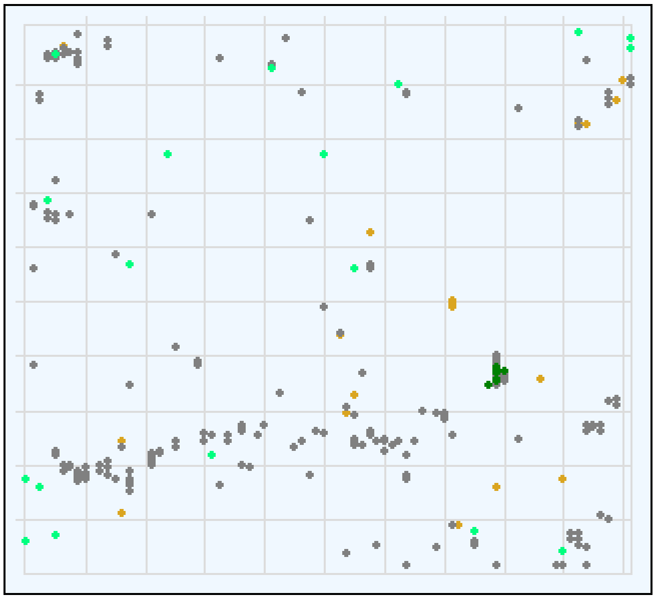

FeatureNonUnif (Green) = 7(0.05%)

GeneNonUnif (Green) = 7 (0.051 %)

BG NonUniform   BG Population

Green FeaturePopulation   Green Feature NonUniform

Negative Control Stats

	Green
Average Net Signals	12.60

Net Signal Statistics

Agilent SpikeIns:

	Green
# Saturated Features	0
99% of Sig. Distrib.	185184
50% of Sig. Distrib.	385
1% of Sig. Distrib.	11

Non-Control probes:

	Green
# Saturated Features	0
99% of Sig. Distrib.	22154
50% of Sig. Distrib.	18
1% of Sig. Distrib.	10

Histogram of Signals Plot

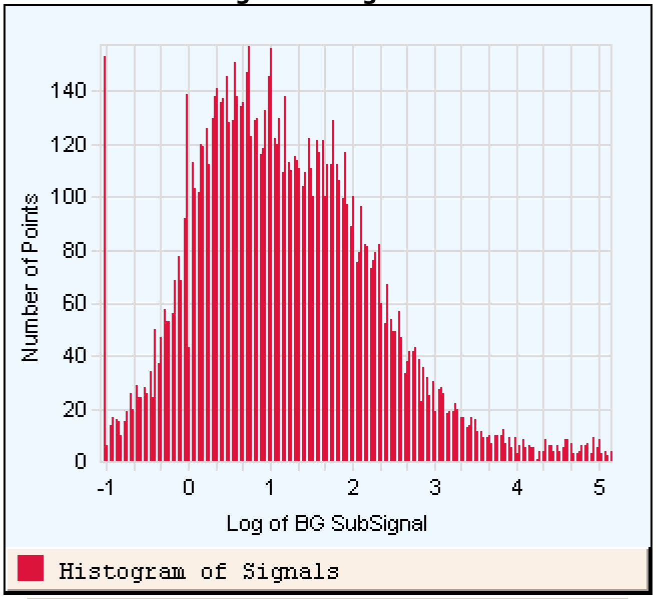

Features (NonCtrl) with BGSubSignal < 0: 3098 (Green)

StdDev Net Signals	1.95
Average BG Sub Signal	-1.03
StdDev BG Sub Signal	1.49

Local Bkg (inliers)

	Green
Number	14012
Avg	44.28
SD	2.52

Foreground Surface Fit

	Green
RMS_Fit	1.14
RMS_Resid	1.59
Avg_Fit	42.01

Multiplicative Surface Fit

	Green
RMS_Fit	0.06

Reproducibility: %CV for Replicated Probes

	Median %CV Signal (inliers)	
	Non-Control probes	Agilent SpikeIns
	Green	Green
BGSubSignal	-1.00	7.90
ProcessedSignal	-1.00	5.07

Agilent SpikeIns Signal Statistics

Probe Name	Log (Relative Conc.)	Median (Log Proc. Sig.)	% CV	StdDev
(+)E1A_r60_3	0.30	0.22	63.05	0.17
(+)E1A_r60_a104	1.30	0.55	52.18	0.22
(+)E1A_r60_a107	2.30	1.27	13.27	0.06
(+)E1A_r60_a135	3.30	2.17	5.84	0.03
(+)E1A_r60_a20	3.83	2.54	4.97	0.02
(+)E1A_r60_a22	4.30	3.11	4.47	0.02
(+)E1A_r60_a97	4.82	3.88	5.07	0.02
(+)E1A_r60_n11	5.30	4.38	3.68	0.02
(+)E1A_r60_n9	5.82	4.71	8.19	0.04
(+)E1A_r60_1	6.30	5.26	5.19	0.02

Agilent SpikeIns: %CV of Avg. Processed Signal Plot

Spatial Distribution of Median Signals for each Row

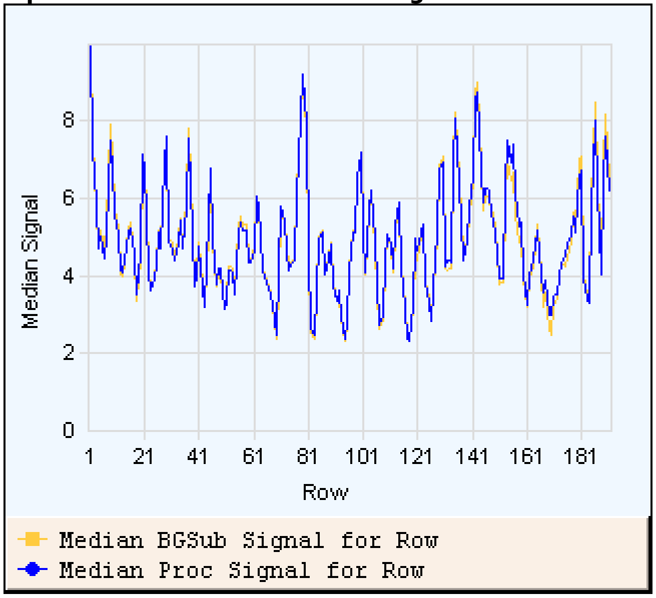

Spatial Distribution of Median Signals for each Column

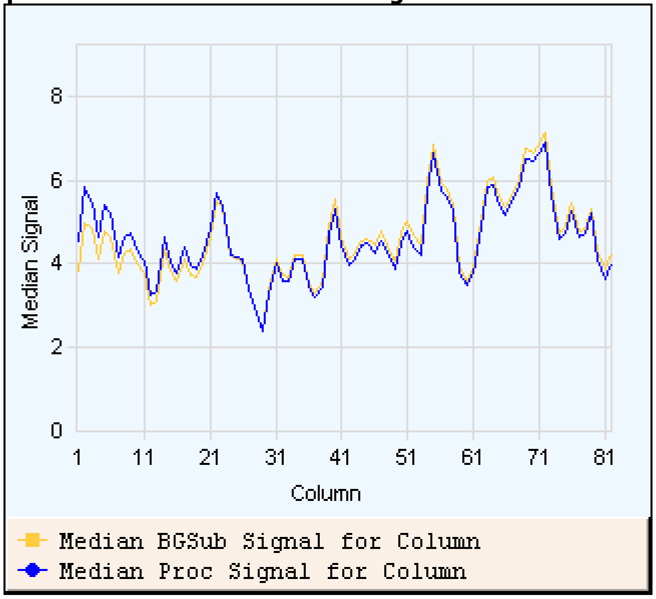

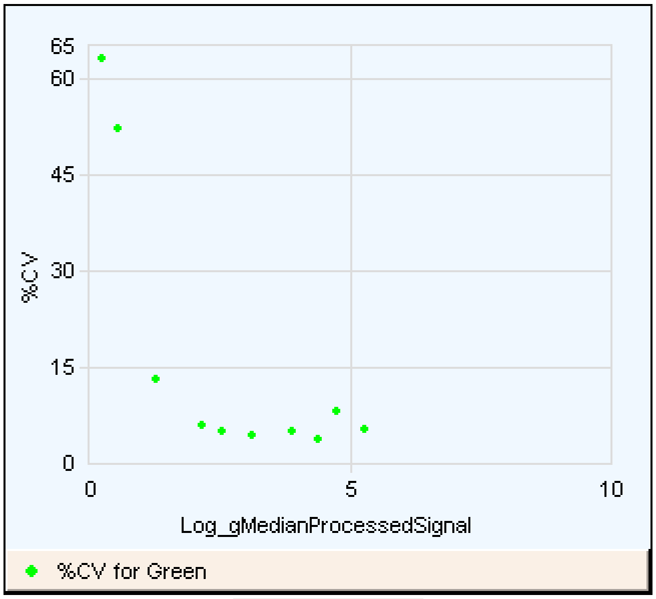

Median %CV:5.07

Agilent SpikeIns: Log(Signal) vs. Log(Relative concentration) Plot

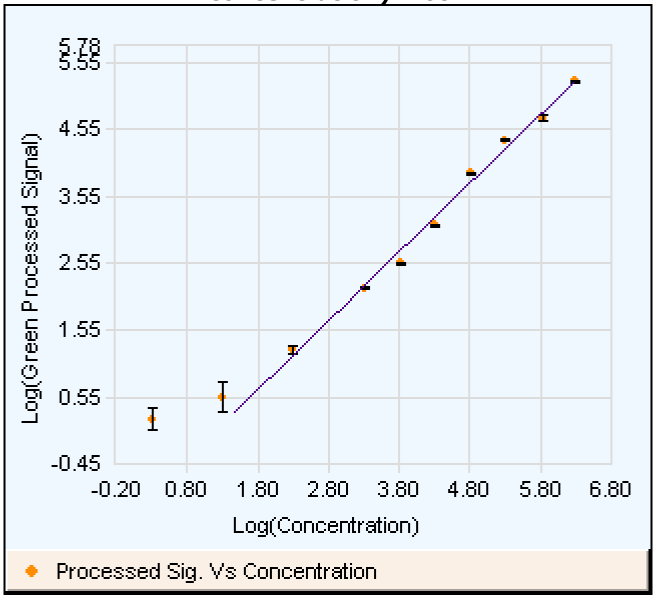

Agilent Spike-In Concentration-Response Statistics

Linear Range Statistics:

Low Signal	0.32
High Signal	5.67
Low Relative Concentration	1.48
High Relative Concentration	6.69
Slope	1.03
R^2 Value	0.99

Signal Detection Limit Statistics

Saturation Point	5.77
Low Threshold	0.07
Low Threshold Error	0.28
Spike-In Detection Limit	0.78

QC Report - Agilent Technologies : 1 Color Gene Expression

Date	Tuesday, September 20, 2011 - 11:10	Grid	035923_D_F_20110809
Image	UniversityPadova_253592310004_S01_H [1_4]	BG Method	No Background
Protocol	GE1-v5_95_Feb07 (Read Only)	Background Detrend	On(FeatNCRange, LoPass)
User Name	Administrator	Multiplicative Detrend	True
FE Version	9.5.1.1	Additive Error	2(Green)
		Saturation Value	583929 (g)

Spot Finding of the Four Corners of the Array

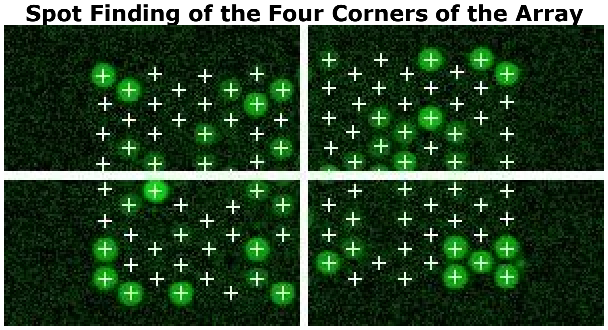

Grid Normal

	Feature	Local Background
	Green	Green
Non Uniform	19	34
Population	14	230

Spatial Distribution of All Outliers on the Array

192 rows x 82 columns

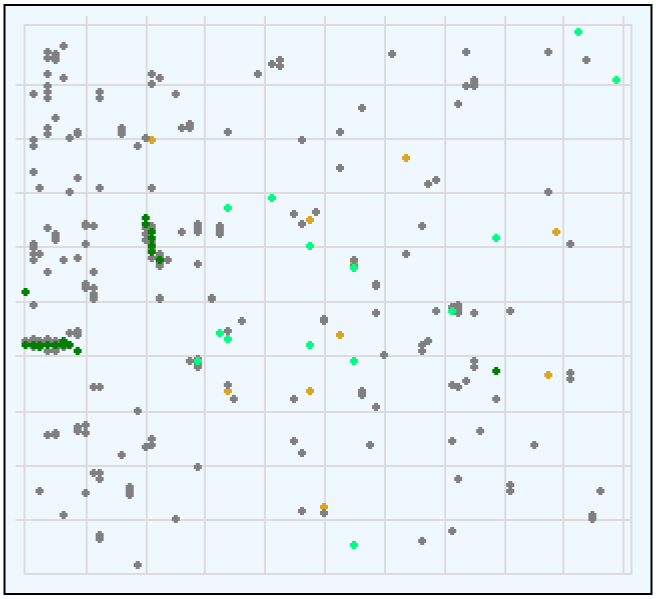

FeatureNonUnif (Green) = 19(0.13%)

GeneNonUnif (Green) = 19 (0.139 %)

BG NonUniform　BG Population

Green FeaturePopulation　Green Feature NonUniform

Negative Control Stats

	Green
Average Net Signals	16.40

Net Signal Statistics

Agilent SpikeIns:

	Green
# Saturated Features	0
99% of Sig. Distrib.	226256
50% of Sig. Distrib.	565
1% of Sig. Distrib.	16

Non-Control probes:

	Green
# Saturated Features	0
99% of Sig. Distrib.	27515
50% of Sig. Distrib.	25
1% of Sig. Distrib.	14

Histogram of Signals Plot

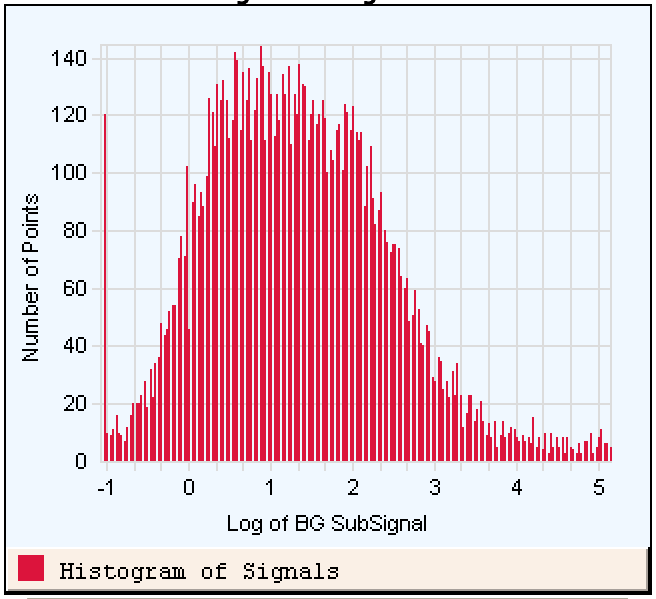

Features (NonCtrl) with BGSubSignal < 0: 2725 (Green)

StdDev Net Signals	1.84
Average BG Sub Signal	-1.06
StdDev BG Sub Signal	1.55

Local Bkg (inliers)

	Green
Number	13967
Avg	42.22
SD	1.92

Foreground Surface Fit

	Green
RMS_Fit	0.81
RMS_Resid	2.07
Avg_Fit	46.77

Multiplicative Surface Fit

	Green
RMS_Fit	0.10

Reproducibility: %CV for Replicated Probes

	Median %CV Signal (inliers)	
	Non-Control probes	Agilent SpikeIns
	Green	Green
BGSubSignal	-1.00	11.30
ProcessedSignal	-1.00	4.74

Agilent SpikeIns Signal Statistics

Probe Name	Log (Relative Conc.)	Median (Log Proc. Sig.)	% CV	StdDev
(+)E1A_r60_3	0.30	0.35	32.44	0.12
(+)E1A_r60_a104	1.30	0.56	37.20	0.16
(+)E1A_r60_a107	2.30	1.29	19.28	0.08
(+)E1A_r60_a135	3.30	2.21	5.71	0.02
(+)E1A_r60_a20	3.83	2.64	3.81	0.02
(+)E1A_r60_a22	4.30	3.09	4.66	0.02
(+)E1A_r60_a97	4.82	3.84	4.85	0.02
(+)E1A_r60_n11	5.30	4.36	5.18	0.02
(+)E1A_r60_n9	5.82	4.77	4.74	0.02
(+)E1A_r60_1	6.30	5.32	4.56	0.02

Agilent SpikeIns: %CV of Avg. Processed Signal Plot

Spatial Distribution of Median Signals for each Row

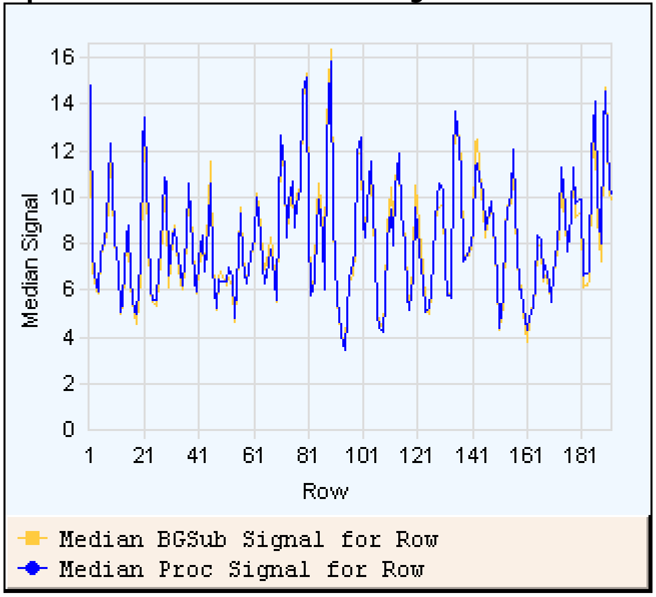

Spatial Distribution of Median Signals for each Column

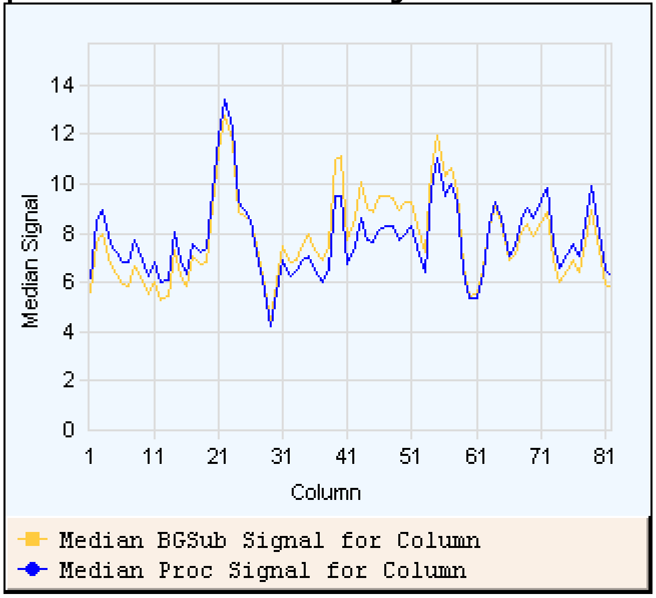

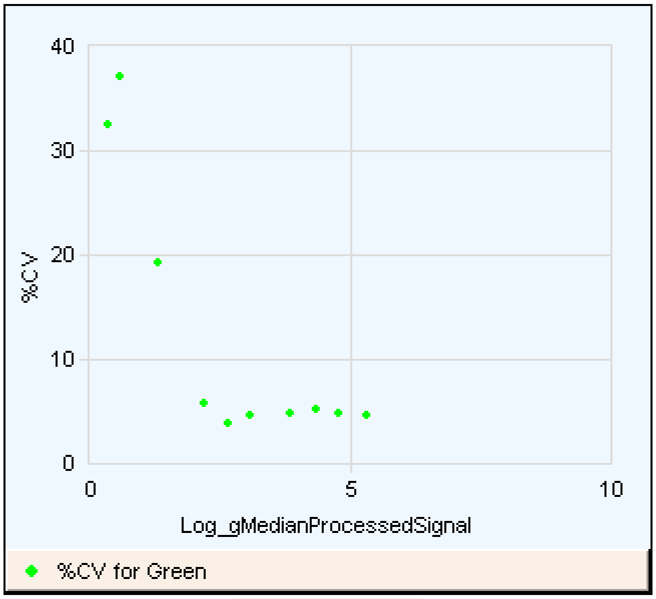

Median %CV:4.74

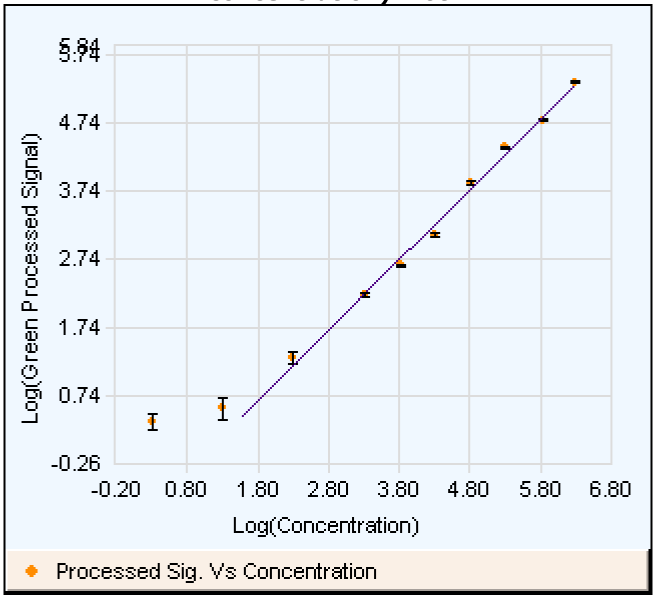

Agilent Spike-In Concentration-Response Statistics

Linear Range Statistics:

Low Signal	0.45
High Signal	5.67
Low Relative Concentration	1.58
High Relative Concentration	6.67
Slope	1.03
R^2 Value	1.00

Signal Detection Limit Statistics

Saturation Point	5.77
Low Threshold	0.19
Low Threshold Error	0.20
Spike-In Detection Limit	0.71

QC Report - Agilent Technologies : 1 Color Gene Expression

Date	Tuesday, September 20, 2011 - 11:10	Grid	035923_D_F_20110809
Image	UniversityPadova_253592310004_S01_H [2_1]	BG Method	No Background
Protocol	GE1-v5_95_Feb07 (Read Only)	Background Detrend	On(FeatNCRange, LoPass)
User Name	Administrator	Multiplicative Detrend	True
FE Version	9.5.1.1	Additive Error	2(Green)
		Saturation Value	587586 (g)

Spot Finding of the Four Corners of the Array

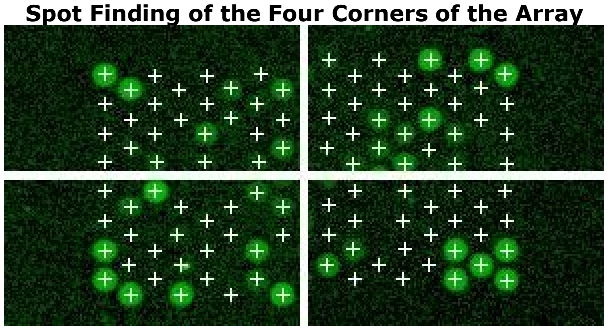

Grid Normal

	Feature	Local Background
	Green	Green
Non Uniform	5	13
Population	15	123

Spatial Distribution of All Outliers on the Array

192 rows x 82 columns

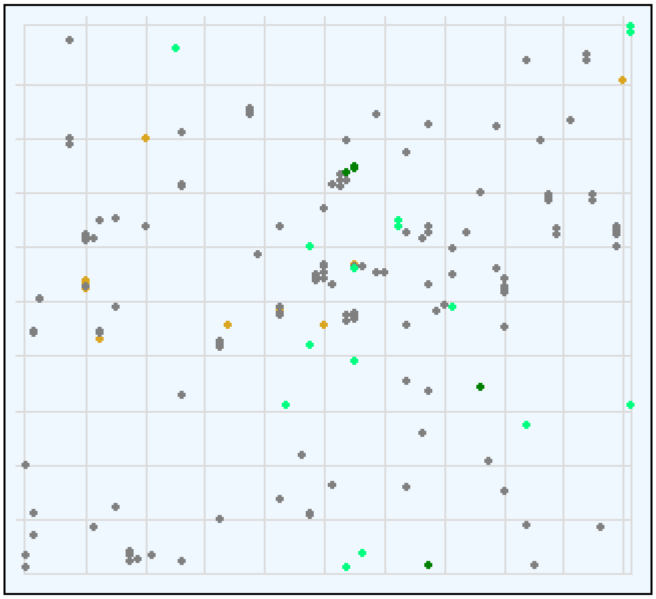

FeatureNonUnif (Green) = 5(0.04%)

GeneNonUnif (Green) = 5 (0.037 %)

BG NonUniform BG Population

Green FeaturePopulation Green Feature NonUniform

Negative Control Stats

	Green
Average Net Signals	15.52

Net Signal Statistics

Agilent SpikeIns:

	Green
# Saturated Features	0
99% of Sig. Distrib.	196127
50% of Sig. Distrib.	515
1% of Sig. Distrib.	14

Non-Control probes:

	Green
# Saturated Features	0
99% of Sig. Distrib.	19840
50% of Sig. Distrib.	22
1% of Sig. Distrib.	12

Histogram of Signals Plot

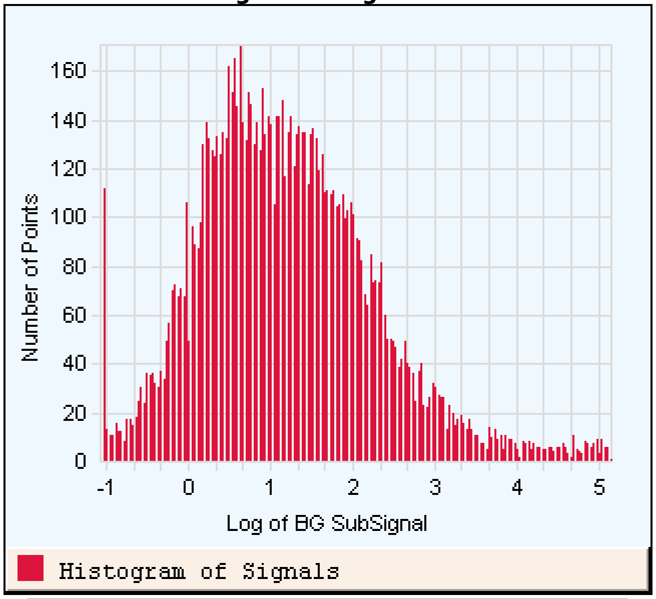

Features (NonCtrl) with BGSubSignal < 0: 2972 (Green)

StdDev Net Signals	2.01
Average BG Sub Signal	-1.09
StdDev BG Sub Signal	1.45

Local Bkg (inliers)

	Green
Number	14073
Avg	43.74
SD	2.23

Foreground Surface Fit

	Green
RMS_Fit	1.29
RMS_Resid	1.76
Avg_Fit	45.88

Multiplicative Surface Fit

	Green
RMS_Fit	0.12

Reproducibility: %CV for Replicated Probes

	Median %CV Signal (inliers)	
	Non-Control probes	Agilent SpikeIns
	Green	Green
BGSubSignal	-1.00	13.44
ProcessedSignal	-1.00	4.41

Agilent SpikeIns Signal Statistics

Probe Name	Log (Relative Conc.)	Median (Log Proc. Sig.)	% CV	StdDev
(+)E1A_r60_3	0.30	0.30	68.76	0.18
(+)E1A_r60_a104	1.30	0.50	34.12	0.15
(+)E1A_r60_a107	2.30	1.25	19.60	0.09
(+)E1A_r60_a135	3.30	2.18	6.63	0.03
(+)E1A_r60_a20	3.83	2.60	3.77	0.02
(+)E1A_r60_a22	4.30	3.04	2.47	0.01
(+)E1A_r60_a97	4.82	3.80	4.41	0.02
(+)E1A_r60_n11	5.30	4.36	4.48	0.02
(+)E1A_r60_n9	5.82	4.70	6.07	0.03
(+)E1A_r60_1	6.30	5.25	4.09	0.02

Agilent SpikeIns: %CV of Avg. Processed Signal Plot

Spatial Distribution of Median Signals for each Row

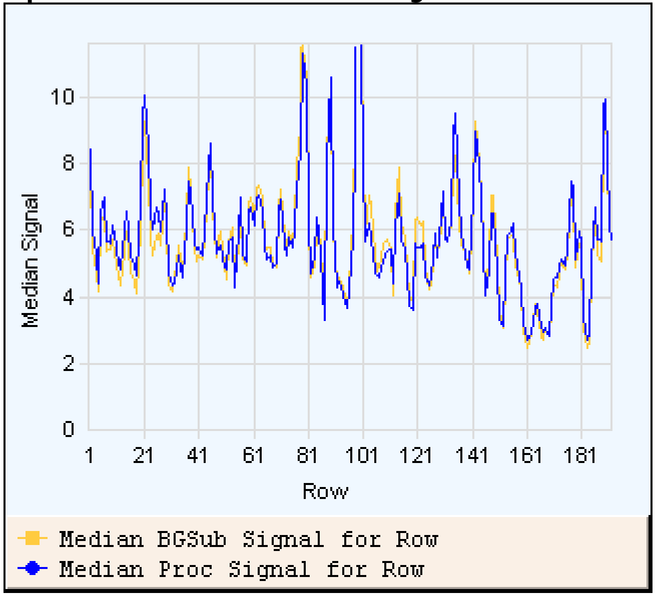

Spatial Distribution of Median Signals for each Column

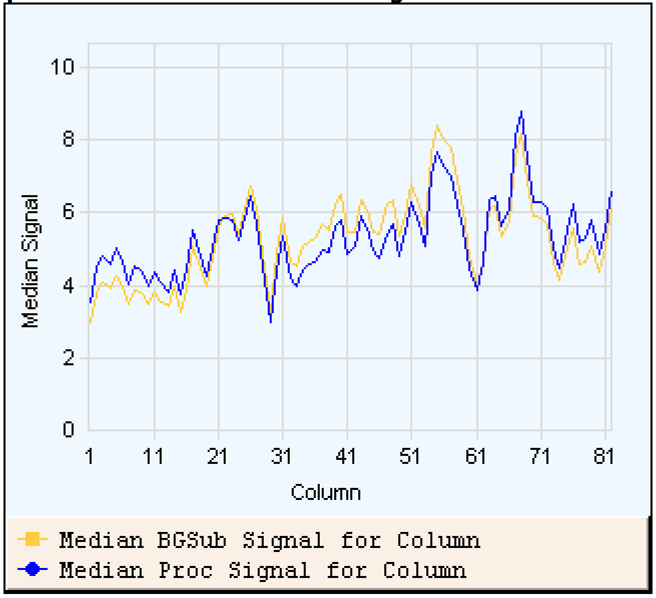

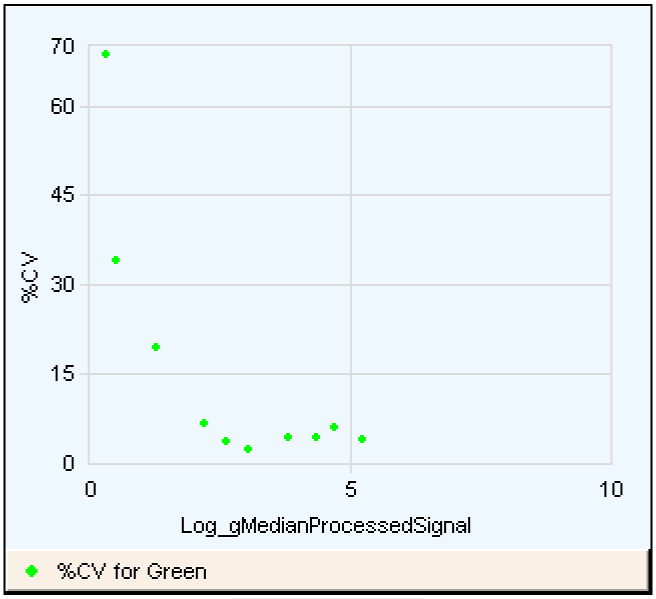

Median %CV:4.41

Agilent SpikeIns: Log(Signal) vs. Log(Relative concentration) Plot

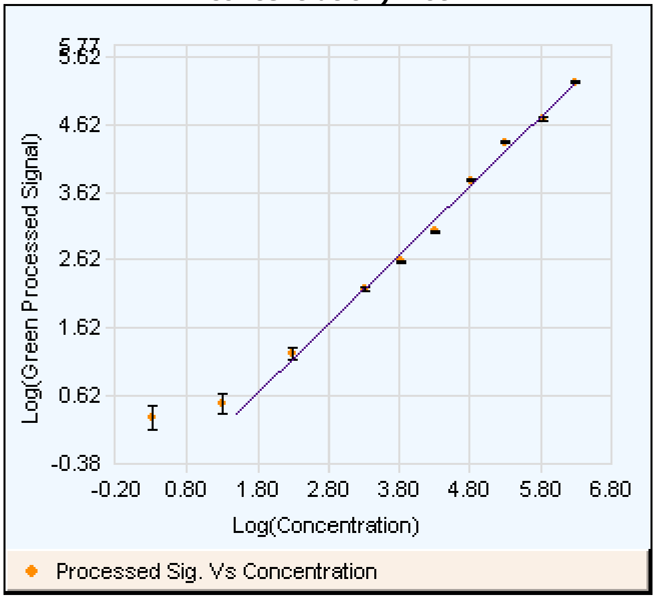

Agilent Spike-In Concentration-Response Statistics

Linear Range Statistics:

Low Signal	0.35
High Signal	5.68
Low Relative Concentration	1.51
High Relative Concentration	6.74
Slope	1.02
R^2 Value	0.99

Signal Detection Limit Statistics

Saturation Point	5.77
Low Threshold	0.11
Low Threshold Error	0.23
Spike-In Detection Limit	0.62

QC Report - Agilent Technologies : 1 Color Gene Expression

Date	Friday, September 02, 2011 - 10:55	Grid	035923_D_F_20110809
Image	UniversityPadova_253592310001_S01_H [2_1]	BG Method	No Background
Protocol	GE1-v5_95_Feb07 (Read Only)	Background Detrend	On(FeatNCRange, LoPass)
User Name	Administrator	Multiplicative Detrend	True
FE Version	9.5.1.1	Additive Error	1(Green)
		Saturation Value	607759 (g)

Spot Finding of the Four Corners of the Array

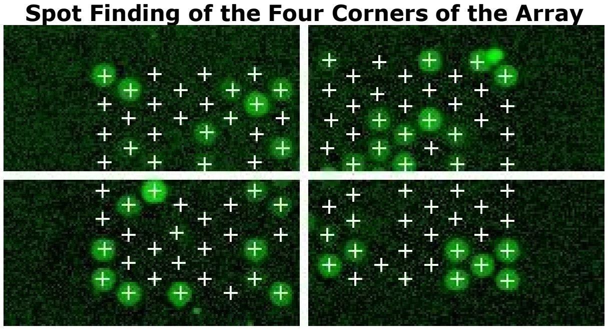

Grid Normal

	Feature	Local Background
	Green	Green
Non Uniform	4	30
Population	20	170

Spatial Distribution of All Outliers on the Array

192 rows x 82 columns

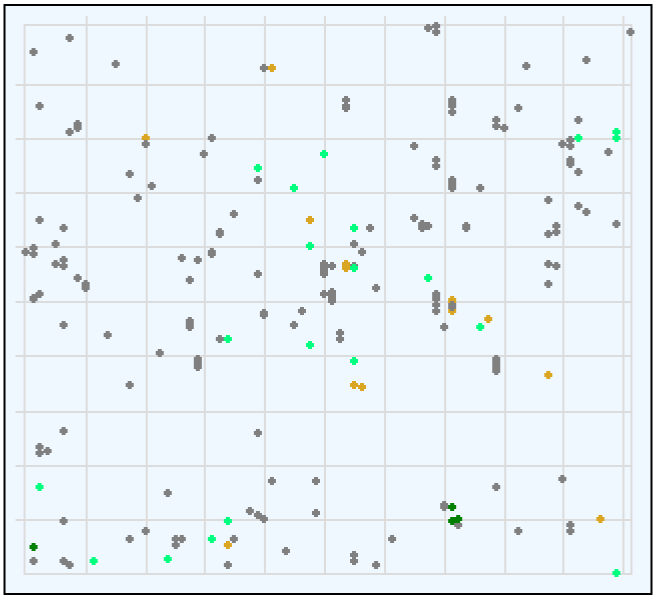

FeatureNonUnif (Green) = 4(0.03%)

GeneNonUnif (Green) = 4 (0.029 %)

BG NonUniform | BG Population | Green FeaturePopulation | Green Feature NonUniform

Negative Control Stats

	Green
Average Net Signals	10.92

Net Signal Statistics

Agilent SpikeIns:

	Green
# Saturated Features	0
99% of Sig. Distrib.	196073
50% of Sig. Distrib.	568
1% of Sig. Distrib.	9

Non-Control probes:

	Green
# Saturated Features	0
99% of Sig. Distrib.	21717
50% of Sig. Distrib.	18
1% of Sig. Distrib.	9

Histogram of Signals Plot

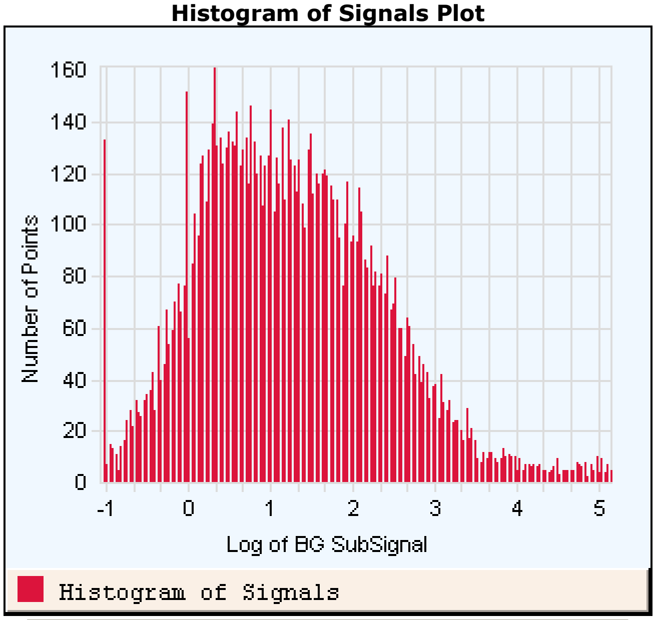

Features (NonCtrl) with BGSubSignal < 0: 2662 (Green)

StdDev Net Signals	1.36
Average BG Sub Signal	-1.00
StdDev BG Sub Signal	1.45

Local Bkg (inliers)

	Green
Number	14023
Avg	42.99
SD	2.10

Foreground Surface Fit

	Green
RMS_Fit	0.99
RMS_Resid	1.42
Avg_Fit	40.59

Multiplicative Surface Fit

	Green
RMS_Fit	0.12

Reproducibility: %CV for Replicated Probes

	Median %CV Signal (inliers)	
	Non-Control probes	Agilent SpikeIns
	Green	Green
BGSubSignal	-1.00	13.65
ProcessedSignal	-1.00	4.60

Agilent SpikeIns Signal Statistics

Probe Name	Log (Relative Conc.)	Median (Log Proc. Sig.)	% CV	StdDev
(+)E1A_r60_3	0.30	0.20	34.72	0.13
(+)E1A_r60_a104	1.30	0.50	48.15	0.21
(+)E1A_r60_a107	2.30	1.24	19.31	0.10
(+)E1A_r60_a135	3.30	2.18	7.45	0.03
(+)E1A_r60_a20	3.83	2.63	3.70	0.02
(+)E1A_r60_a22	4.30	3.07	2.37	0.01
(+)E1A_r60_a97	4.82	3.76	4.60	0.02
(+)E1A_r60_n11	5.30	4.34	3.95	0.02
(+)E1A_r60_n9	5.82	4.68	7.89	0.04
(+)E1A_r60_1	6.30	5.27	5.22	0.02

Agilent SpikeIns: %CV of Avg. Processed Signal Plot

Spatial Distribution of Median Signals for each Row

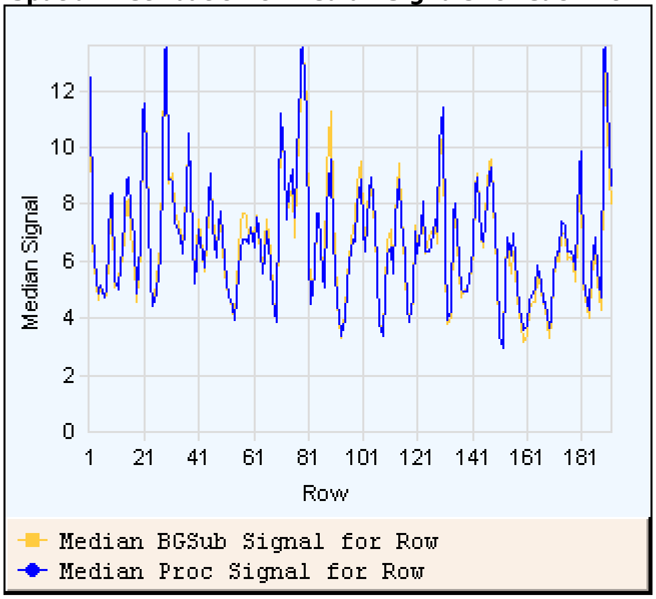

Spatial Distribution of Median Signals for each Column

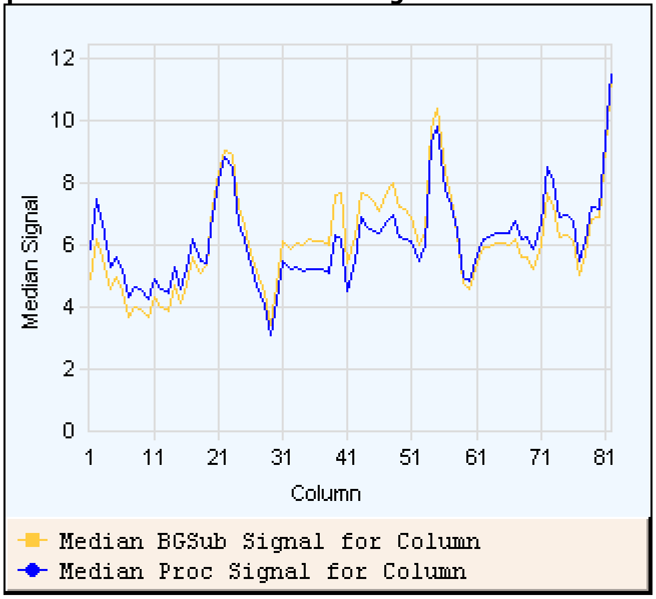

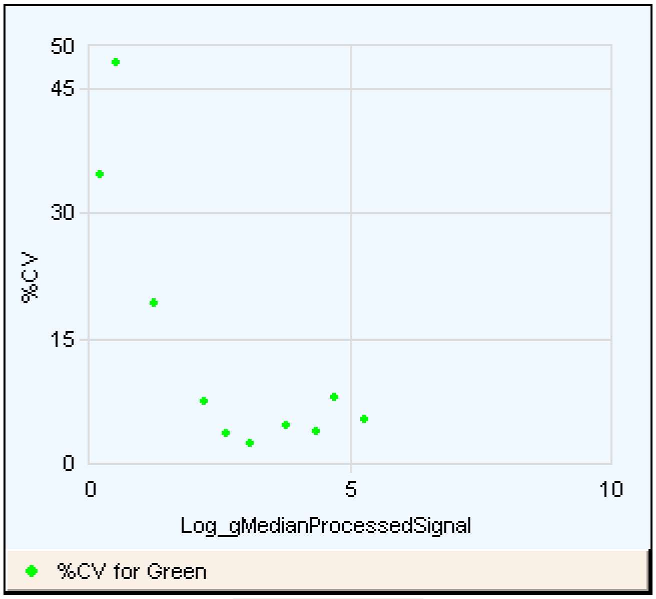

Median %CV:4.60

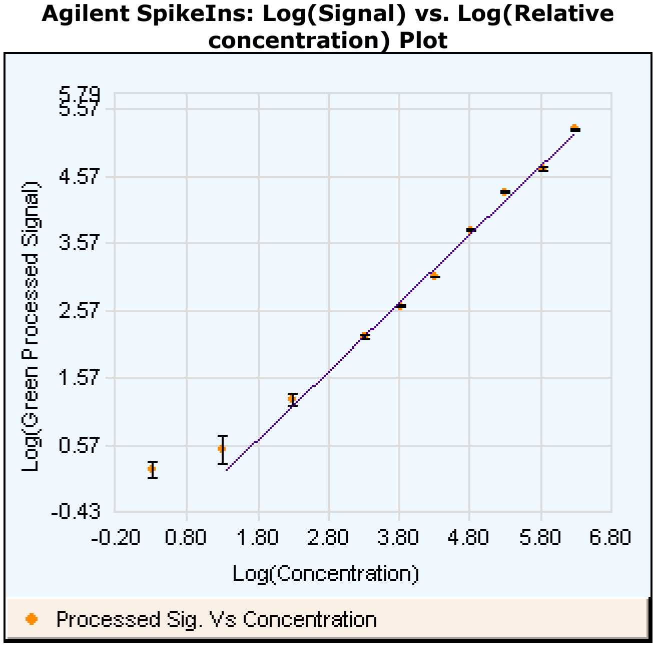

Agilent Spike-In Concentration-Response Statistics

Linear Range Statistics:

Low Signal	0.19
High Signal	5.72
Low Relative Concentration	1.35
High Relative Concentration	6.78
Slope	1.02
R^2 Value	1.00

Signal Detection Limit Statistics

Saturation Point	5.78
Low Threshold	-0.02
Low Threshold Error	0.24
Spike-In Detection Limit	0.67

QC Report - Agilent Technologies : 1 Color Gene Expression

Date	Tuesday, September 20, 2011 - 11:05	Grid	035923_D_F_20110809
Image	UniversityPadova_253592310003_S01_H [1_1]	BG Method	No Background
Protocol	GE1-v5_95_Feb07 (Read Only)	Background Detrend	On(FeatNCRange, LoPass)
User Name	Administrator	Multiplicative Detrend	True
FE Version	9.5.1.1	Additive Error	2(Green)
		Saturation Value	586357 (g)

Spot Finding of the Four Corners of the Array

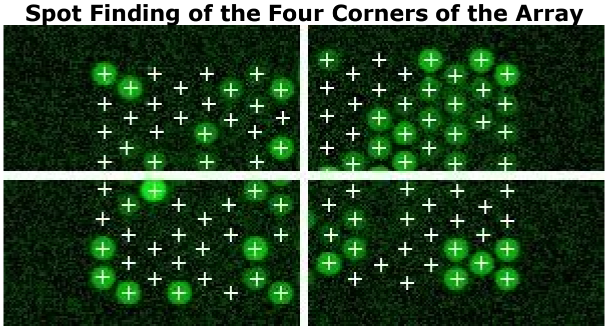

Grid Normal

	Feature Green	Local Background Green
Non Uniform	1	14
Population	25	94

Spatial Distribution of All Outliers on the Array

192 rows x 82 columns

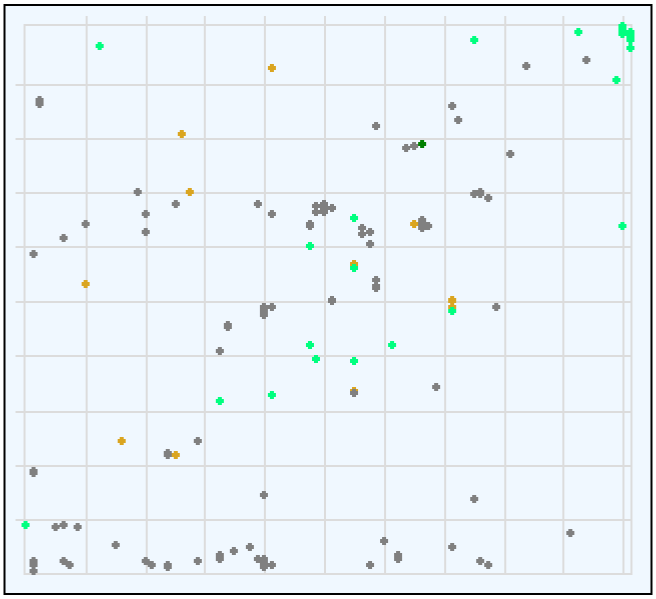

FeatureNonUnif (Green) = 1(0.01%)

GeneNonUnif (Green) = 1 (0.007 %)

BG NonUniform · BG Population · Green FeaturePopulation · Green Feature NonUniform

Negative Control Stats

	Green
Average Net Signals	15.35

Net Signal Statistics

Agilent SpikeIns:

	Green
# Saturated Features	0
99% of Sig. Distrib.	162881
50% of Sig. Distrib.	368
1% of Sig. Distrib.	15

Non-Control probes:

	Green
# Saturated Features	0
99% of Sig. Distrib.	35678
50% of Sig. Distrib.	29
1% of Sig. Distrib.	13

Histogram of Signals Plot

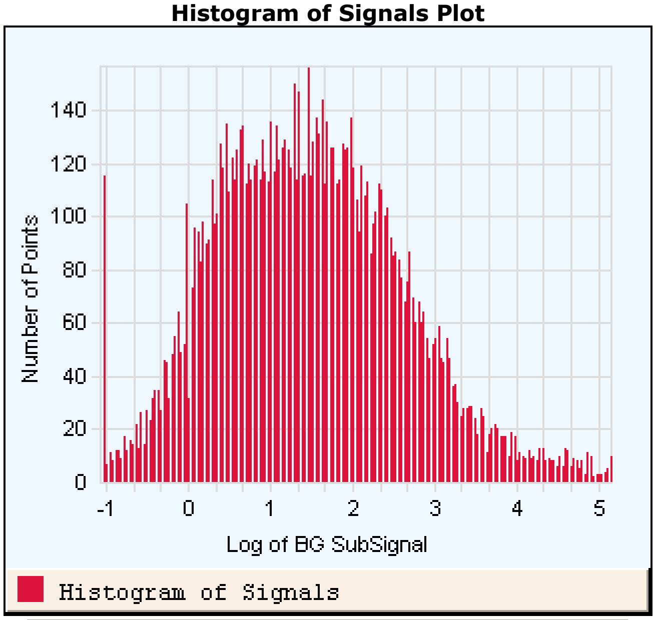

Features (NonCtrl) with BGSubSignal < 0: 2178 (Green)

StdDev Net Signals	1.78
Average BG Sub Signal	-1.32
StdDev BG Sub Signal	1.63

Local Bkg (inliers)

	Green
Number	14101
Avg	45.71
SD	2.70

Foreground Surface Fit

	Green
RMS_Fit	0.99
RMS_Resid	1.70
Avg_Fit	45.83

Multiplicative Surface Fit

	Green
RMS_Fit	0.13

Reproducibility: %CV for Replicated Probes

	Median %CV Signal (inliers)	
	Non-Control probes	Agilent SpikeIns
	Green	Green
BGSubSignal	-1.00	12.87
ProcessedSignal	-1.00	4.83

Agilent SpikeIns Signal Statistics

Probe Name	Log (Relative Conc.)	Median (Log Proc. Sig.)	% CV	StdDev
(+)E1A_r60_3	0.30	0.30	33.71	0.13
(+)E1A_r60_a104	1.30	0.35	60.40	0.22
(+)E1A_r60_a107	2.30	1.05	29.85	0.20
(+)E1A_r60_a135	3.30	1.98	6.80	0.03
(+)E1A_r60_a20	3.83	2.44	4.20	0.02
(+)E1A_r60_a22	4.30	2.91	3.59	0.02
(+)E1A_r60_a97	4.82	3.64	5.02	0.02
(+)E1A_r60_n11	5.30	4.17	4.83	0.02
(+)E1A_r60_n9	5.82	4.56	5.89	0.03
(+)E1A_r60_1	6.30	5.13	3.46	0.02

Agilent SpikeIns: %CV of Avg. Processed Signal Plot

Spatial Distribution of Median Signals for each Row

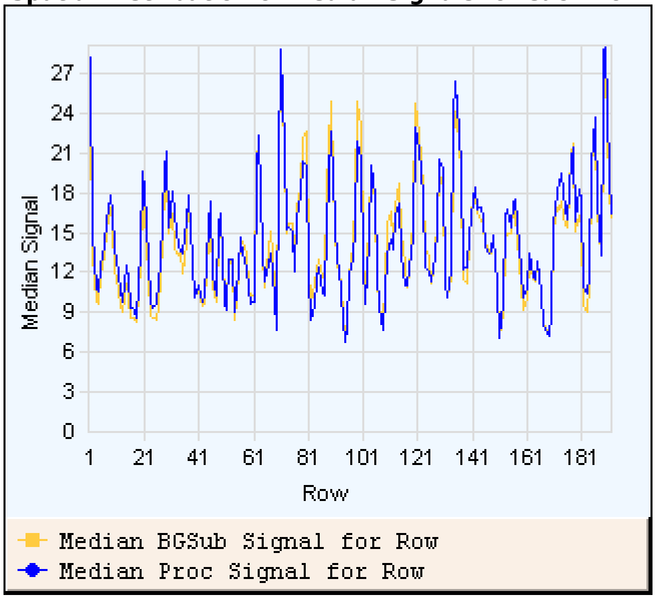

Spatial Distribution of Median Signals for each Column

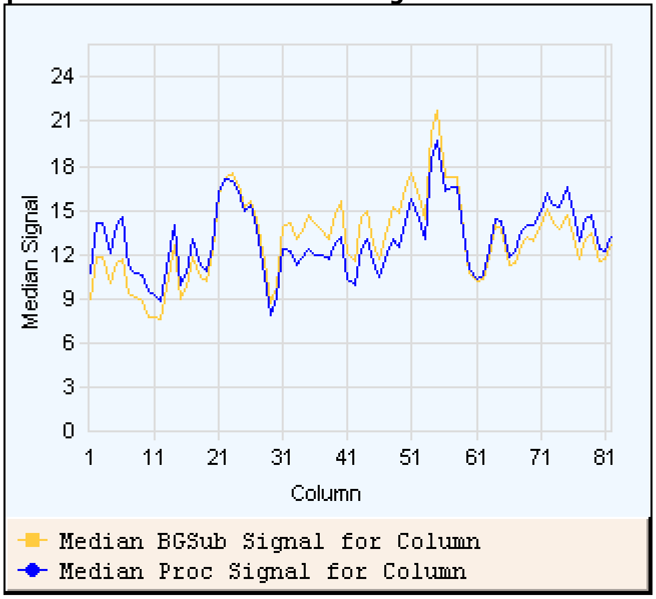

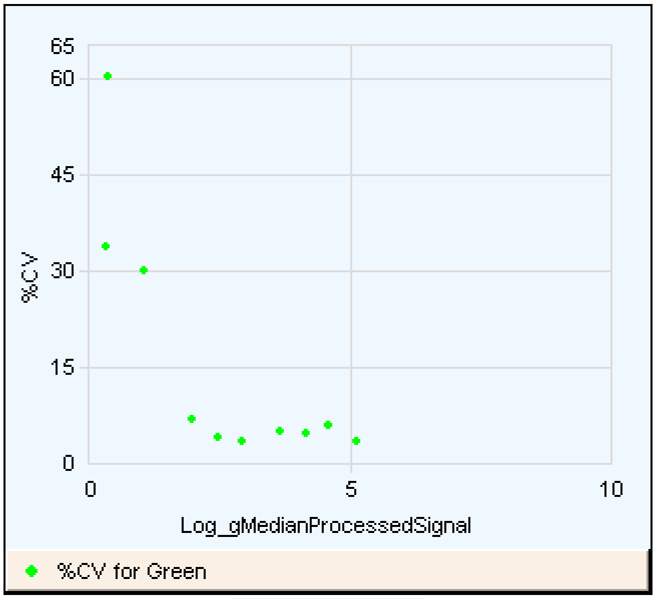

Median %CV:4.83

Agilent SpikeIns: Log(Signal) vs. Log(Relative concentration) Plot

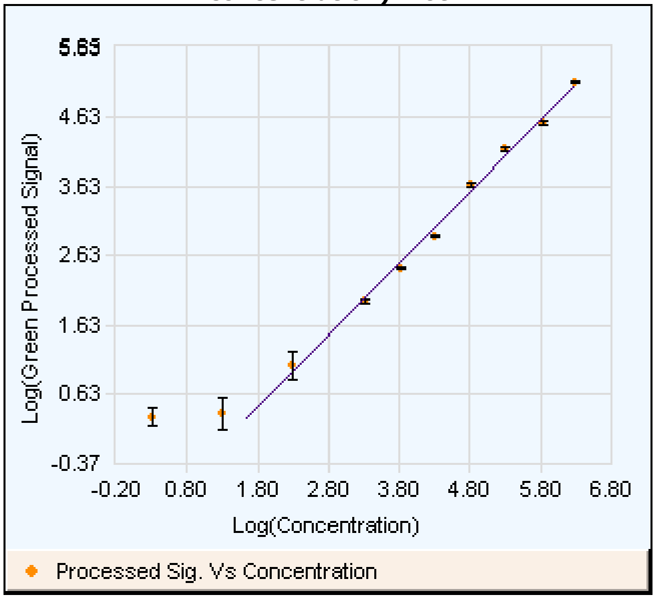

Agilent Spike-In Concentration-Response Statistics

Linear Range Statistics:

Low Signal	0.29
High Signal	5.70
Low Relative Concentration	1.65
High Relative Concentration	6.87
Slope	1.04
R^2 Value	1.00

Signal Detection Limit Statistics

Saturation Point	5.77
Low Threshold	0.06
Low Threshold Error	0.33
Spike-In Detection Limit	0.67

QC Report - Agilent Technologies : 1 Color Gene Expression

Date	Tuesday, September 20, 2011 - 11:10	Grid	035923_D_F_20110809
Image	UniversityPadova_253592310004_S01_H [2_2]	BG Method	No Background
Protocol	GE1-v5_95_Feb07 (Read Only)	Background Detrend	On(FeatNCRange, LoPass)
User Name	Administrator	Multiplicative Detrend	True
FE Version	9.5.1.1	Additive Error	2(Green)
		Saturation Value	586559 (g)

Spot Finding of the Four Corners of the Array

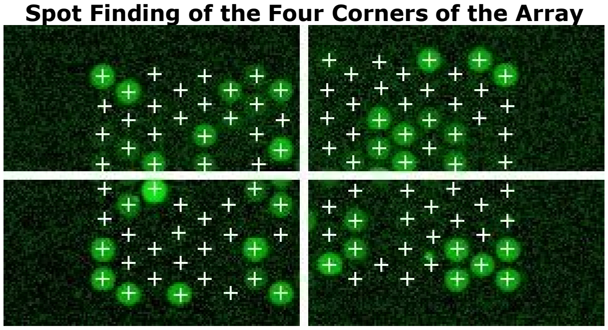

Grid Normal

	Feature	Local Background
	Green	Green
Non Uniform	0	21
Population	13	176

Spatial Distribution of All Outliers on the Array

192 rows x 82 columns

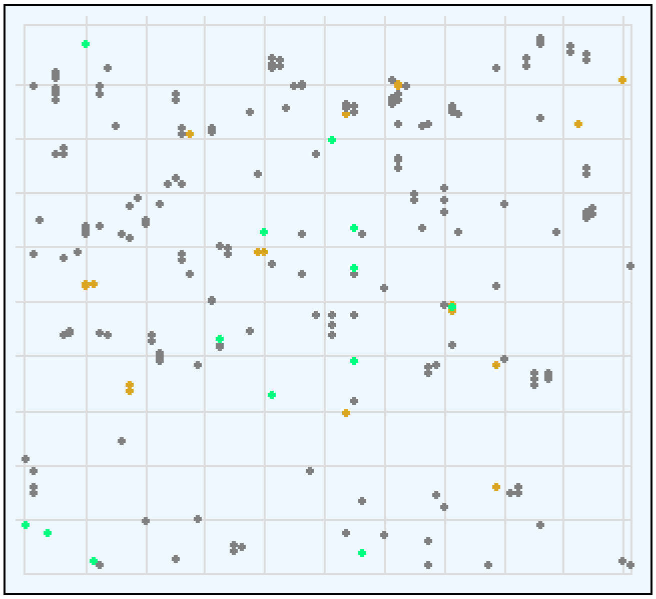

FeatureNonUnif (Green) = 0(0.00%)

GeneNonUnif (Green) = 0 (0.000 %)

BG NonUniform   BG Population

Green FeaturePopulation   Green Feature NonUniform

Negative Control Stats

	Green
Average Net Signals	15.99

Net Signal Statistics

Agilent SpikeIns:

	Green
# Saturated Features	0
99% of Sig. Distrib.	193748
50% of Sig. Distrib.	476
1% of Sig. Distrib.	14

Non-Control probes:

	Green
# Saturated Features	0
99% of Sig. Distrib.	33830
50% of Sig. Distrib.	28
1% of Sig. Distrib.	13

Histogram of Signals Plot

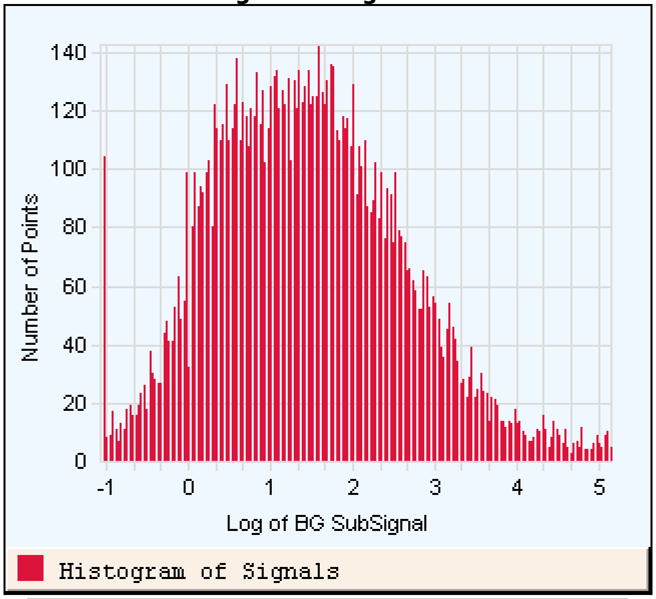

Features (NonCtrl) with BGSubSignal < 0: 2456 (Green)

StdDev Net Signals	2.27
Average BG Sub Signal	-1.39
StdDev BG Sub Signal	1.67

Local Bkg (inliers)

	Green
Number	14013
Avg	43.87
SD	2.15

Foreground Surface Fit

	Green
RMS_Fit	1.72
RMS_Resid	1.51
Avg_Fit	46.61

Multiplicative Surface Fit

	Green
RMS_Fit	0.11

Reproducibility: %CV for Replicated Probes

	Median %CV Signal (inliers)	
	Non-Control probes	Agilent SpikeIns
	Green	Green
BGSubSignal	-1.00	12.44
ProcessedSignal	-1.00	3.30

Agilent SpikeIns Signal Statistics

Probe Name	Log (Relative Conc.)	Median (Log Proc. Sig.)	% CV	StdDev
(+)E1A_r60_3	0.30	0.23	55.35	0.18
(+)E1A_r60_a104	1.30	0.36	359.34	0.50
(+)E1A_r60_a107	2.30	1.22	12.76	0.05
(+)E1A_r60_a135	3.30	2.09	5.17	0.02
(+)E1A_r60_a20	3.83	2.58	3.30	0.01
(+)E1A_r60_a22	4.30	2.99	2.65	0.01
(+)E1A_r60_a97	4.82	3.75	3.07	0.01
(+)E1A_r60_n11	5.30	4.29	3.92	0.02
(+)E1A_r60_n9	5.82	4.68	2.66	0.01
(+)E1A_r60_1	6.30	5.25	4.25	0.02

Agilent SpikeIns: %CV of Avg. Processed Signal Plot

Spatial Distribution of Median Signals for each Row

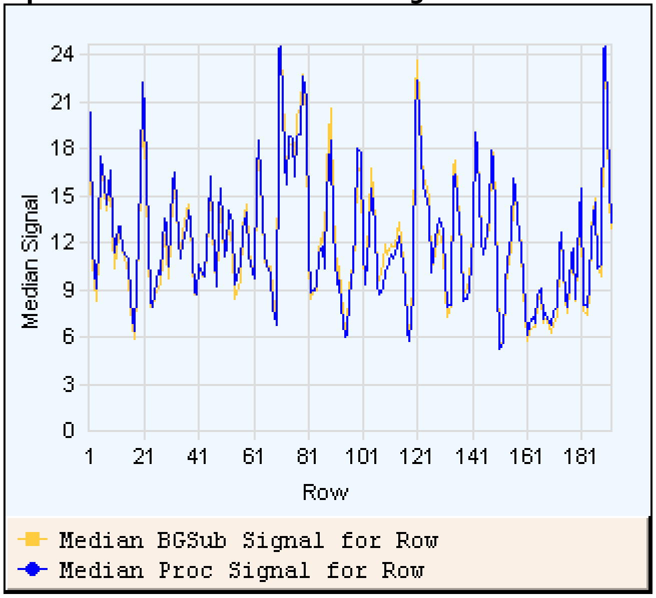

Spatial Distribution of Median Signals for each Column

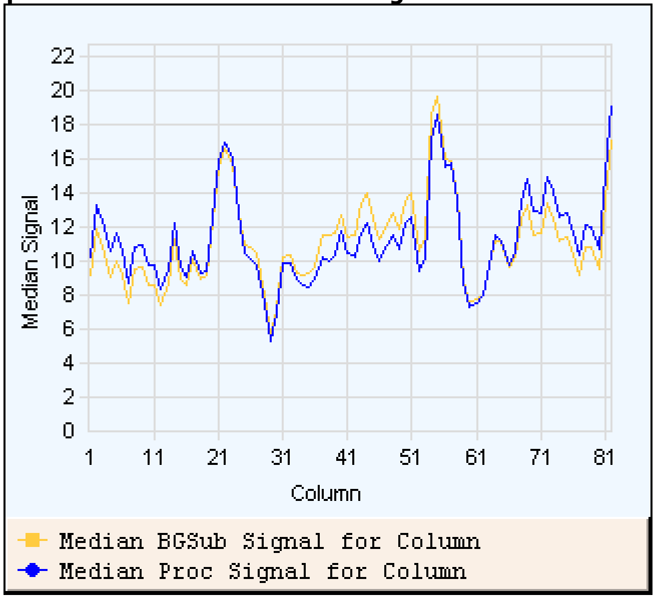

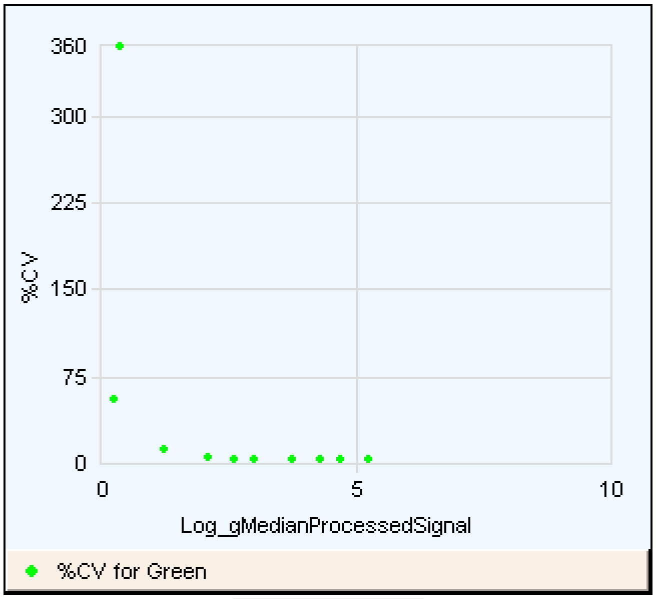

Median %CV:3.30

Agilent SpikeIns: Log(Signal) vs. Log(Relative concentration) Plot

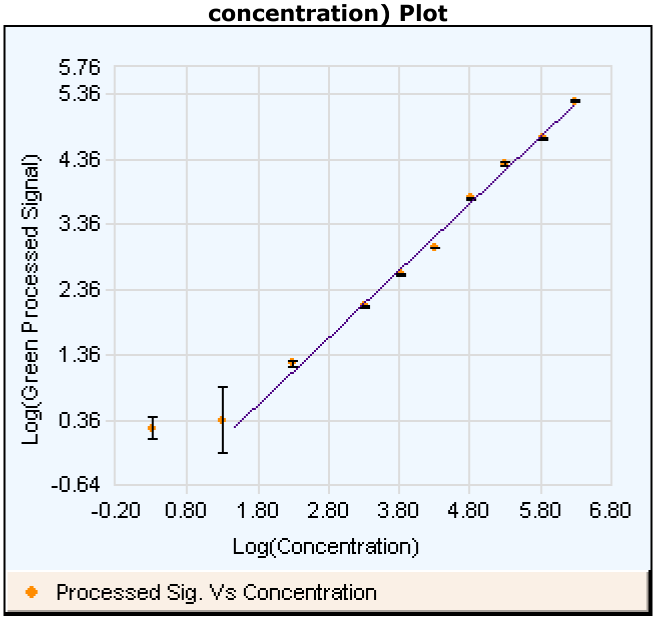

Agilent Spike-In Concentration-Response Statistics

Linear Range Statistics:

Low Signal	0.26
High Signal	5.68
Low Relative Concentration	1.48
High Relative Concentration	6.76
Slope	1.03
R^2 Value	0.99

Signal Detection Limit Statistics

Saturation Point	5.77
Low Threshold	0.00
Low Threshold Error	0.53
Spike-In Detection Limit	1.76

QC Report - Agilent Technologies : 1 Color Gene Expression

Date	Tuesday, September 20, 2011 - 11:10	Grid	035923_D_F_20110809
Image	UniversityPadova_253592310004_S01_H [2_3]	BG Method	No Background
Protocol	GE1-v5_95_Feb07 (Read Only)	Background Detrend	On(FeatNCRange, LoPass)
User Name	Administrator	Multiplicative Detrend	True
FE Version	9.5.1.1	Additive Error	2(Green)
		Saturation Value	587044 (g)

Spot Finding of the Four Corners of the Array

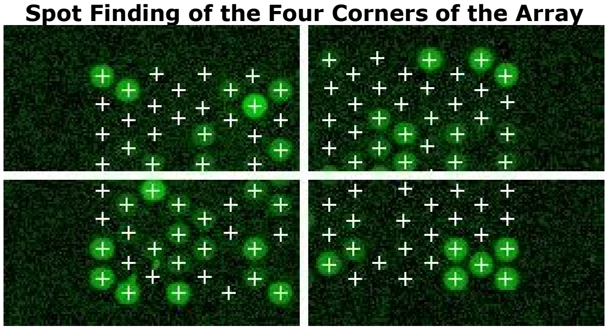

Grid Normal

	Feature Green	Local Background Green
Non Uniform	3	34
Population	12	147

Spatial Distribution of All Outliers on the Array

192 rows x 82 columns

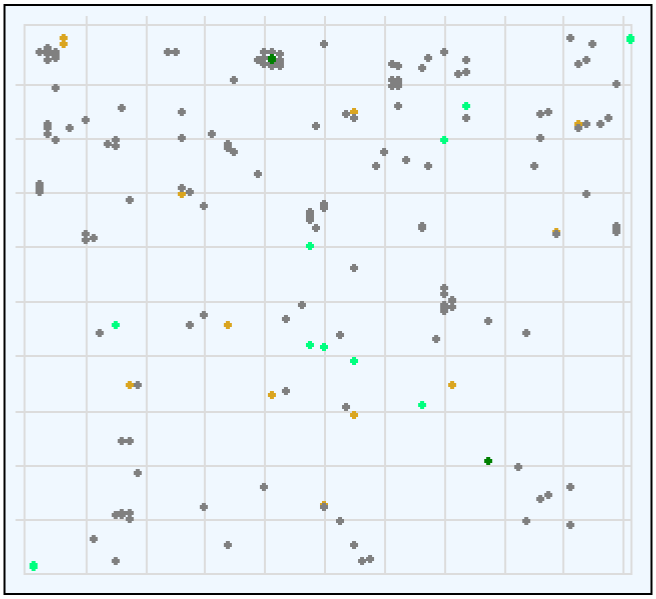

FeatureNonUnif (Green) = 3(0.02%)

GeneNonUnif (Green) = 3 (0.022 %)

BG NonUniform   BG Population

Green FeaturePopulation   Green Feature NonUniform

Negative Control Stats

	Green
Average Net Signals	16.36

Net Signal Statistics

Agilent SpikeIns:

	Green
# Saturated Features	0
99% of Sig. Distrib.	226303
50% of Sig. Distrib.	554
1% of Sig. Distrib.	16

Non-Control probes:

	Green
# Saturated Features	1
99% of Sig. Distrib.	15489
50% of Sig. Distrib.	24
1% of Sig. Distrib.	12

Histogram of Signals Plot

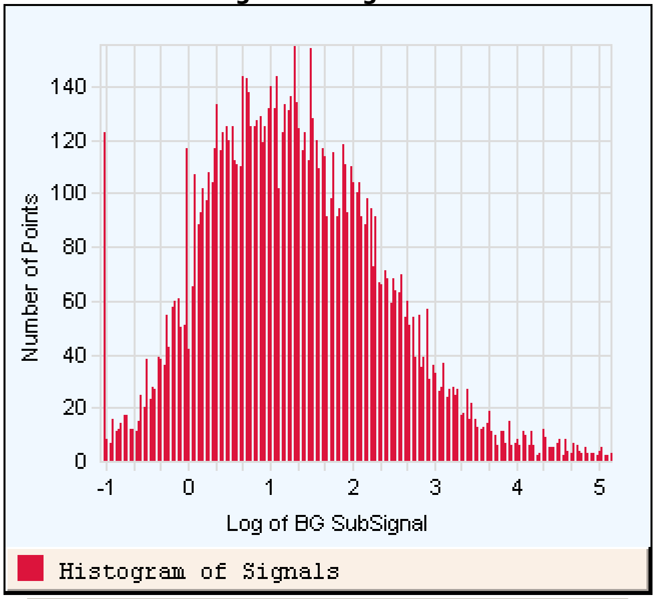

Features (NonCtrl) with BGSubSignal < 0: 3162 (Green)

StdDev Net Signals	2.56
Average BG Sub Signal	-1.57
StdDev BG Sub Signal	1.73

Local Bkg (inliers)

	Green
Number	14048
Avg	43.65
SD	2.17

Foreground Surface Fit

	Green
RMS_Fit	2.04
RMS_Resid	1.78
Avg_Fit	47.21

Multiplicative Surface Fit

	Green
RMS_Fit	0.11

Reproducibility: %CV for Replicated Probes

	Median %CV Signal (inliers)	
	Non-Control probes	Agilent SpikeIns
	Green	Green
BGSubSignal	-1.00	12.03
ProcessedSignal	-1.00	3.53

Agilent SpikeIns Signal Statistics

Probe Name	Log (Relative Conc.)	Median (Log Proc. Sig.)	% CV	StdDev
(+)E1A_r60_3	0.30	0.27	45.45	0.16
(+)E1A_r60_a104	1.30	0.48	75.87	0.26
(+)E1A_r60_a107	2.30	1.21	21.19	0.09
(+)E1A_r60_a135	3.30	2.16	5.63	0.02
(+)E1A_r60_a20	3.83	2.64	3.53	0.02
(+)E1A_r60_a22	4.30	3.04	2.69	0.01
(+)E1A_r60_a97	4.82	3.79	4.08	0.02
(+)E1A_r60_n11	5.30	4.35	2.99	0.01
(+)E1A_r60_n9	5.82	4.74	2.99	0.01
(+)E1A_r60_1	6.30	5.31	3.65	0.02

Agilent SpikeIns: %CV of Avg. Processed Signal Plot

Spatial Distribution of Median Signals for each Row

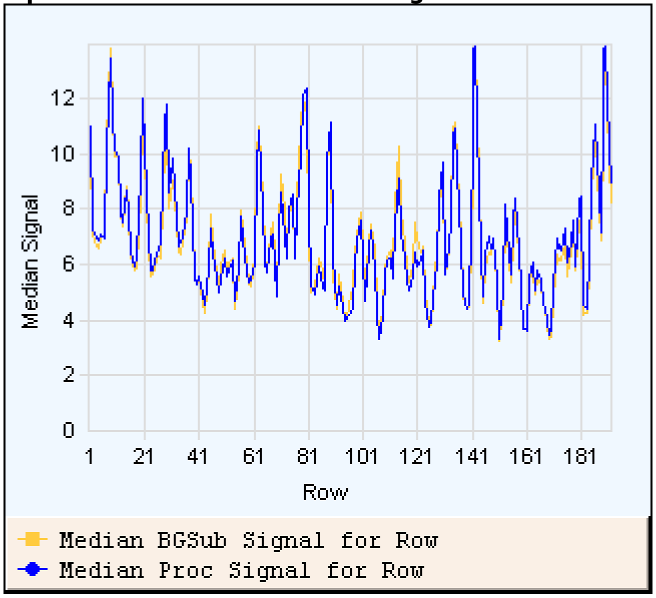

Spatial Distribution of Median Signals for each Column

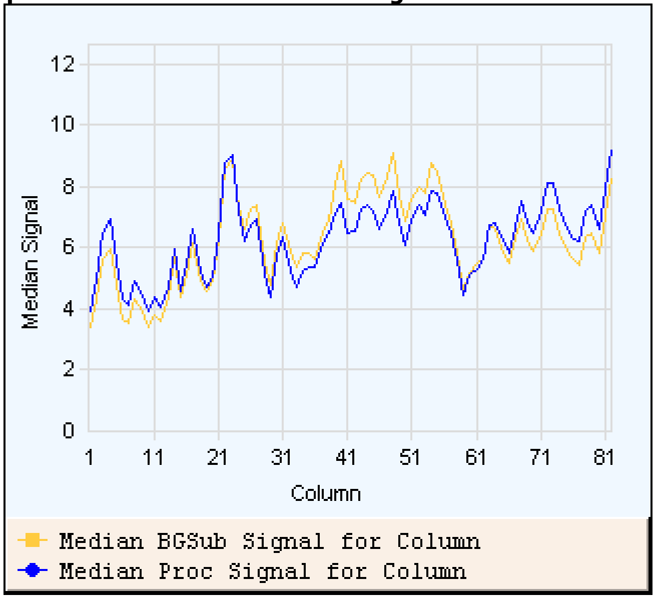

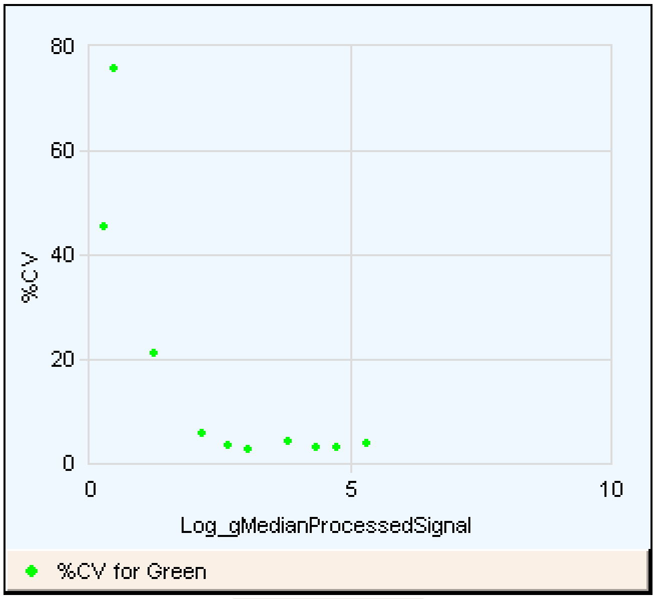

Median %CV:3.53

Agilent SpikeIns: Log(Signal) vs. Log(Relative concentration) Plot

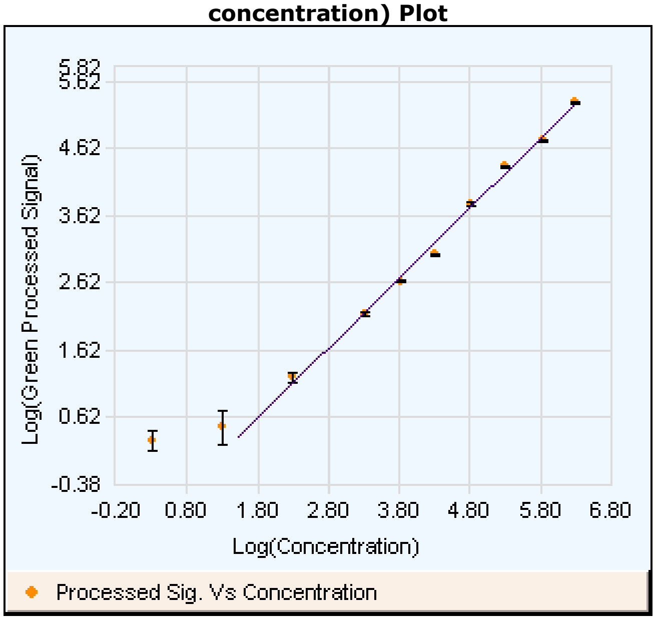

Agilent Spike-In Concentration-Response Statistics

Linear Range Statistics:

Low Signal	0.33
High Signal	5.68
Low Relative Concentration	1.54
High Relative Concentration	6.68
Slope	1.04
R^2 Value	1.00

Signal Detection Limit Statistics

Saturation Point	5.77
Low Threshold	0.09
Low Threshold Error	0.31
Spike-In Detection Limit	0.81

QC Report - Agilent Technologies : 1 Color Gene Expression

Date	Friday, September 23, 2011 - 11:02	Grid	035923_D_F_20110809
Image	UniversityPadova_253592310006_S01_H [1_2]	BG Method	No Background
Protocol	GE1-v5_95_Feb07 (Read Only)	Background Detrend	On(FeatNCRange, LoPass)
User Name	Administrator	Multiplicative Detrend	True
FE Version	9.5.1.1	Additive Error	2(Green)
		Saturation Value	586331 (g)

Spot Finding of the Four Corners of the Array

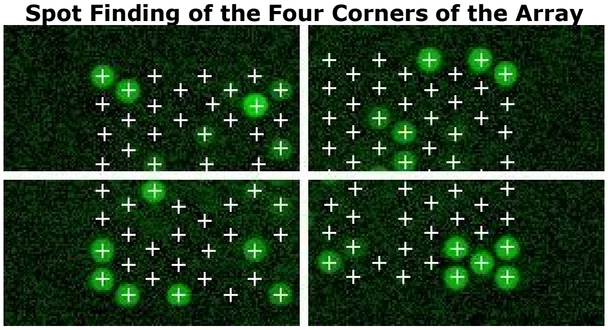

Grid Normal

	Feature	Local Background
	Green	Green
Non Uniform	0	16
Population	11	110

Spatial Distribution of All Outliers on the Array

192 rows x 82 columns

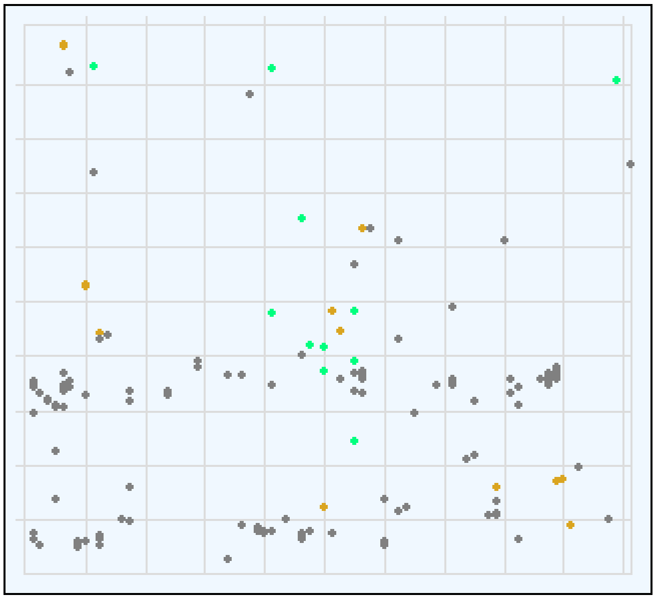

FeatureNonUnif (Green) = 0(0.00%)

GeneNonUnif (Green) = 0 (0.000 %)

BG NonUniform · BG Population

Green FeaturePopulation · Green Feature NonUniform

Negative Control Stats

	Green
Average Net Signals	13.24

Net Signal Statistics

Agilent SpikeIns:

	Green
# Saturated Features	0
99% of Sig. Distrib.	232427
50% of Sig. Distrib.	529
1% of Sig. Distrib.	12

Non-Control probes:

	Green
# Saturated Features	0
99% of Sig. Distrib.	10972
50% of Sig. Distrib.	18
1% of Sig. Distrib.	10

Histogram of Signals Plot

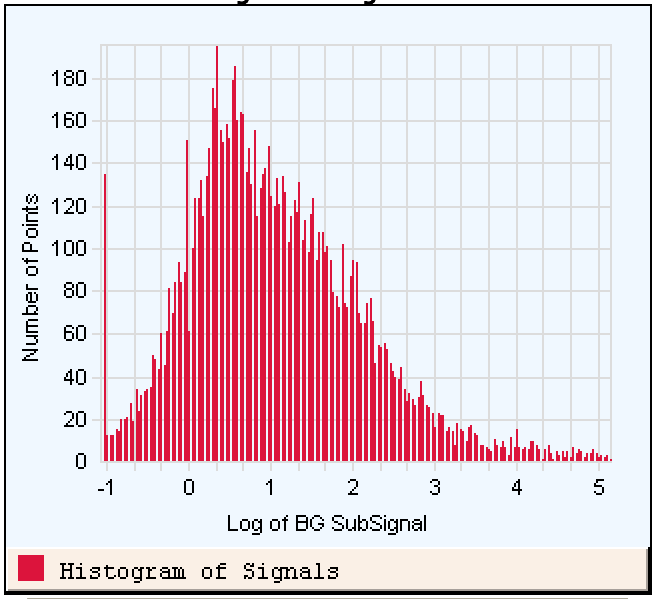

Features (NonCtrl) with BGSubSignal < 0: 3048 (Green)

StdDev Net Signals	2.00
Average BG Sub Signal	-1.10
StdDev BG Sub Signal	1.56

Local Bkg (inliers)

	Green
Number	14083
Avg	44.66
SD	2.52

Foreground Surface Fit

	Green
RMS_Fit	1.55
RMS_Resid	1.63
Avg_Fit	42.77

Multiplicative Surface Fit

	Green
RMS_Fit	0.06

Reproducibility: %CV for Replicated Probes

	Median %CV Signal (inliers)	
	Non-Control probes	Agilent SpikeIns
	Green	Green
BGSubSignal	-1.00	6.84
ProcessedSignal	-1.00	4.70

Agilent SpikeIns Signal Statistics

Probe Name	Log (Relative Conc.)	Median (Log Proc. Sig.)	% CV	StdDev
(+)E1A_r60_3	0.30	0.24	32.21	0.11
(+)E1A_r60_a104	1.30	0.67	45.96	0.21
(+)E1A_r60_a107	2.30	1.38	14.47	0.06
(+)E1A_r60_a135	3.30	2.29	6.23	0.03
(+)E1A_r60_a20	3.83	2.64	4.75	0.02
(+)E1A_r60_a22	4.30	3.23	4.10	0.02
(+)E1A_r60_a97	4.82	3.97	3.55	0.02
(+)E1A_r60_n11	5.30	4.52	3.99	0.02
(+)E1A_r60_n9	5.82	4.85	5.72	0.02
(+)E1A_r60_1	6.30	5.35	4.70	0.02

Agilent SpikeIns: %CV of Avg. Processed Signal Plot

Spatial Distribution of Median Signals for each Row

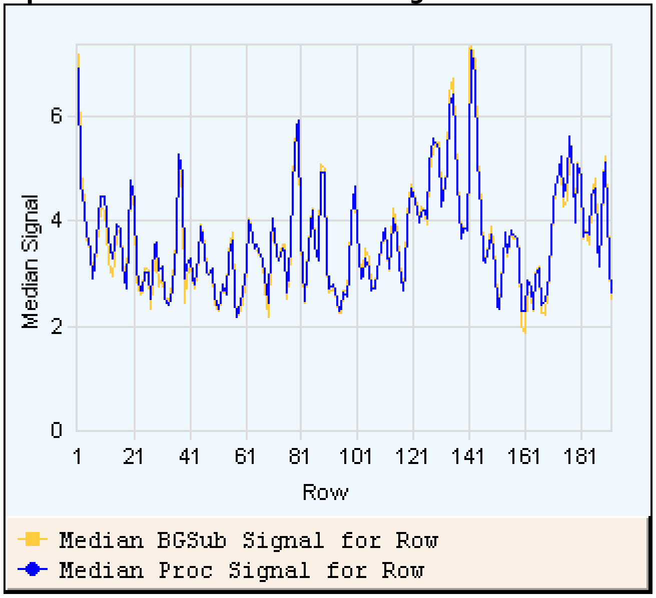

Spatial Distribution of Median Signals for each Column

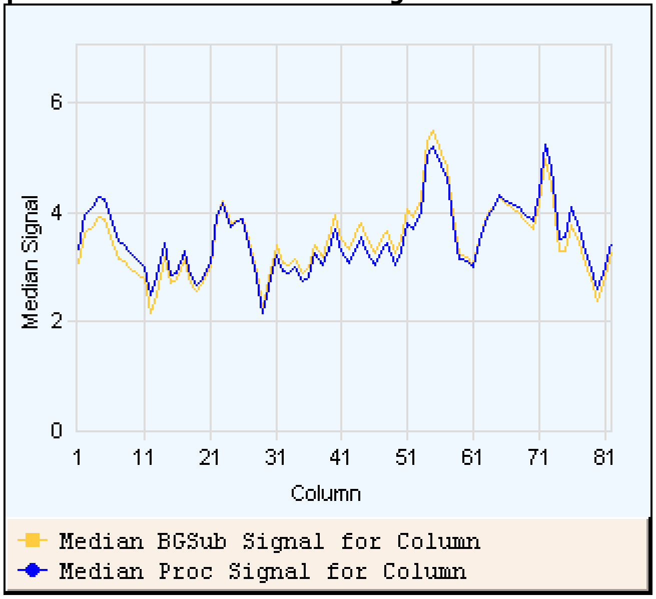

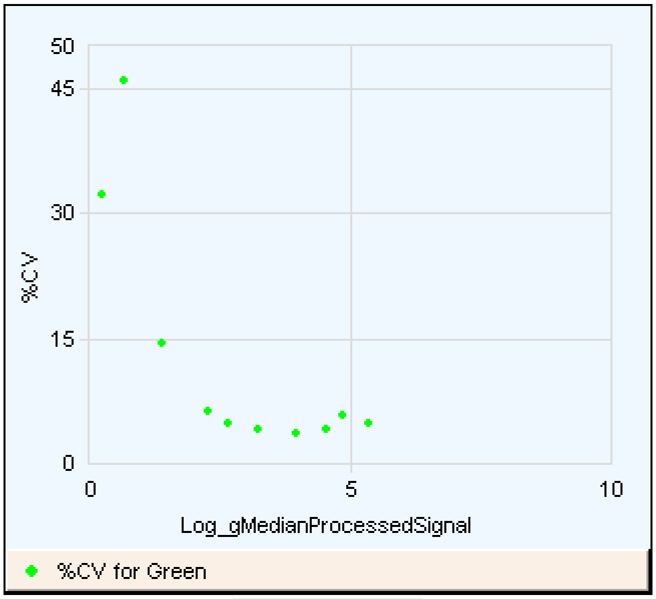

Median %CV:4.70

Agilent SpikeIns: Log(Signal) vs. Log(Relative concentration) Plot

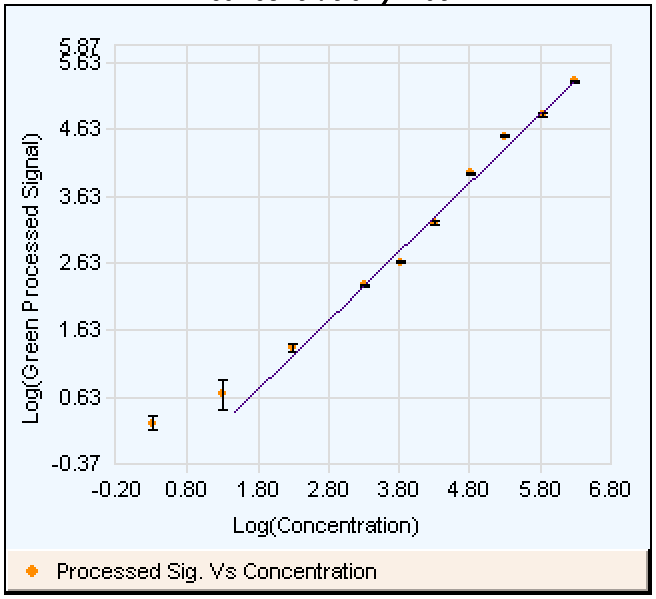

Agilent Spike-In Concentration-Response Statistics

Linear Range Statistics:

Low Signal	0.41
High Signal	5.64
Low Relative Concentration	1.47
High Relative Concentration	6.56
Slope	1.03
R^2 Value	0.99

Signal Detection Limit Statistics

Saturation Point	5.77
Low Threshold	0.15
Low Threshold Error	0.24
Spike-In Detection Limit	0.84

QC Report - Agilent Technologies : 1 Color Gene Expression

Date	Wednesday, September 21, 2011 - 10:49	Grid	035923_D_F_20110809
Image	UniversityPadova_253592310005_S01_H [1_2]	BG Method	No Background
Protocol	GE1-v5_95_Feb07 (Read Only)	Background Detrend	On(FeatNCRange, LoPass)
User Name	Administrator	Multiplicative Detrend	True
FE Version	9.5.1.1	Additive Error	2(Green)
		Saturation Value	580515 (g)

Spot Finding of the Four Corners of the Array

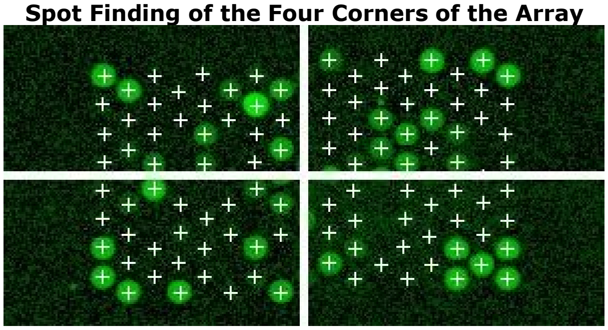

Grid Normal

	Feature Green	Local Background Green
Non Uniform	0	20
Population	28	260

Spatial Distribution of All Outliers on the Array

192 rows x 82 columns

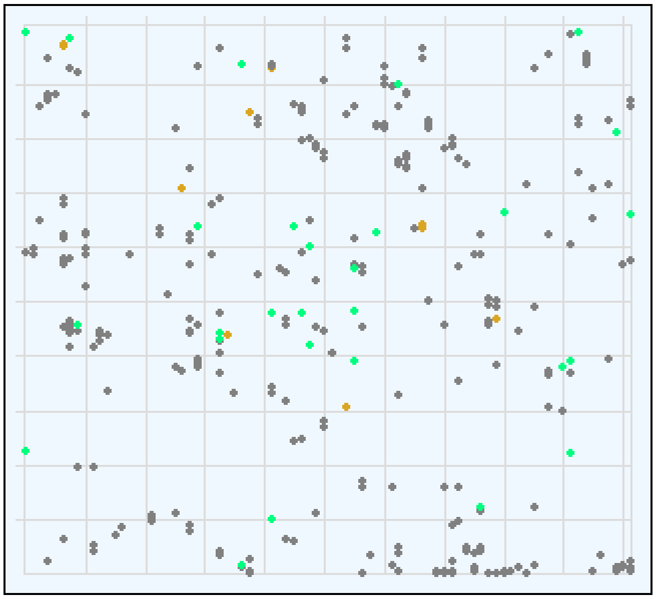

FeatureNonUnif (Green) = 0(0.00%)

GeneNonUnif (Green) = 0 (0.000 %)

BG NonUniform BG Population

Green FeaturePopulation Green Feature NonUniform

Negative Control Stats

	Green
Average Net Signals	16.07

Net Signal Statistics

Agilent SpikeIns:

	Green
# Saturated Features	0
99% of Sig. Distrib.	220787
50% of Sig. Distrib.	527
1% of Sig. Distrib.	16

Non-Control probes:

	Green
# Saturated Features	0
99% of Sig. Distrib.	22030
50% of Sig. Distrib.	25
1% of Sig. Distrib.	14

Histogram of Signals Plot

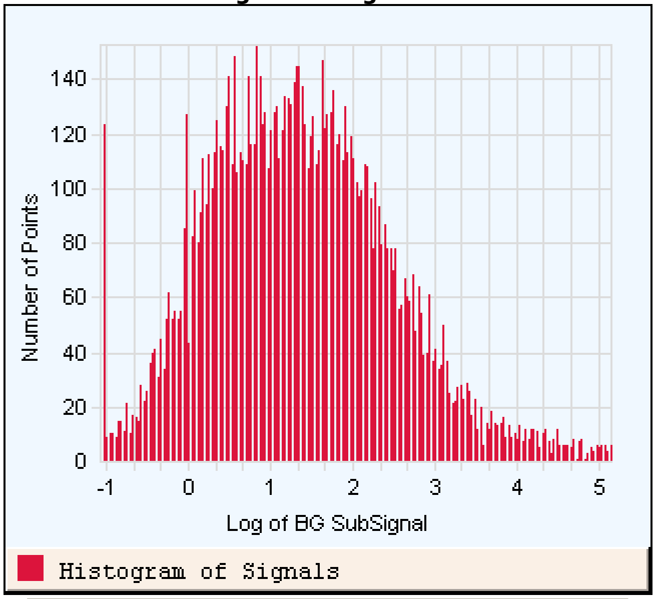

Features (NonCtrl) with BGSubSignal < 0: 2568 (Green)

StdDev Net Signals	1.52
Average BG Sub Signal	-1.02
StdDev BG Sub Signal	1.49

Local Bkg (inliers)

	Green
Number	13934
Avg	43.21
SD	1.84

Foreground Surface Fit

	Green
RMS_Fit	0.62
RMS_Resid	1.61
Avg_Fit	45.94

Multiplicative Surface Fit

	Green
RMS_Fit	0.10

Reproducibility: %CV for Replicated Probes

	Median %CV Signal (inliers)	
	Non-Control probes	Agilent SpikeIns
	Green	Green
BGSubSignal	-1.00	11.60
ProcessedSignal	-1.00	3.91

Agilent SpikeIns Signal Statistics

Probe Name	Log (Relative Conc.)	Median (Log Proc. Sig.)	% CV	StdDev
(+)E1A_r60_3	0.30	0.23	53.69	0.15
(+)E1A_r60_a104	1.30	0.52	40.68	0.17
(+)E1A_r60_a107	2.30	1.25	16.81	0.08
(+)E1A_r60_a135	3.30	2.15	5.21	0.02
(+)E1A_r60_a20	3.83	2.61	3.91	0.02
(+)E1A_r60_a22	4.30	3.02	3.41	0.01
(+)E1A_r60_a97	4.82	3.81	3.21	0.01
(+)E1A_r60_n11	5.30	4.36	2.98	0.01
(+)E1A_r60_n9	5.82	4.75	3.91	0.02
(+)E1A_r60_1	6.30	5.30	5.11	0.02

Agilent SpikeIns: %CV of Avg. Processed Signal Plot

Spatial Distribution of Median Signals for each Row

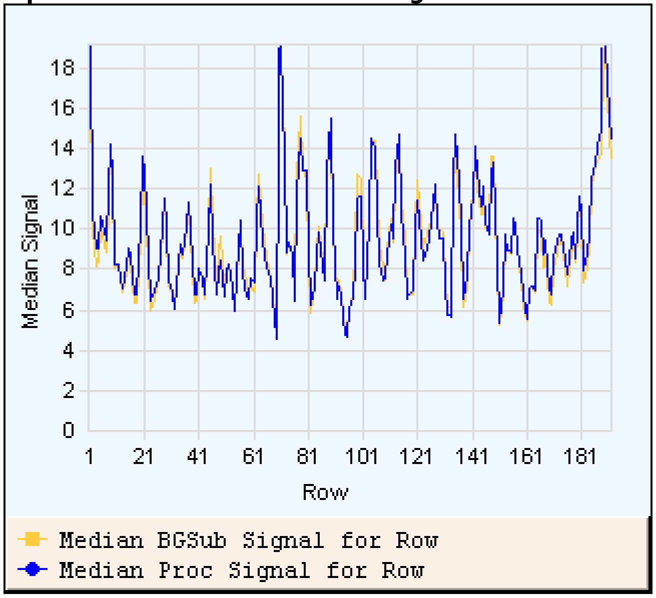

Spatial Distribution of Median Signals for each Column

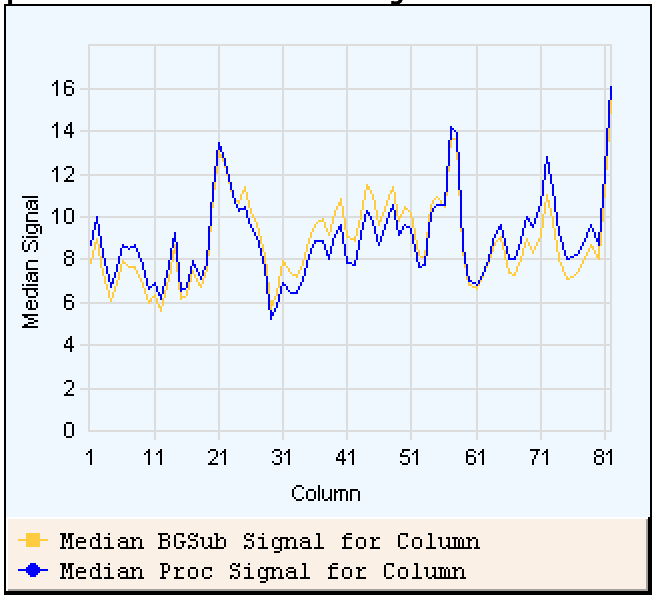

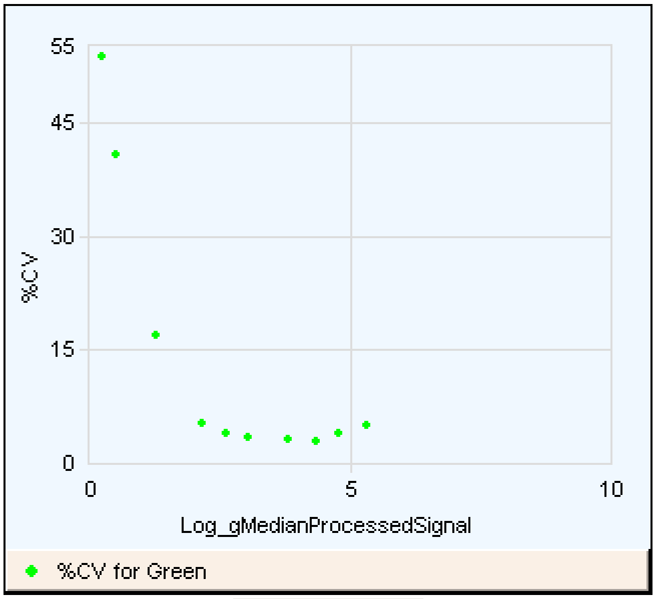

Median %CV:3.91

Agilent SpikeIns: Log(Signal) vs. Log(Relative concentration) Plot

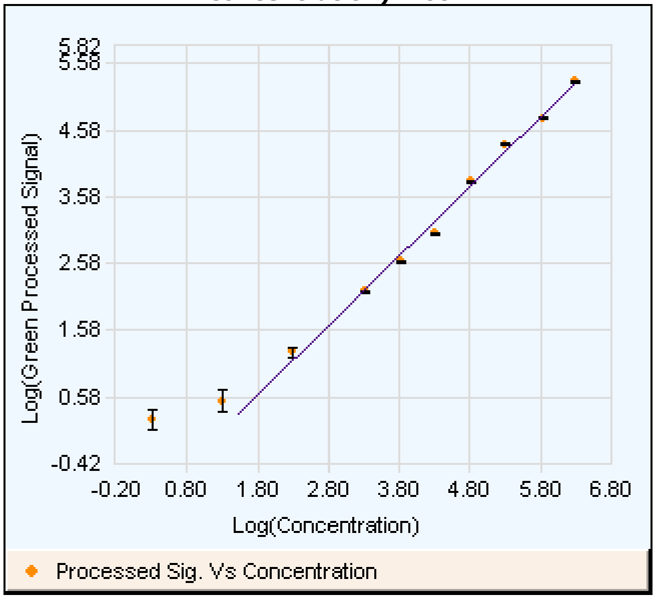

Agilent Spike-In Concentration-Response Statistics

Linear Range Statistics:

Low Signal	0.34
High Signal	5.67
Low Relative Concentration	1.53
High Relative Concentration	6.69
Slope	1.03
R^2 Value	0.99

Signal Detection Limit Statistics

Saturation Point	5.76
Low Threshold	0.09
Low Threshold Error	0.23
Spike-In Detection Limit	0.70

QC Report - Agilent Technologies : 1 Color Gene Expression

Date	Friday, September 23, 2011 - 11:02	Grid	035923_D_F_20110809
Image	UniversityPadova_253592310006_S01_H [1_3]	BG Method	No Background
Protocol	GE1-v5_95_Feb07 (Read Only)	Background Detrend	On(FeatNCRange, LoPass)
User Name	Administrator	Multiplicative Detrend	True
FE Version	9.5.1.1	Additive Error	2(Green)
		Saturation Value	588741 (g)

Spot Finding of the Four Corners of the Array

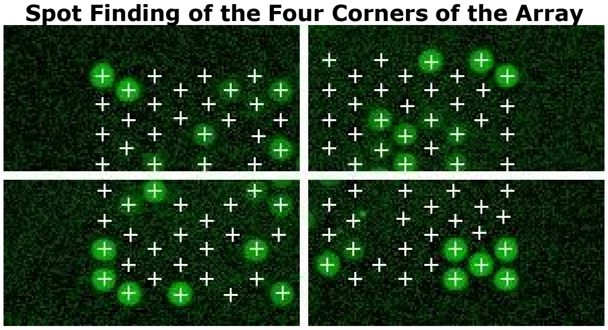

Grid Normal

	Feature	Local Background
	Green	Green
Non Uniform	1	14
Population	15	135

Spatial Distribution of All Outliers on the Array
192 rows x 82 columns

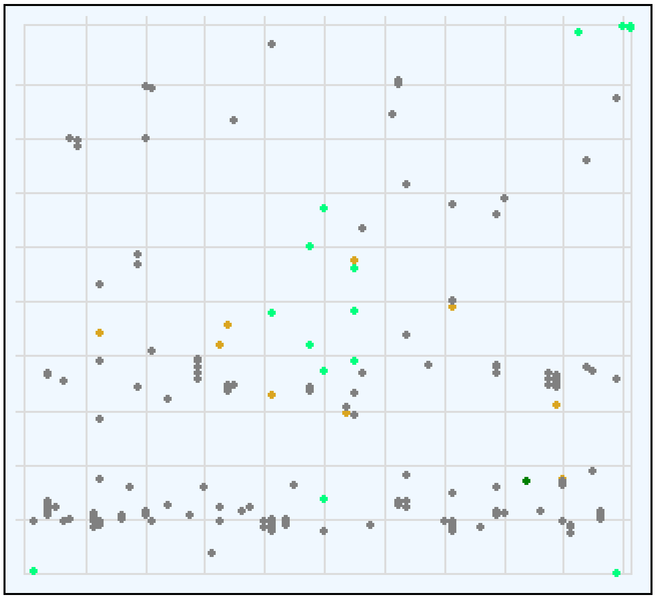

FeatureNonUnif (Green) = 1(0.01%)

GeneNonUnif (Green) = 1 (0.007 %)

BG NonUniform BG Population
Green FeaturePopulation Green Feature NonUniform

Negative Control Stats

	Green
Average Net Signals	13.92

Net Signal Statistics

Agilent SpikeIns:

	Green
# Saturated Features	0
99% of Sig. Distrib.	173126
50% of Sig. Distrib.	375
1% of Sig. Distrib.	11

Non-Control probes:

	Green
# Saturated Features	0
99% of Sig. Distrib.	13138
50% of Sig. Distrib.	20
1% of Sig. Distrib.	10

Histogram of Signals Plot

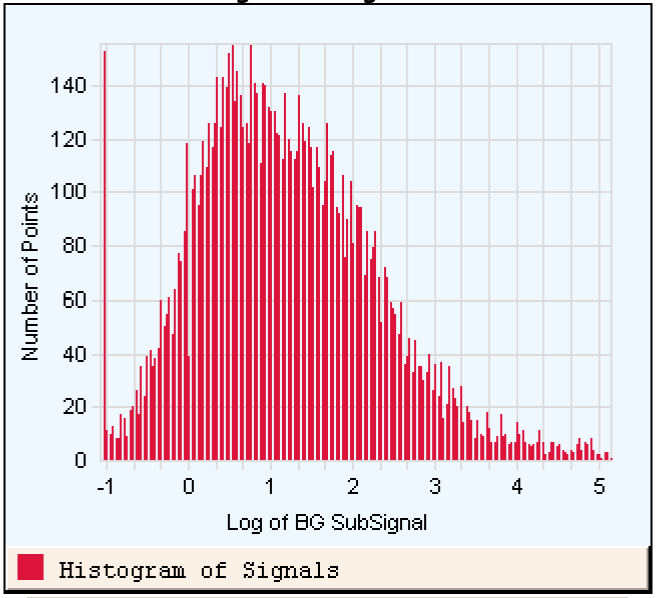

Features (NonCtrl) with BGSubSignal < 0: 3097 (Green)

StdDev Net Signals	2.22
Average BG Sub Signal	-0.80
StdDev BG Sub Signal	1.61

Local Bkg (inliers)

	Green
Number	14063
Avg	44.54
SD	2.45

Foreground Surface Fit

	Green
RMS_Fit	1.43
RMS_Resid	1.69
Avg_Fit	43.09

Multiplicative Surface Fit

	Green
RMS_Fit	0.06

Reproducibility: %CV for Replicated Probes

	Median %CV Signal (inliers)	
	Non-Control probes	Agilent SpikeIns
	Green	Green
BGSubSignal	-1.00	7.45
ProcessedSignal	-1.00	4.10

Agilent SpikeIns Signal Statistics

Probe Name	Log (Relative Conc.)	Median (Log Proc. Sig.)	% CV	StdDev
(+)E1A_r60_3	0.30	0.25	14.03	0.05
(+)E1A_r60_a104	1.30	0.57	34.21	0.16
(+)E1A_r60_a107	2.30	1.23	17.61	0.08
(+)E1A_r60_a135	3.30	2.08	4.88	0.02
(+)E1A_r60_a20	3.83	2.44	4.87	0.02
(+)E1A_r60_a22	4.30	3.03	4.10	0.02
(+)E1A_r60_a97	4.82	3.80	2.95	0.01
(+)E1A_r60_n11	5.30	4.32	3.82	0.02
(+)E1A_r60_n9	5.82	4.70	4.68	0.02
(+)E1A_r60_1	6.30	5.23	3.30	0.01

Agilent SpikeIns: %CV of Avg. Processed Signal Plot

Spatial Distribution of Median Signals for each Row

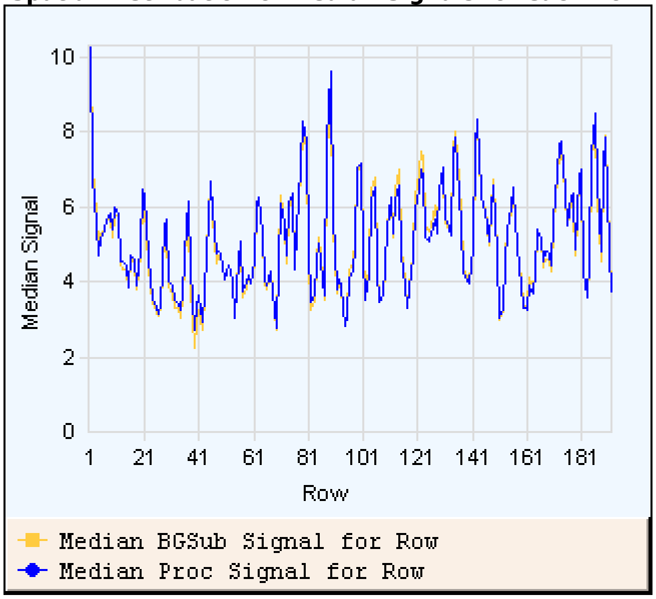

Spatial Distribution of Median Signals for each Column

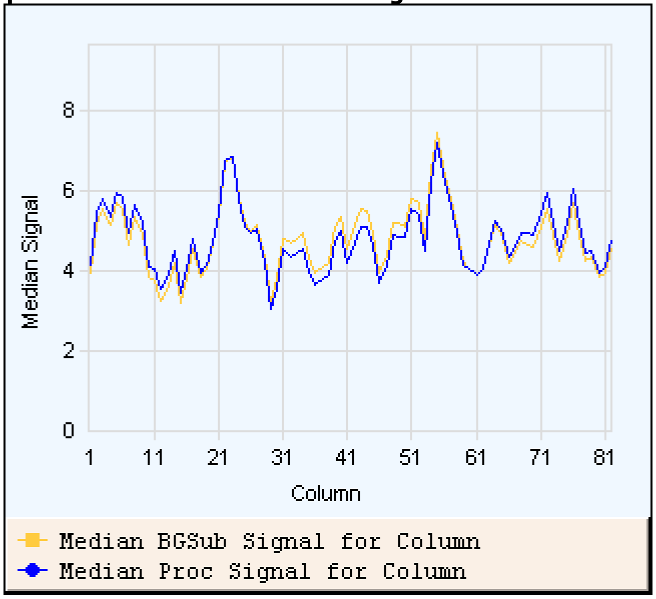

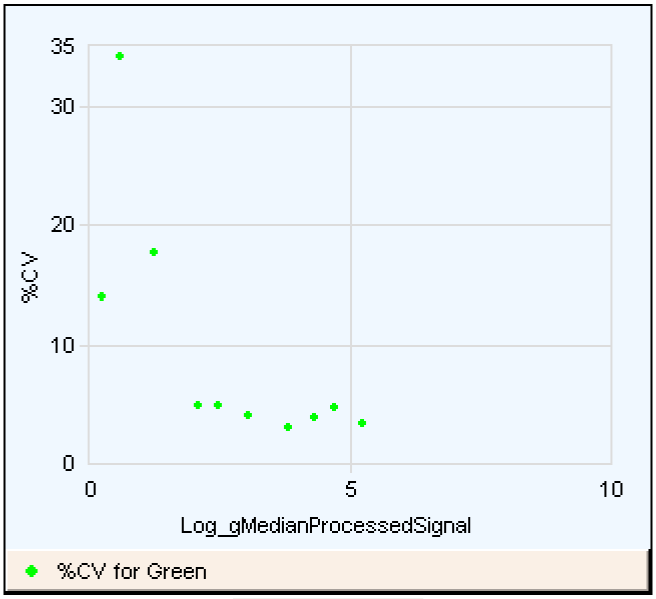

Median %CV:4.10

Agilent SpikeIns: Log(Signal) vs. Log(Relative concentration) Plot

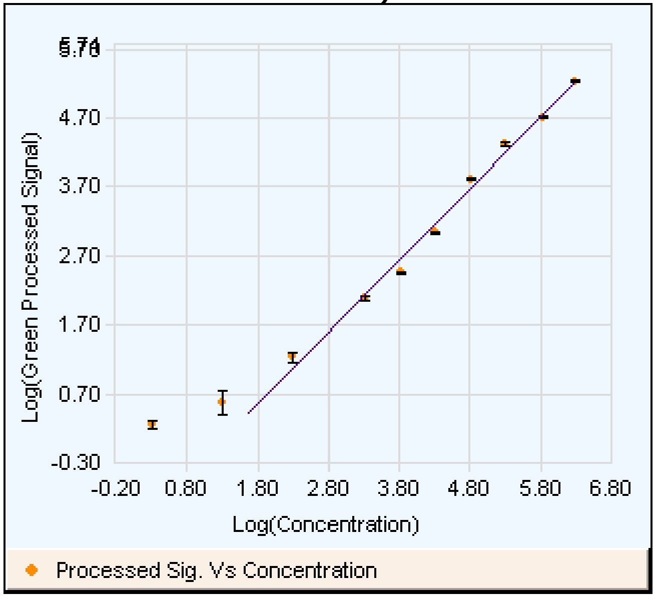

Agilent Spike-In Concentration-Response Statistics

Linear Range Statistics:

Low Signal	0.42
High Signal	5.67
Low Relative Concentration	1.66
High Relative Concentration	6.74
Slope	1.04
R^2 Value	0.99

Signal Detection Limit Statistics

Saturation Point	5.77
Low Threshold	0.16
Low Threshold Error	0.17
Spike-In Detection Limit	0.70

QC Report - Agilent Technologies : 1 Color Gene Expression

Date	Wednesday, September 21, 2011 - 10:49	Grid	035923_D_F_20110809
Image	UniversityPadova_253592310005_S01_H [1_3]	BG Method	No Background
Protocol	GE1-v5_95_Feb07 (Read Only)	Background Detrend	On(FeatNCRange, LoPass)
User Name	Administrator	Multiplicative Detrend	True
FE Version	9.5.1.1	Additive Error	2(Green)
		Saturation Value	581067 (g)

Spot Finding of the Four Corners of the Array

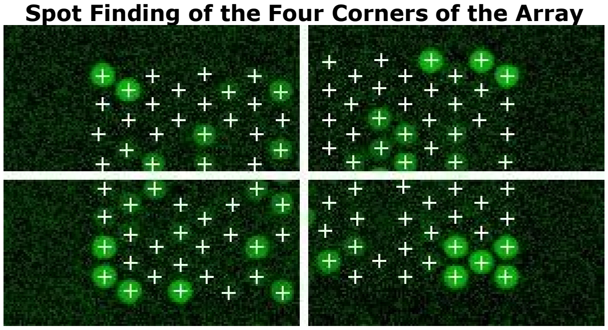

Grid Normal

	Feature Green	Local Background Green
Non Uniform	0	13
Population	21	177

Spatial Distribution of All Outliers on the Array

192 rows x 82 columns

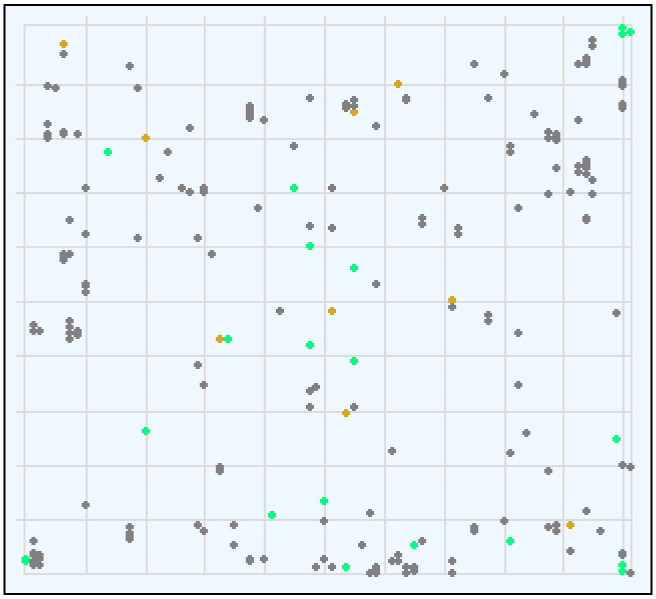

FeatureNonUnif (Green) = 0(0.00%)

GeneNonUnif (Green) = 0 (0.000 %)

Negative Control Stats

	Green
Average Net Signals	16.02

Net Signal Statistics

Agilent SpikeIns:

	Green
# Saturated Features	0
99% of Sig. Distrib.	227057
50% of Sig. Distrib.	587
1% of Sig. Distrib.	15

Non-Control probes:

	Green
# Saturated Features	0
99% of Sig. Distrib.	11296
50% of Sig. Distrib.	22
1% of Sig. Distrib.	14

Histogram of Signals Plot

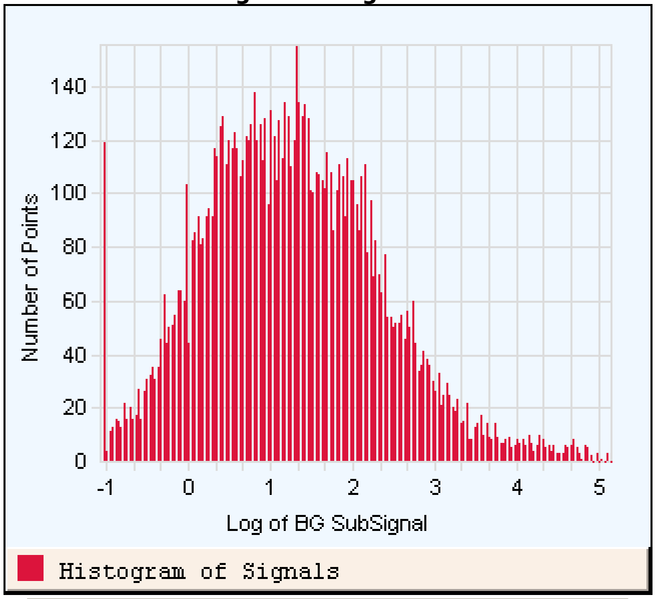

Features (NonCtrl) with BGSubSignal < 0: 3658 (Green)

StdDev Net Signals	1.94
Average BG Sub Signal	-1.74
StdDev BG Sub Signal	1.74

Local Bkg (inliers)

	Green
Number	14021
Avg	42.82
SD	1.97

Foreground Surface Fit

	Green
RMS_Fit	0.90
RMS_Resid	2.05
Avg_Fit	46.65

Multiplicative Surface Fit

	Green
RMS_Fit	0.11

Reproducibility: %CV for Replicated Probes

	Median %CV Signal (inliers)	
	Non-Control probes	Agilent SpikeIns
	Green	Green
BGSubSignal	-1.00	12.28
ProcessedSignal	-1.00	3.42

Agilent SpikeIns Signal Statistics

Probe Name	Log (Relative Conc.)	Median (Log Proc. Sig.)	% CV	StdDev
(+)E1A_r60_3	0.30	0.34	10.97	0.05
(+)E1A_r60_a104	1.30	0.48	40.62	0.17
(+)E1A_r60_a107	2.30	1.33	14.00	0.06
(+)E1A_r60_a135	3.30	2.19	4.88	0.02
(+)E1A_r60_a20	3.83	2.64	3.26	0.01
(+)E1A_r60_a22	4.30	3.08	3.32	0.01
(+)E1A_r60_a97	4.82	3.83	4.17	0.02
(+)E1A_r60_n11	5.30	4.39	3.39	0.01
(+)E1A_r60_n9	5.82	4.77	3.42	0.02
(+)E1A_r60_1	6.30	5.32	4.43	0.02

Agilent SpikeIns: %CV of Avg. Processed Signal Plot

Spatial Distribution of Median Signals for each Row

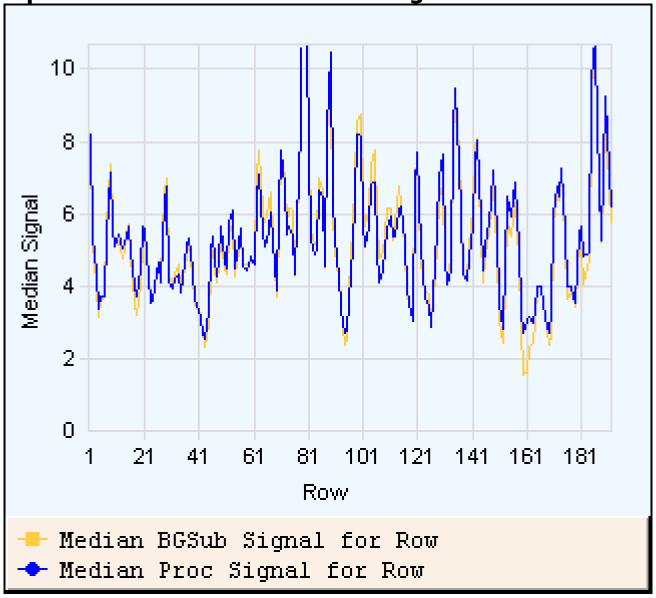

Spatial Distribution of Median Signals for each Column

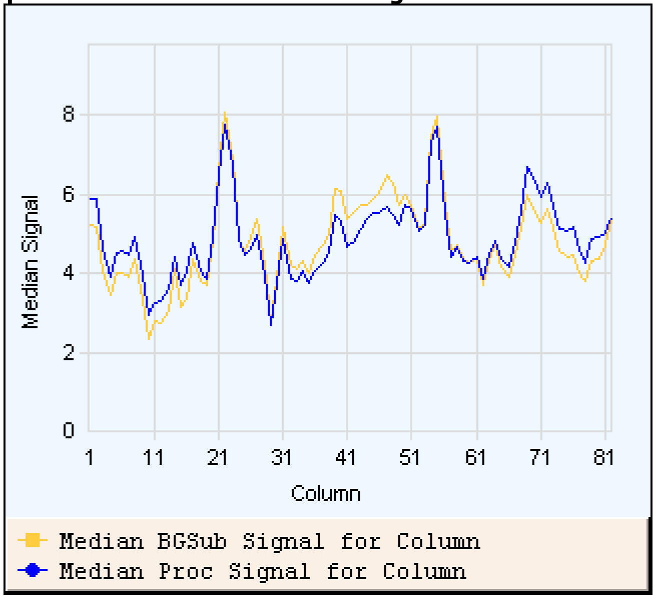

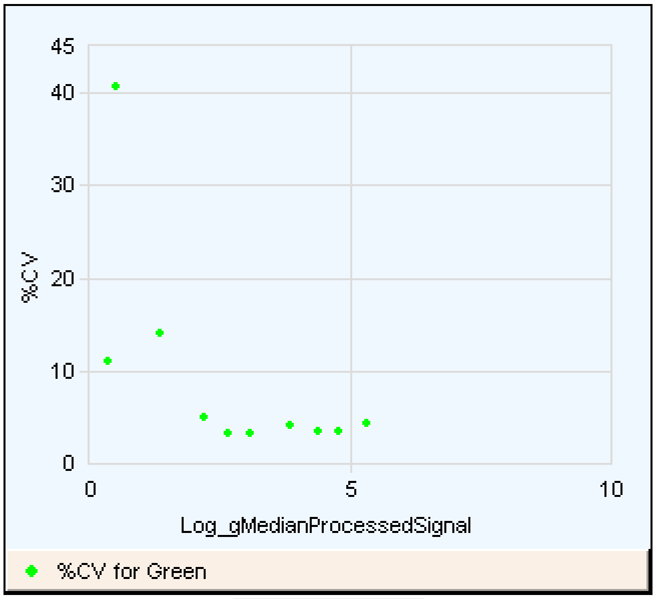

Median %CV:3.42

Agilent SpikeIns: Log(Signal) vs. Log(Relative concentration) Plot

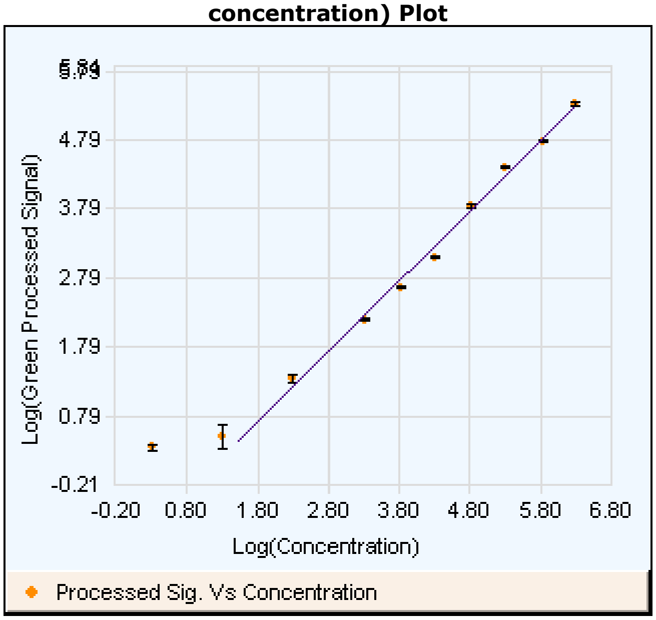

Agilent Spike-In Concentration-Response Statistics

Linear Range Statistics:

Low Signal	0.43
High Signal	5.66
Low Relative Concentration	1.54
High Relative Concentration	6.66
Slope	1.02
R^2 Value	0.99

Signal Detection Limit Statistics

Saturation Point	5.76
Low Threshold	0.15
Low Threshold Error	0.17
Spike-In Detection Limit	0.70

QC Report - Agilent Technologies : 1 Color Gene Expression

Date	Friday, September 23, 2011 - 11:03	Grid	035923_D_F_20110809
Image	UniversityPadova_253592310006_S01_H [1_4]	BG Method	No Background
Protocol	GE1-v5_95_Feb07 (Read Only)	Background Detrend	On(FeatNCRange, LoPass)
User Name	Administrator	Multiplicative Detrend	True
FE Version	9.5.1.1	Additive Error	2(Green)
		Saturation Value	589576 (g)

Spot Finding of the Four Corners of the Array

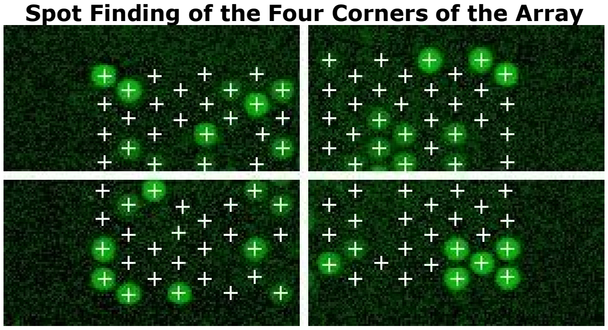

Grid Normal

	Feature Green	Local Background Green
Non Uniform	6	39
Population	15	146

Spatial Distribution of All Outliers on the Array

192 rows x 82 columns

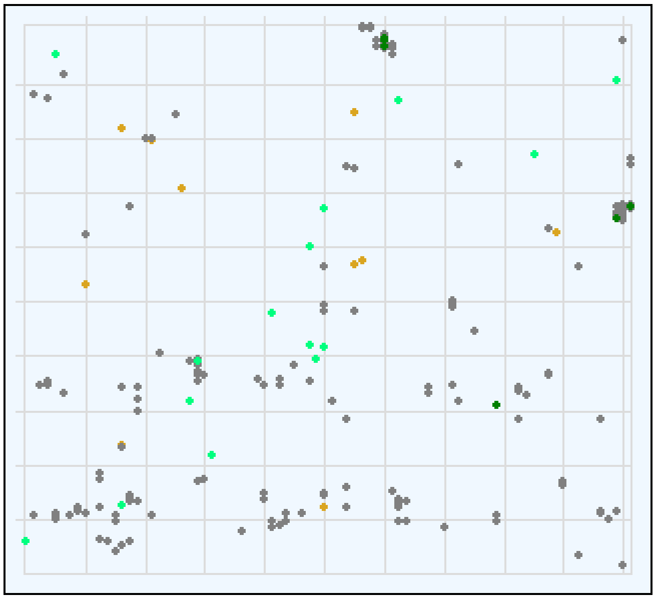

FeatureNonUnif (Green) = 6(0.04%)

GeneNonUnif (Green) = 6 (0.044 %)

BG NonUniform, BG Population, Green FeaturePopulation, Green Feature NonUniform

Negative Control Stats

	Green
Average Net Signals	12.99

Net Signal Statistics

Agilent SpikeIns:

	Green
# Saturated Features	0
99% of Sig. Distrib.	234760
50% of Sig. Distrib.	559
1% of Sig. Distrib.	12

Non-Control probes:

	Green
# Saturated Features	0
99% of Sig. Distrib.	14253
50% of Sig. Distrib.	20
1% of Sig. Distrib.	10

Histogram of Signals Plot

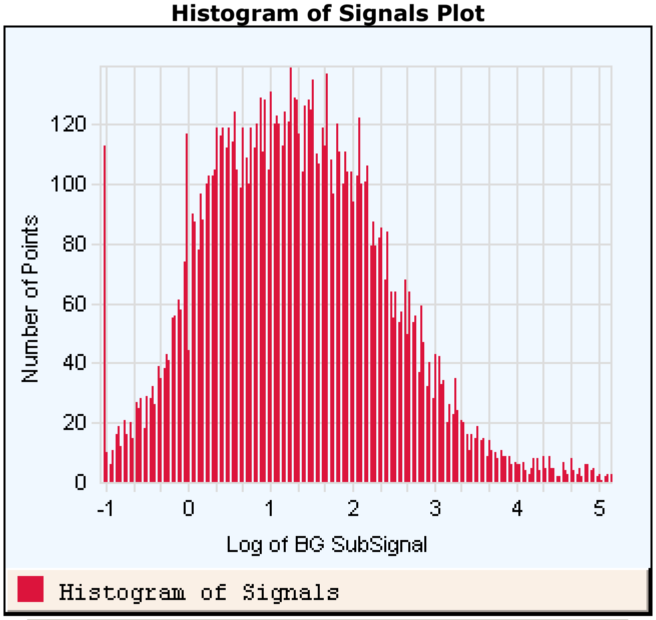

Features (NonCtrl) with BGSubSignal < 0: 3340 (Green)

StdDev Net Signals	1.70
Average BG Sub Signal	-1.09
StdDev BG Sub Signal	1.59

Local Bkg (inliers)

	Green
Number	14051
Avg	44.05
SD	2.38

Foreground Surface Fit

	Green
RMS_Fit	0.77
RMS_Resid	1.63
Avg_Fit	42.48

Multiplicative Surface Fit

	Green
RMS_Fit	0.06

Reproducibility: %CV for Replicated Probes

	Median %CV Signal (inliers)	
	Non-Control probes	Agilent SpikeIns
	Green	Green
BGSubSignal	-1.00	6.45
ProcessedSignal	-1.00	4.96

Agilent SpikeIns Signal Statistics

Probe Name	Log (Relative Conc.)	Median (Log Proc. Sig.)	% CV	StdDev
(+)E1A_r60_3	0.30	0.25	30.44	0.11
(+)E1A_r60_a104	1.30	0.62	37.74	0.18
(+)E1A_r60_a107	2.30	1.42	12.63	0.05
(+)E1A_r60_a135	3.30	2.28	4.96	0.02
(+)E1A_r60_a20	3.83	2.64	5.55	0.02
(+)E1A_r60_a22	4.30	3.21	6.80	0.03
(+)E1A_r60_a97	4.82	3.95	4.25	0.02
(+)E1A_r60_n11	5.30	4.48	4.14	0.02
(+)E1A_r60_n9	5.82	4.88	4.95	0.02
(+)E1A_r60_1	6.30	5.34	5.17	0.02

Agilent SpikeIns: %CV of Avg. Processed Signal Plot

Spatial Distribution of Median Signals for each Row

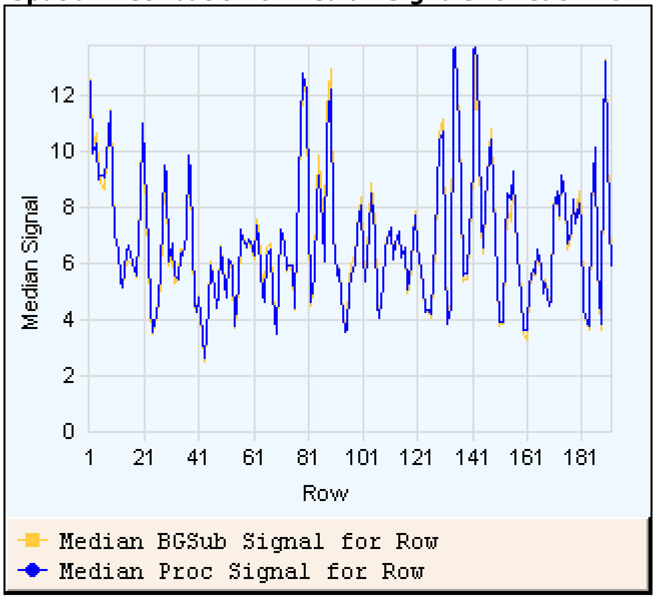

Spatial Distribution of Median Signals for each Column

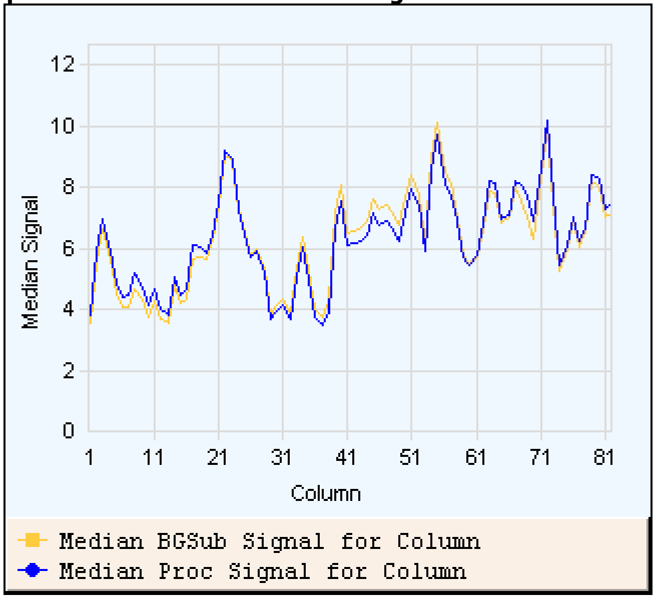

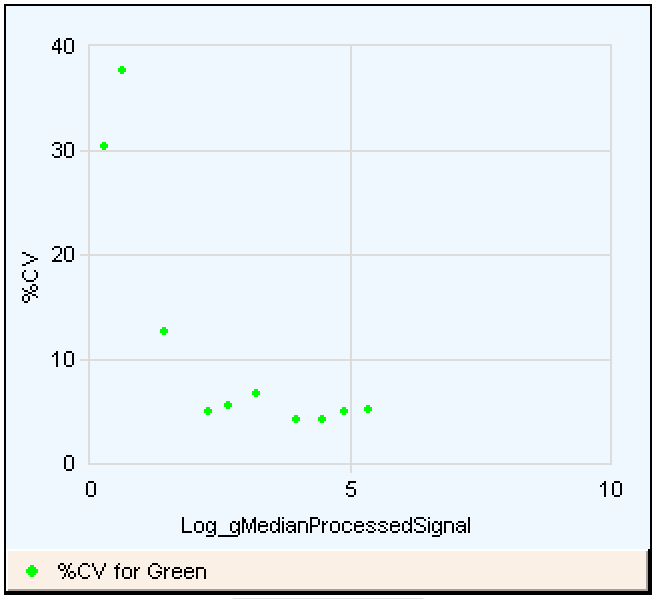

Median %CV:4.96

Agilent SpikeIns: Log(Signal) vs. Log(Relative concentration) Plot

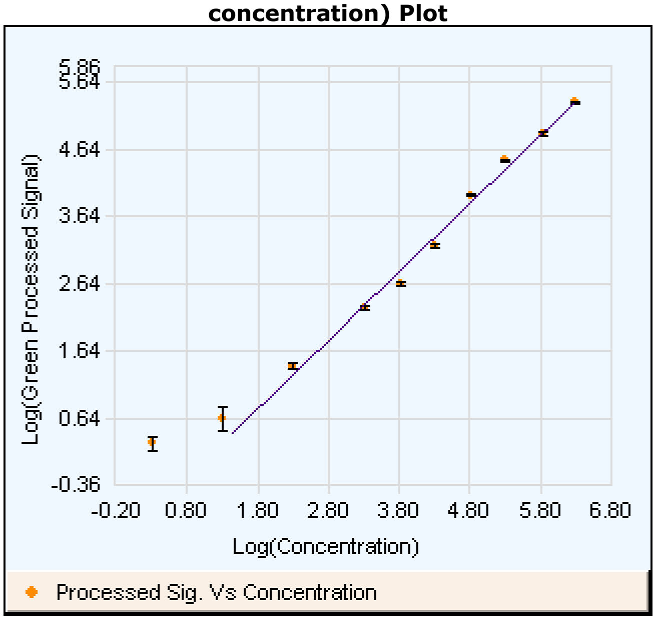

Agilent Spike-In Concentration-Response Statistics

Linear Range Statistics:

Low Signal	0.42
High Signal	5.65
Low Relative Concentration	1.45
High Relative Concentration	6.58
Slope	1.02
R^2 Value	0.99

Signal Detection Limit Statistics

Saturation Point	5.77
Low Threshold	0.14
Low Threshold Error	0.22
Spike-In Detection Limit	0.72

QC Report - Agilent Technologies : 1 Color Gene Expression

Date	Wednesday, September 21, 2011 - 10:49	Grid	035923_D_F_20110809
Image	UniversityPadova_253592310005_S01_H [1_4]	BG Method	No Background
Protocol	GE1-v5_95_Feb07 (Read Only)	Background Detrend	On(FeatNCRange, LoPass)
User Name	Administrator	Multiplicative Detrend	True
FE Version	9.5.1.1	Additive Error	2(Green)
		Saturation Value	581811 (g)

Spot Finding of the Four Corners of the Array

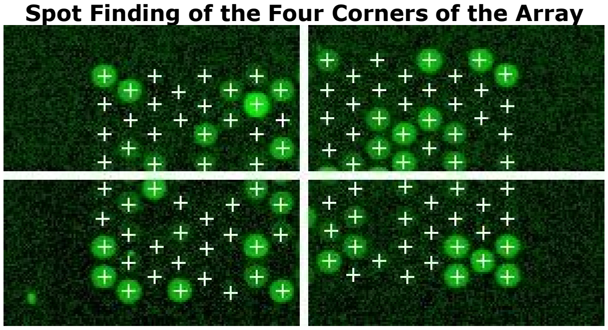

Grid Normal

	Feature Green	Local Background Green
Non Uniform	6	47
Population	17	168

Spatial Distribution of All Outliers on the Array

192 rows x 82 columns

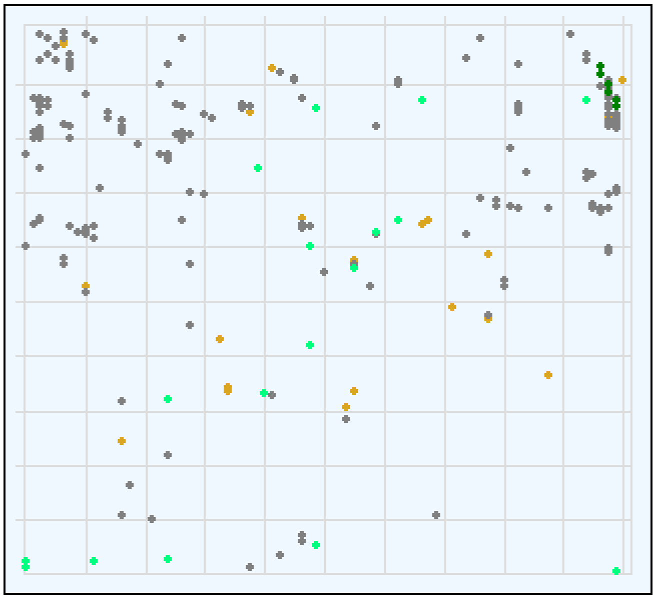

FeatureNonUnif (Green) = 6(0.04%)

GeneNonUnif (Green) = 6 (0.044 %)

BG NonUniform · BG Population · Green FeaturePopulation · Green Feature NonUniform

Negative Control Stats

	Green
Average Net Signals	18.12

Net Signal Statistics

Agilent SpikeIns:

	Green
# Saturated Features	0
99% of Sig. Distrib.	213010
50% of Sig. Distrib.	498
1% of Sig. Distrib.	18

Non-Control probes:

	Green
# Saturated Features	1
99% of Sig. Distrib.	38102
50% of Sig. Distrib.	39
1% of Sig. Distrib.	15

Histogram of Signals Plot

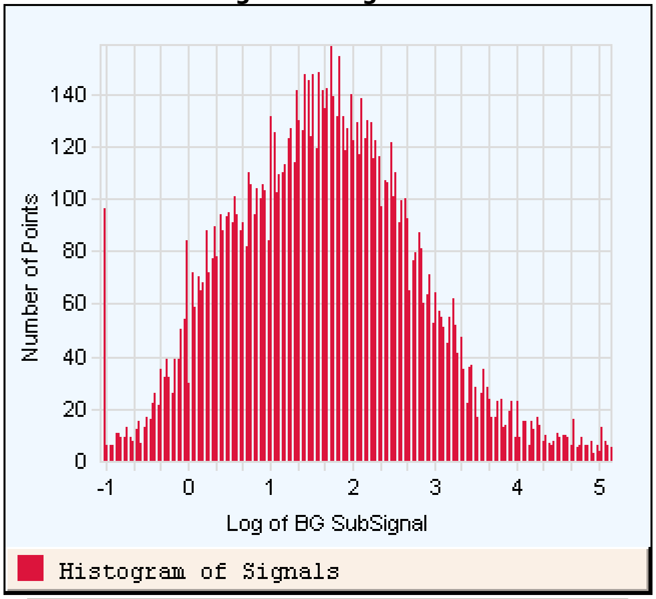

Features (NonCtrl) with BGSubSignal < 0: 2364 (Green)

StdDev Net Signals	2.30
Average BG Sub Signal	-1.51
StdDev BG Sub Signal	1.93

Local Bkg (inliers)

	Green
Number	14018
Avg	41.31
SD	2.22

Foreground Surface Fit

	Green
RMS_Fit	0.90
RMS_Resid	2.24
Avg_Fit	48.47

Multiplicative Surface Fit

	Green
RMS_Fit	0.11

Reproducibility: %CV for Replicated Probes

	Median %CV Signal (inliers)	
	Non-Control probes	Agilent SpikeIns
	Green	Green
BGSubSignal	-1.00	12.39
ProcessedSignal	-1.00	3.94

Agilent SpikeIns Signal Statistics

Probe Name	Log (Relative Conc.)	Median (Log Proc. Sig.)	% CV	StdDev
(+)E1A_r60_3	0.30	0.38	38.38	0.14
(+)E1A_r60_a104	1.30	0.67	244.53	0.39
(+)E1A_r60_a107	2.30	1.19	21.66	0.10
(+)E1A_r60_a135	3.30	2.12	5.32	0.02
(+)E1A_r60_a20	3.83	2.55	6.16	0.03
(+)E1A_r60_a22	4.30	3.02	2.55	0.01
(+)E1A_r60_a97	4.82	3.78	3.03	0.01
(+)E1A_r60_n11	5.30	4.31	3.63	0.02
(+)E1A_r60_n9	5.82	4.71	3.94	0.02
(+)E1A_r60_1	6.30	5.27	4.34	0.02

Agilent SpikeIns: %CV of Avg. Processed Signal Plot

Spatial Distribution of Median Signals for each Row

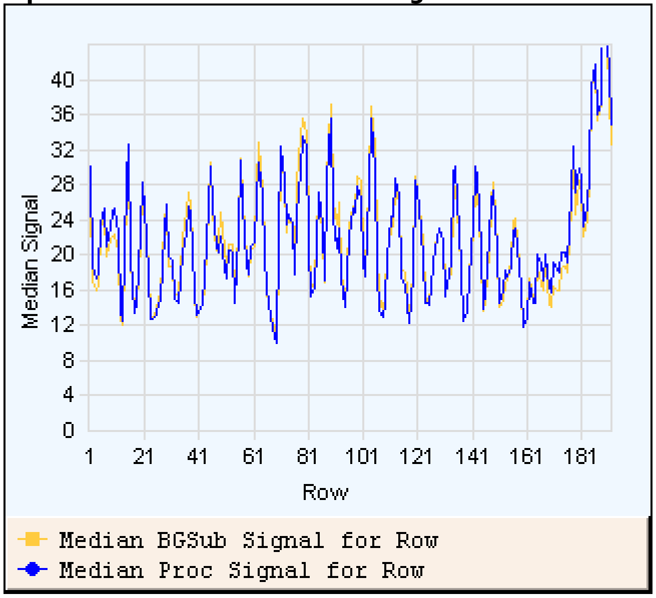

Spatial Distribution of Median Signals for each Column

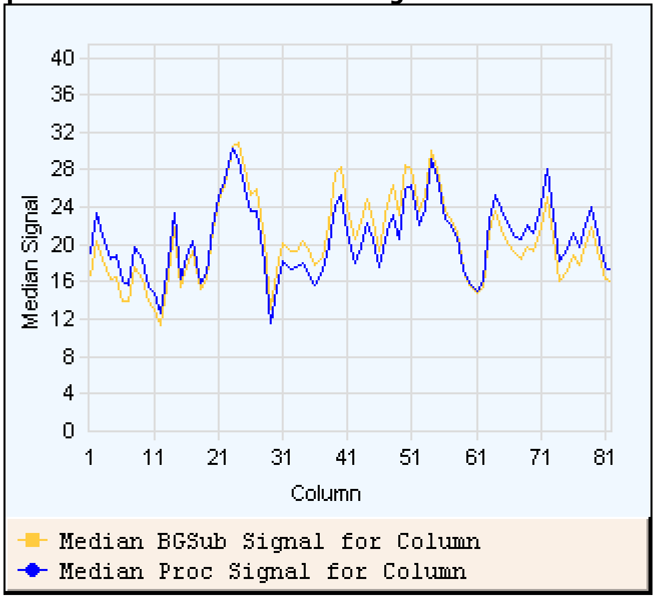

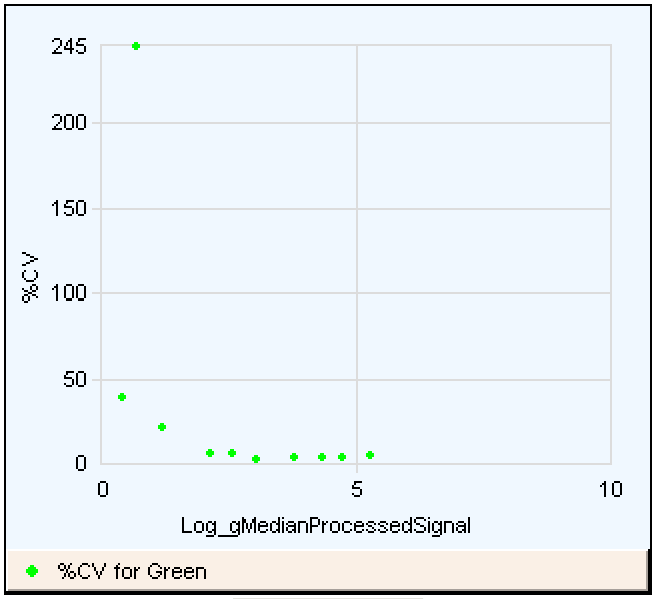

Median %CV:3.94

Agilent SpikeIns: Log(Signal) vs. Log(Relative concentration) Plot

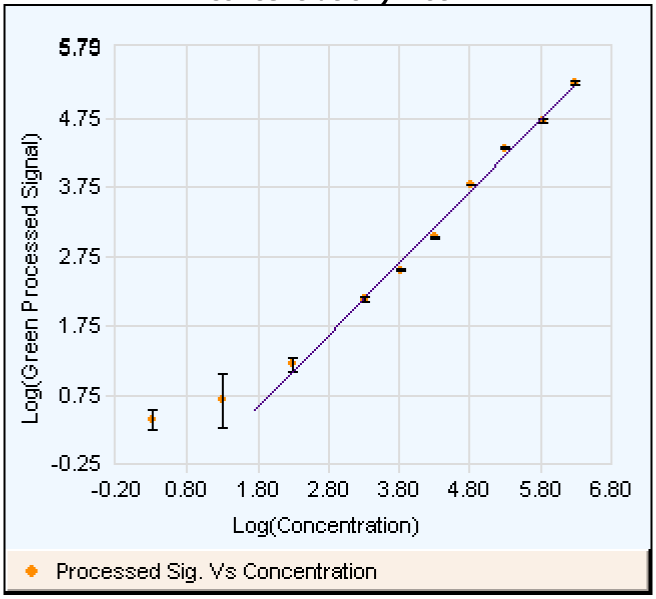

Agilent Spike-In Concentration-Response Statistics

Linear Range Statistics:

Low Signal	0.53
High Signal	5.68
Low Relative Concentration	1.76
High Relative Concentration	6.72
Slope	1.04
R^2 Value	0.99

Signal Detection Limit Statistics

Saturation Point	5.76
Low Threshold	0.30
Low Threshold Error	0.43
Spike-In Detection Limit	1.53

QC Report - Agilent Technologies : 1 Color Gene Expression

Date	Wednesday, September 21, 2011 - 10:49	Grid	035923_D_F_20110809
Image	UniversityPadova_253592310005_S01_H [2_1]	BG Method	No Background
Protocol	GE1-v5_95_Feb07 (Read Only)	Background Detrend	On(FeatNCRange, LoPass)
User Name	Administrator	Multiplicative Detrend	True
FE Version	9.5.1.1	Additive Error	2(Green)
		Saturation Value	582344 (g)

Spot Finding of the Four Corners of the Array

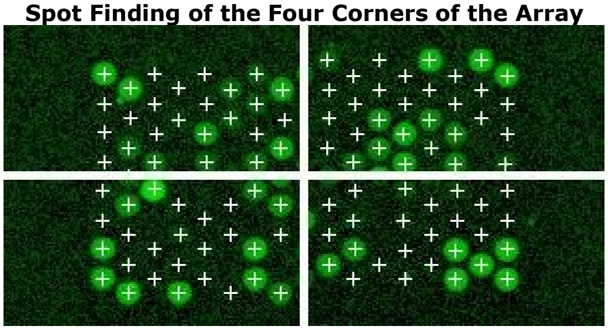

Grid Normal

	Feature	Local Background
	Green	Green
Non Uniform	7	19
Population	17	180

Spatial Distribution of All Outliers on the Array

192 rows x 82 columns

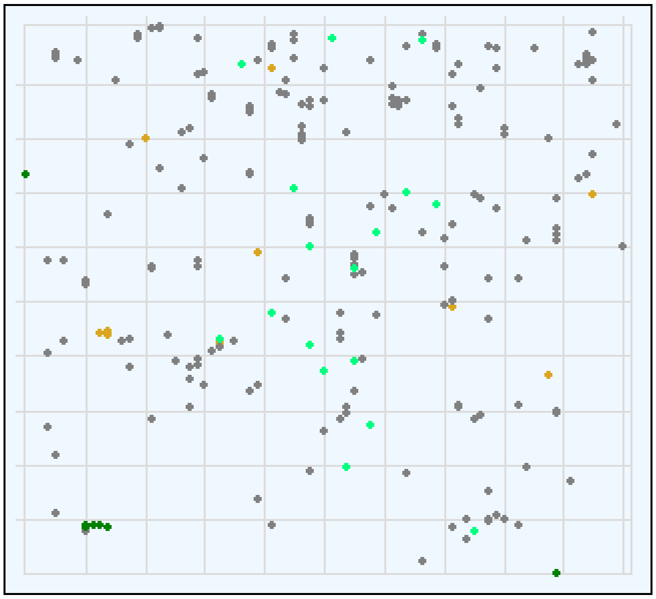

FeatureNonUnif (Green) = 7(0.05%)

GeneNonUnif (Green) = 7 (0.051 %)

BG NonUniform   BG Population

Green FeaturePopulation   Green Feature NonUniform

Negative Control Stats

	Green
Average Net Signals	14.15

Net Signal Statistics

Agilent SpikeIns:

	Green
# Saturated Features	0
99% of Sig. Distrib.	201884
50% of Sig. Distrib.	507
1% of Sig. Distrib.	14

Non-Control probes:

	Green
# Saturated Features	0
99% of Sig. Distrib.	29154
50% of Sig. Distrib.	35
1% of Sig. Distrib.	12

Histogram of Signals Plot

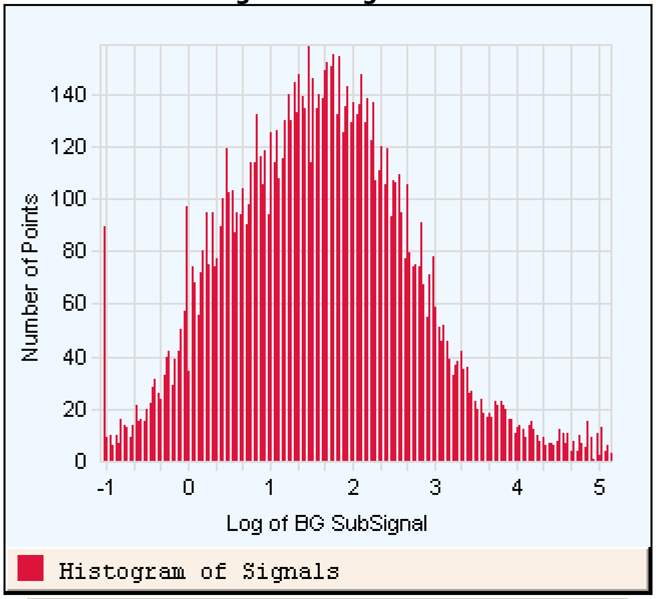

Features (NonCtrl) with BGSubSignal < 0: 2033 (Green)

StdDev Net Signals	1.09
Average BG Sub Signal	-1.20
StdDev BG Sub Signal	1.14

Local Bkg (inliers)

	Green
Number	14014
Avg	41.84
SD	1.82

Foreground Surface Fit

	Green
RMS_Fit	0.79
RMS_Resid	1.58
Avg_Fit	44.29

Multiplicative Surface Fit

	Green
RMS_Fit	0.11

Reproducibility: %CV for Replicated Probes

	Median %CV Signal (inliers)	
	Non-Control probes	Agilent SpikeIns
	Green	Green
BGSubSignal	-1.00	12.09
ProcessedSignal	-1.00	4.51

Agilent SpikeIns Signal Statistics

Probe Name	Log (Relative Conc.)	Median (Log Proc. Sig.)	% CV	StdDev
(+)E1A_r60_3	0.30	0.25	33.43	0.11
(+)E1A_r60_a104	1.30	0.56	40.89	0.18
(+)E1A_r60_a107	2.30	1.25	15.92	0.08
(+)E1A_r60_a135	3.30	2.20	7.28	0.03
(+)E1A_r60_a20	3.83	2.58	3.86	0.02
(+)E1A_r60_a22	4.30	3.07	2.87	0.01
(+)E1A_r60_a97	4.82	3.82	4.35	0.02
(+)E1A_r60_n11	5.30	4.37	4.51	0.02
(+)E1A_r60_n9	5.82	4.71	5.64	0.03
(+)E1A_r60_1	6.30	5.27	4.78	0.02

Agilent SpikeIns: %CV of Avg. Processed Signal Plot

Spatial Distribution of Median Signals for each Row

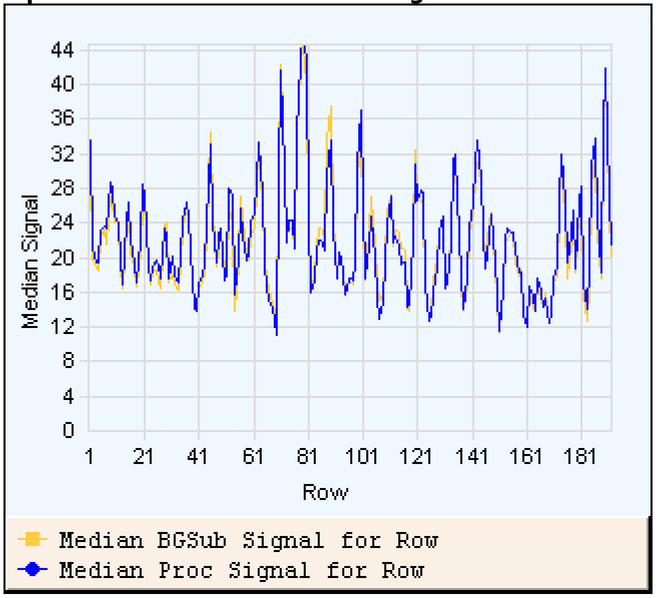

Spatial Distribution of Median Signals for each Column

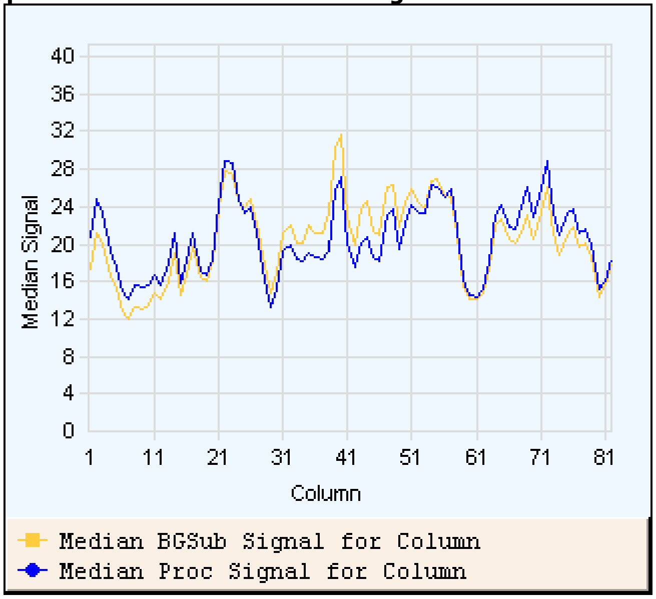

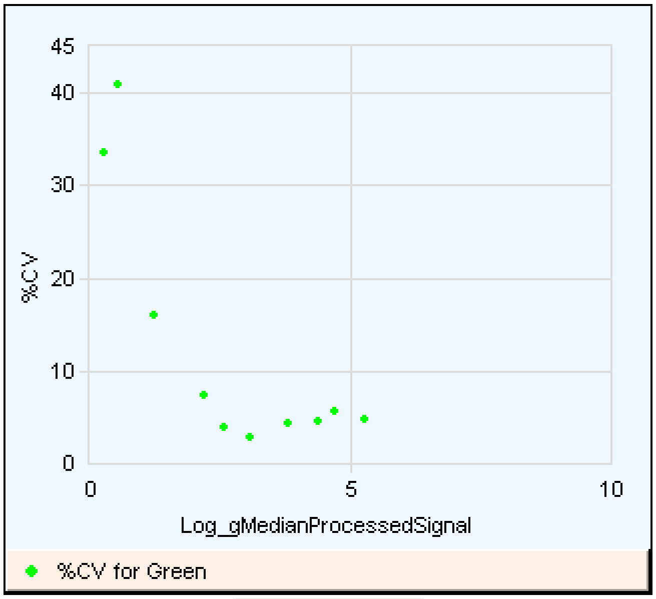

Median %CV:4.51

Agilent SpikeIns: Log(Signal) vs. Log(Relative concentration) Plot

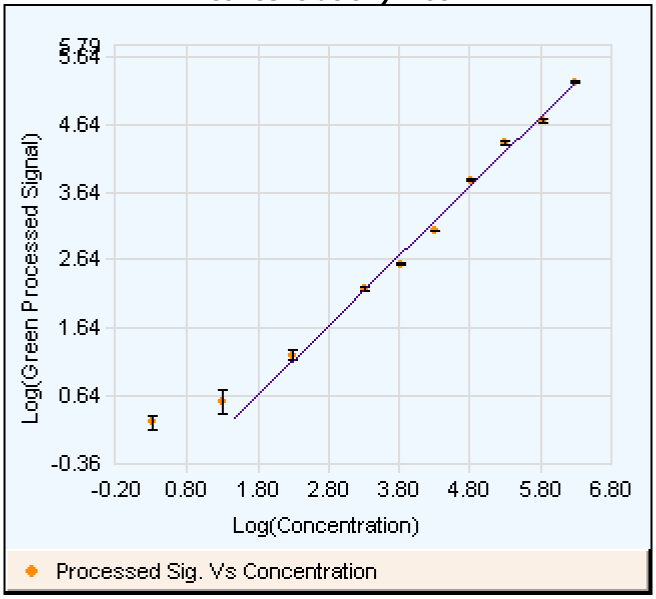

Agilent Spike-In Concentration-Response Statistics

Linear Range Statistics:

Low Signal	0.33
High Signal	5.68
Low Relative Concentration	1.48
High Relative Concentration	6.72
Slope	1.02
R^2 Value	0.99

Signal Detection Limit Statistics

Saturation Point	5.77
Low Threshold	0.10
Low Threshold Error	0.21
Spike-In Detection Limit	0.73

QC Report - Agilent Technologies : 1 Color Gene Expression

Date	Wednesday, September 21, 2011 - 10:49	Grid	035923_D_F_20110809
Image	UniversityPadova_253592310005_S01_H [2_2]	BG Method	No Background
Protocol	GE1-v5_95_Feb07 (Read Only)	Background Detrend	On(FeatNCRange, LoPass)
User Name	Administrator	Multiplicative Detrend	True
FE Version	9.5.1.1	Additive Error	2(Green)
		Saturation Value	583743 (g)

Spot Finding of the Four Corners of the Array

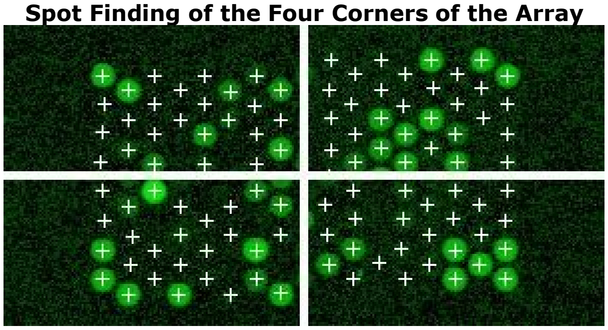

Grid Normal

	Feature Green	Local Background Green
Non Uniform	1	29
Population	10	224

Spatial Distribution of All Outliers on the Array

192 rows x 82 columns

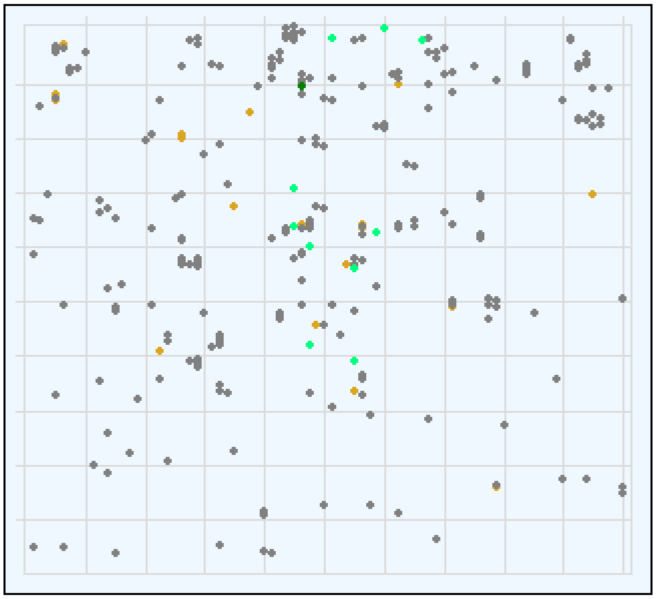

FeatureNonUnif (Green) = 1(0.01%)

GeneNonUnif (Green) = 1 (0.007 %)

BG NonUniform, BG Population, Green FeaturePopulation, Green Feature NonUniform

Negative Control Stats

	Green
Average Net Signals	15.89

Net Signal Statistics

Agilent SpikeIns:

	Green
# Saturated Features	0
99% of Sig. Distrib.	193358
50% of Sig. Distrib.	506
1% of Sig. Distrib.	15

Non-Control probes:

	Green
# Saturated Features	0
99% of Sig. Distrib.	42654
50% of Sig. Distrib.	27
1% of Sig. Distrib.	12

Histogram of Signals Plot

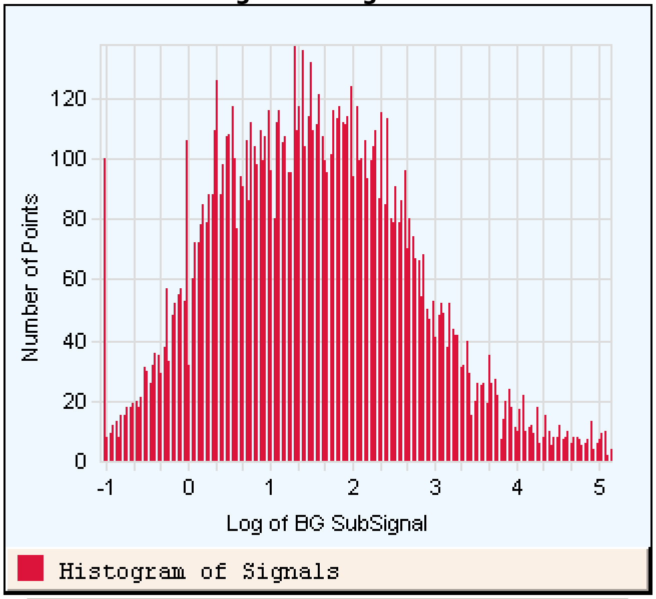

Features (NonCtrl) with BGSubSignal < 0: 3037 (Green)

StdDev Net Signals	2.12
Average BG Sub Signal	-1.08
StdDev BG Sub Signal	1.91

Local Bkg (inliers)

	Green
Number	13964
Avg	42.23
SD	1.93

Foreground Surface Fit

	Green
RMS_Fit	1.05
RMS_Resid	1.78
Avg_Fit	45.87

Multiplicative Surface Fit

	Green
RMS_Fit	0.11

Reproducibility: %CV for Replicated Probes

	Median %CV Signal (inliers)	
	Non-Control probes	Agilent SpikeIns
	Green	Green
BGSubSignal	-1.00	12.28
ProcessedSignal	-1.00	3.87

Agilent SpikeIns Signal Statistics

Probe Name	Log (Relative Conc.)	Median (Log Proc. Sig.)	% CV	StdDev
(+)E1A_r60_3	0.30	0.36	70.91	0.23
(+)E1A_r60_a104	1.30	0.46	52.41	0.22
(+)E1A_r60_a107	2.30	1.28	17.22	0.08
(+)E1A_r60_a135	3.30	2.18	4.87	0.02
(+)E1A_r60_a20	3.83	2.58	3.87	0.02
(+)E1A_r60_a22	4.30	3.08	2.99	0.01
(+)E1A_r60_a97	4.82	3.81	2.98	0.01
(+)E1A_r60_n11	5.30	4.35	3.51	0.02
(+)E1A_r60_n9	5.82	4.71	3.91	0.02
(+)E1A_r60_1	6.30	5.25	4.76	0.02

Agilent SpikeIns: %CV of Avg. Processed Signal Plot

Spatial Distribution of Median Signals for each Row

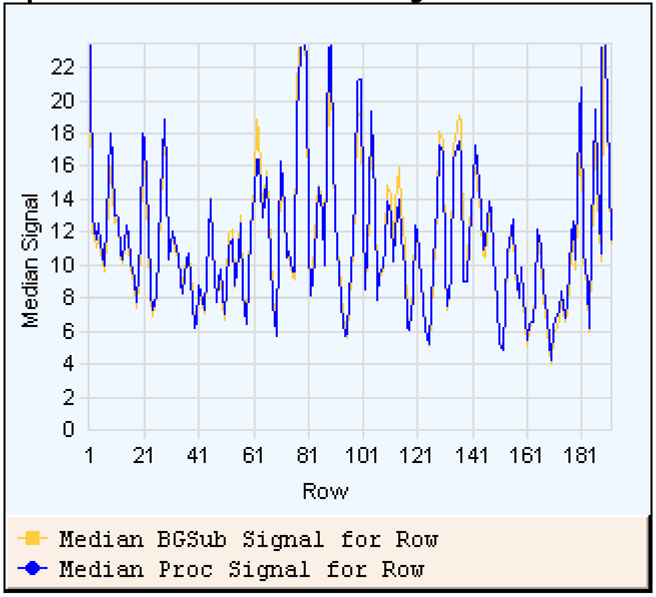

Spatial Distribution of Median Signals for each Column

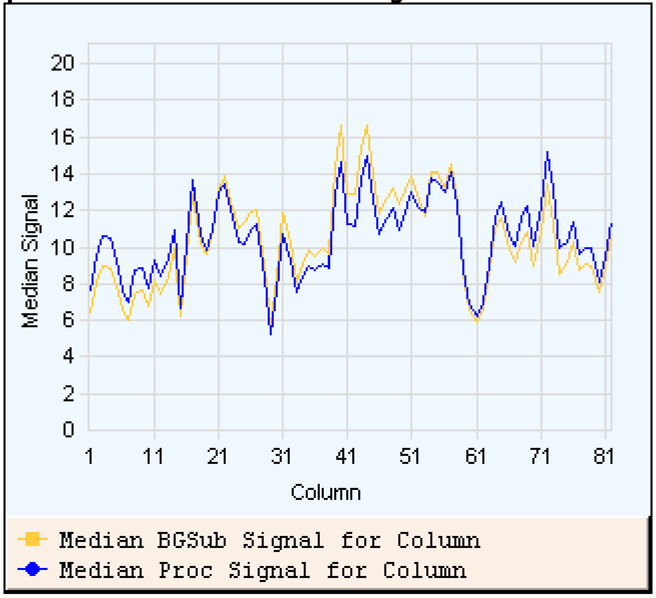

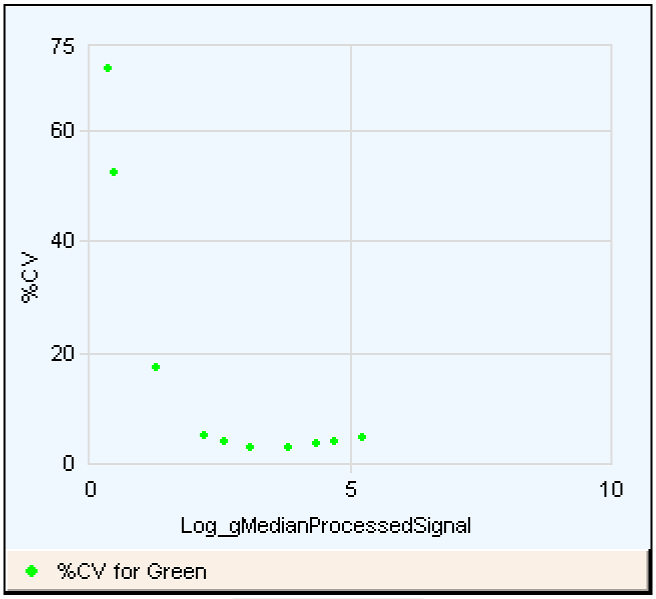

Median %CV:3.87

Agilent SpikeIns: Log(Signal) vs. Log(Relative concentration) Plot

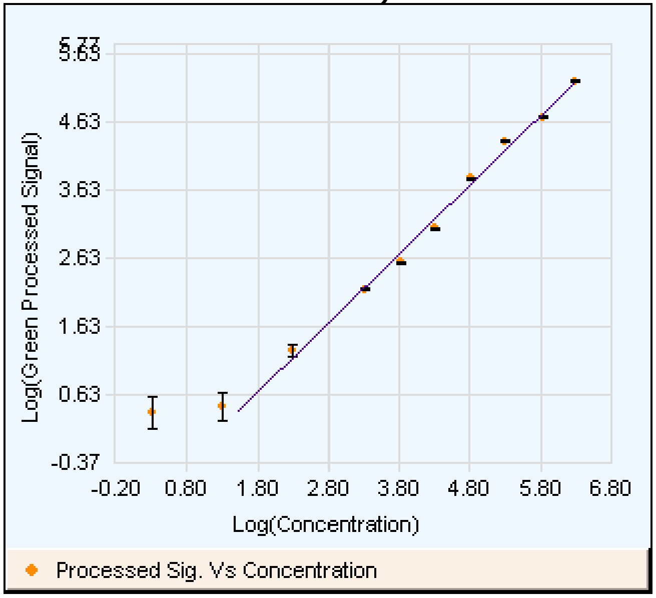

Agilent Spike-In Concentration-Response Statistics

Linear Range Statistics:

Low Signal	0.40
High Signal	5.66
Low Relative Concentration	1.54
High Relative Concentration	6.73
Slope	1.01
R^2 Value	0.99

Signal Detection Limit Statistics

Saturation Point	5.77
Low Threshold	0.14
Low Threshold Error	0.33
Spike-In Detection Limit	0.73

QC Report - Agilent Technologies : 1 Color Gene Expression

Date	Friday, September 02, 2011 - 10:55	Grid	035923_D_F_20110809
Image	UniversityPadova_253592310001_S01_H [1_2]	BG Method	No Background
Protocol	GE1-v5_95_Feb07 (Read Only)	Background Detrend	On(FeatNCRange, LoPass)
User Name	Administrator	Multiplicative Detrend	True
FE Version	9.5.1.1	Additive Error	1(Green)
		Saturation Value	597285 (g)

Spot Finding of the Four Corners of the Array

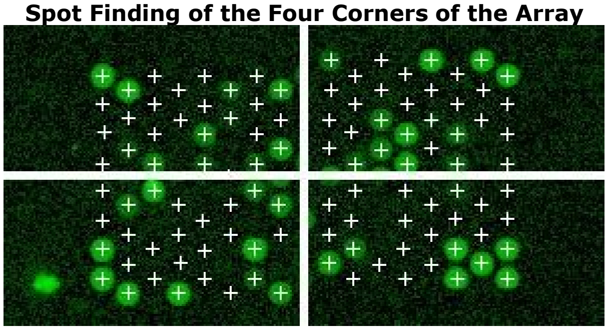

Grid Normal

	Feature	Local Background
	Green	Green
Non Uniform	1	14
Population	9	226

Spatial Distribution of All Outliers on the Array

192 rows x 82 columns

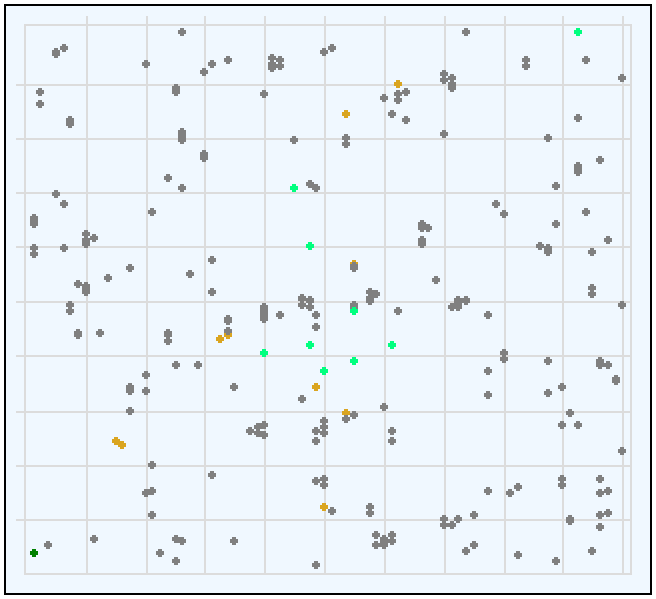

FeatureNonUnif (Green) = 1(0.01%)

GeneNonUnif (Green) = 1 (0.007 %)

BG NonUniform · BG Population · Green FeaturePopulation · Green Feature NonUniform

Negative Control Stats

	Green
Average Net Signals	11.21

Net Signal Statistics

Agilent SpikeIns:

	Green
# Saturated Features	0
99% of Sig. Distrib.	218286
50% of Sig. Distrib.	555
1% of Sig. Distrib.	11

Non-Control probes:

	Green
# Saturated Features	0
99% of Sig. Distrib.	22160
50% of Sig. Distrib.	18
1% of Sig. Distrib.	9

Histogram of Signals Plot

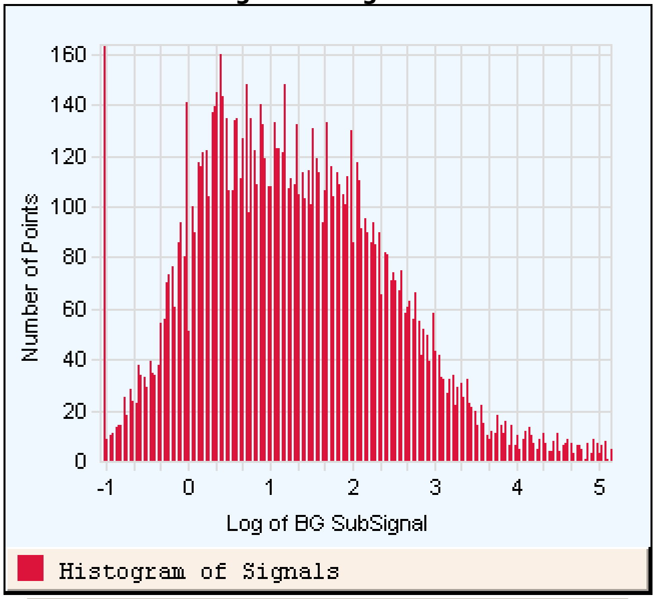

Features (NonCtrl) with BGSubSignal < 0: 2386 (Green)

StdDev Net Signals	1.30
Average BG Sub Signal	-0.65
StdDev BG Sub Signal	1.21

Local Bkg (inliers)

	Green
Number	13971
Avg	43.12
SD	1.78

Foreground Surface Fit

	Green
RMS_Fit	0.78
RMS_Resid	1.30
Avg_Fit	40.49

Multiplicative Surface Fit

	Green
RMS_Fit	0.11

Reproducibility: %CV for Replicated Probes

	Median %CV Signal (inliers)	
	Non-Control probes	Agilent SpikeIns
	Green	Green
BGSubSignal	-1.00	11.88
ProcessedSignal	-1.00	4.38

Agilent SpikeIns Signal Statistics

Probe Name	Log (Relative Conc.)	Median (Log Proc. Sig.)	% CV	StdDev
(+)E1A_r60_3	0.30	0.14	32.09	0.11
(+)E1A_r60_a104	1.30	0.49	43.49	0.21
(+)E1A_r60_a107	2.30	1.29	8.98	0.04
(+)E1A_r60_a135	3.30	2.21	5.42	0.02
(+)E1A_r60_a20	3.83	2.63	4.38	0.02
(+)E1A_r60_a22	4.30	3.12	2.93	0.01
(+)E1A_r60_a97	4.82	3.82	3.85	0.02
(+)E1A_r60_n11	5.30	4.37	3.91	0.02
(+)E1A_r60_n9	5.82	4.72	4.70	0.02
(+)E1A_r60_1	6.30	5.30	5.82	0.03

Agilent SpikeIns: %CV of Avg. Processed Signal Plot

Spatial Distribution of Median Signals for each Row

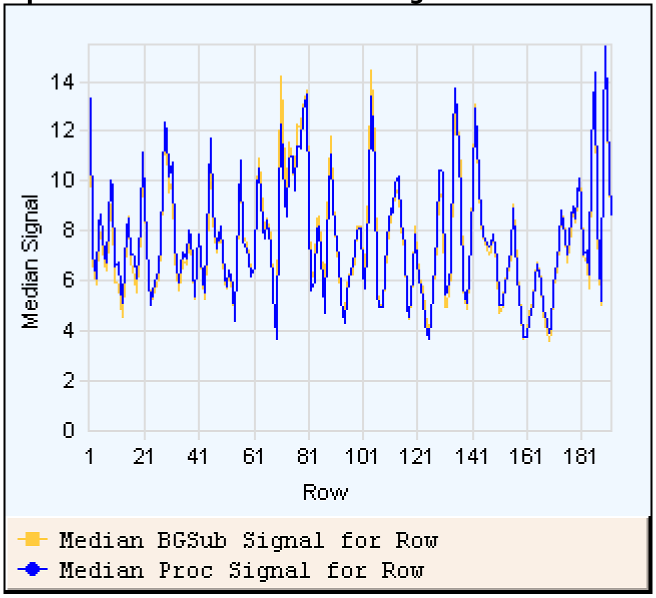

Spatial Distribution of Median Signals for each Column

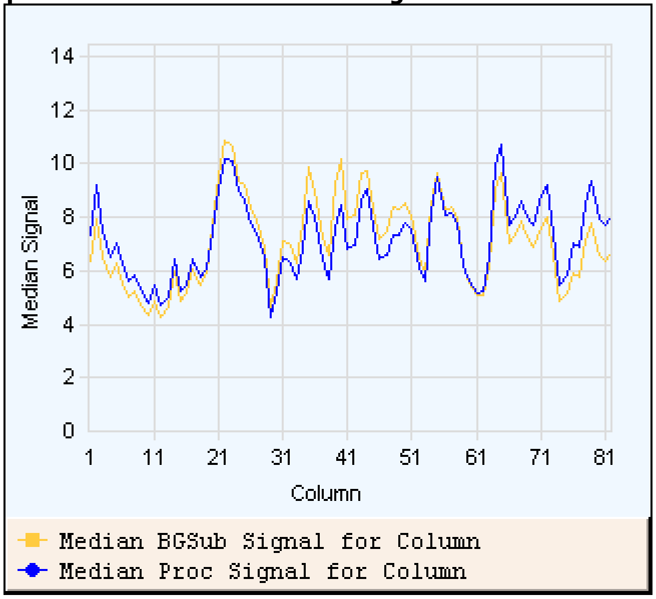

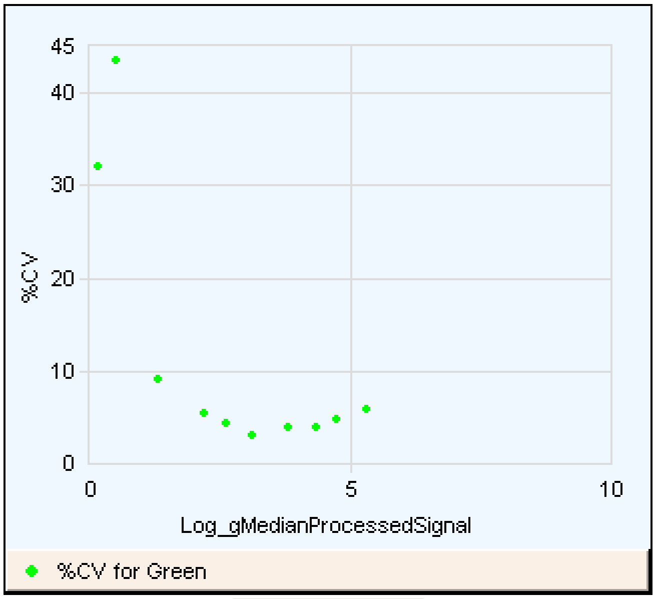

Median %CV:4.38

Agilent SpikeIns: Log(Signal) vs. Log(Relative concentration) Plot

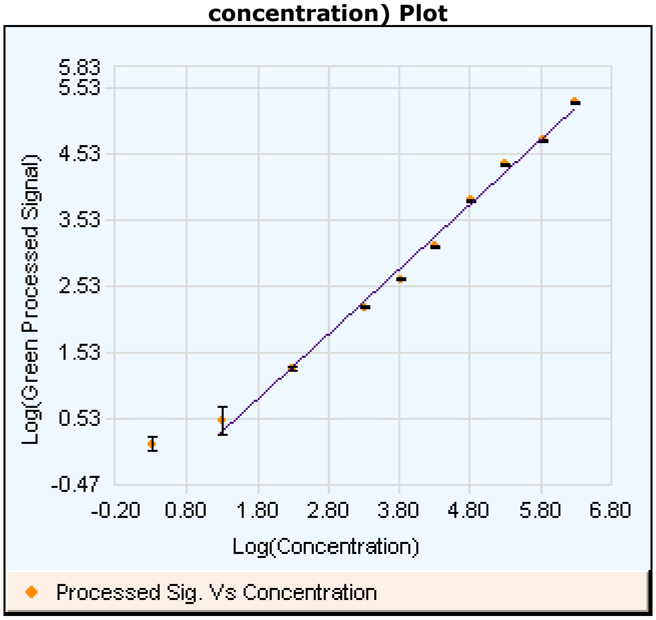

Agilent Spike-In Concentration-Response Statistics

Linear Range Statistics:

Low Signal	0.32
High Signal	5.63
Low Relative Concentration	1.29
High Relative Concentration	6.73
Slope	0.98
R^2 Value	0.99

Signal Detection Limit Statistics

Saturation Point	5.78
Low Threshold	-0.06
Low Threshold Error	0.24
Spike-In Detection Limit	0.24

QC Report - Agilent Technologies : 1 Color Gene Expression

Date	Wednesday, September 21, 2011 - 10:49	Grid	035923_D_F_20110809
Image	UniversityPadova_253592310005_S01_H [2_3]	BG Method	No Background
Protocol	GE1-v5_95_Feb07 (Read Only)	Background Detrend	On(FeatNCRange, LoPass)
User Name	Administrator	Multiplicative Detrend	True
FE Version	9.5.1.1	Additive Error	2(Green)
		Saturation Value	584301 (g)

Spot Finding of the Four Corners of the Array

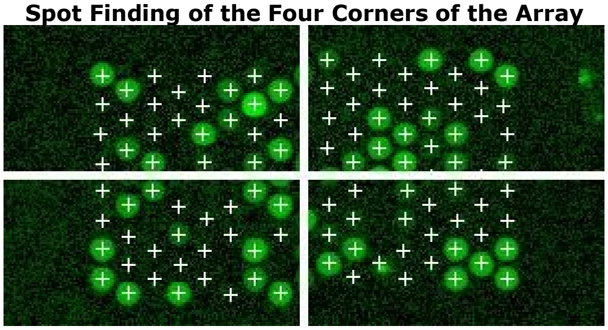

Grid Normal

	Feature Green	Local Background Green
Non Uniform	2	22
Population	17	153

Spatial Distribution of All Outliers on the Array

192 rows x 82 columns

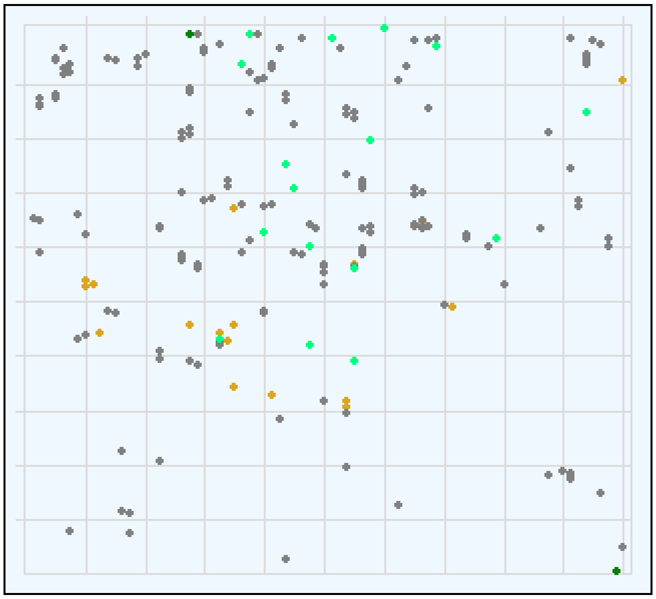

FeatureNonUnif (Green) = 2(0.01%)

GeneNonUnif (Green) = 1 (0.007 %)

BG NonUniform, BG Population, Green FeaturePopulation, Green Feature NonUniform

Negative Control Stats

	Green
Average Net Signals	15.87

Net Signal Statistics

Agilent SpikeIns:

	Green
# Saturated Features	0
99% of Sig. Distrib.	177082
50% of Sig. Distrib.	480
1% of Sig. Distrib.	15

Non-Control probes:

	Green
# Saturated Features	0
99% of Sig. Distrib.	46151
50% of Sig. Distrib.	46
1% of Sig. Distrib.	13

Histogram of Signals Plot

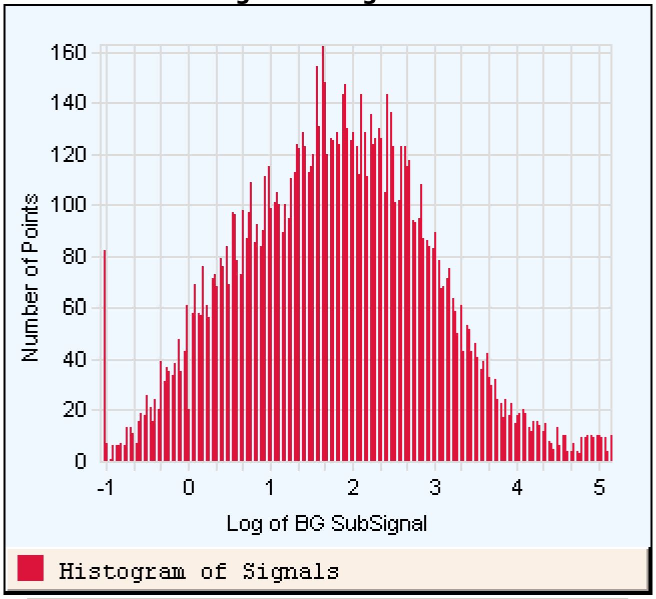

Features (NonCtrl) with BGSubSignal < 0: 2112 (Green)

StdDev Net Signals	2.02
Average BG Sub Signal	-1.25
StdDev BG Sub Signal	1.43

Local Bkg (inliers)

	Green
Number	14035
Avg	42.21
SD	2.12

Foreground Surface Fit

	Green
RMS_Fit	1.93
RMS_Resid	1.95
Avg_Fit	46.06

Multiplicative Surface Fit

	Green
RMS_Fit	0.11

Reproducibility: %CV for Replicated Probes

	Median %CV Signal (inliers)	
	Non-Control probes	Agilent SpikeIns
	Green	Green
BGSubSignal	-1.00	12.58
ProcessedSignal	-1.00	3.58

Agilent SpikeIns Signal Statistics

Probe Name	Log (Relative Conc.)	Median (Log Proc. Sig.)	% CV	StdDev
(+)E1A_r60_3	0.30	0.31	27.17	0.10
(+)E1A_r60_a104	1.30	0.56	45.70	0.18
(+)E1A_r60_a107	2.30	1.26	14.77	0.07
(+)E1A_r60_a135	3.30	2.12	5.48	0.02
(+)E1A_r60_a20	3.83	2.54	4.43	0.02
(+)E1A_r60_a22	4.30	3.02	3.58	0.02
(+)E1A_r60_a97	4.82	3.76	3.23	0.01
(+)E1A_r60_n11	5.30	4.32	3.07	0.01
(+)E1A_r60_n9	5.82	4.68	2.29	0.01
(+)E1A_r60_1	6.30	5.21	4.45	0.02

Agilent SpikeIns: %CV of Avg. Processed Signal Plot

Spatial Distribution of Median Signals for each Row

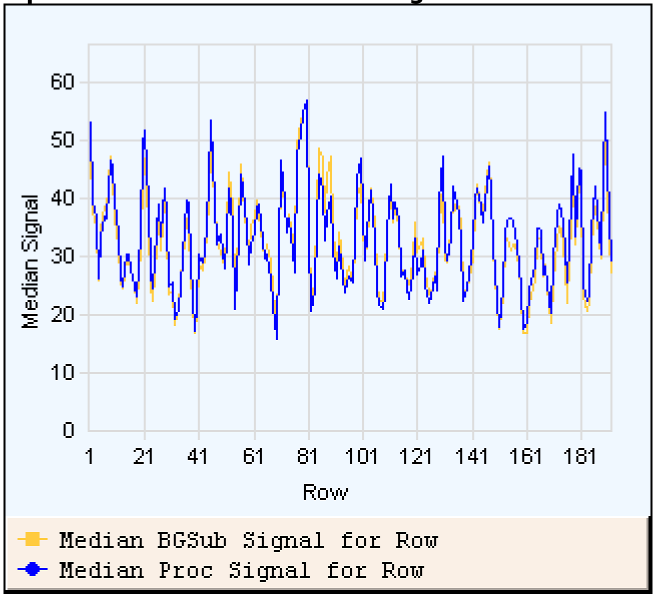

Spatial Distribution of Median Signals for each Column

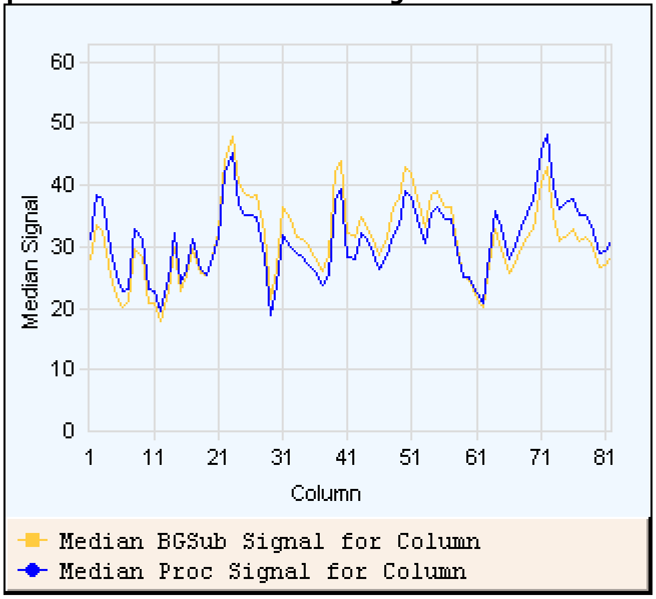

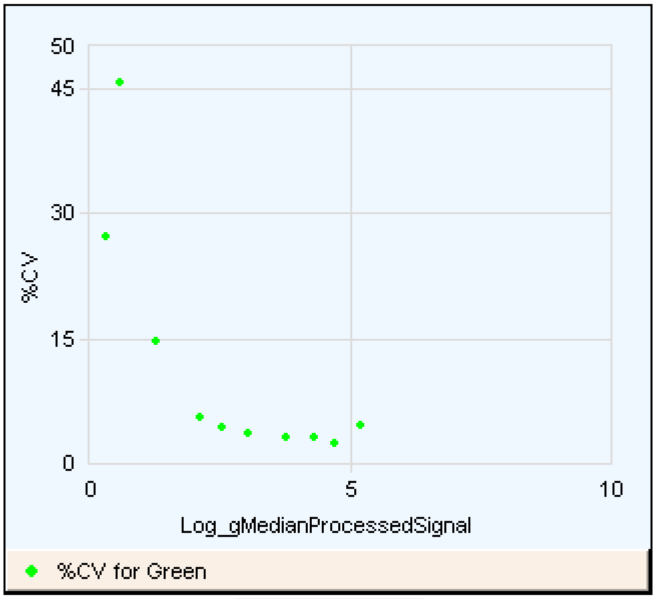

Median %CV:3.58

Agilent SpikeIns: Log(Signal) vs. Log(Relative concentration) Plot

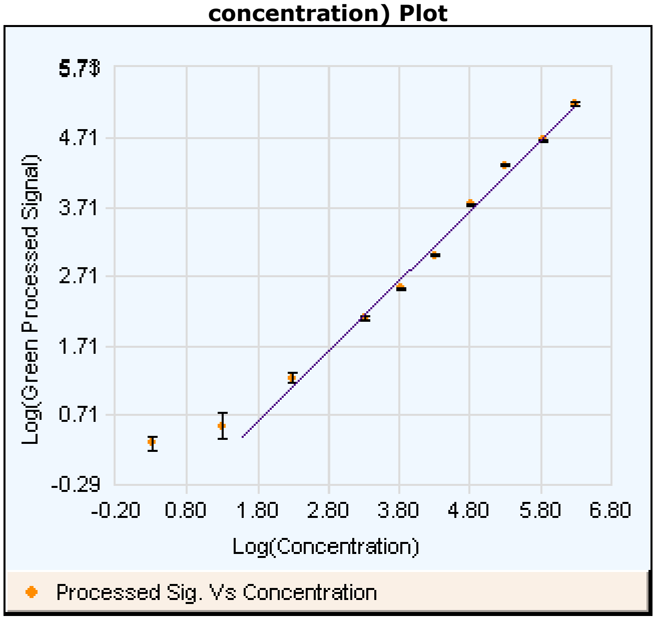

Agilent Spike-In Concentration-Response Statistics

Linear Range Statistics:

Low Signal	0.40
High Signal	5.69
Low Relative Concentration	1.58
High Relative Concentration	6.79
Slope	1.01
R^2 Value	0.99

Signal Detection Limit Statistics

Saturation Point	5.77
Low Threshold	0.17
Low Threshold Error	0.21
Spike-In Detection Limit	0.75

QC Report - Agilent Technologies : 1 Color Gene Expression

Date	Wednesday, September 21, 2011 - 10:50	Grid	035923_D_F_20110809
Image	UniversityPadova_253592310005_S01_H [2_4]	BG Method	No Background
Protocol	GE1-v5_95_Feb07 (Read Only)	Background Detrend	On(FeatNCRange, LoPass)
User Name	Administrator	Multiplicative Detrend	True
FE Version	9.5.1.1	Additive Error	2(Green)
		Saturation Value	584625 (g)

Spot Finding of the Four Corners of the Array

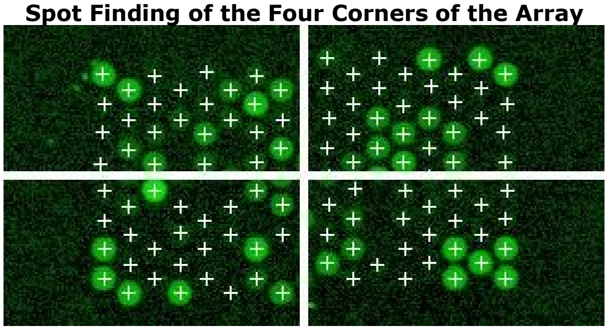

Grid Normal

	Feature Green	Local Background Green
Non Uniform	0	24
Population	17	245

Spatial Distribution of All Outliers on the Array

192 rows x 82 columns

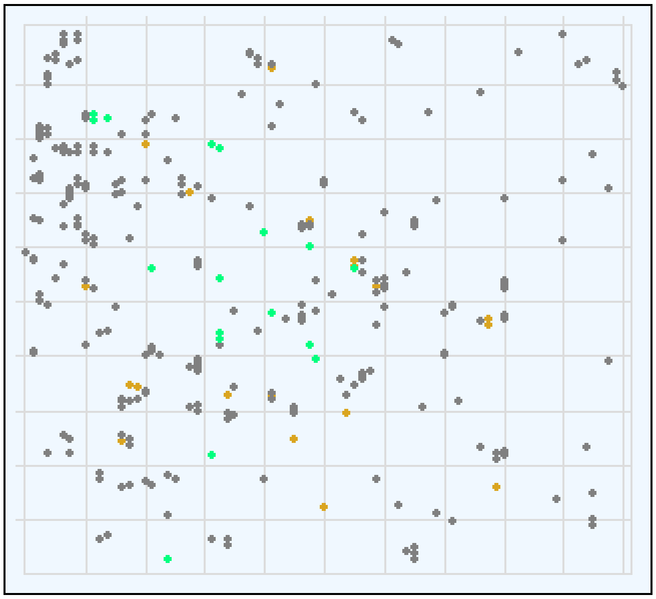

FeatureNonUnif (Green) = 0(0.00%)

GeneNonUnif (Green) = 0 (0.000 %)

BG NonUniform · BG Population · Green FeaturePopulation · Green Feature NonUniform

Negative Control Stats

	Green
Average Net Signals	17.18

Net Signal Statistics

Agilent SpikeIns:

	Green
# Saturated Features	0
99% of Sig. Distrib.	215951
50% of Sig. Distrib.	540
1% of Sig. Distrib.	16

Non-Control probes:

	Green
# Saturated Features	0
99% of Sig. Distrib.	34423
50% of Sig. Distrib.	32
1% of Sig. Distrib.	14

Histogram of Signals Plot

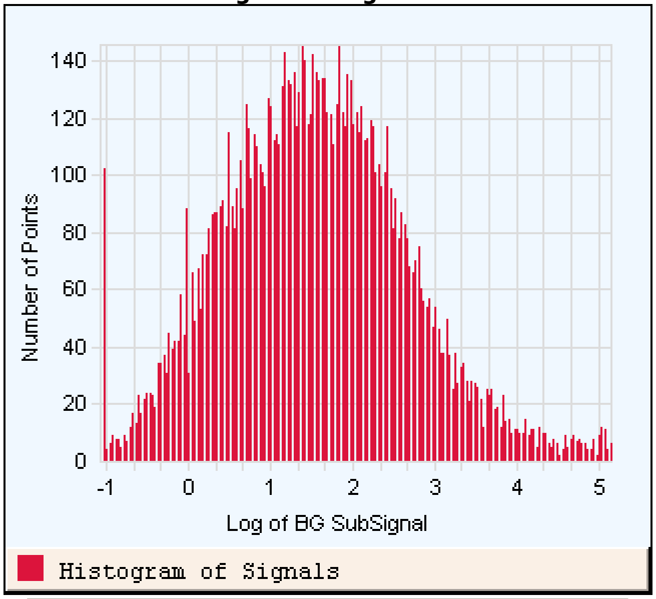

Features (NonCtrl) with BGSubSignal < 0: 2837 (Green)

StdDev Net Signals	1.90
Average BG Sub Signal	-1.67
StdDev BG Sub Signal	1.53

Local Bkg (inliers)

	Green
Number	13943
Avg	40.96
SD	1.77

Foreground Surface Fit

	Green
RMS_Fit	1.52
RMS_Resid	1.94
Avg_Fit	47.80

Multiplicative Surface Fit

	Green
RMS_Fit	0.11

Reproducibility: %CV for Replicated Probes

	Median %CV Signal (inliers)	
	Non-Control probes	Agilent SpikeIns
	Green	Green
BGSubSignal	-1.00	11.35
ProcessedSignal	-1.00	3.91

Agilent SpikeIns Signal Statistics

Probe Name	Log (Relative Conc.)	Median (Log Proc. Sig.)	% CV	StdDev
(+)E1A_r60_3	0.30	0.33	42.73	0.12
(+)E1A_r60_a104	1.30	0.66	45.85	0.22
(+)E1A_r60_a107	2.30	1.35	12.34	0.05
(+)E1A_r60_a135	3.30	2.22	5.05	0.02
(+)E1A_r60_a20	3.83	2.60	3.91	0.02
(+)E1A_r60_a22	4.30	3.09	3.99	0.02
(+)E1A_r60_a97	4.82	3.85	2.90	0.01
(+)E1A_r60_n11	5.30	4.41	2.72	0.01
(+)E1A_r60_n9	5.82	4.76	3.08	0.01
(+)E1A_r60_1	6.30	5.30	4.71	0.02

Agilent SpikeIns: %CV of Avg. Processed Signal Plot

Spatial Distribution of Median Signals for each Row

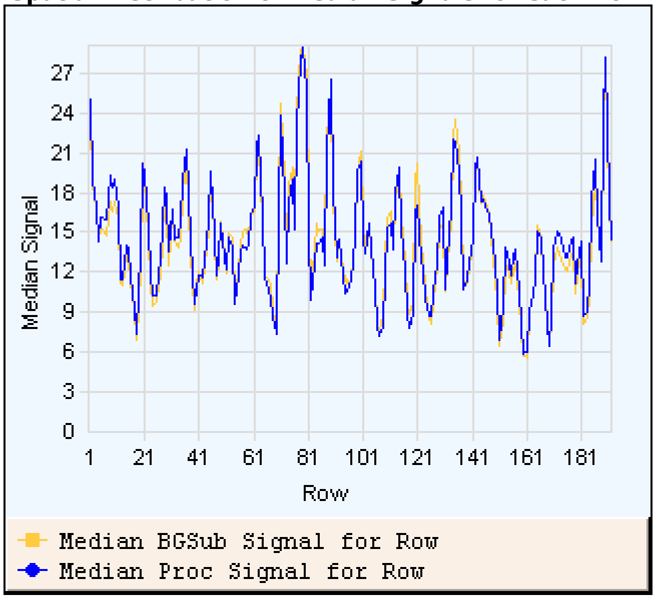

Spatial Distribution of Median Signals for each Column

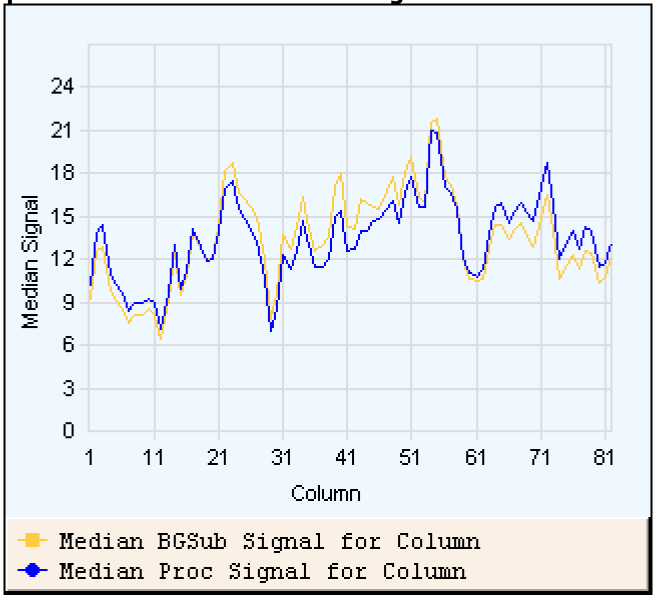

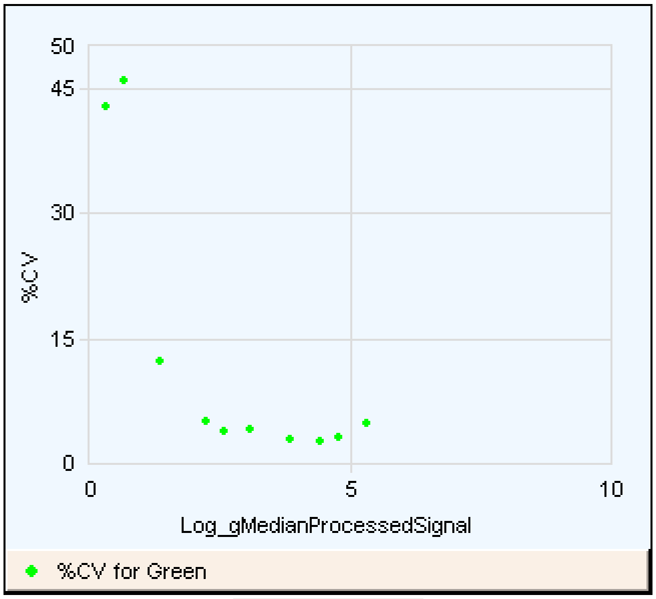

Median %CV:3.91

Agilent SpikeIns: Log(Signal) vs. Log(Relative concentration) Plot

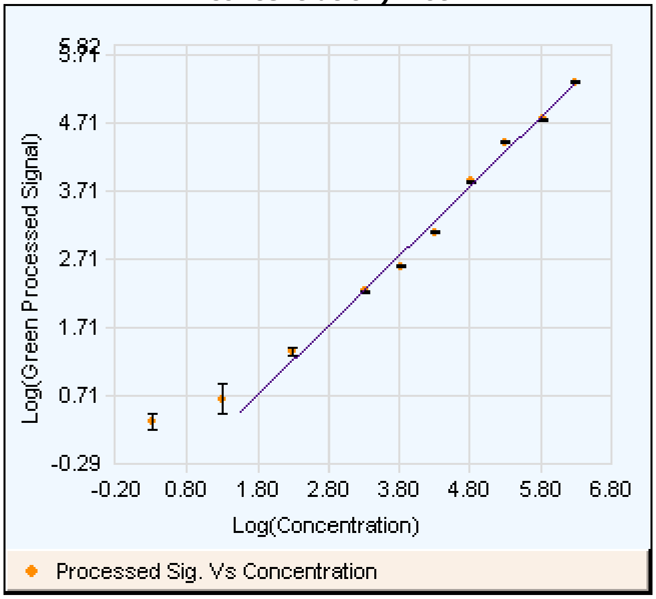

Agilent Spike-In Concentration-Response Statistics

Linear Range Statistics:

Low Signal	0.47
High Signal	5.67
Low Relative Concentration	1.56
High Relative Concentration	6.69
Slope	1.01
R^2 Value	0.99

Signal Detection Limit Statistics

Saturation Point	5.77
Low Threshold	0.22
Low Threshold Error	0.25
Spike-In Detection Limit	0.81

QC Report - Agilent Technologies : 1 Color Gene Expression

Date	Friday, September 23, 2011 - 11:03	Grid	035923_D_F_20110809
Image	UniversityPadova_253592310006_S01_H [2_1]	BG Method	No Background
Protocol	GE1-v5_95_Feb07 (Read Only)	Background Detrend	On(FeatNCRange, LoPass)
User Name	Administrator	Multiplicative Detrend	True
FE Version	9.5.1.1	Additive Error	3(Green)
		Saturation Value	590544 (g)

Spot Finding of the Four Corners of the Array

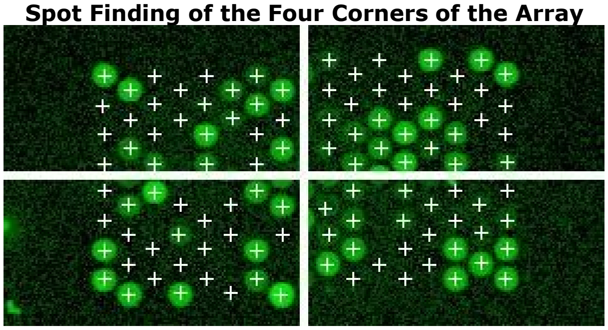

Grid Normal

	Feature Green	Local Background Green
Non Uniform	6	34
Population	29	150

Spatial Distribution of All Outliers on the Array

192 rows x 82 columns

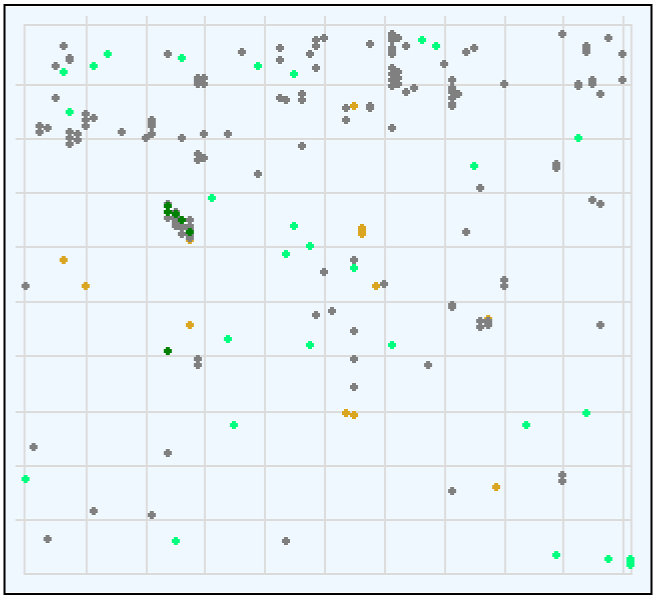

FeatureNonUnif (Green) = 6(0.04%)

GeneNonUnif (Green) = 6 (0.044 %)

BG NonUniform BG Population

Green FeaturePopulation Green Feature NonUniform

Negative Control Stats

	Green
Average Net Signals	13.41

Net Signal Statistics

Agilent SpikeIns:

	Green
# Saturated Features	0
99% of Sig. Distrib.	91770
50% of Sig. Distrib.	251
1% of Sig. Distrib.	11

Non-Control probes:

	Green
# Saturated Features	0
99% of Sig. Distrib.	49311
50% of Sig. Distrib.	43
1% of Sig. Distrib.	10

Histogram of Signals Plot

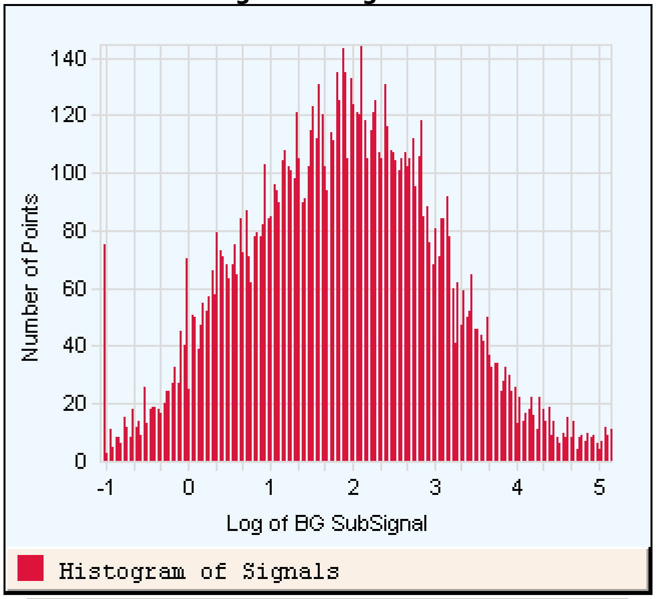

Features (NonCtrl) with BGSubSignal < 0: 2834 (Green)

StdDev Net Signals	2.48
Average BG Sub Signal	-2.46
StdDev BG Sub Signal	1.69

Local Bkg (inliers)

	Green
Number	14044
Avg	43.91
SD	2.67

Foreground Surface Fit

	Green
RMS_Fit	1.86
RMS_Resid	2.74
Avg_Fit	44.19

Multiplicative Surface Fit

	Green
RMS_Fit	0.08

Reproducibility: %CV for Replicated Probes

	Median %CV Signal (inliers)	
	Non-Control probes	Agilent SpikeIns
	Green	Green
BGSubSignal	-1.00	10.83
ProcessedSignal	-1.00	6.69

Agilent SpikeIns Signal Statistics

Probe Name	Log (Relative Conc.)	Median (Log Proc. Sig.)	% CV	StdDev
(+)E1A_r60_3	0.30	0.49	163.48	0.34
(+)E1A_r60_a104	1.30	0.46	185.37	0.29
(+)E1A_r60_a107	2.30	0.98	23.39	0.11
(+)E1A_r60_a135	3.30	1.81	26.47	0.10
(+)E1A_r60_a20	3.83	2.26	8.14	0.03
(+)E1A_r60_a22	4.30	2.78	6.69	0.03
(+)E1A_r60_a97	4.82	3.55	6.35	0.03
(+)E1A_r60_n11	5.30	4.02	6.01	0.03
(+)E1A_r60_n9	5.82	4.43	11.13	0.05
(+)E1A_r60_1	6.30	4.94	4.10	0.02

Agilent SpikeIns: %CV of Avg. Processed Signal Plot

Spatial Distribution of Median Signals for each Row

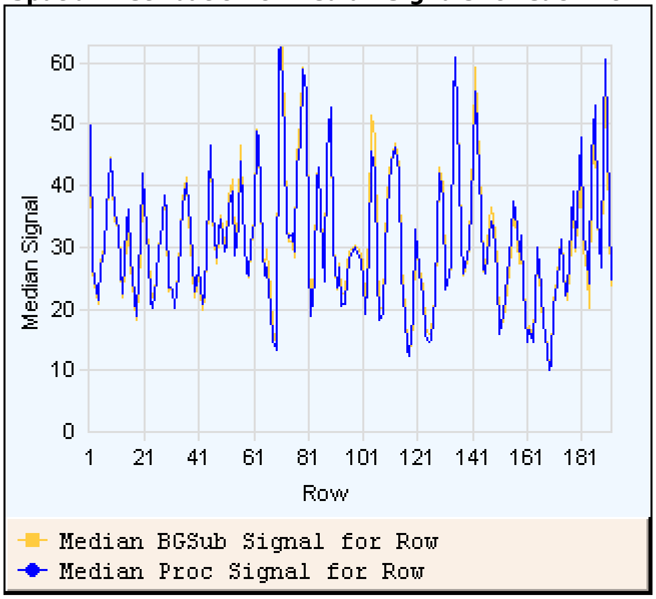

Spatial Distribution of Median Signals for each Column

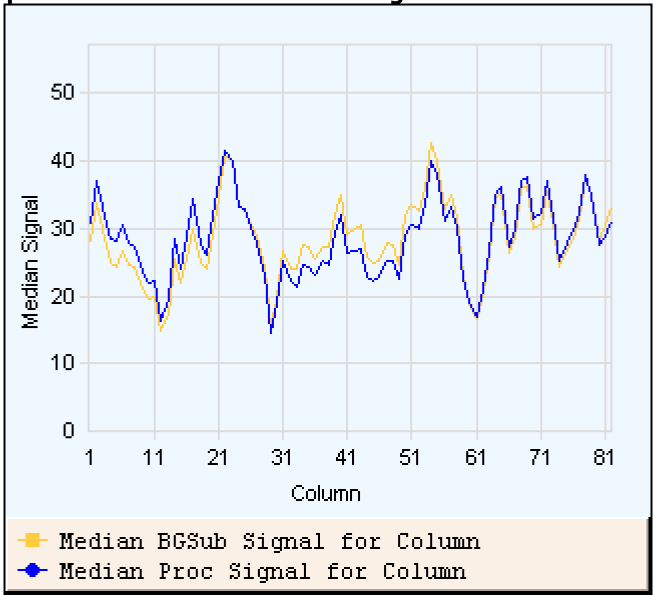

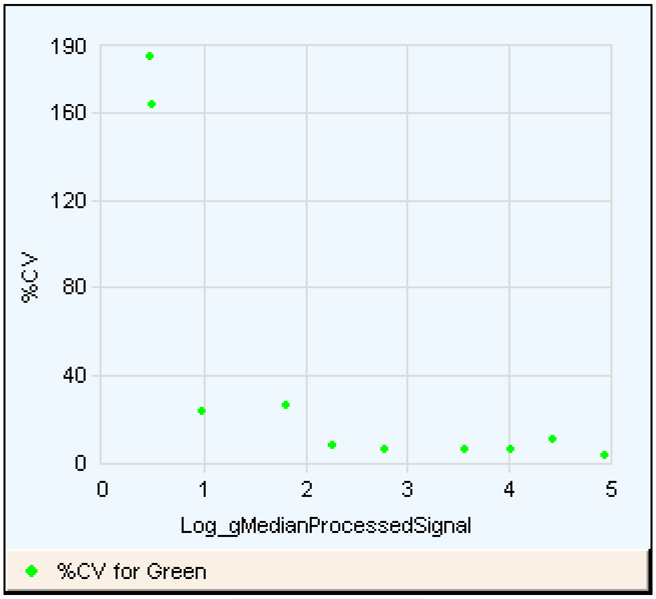

Median %CV:6.69

Agilent SpikeIns: Log(Signal) vs. Log(Relative concentration) Plot

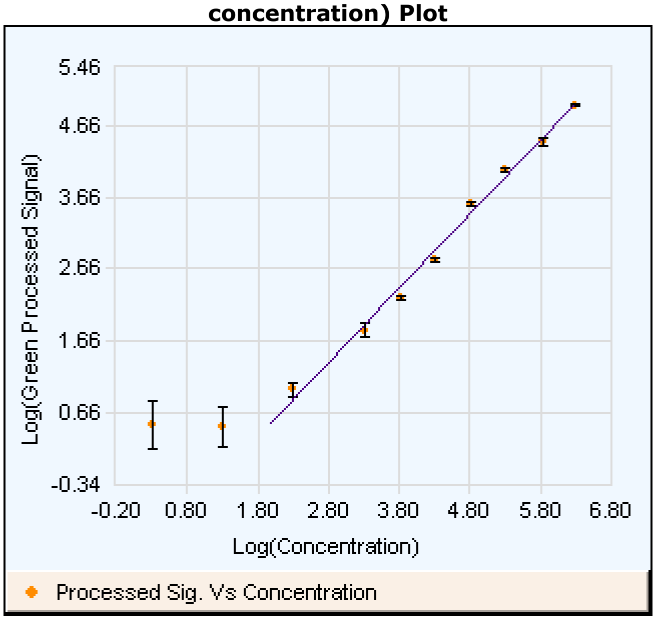

Agilent Spike-In Concentration-Response Statistics

Linear Range Statistics:

Low Signal	0.51
High Signal	5.69
Low Relative Concentration	1.97
High Relative Concentration	7.03
Slope	1.02
R^2 Value	0.99

Signal Detection Limit Statistics

Saturation Point	5.77
Low Threshold	0.27
Low Threshold Error	0.46
Spike-In Detection Limit	1.24

QC Report - Agilent Technologies : 1 Color Gene Expression

Date	Friday, September 23, 2011 - 11:03	Grid	035923_D_F_20110809
Image	UniversityPadova_253592310006_S01_H [2_2]	BG Method	No Background
Protocol	GE1-v5_95_Feb07 (Read Only)	Background Detrend	On(FeatNCRange, LoPass)
User Name	Administrator	Multiplicative Detrend	True
FE Version	9.5.1.1	Additive Error	2(Green)
		Saturation Value	590747 (g)

Spot Finding of the Four Corners of the Array

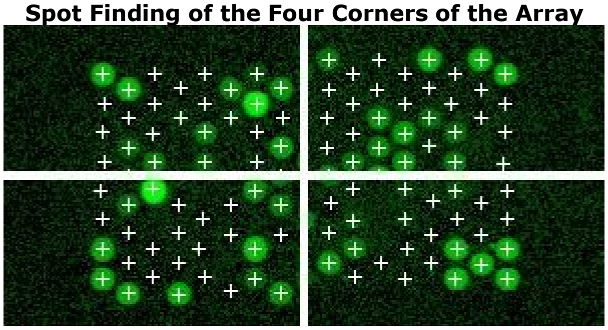

Grid Normal

	Feature	Local Background
	Green	Green
Non Uniform	0	20
Population	27	150

Spatial Distribution of All Outliers on the Array

192 rows x 82 columns

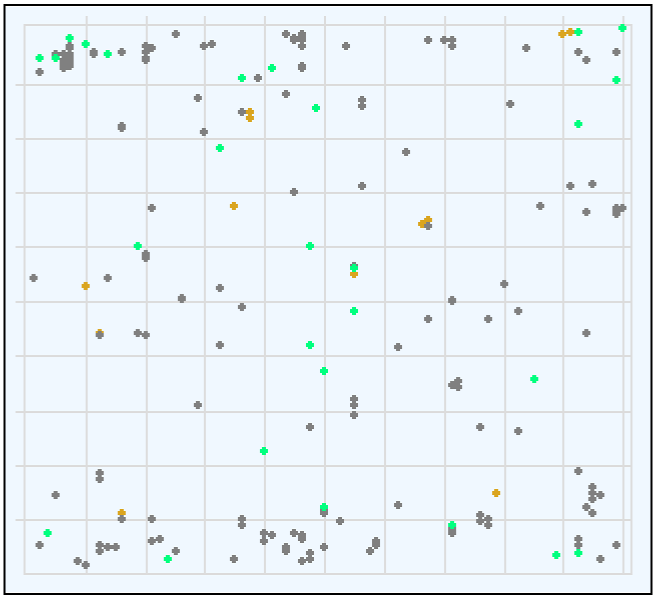

FeatureNonUnif (Green) = 0(0.00%)

GeneNonUnif (Green) = 0 (0.000 %)

BG NonUniform BG Population

Green FeaturePopulation Green Feature NonUniform

Negative Control Stats

	Green
Average Net Signals	14.34

Net Signal Statistics

Agilent SpikeIns:

	Green
# Saturated Features	0
99% of Sig. Distrib.	119352
50% of Sig. Distrib.	274
1% of Sig. Distrib.	12

Non-Control probes:

	Green
# Saturated Features	0
99% of Sig. Distrib.	32292
50% of Sig. Distrib.	33
1% of Sig. Distrib.	12

Histogram of Signals Plot

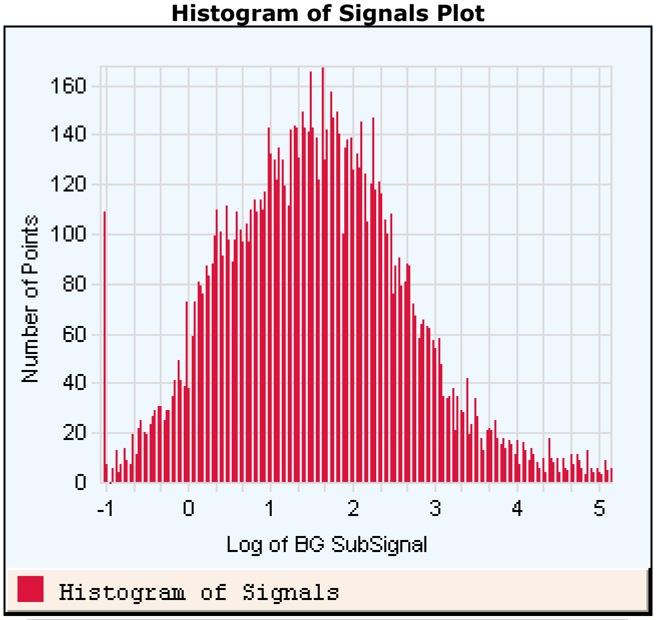

Features (NonCtrl) with BGSubSignal < 0: 2193 (Green)

StdDev Net Signals	1.92
Average BG Sub Signal	-1.40
StdDev BG Sub Signal	1.60

Local Bkg (inliers)

	Green
Number	14044
Avg	44.29
SD	2.35

Foreground Surface Fit

	Green
RMS_Fit	1.08
RMS_Resid	1.83
Avg_Fit	44.14

Multiplicative Surface Fit

	Green
RMS_Fit	0.05

Reproducibility: %CV for Replicated Probes

	Median %CV Signal (inliers)	
	Non-Control probes	Agilent SpikeIns
	Green	Green
BGSubSignal	-1.00	6.52
ProcessedSignal	-1.00	4.15

Agilent SpikeIns Signal Statistics

Probe Name	Log (Relative Conc.)	Median (Log Proc. Sig.)	% CV	StdDev
(+)E1A_r60_3	0.30	0.28	51.09	0.17
(+)E1A_r60_a104	1.30	0.31	39.79	0.15
(+)E1A_r60_a107	2.30	1.11	23.51	0.10
(+)E1A_r60_a135	3.30	1.88	6.54	0.03
(+)E1A_r60_a20	3.83	2.36	4.63	0.02
(+)E1A_r60_a22	4.30	2.85	3.99	0.02
(+)E1A_r60_a97	4.82	3.62	3.04	0.01
(+)E1A_r60_n11	5.30	4.12	4.15	0.02
(+)E1A_r60_n9	5.82	4.56	5.46	0.02
(+)E1A_r60_1	6.30	5.06	2.79	0.01

Agilent SpikeIns: %CV of Avg. Processed Signal Plot

Spatial Distribution of Median Signals for each Row

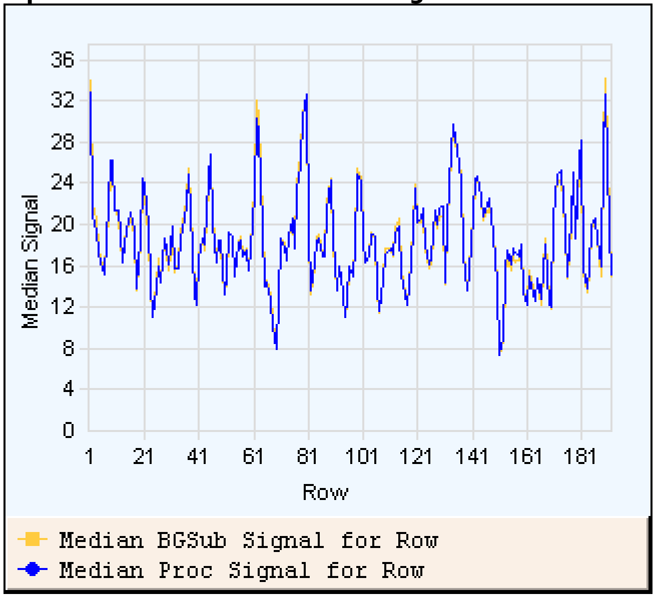

Spatial Distribution of Median Signals for each Column

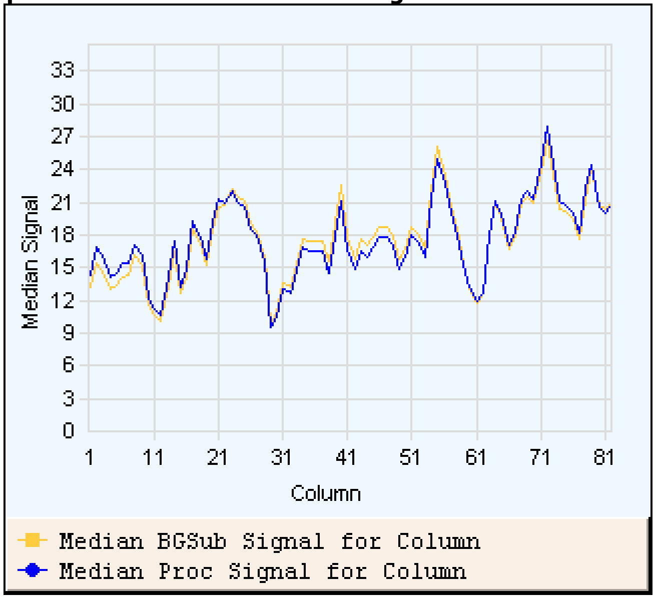

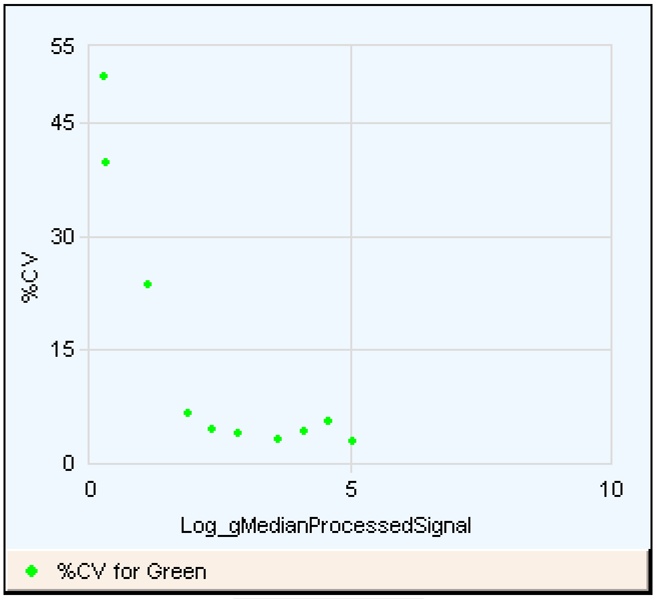

Median %CV:4.15

Agilent SpikeIns: Log(Signal) vs. Log(Relative concentration) Plot

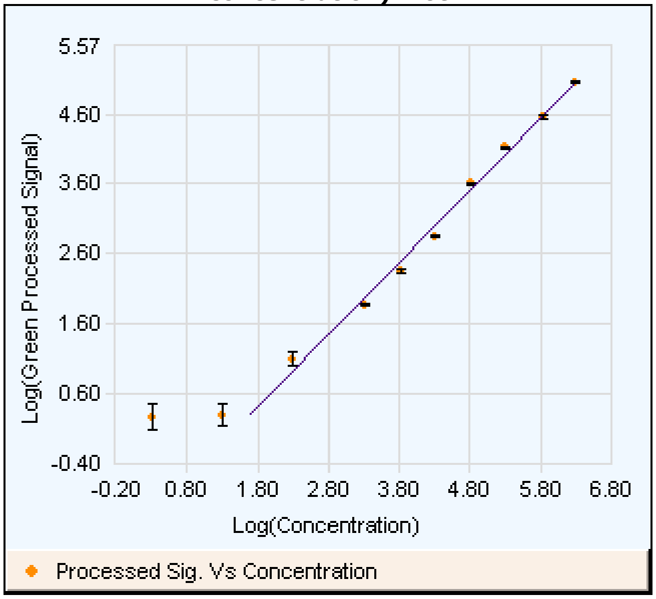

Agilent Spike-In Concentration-Response Statistics

Linear Range Statistics:

Low Signal	0.32
High Signal	5.69
Low Relative Concentration	1.70
High Relative Concentration	6.93
Slope	1.03
R^2 Value	0.99

Signal Detection Limit Statistics

Saturation Point	5.77
Low Threshold	0.05
Low Threshold Error	0.25
Spike-In Detection Limit	0.56

QC Report - Agilent Technologies : 1 Color Gene Expression

Date	Friday, September 02, 2011 - 10:56	Grid	035923_D_F_20110809
Image	UniversityPadova_253592310001_S01_H [2_4]	BG Method	No Background
Protocol	GE1-v5_95_Feb07 (Read Only)	Background Detrend	On(FeatNCRange, LoPass)
User Name	Administrator	Multiplicative Detrend	True
FE Version	9.5.1.1	Additive Error	2(Green)
		Saturation Value	608208 (g)

Spot Finding of the Four Corners of the Array

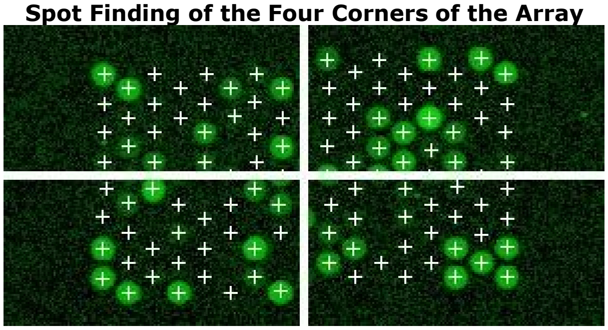

Grid Normal

	Feature Green	Local Background Green
Non Uniform	0	18
Population	16	196

Spatial Distribution of All Outliers on the Array

192 rows x 82 columns

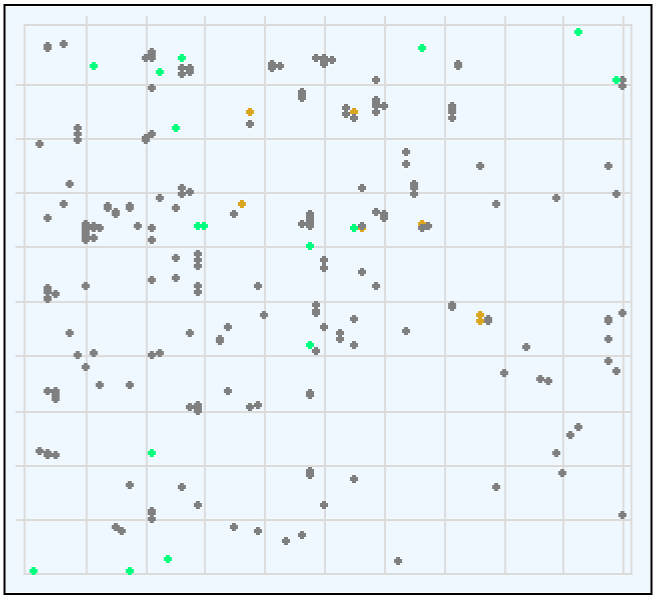

FeatureNonUnif (Green) = 0(0.00%)

GeneNonUnif (Green) = 0 (0.000 %)

BG NonUniform BG Population

Green FeaturePopulation Green Feature NonUniform

Negative Control Stats

	Green
Average Net Signals	12.19

Net Signal Statistics

Agilent SpikeIns:

	Green
# Saturated Features	0
99% of Sig. Distrib.	89946
50% of Sig. Distrib.	223
1% of Sig. Distrib.	10

Non-Control probes:

	Green
# Saturated Features	0
99% of Sig. Distrib.	29974
50% of Sig. Distrib.	25
1% of Sig. Distrib.	10

Histogram of Signals Plot

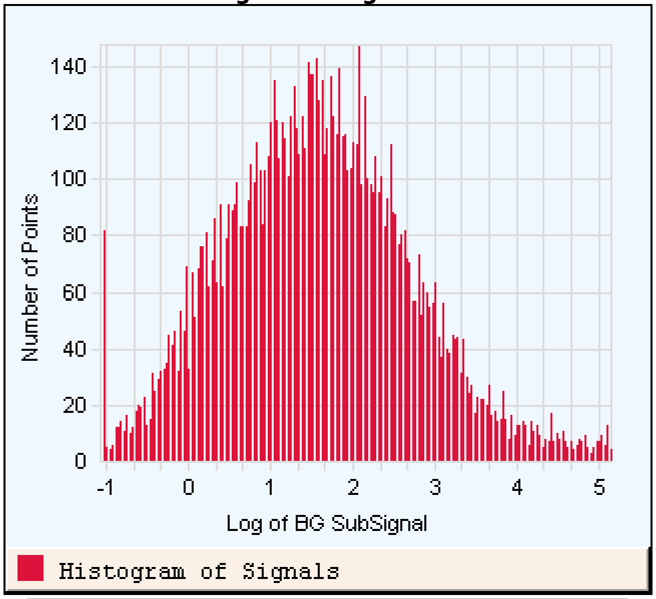

Features (NonCtrl) with BGSubSignal < 0: 3266 (Green)

StdDev Net Signals	1.86
Average BG Sub Signal	-1.67
StdDev BG Sub Signal	1.83

Local Bkg (inliers)

	Green
Number	14003
Avg	40.53
SD	1.83

Foreground Surface Fit

	Green
RMS_Fit	1.09
RMS_Resid	2.27
Avg_Fit	42.53

Multiplicative Surface Fit

	Green
RMS_Fit	0.11

Reproducibility: %CV for Replicated Probes

	Median %CV Signal (inliers)	
	Non-Control probes	Agilent SpikeIns
	Green	Green
BGSubSignal	-1.00	12.38
ProcessedSignal	-1.00	4.45

Agilent SpikeIns Signal Statistics

Probe Name	Log (Relative Conc.)	Median (Log Proc. Sig.)	% CV	StdDev
(+)E1A_r60_3	0.30	0.37	21.33	0.08
(+)E1A_r60_a104	1.30	0.41	102.79	0.21
(+)E1A_r60_a107	2.30	0.71	106.14	0.22
(+)E1A_r60_a135	3.30	1.71	7.46	0.03
(+)E1A_r60_a20	3.83	2.22	6.19	0.03
(+)E1A_r60_a22	4.30	2.67	3.85	0.02
(+)E1A_r60_a97	4.82	3.39	4.82	0.02
(+)E1A_r60_n11	5.30	3.91	4.18	0.02
(+)E1A_r60_n9	5.82	4.33	4.45	0.02
(+)E1A_r60_1	6.30	4.88	3.31	0.01

Agilent SpikeIns: %CV of Avg. Processed Signal Plot

Spatial Distribution of Median Signals for each Row

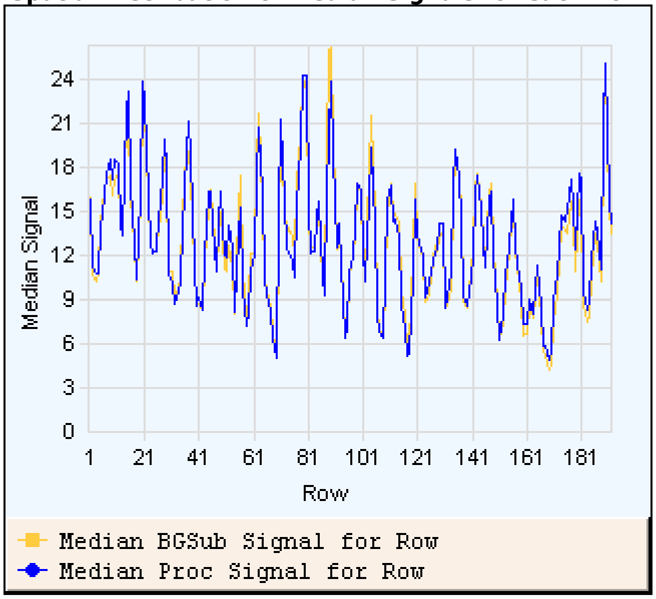

Spatial Distribution of Median Signals for each Column

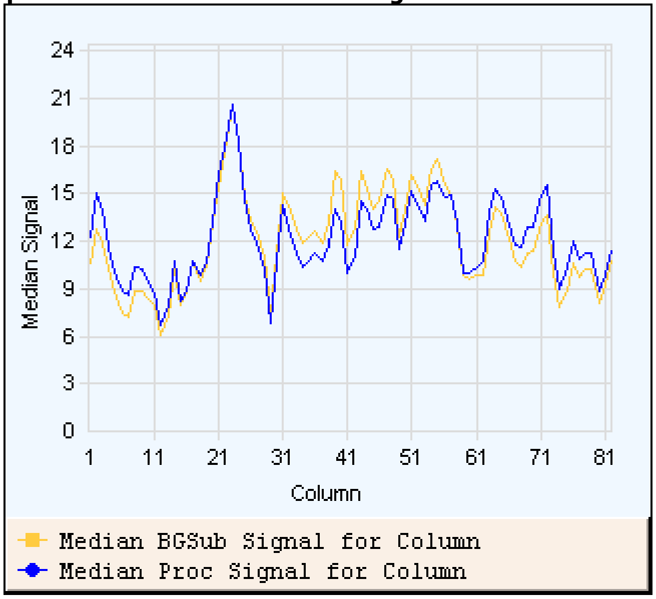

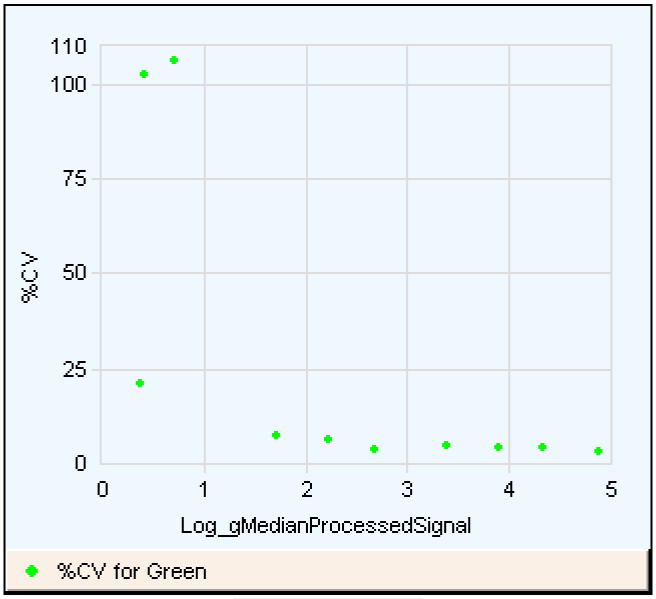

Median %CV:4.45

Agilent SpikeIns: Log(Signal) vs. Log(Relative concentration) Plot

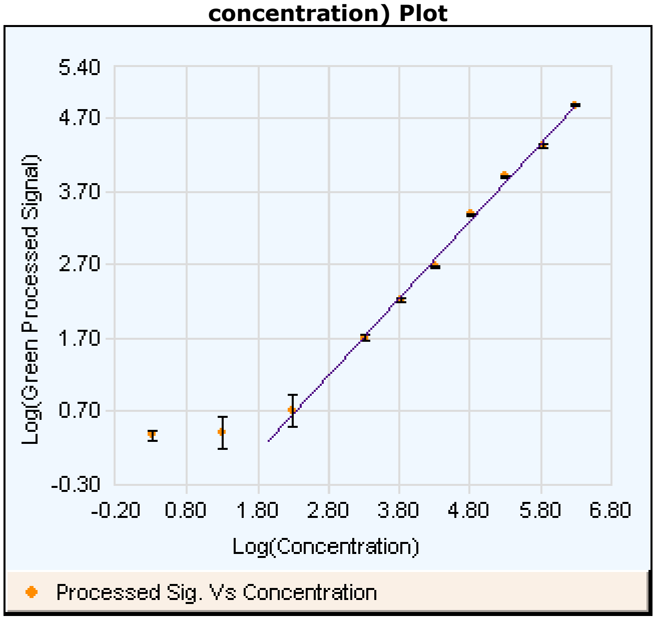

Agilent Spike-In Concentration-Response Statistics

Linear Range Statistics:

Low Signal	0.31
High Signal	5.76
Low Relative Concentration	1.96
High Relative Concentration	7.14
Slope	1.05
R^2 Value	1.00

Signal Detection Limit Statistics

Saturation Point	5.78
Low Threshold	0.13
Low Threshold Error	0.31
Spike-In Detection Limit	0.73

QC Report - Agilent Technologies : 1 Color Gene Expression

Date	Friday, September 23, 2011 - 11:03	Grid	035923_D_F_20110809
Image	UniversityPadova_253592310006_S01_H [2_3]	BG Method	No Background
Protocol	GE1-v5_95_Feb07 (Read Only)	Background Detrend	On(FeatNCRange, LoPass)
User Name	Administrator	Multiplicative Detrend	True
FE Version	9.5.1.1	Additive Error	2(Green)
		Saturation Value	590784 (g)

Spot Finding of the Four Corners of the Array

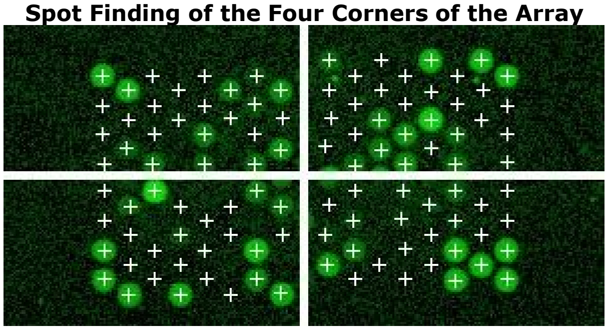

Grid Normal

	Feature	Local Background
	Green	Green
Non Uniform	0	26
Population	23	193

Spatial Distribution of All Outliers on the Array

192 rows x 82 columns

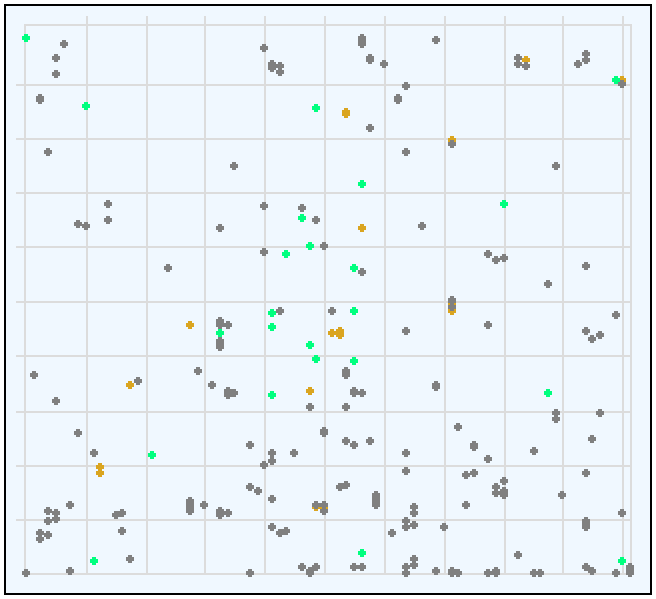

FeatureNonUnif (Green) = 0(0.00%)

GeneNonUnif (Green) = 0 (0.000 %)

BG NonUniform   BG Population
Green FeaturePopulation   Green Feature NonUniform

Negative Control Stats

	Green
Average Net Signals	13.90

Net Signal Statistics

Agilent SpikeIns:

	Green
# Saturated Features	0
99% of Sig. Distrib.	188658
50% of Sig. Distrib.	468
1% of Sig. Distrib.	13

Non-Control probes:

	Green
# Saturated Features	0
99% of Sig. Distrib.	30565
50% of Sig. Distrib.	30
1% of Sig. Distrib.	11

Histogram of Signals Plot

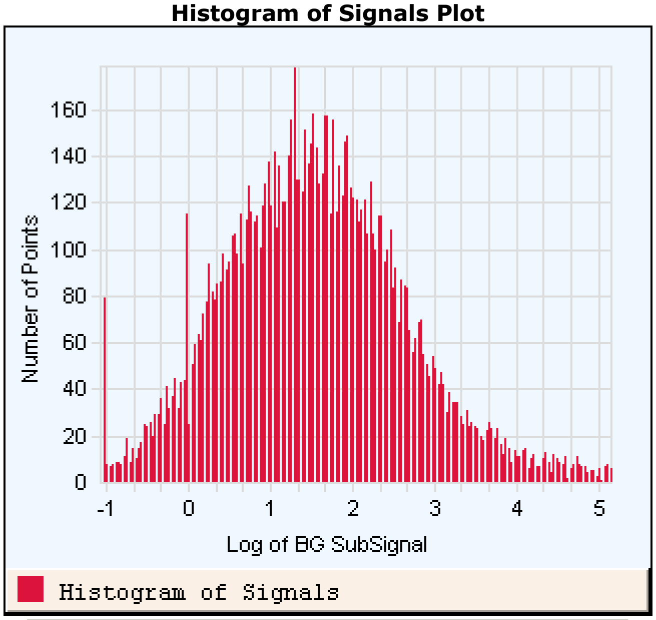

Features (NonCtrl) with BGSubSignal < 0: 2477 (Green)

StdDev Net Signals	2.66
Average BG Sub Signal	-1.36
StdDev BG Sub Signal	2.54

Local Bkg (inliers)

	Green
Number	13995
Avg	44.23
SD	2.25

Foreground Surface Fit

	Green
RMS_Fit	1.64
RMS_Resid	2.33
Avg_Fit	43.54

Multiplicative Surface Fit

	Green
RMS_Fit	0.10

Reproducibility: %CV for Replicated Probes

	Median %CV Signal (inliers)	
	Non-Control probes	Agilent SpikeIns
	Green	Green
BGSubSignal	-1.00	11.48
ProcessedSignal	-1.00	4.12

Agilent SpikeIns Signal Statistics

Probe Name	Log (Relative Conc.)	Median (Log Proc. Sig.)	% CV	StdDev
(+)E1A_r60_3	0.30	0.40	74.91	0.22
(+)E1A_r60_a104	1.30	0.59	32.50	0.14
(+)E1A_r60_a107	2.30	1.23	73.52	0.21
(+)E1A_r60_a135	3.30	2.12	4.45	0.02
(+)E1A_r60_a20	3.83	2.55	4.16	0.02
(+)E1A_r60_a22	4.30	3.07	4.77	0.02
(+)E1A_r60_a97	4.82	3.83	3.37	0.01
(+)E1A_r60_n11	5.30	4.35	3.41	0.01
(+)E1A_r60_n9	5.82	4.75	3.91	0.02
(+)E1A_r60_1	6.30	5.25	4.12	0.02

Agilent SpikeIns: %CV of Avg. Processed Signal Plot

Spatial Distribution of Median Signals for each Row

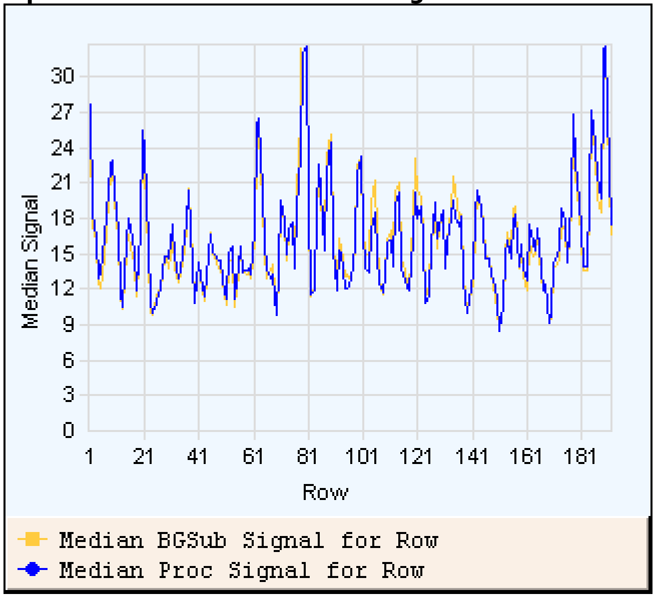

Spatial Distribution of Median Signals for each Column

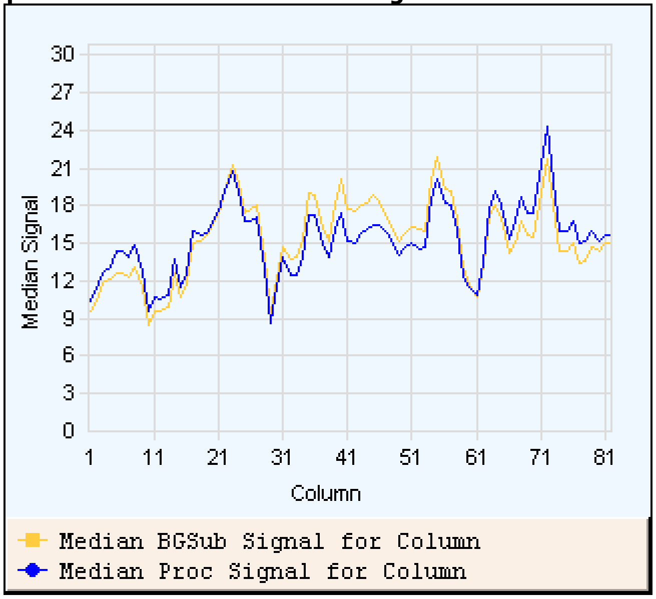

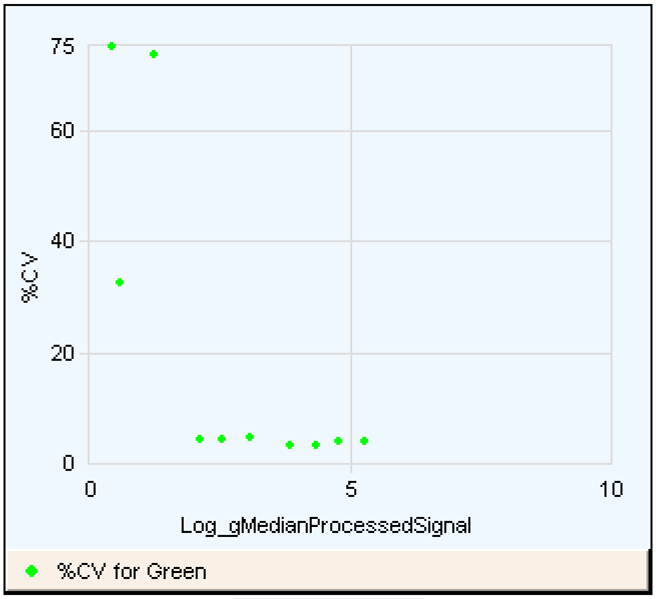

Median %CV:4.12

Agilent SpikeIns: Log(Signal) vs. Log(Relative concentration) Plot

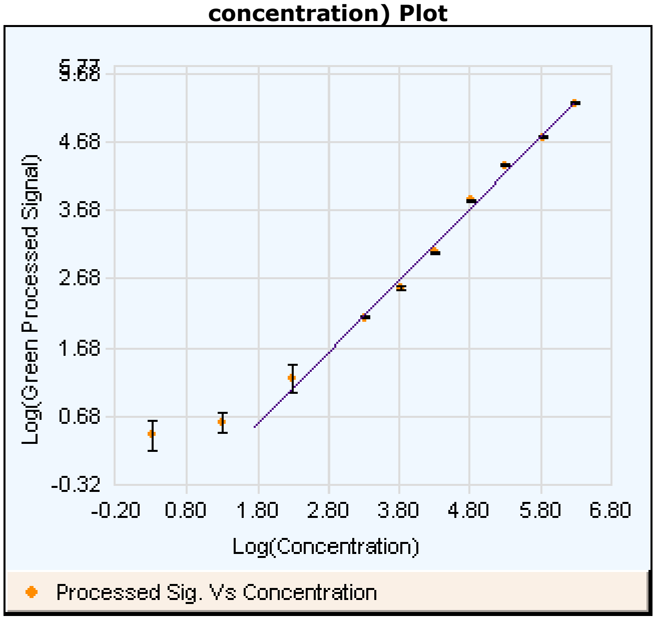

Agilent Spike-In Concentration-Response Statistics

Linear Range Statistics:

Low Signal	0.54
High Signal	5.66
Low Relative Concentration	1.74
High Relative Concentration	6.68
Slope	1.04
R^2 Value	0.99

Signal Detection Limit Statistics

Saturation Point	5.77
Low Threshold	0.27
Low Threshold Error	0.34
Spike-In Detection Limit	0.79

QC Report - Agilent Technologies : 1 Color Gene Expression

Date	Friday, September 23, 2011 - 11:03	Grid	035923_D_F_20110809
Image	UniversityPadova_253592310006_S01_H [2_4]	BG Method	No Background
Protocol	GE1-v5_95_Feb07 (Read Only)	Background Detrend	On(FeatNCRange, LoPass)
User Name	Administrator	Multiplicative Detrend	True
FE Version	9.5.1.1	Additive Error	2(Green)
		Saturation Value	593164 (g)

Spot Finding of the Four Corners of the Array

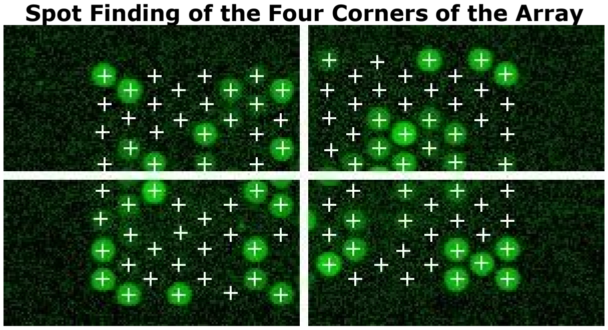

Grid Normal

	Feature Green	Local Background Green
Non Uniform	1	33
Population	19	208

Spatial Distribution of All Outliers on the Array

192 rows x 82 columns

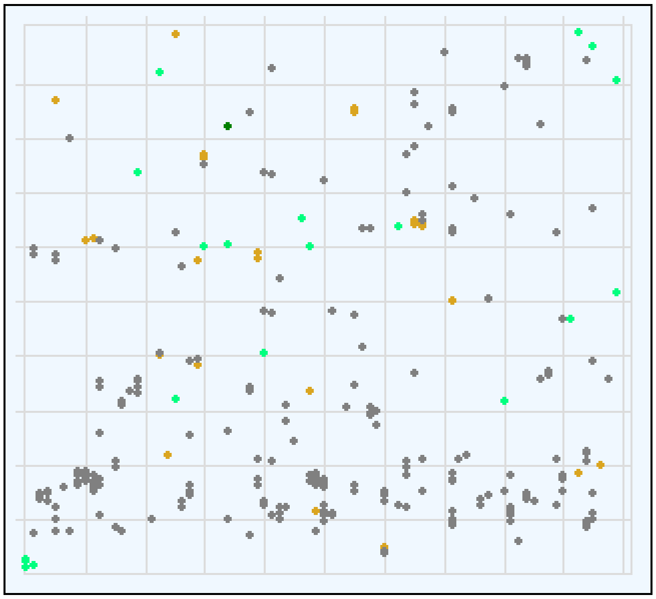

FeatureNonUnif (Green) = 1(0.01%)

GeneNonUnif (Green) = 1 (0.007 %)

BG NonUniform   BG Population

Green FeaturePopulation   Green Feature NonUniform

Negative Control Stats

	Green
Average Net Signals	12.47

Net Signal Statistics

Agilent SpikeIns:

	Green
# Saturated Features	0
99% of Sig. Distrib.	120558
50% of Sig. Distrib.	265
1% of Sig. Distrib.	12

Non-Control probes:

	Green
# Saturated Features	0
99% of Sig. Distrib.	58039
50% of Sig. Distrib.	43
1% of Sig. Distrib.	10

Histogram of Signals Plot

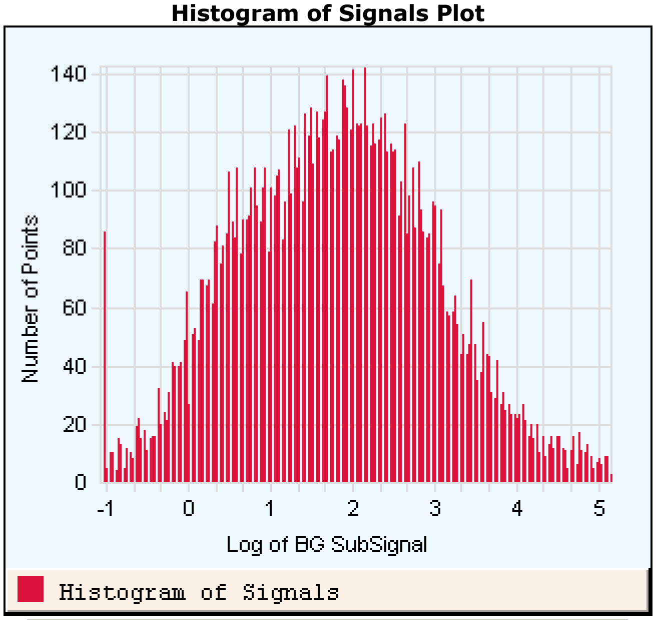

Features (NonCtrl) with BGSubSignal < 0: 2133 (Green)

StdDev Net Signals	1.87
Average BG Sub Signal	-1.42
StdDev BG Sub Signal	1.65

Local Bkg (inliers)

	Green
Number	13975
Avg	43.86
SD	2.30

Foreground Surface Fit

	Green
RMS_Fit	0.82
RMS_Resid	1.95
Avg_Fit	42.22

Multiplicative Surface Fit

	Green
RMS_Fit	0.11

Reproducibility: %CV for Replicated Probes

	Median %CV Signal (inliers)	
	Non-Control probes	Agilent SpikeIns
	Green	Green
BGSubSignal	-1.00	12.70
ProcessedSignal	-1.00	5.08

Agilent SpikeIns Signal Statistics

Probe Name	Log (Relative Conc.)	Median (Log Proc. Sig.)	% CV	StdDev
(+)E1A_r60_3	0.30	0.33	40.36	0.13
(+)E1A_r60_a104	1.30	0.34	35.20	0.12
(+)E1A_r60_a107	2.30	1.03	19.91	0.10
(+)E1A_r60_a135	3.30	1.87	10.60	0.04
(+)E1A_r60_a20	3.83	2.30	5.26	0.02
(+)E1A_r60_a22	4.30	2.86	3.52	0.02
(+)E1A_r60_a97	4.82	3.61	5.62	0.03
(+)E1A_r60_n11	5.30	4.12	4.33	0.02
(+)E1A_r60_n9	5.82	4.52	5.08	0.02
(+)E1A_r60_1	6.30	5.02	4.03	0.02

Agilent SpikeIns: %CV of Avg. Processed Signal Plot

Spatial Distribution of Median Signals for each Row

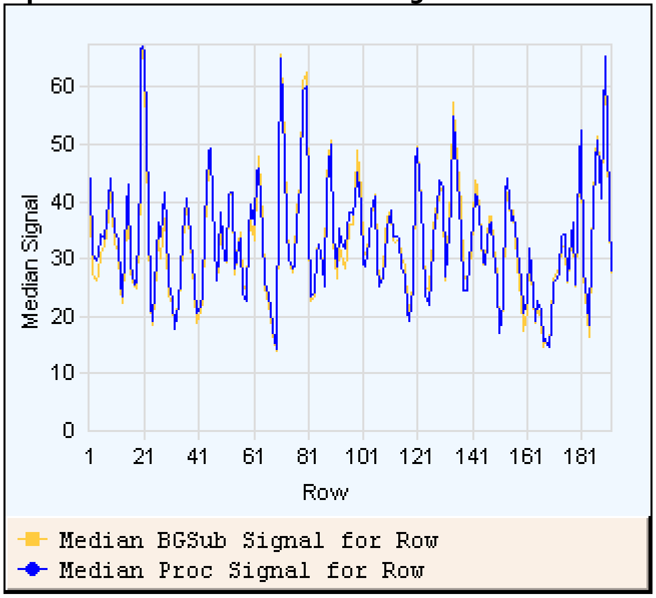

Spatial Distribution of Median Signals for each Column

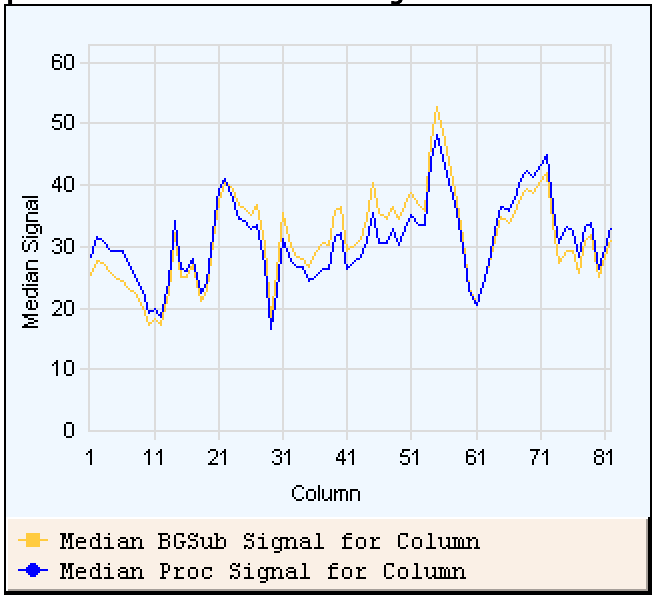

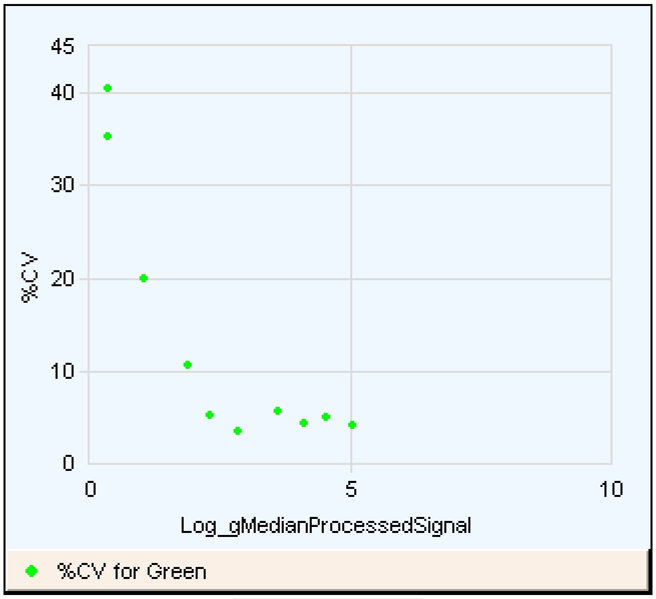

Median %CV:5.08

Agilent SpikeIns: Log(Signal) vs. Log(Relative concentration) Plot

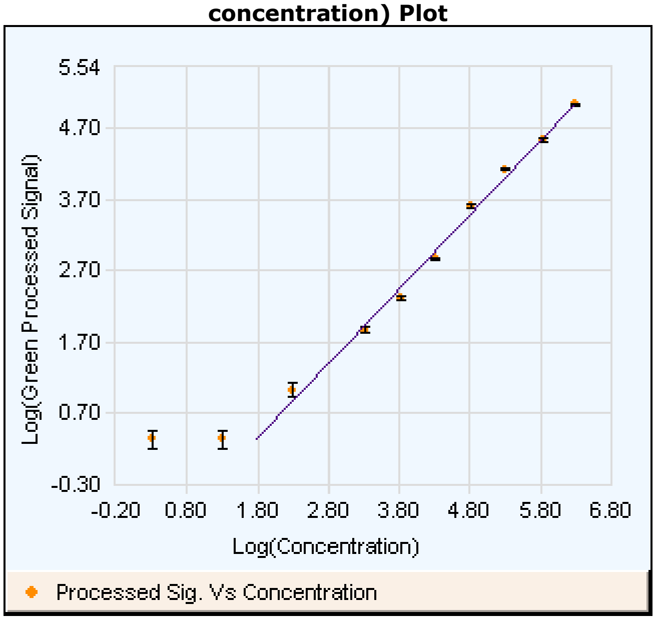

Agilent Spike-In Concentration-Response Statistics

Linear Range Statistics:

Low Signal	0.35
High Signal	5.69
Low Relative Concentration	1.78
High Relative Concentration	6.93
Slope	1.03
R^2 Value	0.99

Signal Detection Limit Statistics

Saturation Point	5.77
Low Threshold	0.10
Low Threshold Error	0.20
Spike-In Detection Limit	0.49

QC Report - Agilent Technologies : 1 Color Gene Expression

Date	Tuesday, September 20, 2011 - 11:05	Grid	035923_D_F_20110809
Image	UniversityPadova_253592310003_S01_H [1_2]	BG Method	No Background
Protocol	GE1-v5_95_Feb07 (Read Only)	Background Detrend	On(FeatNCRange, LoPass)
User Name	Administrator	Multiplicative Detrend	True
FE Version	9.5.1.1	Additive Error	2(Green)
		Saturation Value	587651 (g)

Spot Finding of the Four Corners of the Array

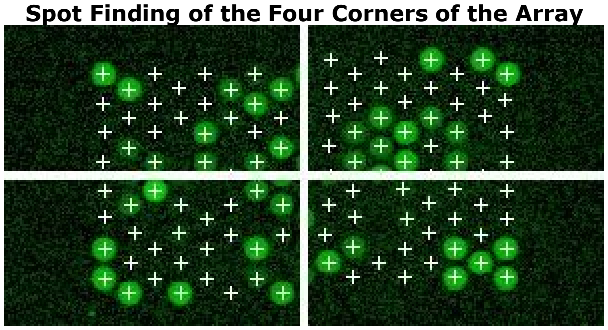

Grid Normal

	Feature	Local Background
	Green	Green
Non Uniform	0	19
Population	9	175

Spatial Distribution of All Outliers on the Array

192 rows x 82 columns

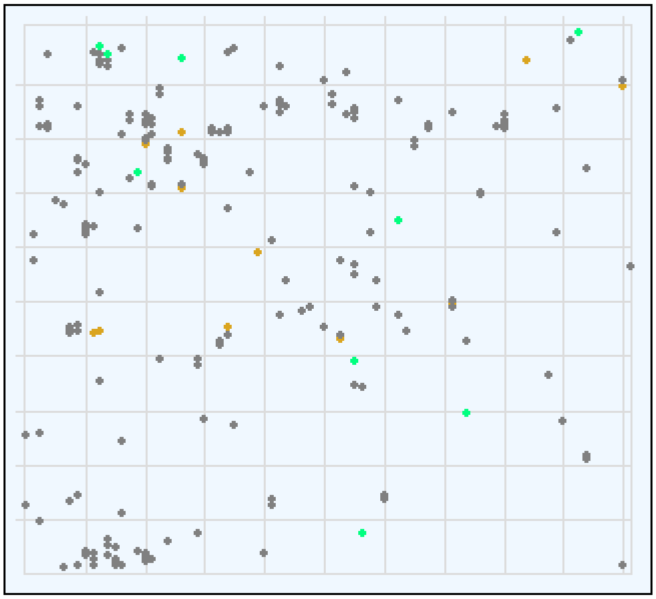

FeatureNonUnif (Green) = 0(0.00%)

GeneNonUnif (Green) = 0 (0.000 %)

BG NonUniform BG Population

Green FeaturePopulation Green Feature NonUniform

Negative Control Stats

	Green
Average Net Signals	16.01

Net Signal Statistics

Agilent SpikeIns:

	Green
# Saturated Features	0
99% of Sig. Distrib.	273221
50% of Sig. Distrib.	611
1% of Sig. Distrib.	15

Non-Control probes:

	Green
# Saturated Features	0
99% of Sig. Distrib.	34252
50% of Sig. Distrib.	29
1% of Sig. Distrib.	13

Histogram of Signals Plot

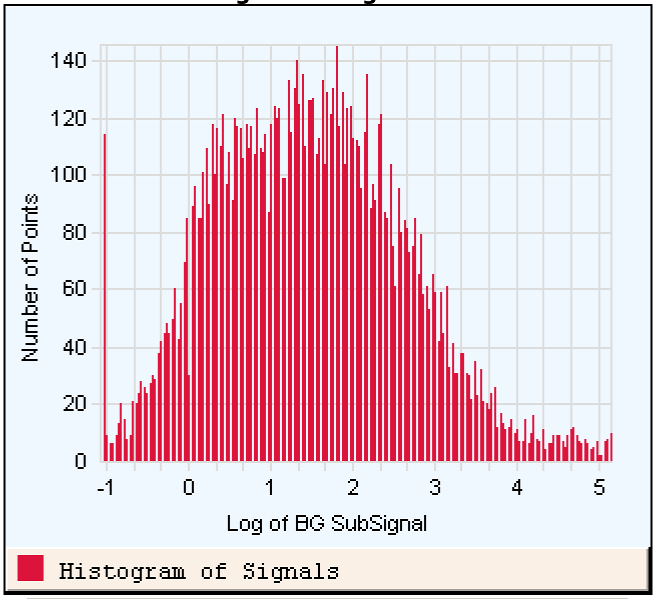

Features (NonCtrl) with BGSubSignal < 0: 2401 (Green)

StdDev Net Signals	1.99
Average BG Sub Signal	-1.13
StdDev BG Sub Signal	1.80

Local Bkg (inliers)

	Green
Number	14021
Avg	45.45
SD	2.28

Foreground Surface Fit

	Green
RMS_Fit	1.08
RMS_Resid	1.73
Avg_Fit	46.39

Multiplicative Surface Fit

	Green
RMS_Fit	0.12

Reproducibility: %CV for Replicated Probes

	Median %CV Signal (inliers)	
	Non-Control probes	Agilent SpikeIns
	Green	Green
BGSubSignal	-1.00	13.16
ProcessedSignal	-1.00	3.75

Agilent SpikeIns Signal Statistics

Probe Name	Log (Relative Conc.)	Median (Log Proc. Sig.)	% CV	StdDev
(+)E1A_r60_3	0.30	0.27	21.39	0.09
(+)E1A_r60_a104	1.30	0.57	52.17	0.22
(+)E1A_r60_a107	2.30	1.40	11.78	0.06
(+)E1A_r60_a135	3.30	2.27	4.57	0.02
(+)E1A_r60_a20	3.83	2.68	3.53	0.02
(+)E1A_r60_a22	4.30	3.20	2.78	0.01
(+)E1A_r60_a97	4.82	3.92	2.36	0.01
(+)E1A_r60_n11	5.30	4.45	4.32	0.02
(+)E1A_r60_n9	5.82	4.82	3.75	0.02
(+)E1A_r60_1	6.30	5.39	4.20	0.02

Agilent SpikeIns: %CV of Avg. Processed Signal Plot

Spatial Distribution of Median Signals for each Row

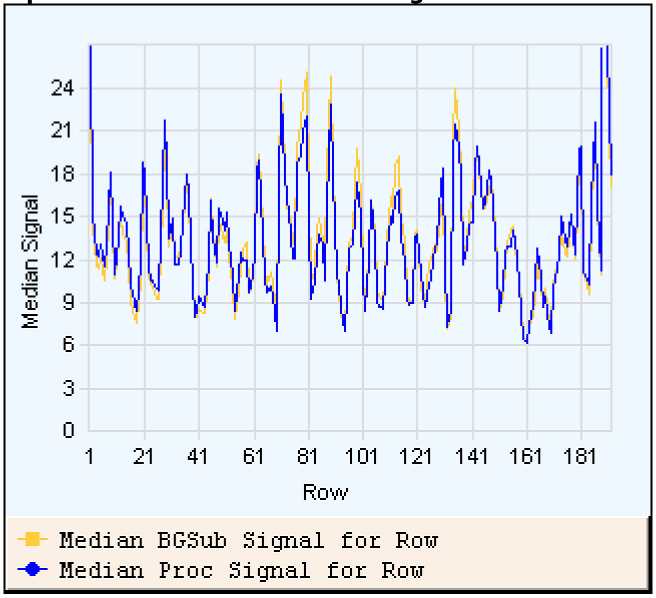

Spatial Distribution of Median Signals for each Column

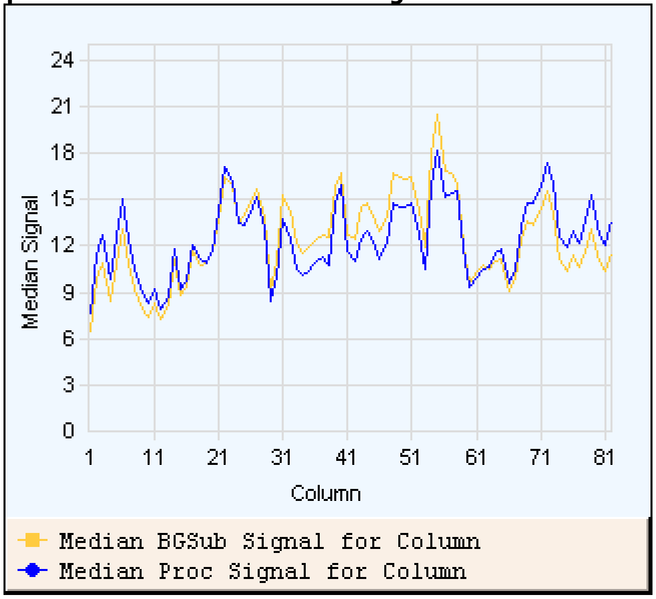

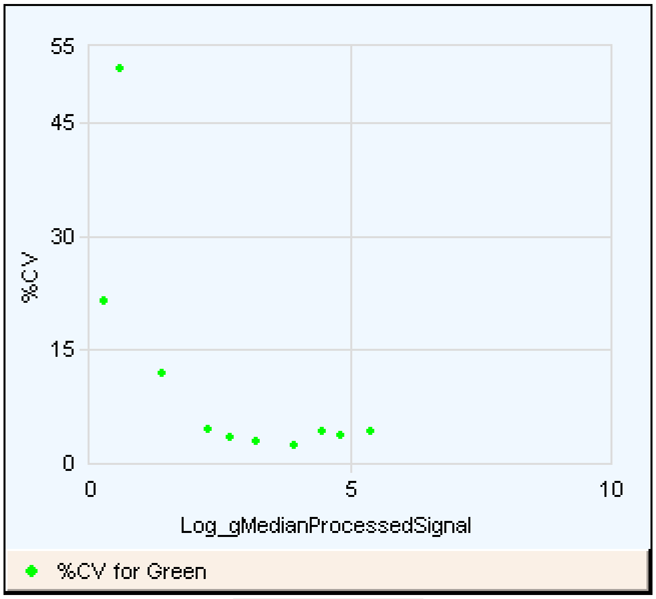

Median %CV:3.75

Agilent SpikeIns: Log(Signal) vs. Log(Relative concentration) Plot

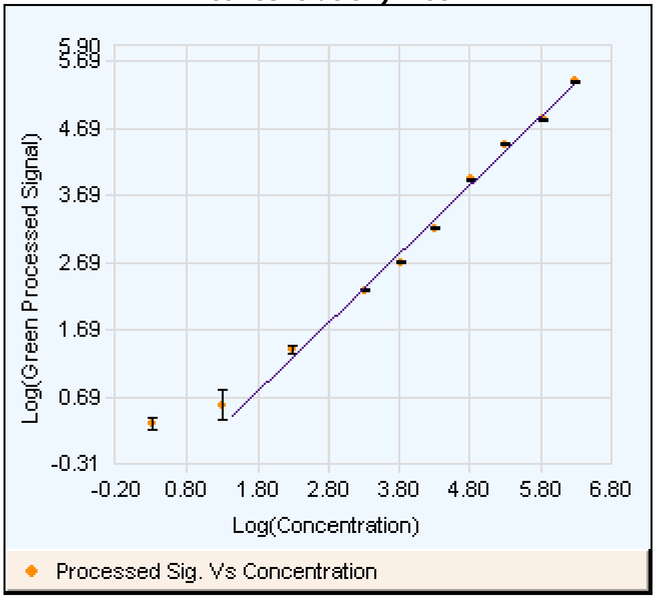

Agilent Spike-In Concentration-Response Statistics

Linear Range Statistics:

Low Signal	0.39
High Signal	5.66
Low Relative Concentration	1.43
High Relative Concentration	6.60
Slope	1.02
R^2 Value	0.99

Signal Detection Limit Statistics

Saturation Point	5.77
Low Threshold	0.12
Low Threshold Error	0.24
Spike-In Detection Limit	0.78

QC Report - Agilent Technologies : 1 Color Gene Expression

Date	Tuesday, September 20, 2011 - 11:05	Grid	035923_D_F_20110809
Image	UniversityPadova_253592310003_S01_H [1_3]	BG Method	No Background
Protocol	GE1-v5_95_Feb07 (Read Only)	Background Detrend	On(FeatNCRange, LoPass)
User Name	Administrator	Multiplicative Detrend	True
FE Version	9.5.1.1	Additive Error	2(Green)
		Saturation Value	587172 (g)

Spot Finding of the Four Corners of the Array

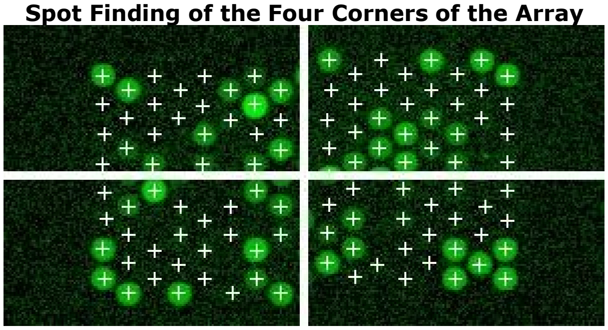

Grid Normal

	Feature	Local Background
	Green	Green
Non Uniform	1	35
Population	8	184

Spatial Distribution of All Outliers on the Array

192 rows x 82 columns

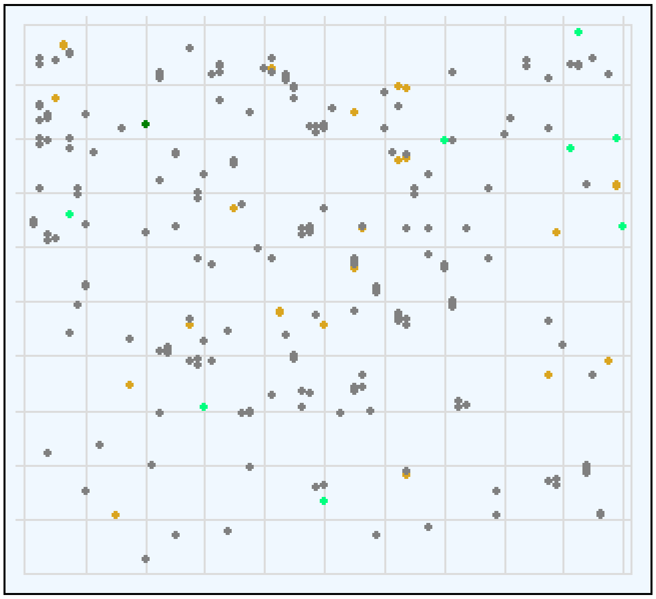

FeatureNonUnif (Green) = 1(0.01%)

GeneNonUnif (Green) = 1 (0.007 %)

BG NonUniform · BG Population · Green FeaturePopulation · Green Feature NonUniform

Negative Control Stats

	Green
Average Net Signals	16.39

Net Signal Statistics

Agilent SpikeIns:

	Green
# Saturated Features	0
99% of Sig. Distrib.	254778
50% of Sig. Distrib.	568
1% of Sig. Distrib.	15

Non-Control probes:

	Green
# Saturated Features	1
99% of Sig. Distrib.	50422
50% of Sig. Distrib.	35
1% of Sig. Distrib.	13

Histogram of Signals Plot

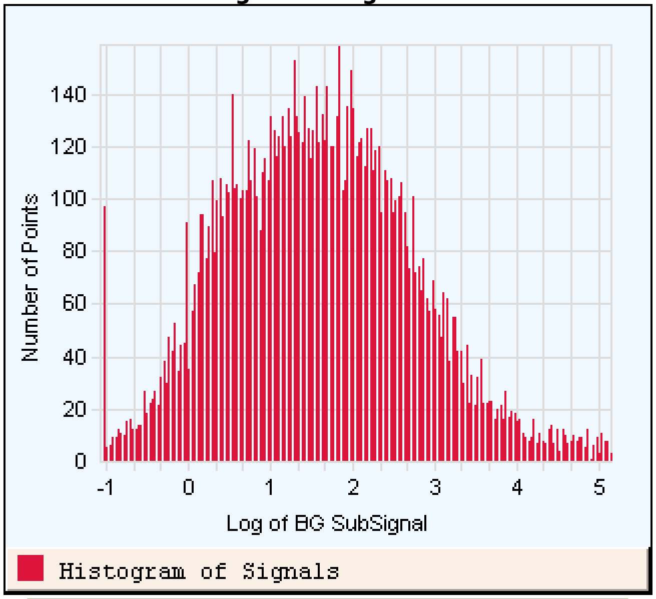

Features (NonCtrl) with BGSubSignal < 0: 2073 (Green)

StdDev Net Signals	1.77
Average BG Sub Signal	-1.06
StdDev BG Sub Signal	1.62

Local Bkg (inliers)

	Green
Number	13998
Avg	44.69
SD	2.08

Foreground Surface Fit

	Green
RMS_Fit	0.80
RMS_Resid	1.80
Avg_Fit	46.65

Multiplicative Surface Fit

	Green
RMS_Fit	0.12

Reproducibility: %CV for Replicated Probes

	Median %CV Signal (inliers)	
	Non-Control probes	Agilent SpikeIns
	Green	Green
BGSubSignal	-1.00	12.42
ProcessedSignal	-1.00	3.08

Agilent SpikeIns Signal Statistics

Probe Name	Log (Relative Conc.)	Median (Log Proc. Sig.)	% CV	StdDev
(+)E1A_r60_3	0.30	0.30	297.89	0.38
(+)E1A_r60_a104	1.30	0.67	28.82	0.13
(+)E1A_r60_a107	2.30	1.32	13.88	0.06
(+)E1A_r60_a135	3.30	2.21	4.56	0.02
(+)E1A_r60_a20	3.83	2.66	3.15	0.01
(+)E1A_r60_a22	4.30	3.14	2.41	0.01
(+)E1A_r60_a97	4.82	3.87	3.23	0.01
(+)E1A_r60_n11	5.30	4.41	1.98	0.01
(+)E1A_r60_n9	5.82	4.80	2.51	0.01
(+)E1A_r60_1	6.30	5.35	3.08	0.01

Agilent SpikeIns: %CV of Avg. Processed Signal Plot

Spatial Distribution of Median Signals for each Row

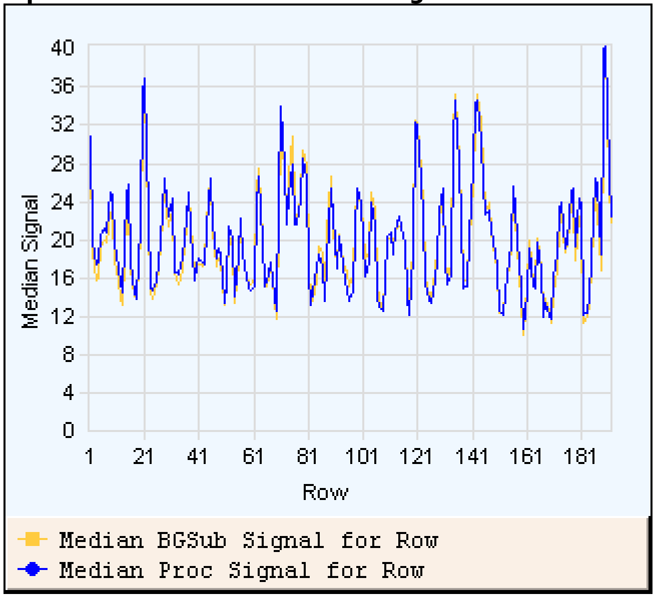

Spatial Distribution of Median Signals for each Column

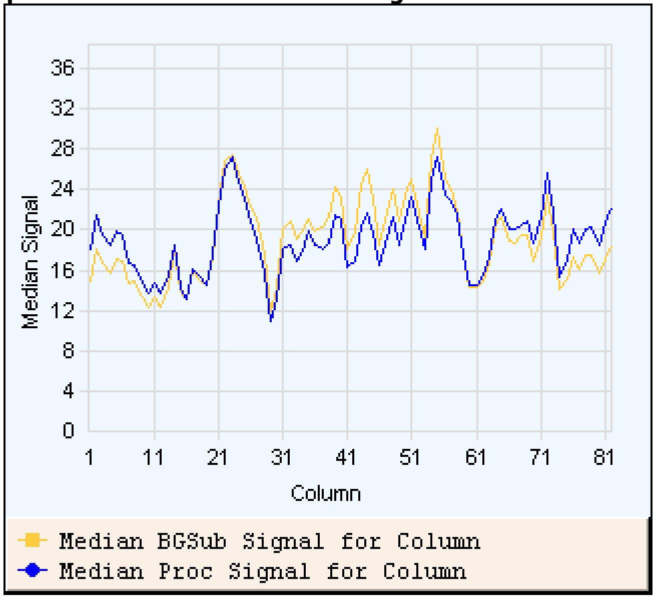

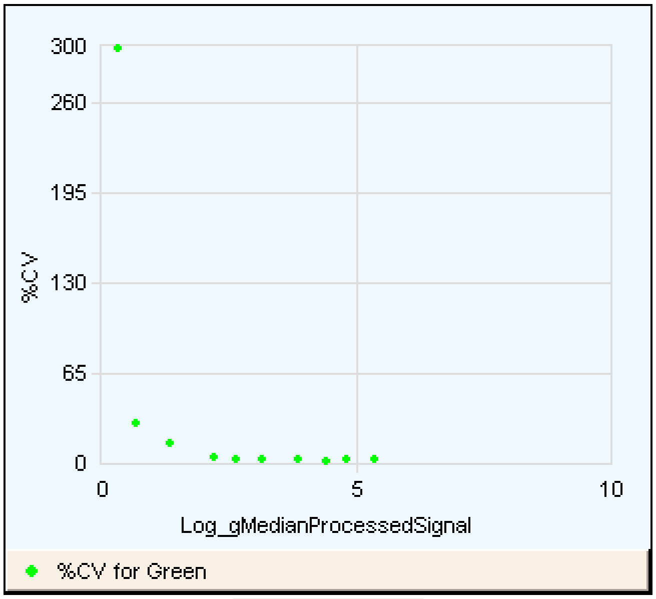

Median %CV:3.08

Agilent SpikeIns: Log(Signal) vs. Log(Relative concentration) Plot

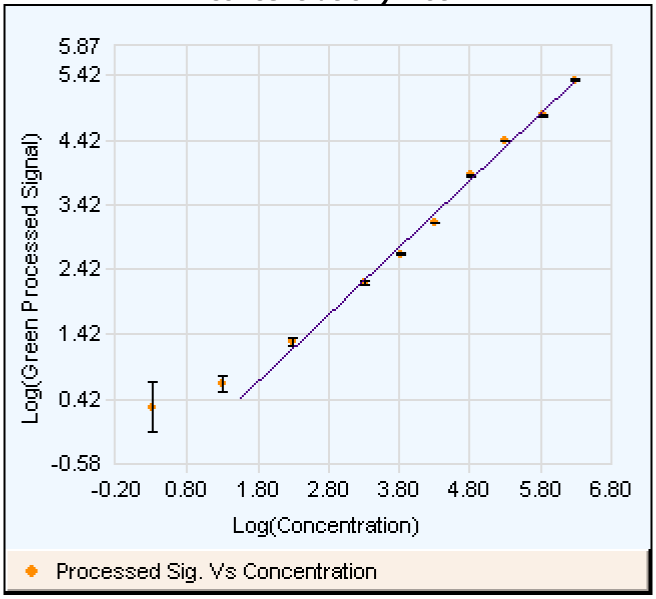

Agilent Spike-In Concentration-Response Statistics

Linear Range Statistics:

Low Signal	0.45
High Signal	5.67
Low Relative Concentration	1.56
High Relative Concentration	6.64
Slope	1.03
R^2 Value	1.00

Signal Detection Limit Statistics

Saturation Point	5.77
Low Threshold	0.21
Low Threshold Error	0.40
Spike-In Detection Limit	1.35

QC Report - Agilent Technologies : 1 Color Gene Expression

Date	Wednesday, September 21, 2011 - 10:49	Grid	035923_D_F_20110809
Image	UniversityPadova_253592310005_S01_H [1_1]	BG Method	No Background
Protocol	GE1-v5_95_Feb07 (Read Only)	Background Detrend	On(FeatNCRange, LoPass)
User Name	Administrator	Multiplicative Detrend	True
FE Version	9.5.1.1	Additive Error	2(Green)
		Saturation Value	580149 (g)

Spot Finding of the Four Corners of the Array

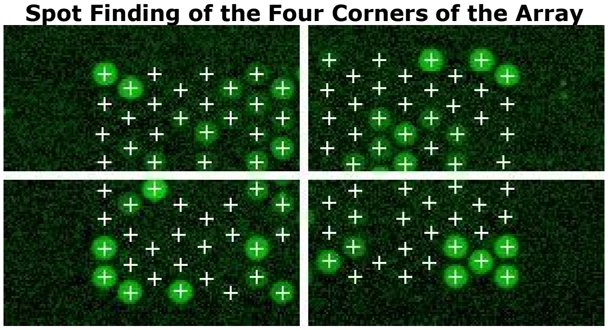

Grid Normal

	Feature	Local Background
	Green	Green
Non Uniform	2	14
Population	20	228

Spatial Distribution of All Outliers on the Array

192 rows x 82 columns

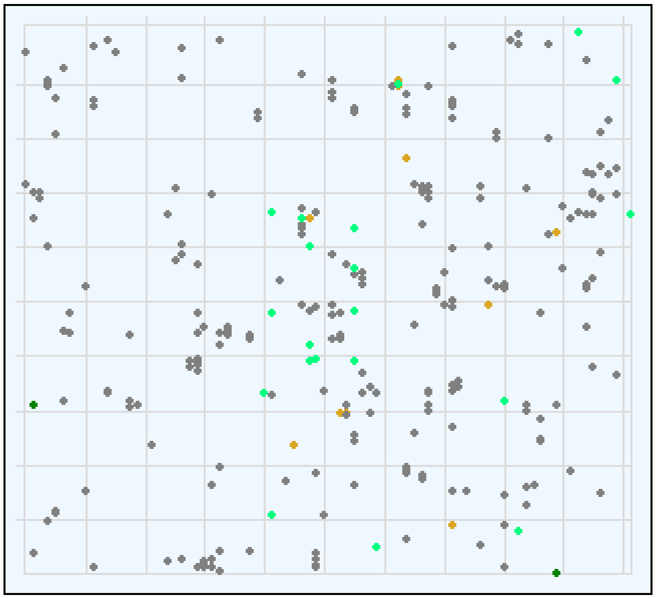

FeatureNonUnif (Green) = 2(0.01%)

GeneNonUnif (Green) = 2 (0.015 %)

BG NonUniform BG Population

Green FeaturePopulation Green Feature NonUniform

Negative Control Stats

	Green
Average Net Signals	15.69

Net Signal Statistics

Agilent SpikeIns:

	Green
# Saturated Features	0
99% of Sig. Distrib.	190395
50% of Sig. Distrib.	471
1% of Sig. Distrib.	14

Non-Control probes:

	Green
# Saturated Features	0
99% of Sig. Distrib.	19636
50% of Sig. Distrib.	24
1% of Sig. Distrib.	13

Histogram of Signals Plot

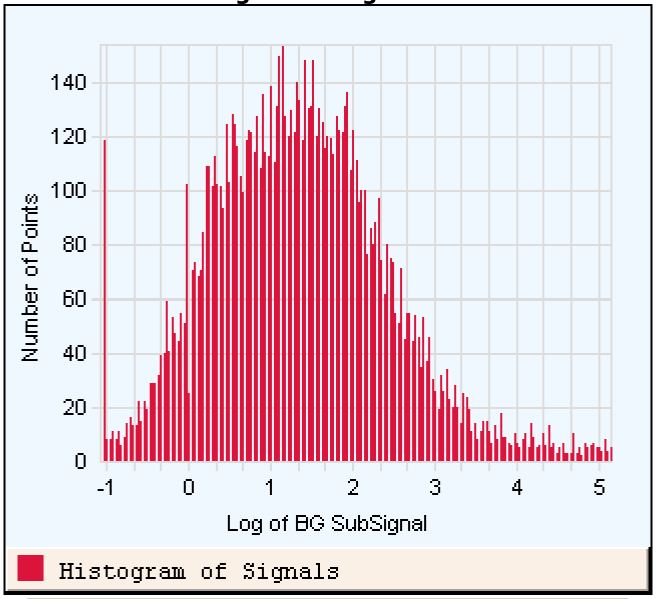

Features (NonCtrl) with BGSubSignal < 0: 3288 (Green)

StdDev Net Signals	1.59
Average BG Sub Signal	-1.42
StdDev BG Sub Signal	1.60

Local Bkg (inliers)

	Green
Number	13968
Avg	43.08
SD	1.78

Foreground Surface Fit

	Green
RMS_Fit	0.67
RMS_Resid	2.21
Avg_Fit	46.01

Multiplicative Surface Fit

	Green
RMS_Fit	0.10

Reproducibility: %CV for Replicated Probes

	Median %CV Signal (inliers)	
	Non-Control probes	Agilent SpikeIns
	Green	Green
BGSubSignal	-1.00	12.41
ProcessedSignal	-1.00	4.67

Agilent SpikeIns Signal Statistics

Probe Name	Log (Relative Conc.)	Median (Log Proc. Sig.)	% CV	StdDev
(+)E1A_r60_3	0.30	0.38	26.39	0.10
(+)E1A_r60_a104	1.30	0.52	33.77	0.15
(+)E1A_r60_a107	2.30	1.19	43.19	0.16
(+)E1A_r60_a135	3.30	2.11	5.32	0.02
(+)E1A_r60_a20	3.83	2.53	5.21	0.02
(+)E1A_r60_a22	4.30	2.97	4.60	0.02
(+)E1A_r60_a97	4.82	3.75	3.79	0.02
(+)E1A_r60_n11	5.30	4.30	4.47	0.02
(+)E1A_r60_n9	5.82	4.66	6.41	0.03
(+)E1A_r60_1	6.30	5.25	4.67	0.02

Agilent SpikeIns: %CV of Avg. Processed Signal Plot

Spatial Distribution of Median Signals for each Row

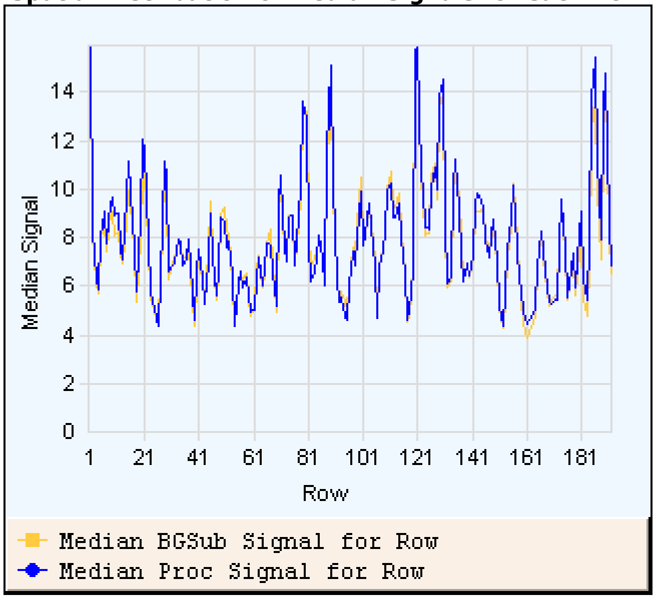

Spatial Distribution of Median Signals for each Column

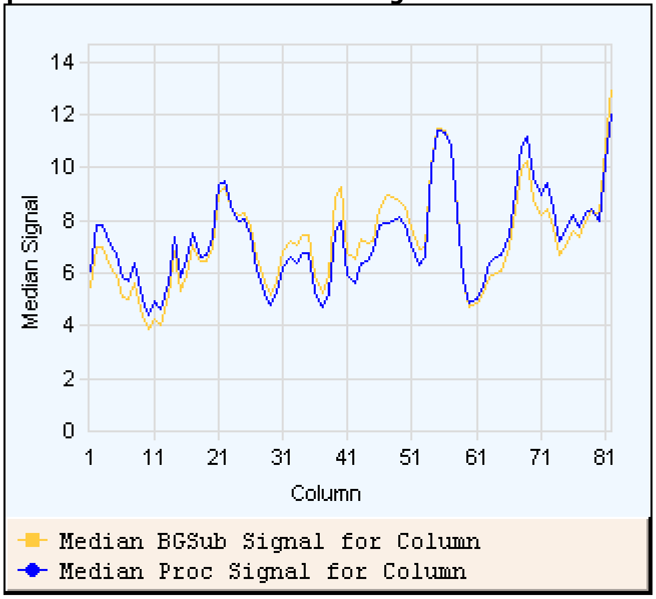

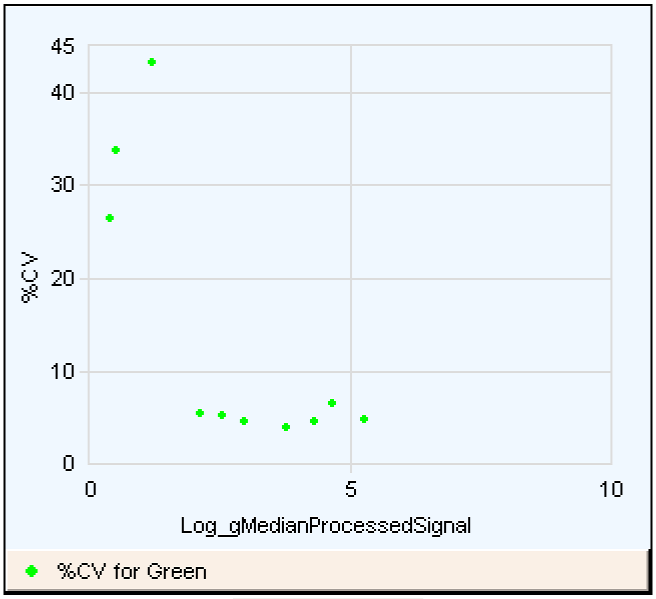

Median %CV:4.67

Agilent SpikeIns: Log(Signal) vs. Log(Relative concentration) Plot

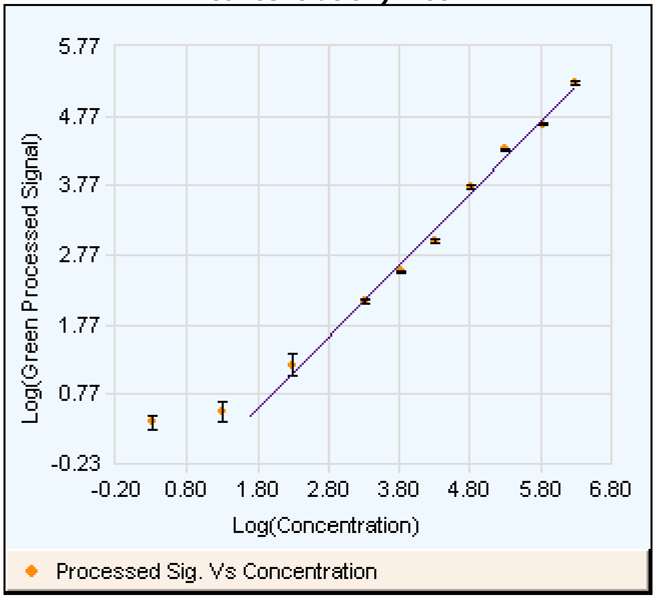

Agilent Spike-In Concentration-Response Statistics

Linear Range Statistics:

Low Signal	0.46
High Signal	5.67
Low Relative Concentration	1.70
High Relative Concentration	6.75
Slope	1.03
R^2 Value	0.99

Signal Detection Limit Statistics

Saturation Point	5.76
Low Threshold	0.21
Low Threshold Error	0.18
Spike-In Detection Limit	0.68

QC Report - Agilent Technologies : 1 Color Gene Expression

Date	Tuesday, September 20, 2011 - 11:05	Grid	035923_D_F_20110809
Image	UniversityPadova_253592310003_S01_H [1_4]	BG Method	No Background
Protocol	GE1-v5_95_Feb07 (Read Only)	Background Detrend	On(FeatNCRange, LoPass)
User Name	Administrator	Multiplicative Detrend	True
FE Version	9.5.1.1	Additive Error	2(Green)
		Saturation Value	586871 (g)

Spot Finding of the Four Corners of the Array

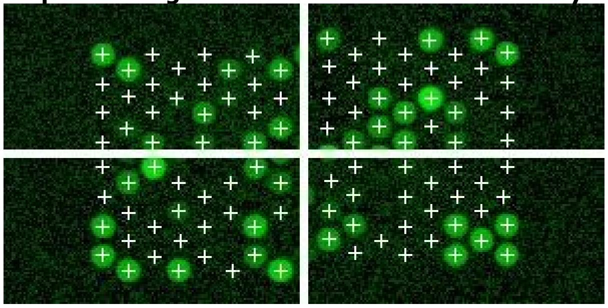

Grid Normal

	Feature	Local Background
	Green	Green
Non Uniform	7	41
Population	14	185

Spatial Distribution of All Outliers on the Array

192 rows x 82 columns

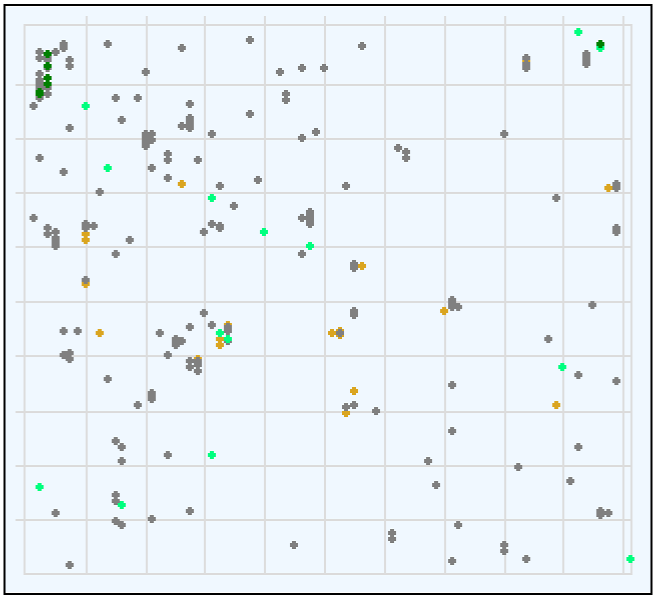

FeatureNonUnif (Green) = 7(0.05%)

GeneNonUnif (Green) = 7 (0.051 %)

BG NonUniform, BG Population, Green FeaturePopulation, Green Feature NonUniform

Negative Control Stats

	Green
Average Net Signals	16.00

Net Signal Statistics

Agilent SpikeIns:

	Green
# Saturated Features	0
99% of Sig. Distrib.	289235
50% of Sig. Distrib.	714
1% of Sig. Distrib.	16

Non-Control probes:

	Green
# Saturated Features	0
99% of Sig. Distrib.	39222
50% of Sig. Distrib.	31
1% of Sig. Distrib.	13

Histogram of Signals Plot

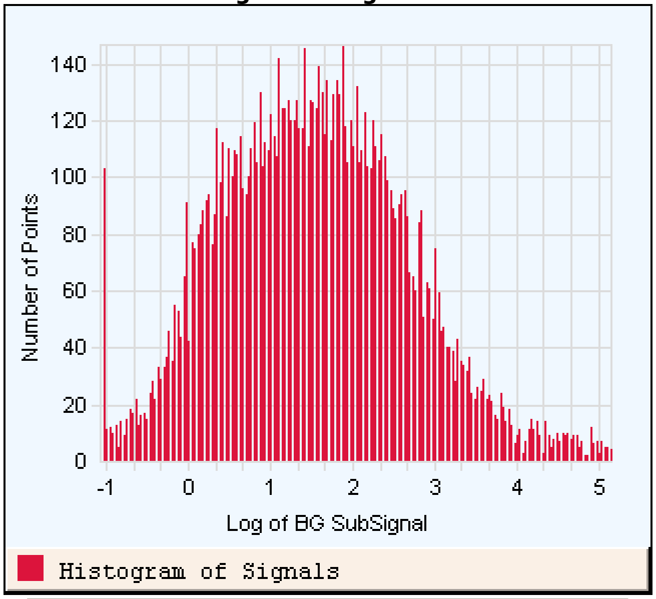

Features (NonCtrl) with BGSubSignal < 0: 2445 (Green)

StdDev Net Signals	1.88
Average BG Sub Signal	-1.22
StdDev BG Sub Signal	1.64

Local Bkg (inliers)

	Green
Number	14002
Avg	42.71
SD	2.04

Foreground Surface Fit

	Green
RMS_Fit	1.02
RMS_Resid	1.92
Avg_Fit	46.43

Multiplicative Surface Fit

	Green
RMS_Fit	0.11

Reproducibility: %CV for Replicated Probes

	Median %CV Signal (inliers)	
	Non-Control probes	Agilent SpikeIns
	Green	Green
BGSubSignal	-1.00	12.16
ProcessedSignal	-1.00	3.55

Agilent SpikeIns Signal Statistics

Probe Name	Log (Relative Conc.)	Median (Log Proc. Sig.)	% CV	StdDev
(+)E1A_r60_3	0.30	0.30	40.34	0.14
(+)E1A_r60_a104	1.30	0.69	38.40	0.16
(+)E1A_r60_a107	2.30	1.40	13.82	0.06
(+)E1A_r60_a135	3.30	2.30	4.76	0.02
(+)E1A_r60_a20	3.83	2.75	3.55	0.02
(+)E1A_r60_a22	4.30	3.20	2.87	0.01
(+)E1A_r60_a97	4.82	3.97	3.46	0.02
(+)E1A_r60_n11	5.30	4.49	2.82	0.01
(+)E1A_r60_n9	5.82	4.88	3.64	0.02
(+)E1A_r60_1	6.30	5.41	3.83	0.02

Agilent SpikeIns: %CV of Avg. Processed Signal Plot

Spatial Distribution of Median Signals for each Row

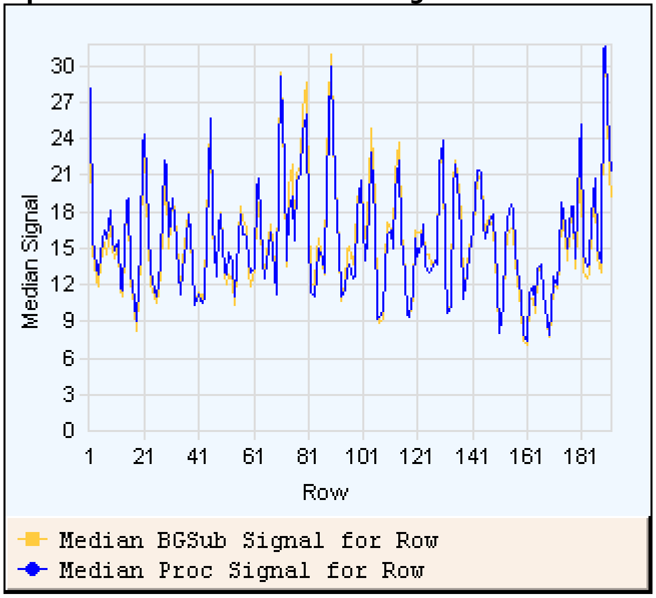

Spatial Distribution of Median Signals for each Column

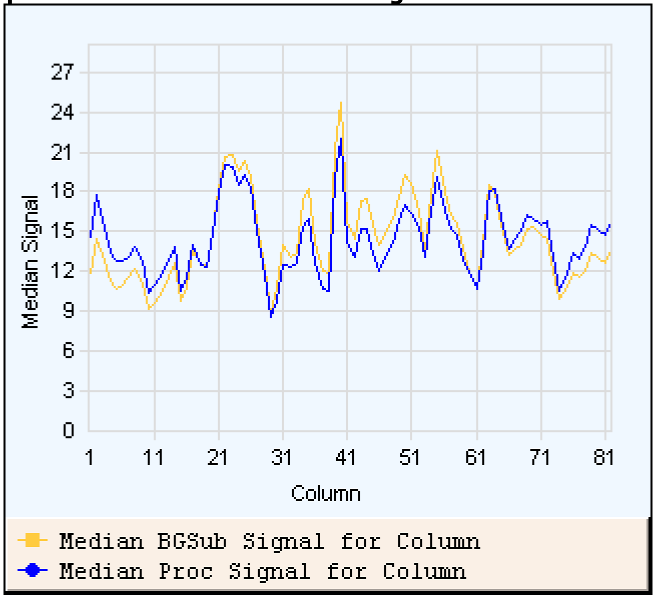

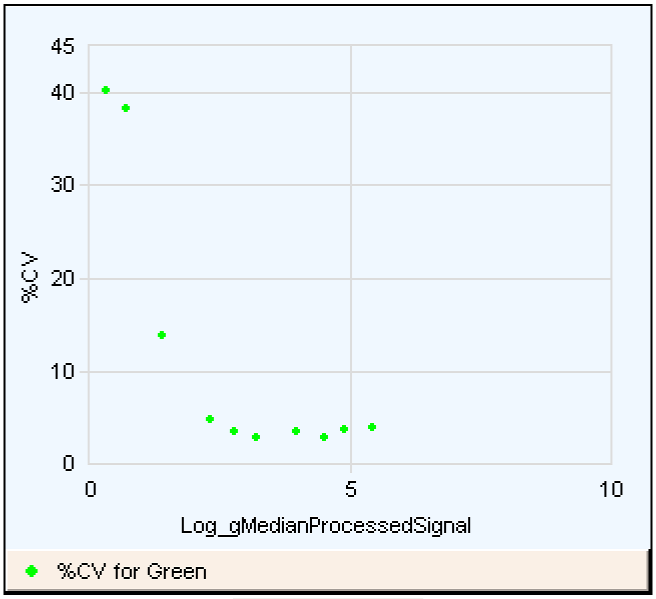

Median %CV:3.55

Agilent SpikeIns: Log(Signal) vs. Log(Relative concentration) Plot

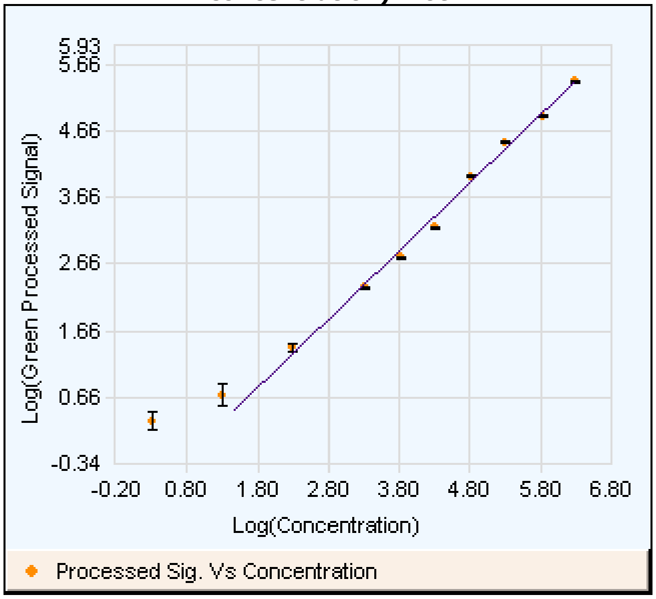

Agilent Spike-In Concentration-Response Statistics

Linear Range Statistics:

Low Signal	0.46
High Signal	5.65
Low Relative Concentration	1.48
High Relative Concentration	6.54
Slope	1.03
R^2 Value	1.00

Signal Detection Limit Statistics

Saturation Point	5.77
Low Threshold	0.20
Low Threshold Error	0.21
Spike-In Detection Limit	0.84

QC Report - Agilent Technologies : 1 Color Gene Expression

Date	Tuesday, September 20, 2011 - 11:05	Grid	035923_D_F_20110809
Image	UniversityPadova_253592310003_S01_H [2_1]	BG Method	No Background
Protocol	GE1-v5_95_Feb07 (Read Only)	Background Detrend	On(FeatNCRange, LoPass)
User Name	Administrator	Multiplicative Detrend	True
FE Version	9.5.1.1	Additive Error	3(Green)
		Saturation Value	588580 (g)

Spot Finding of the Four Corners of the Array

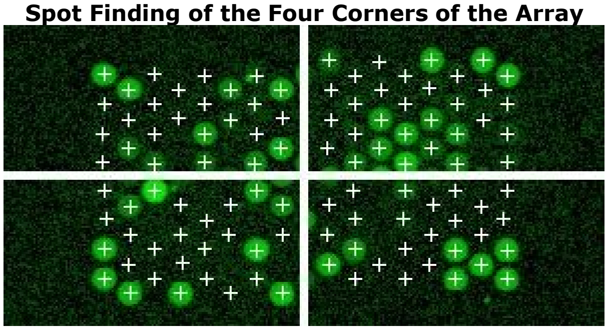

Grid Normal

	Feature	Local Background
	Green	Green
Non Uniform	3	28
Population	21	255

Spatial Distribution of All Outliers on the Array

192 rows x 82 columns

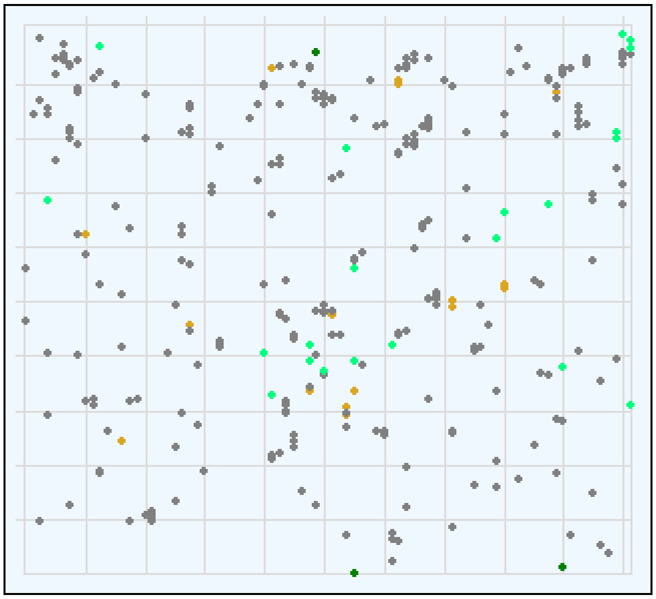

FeatureNonUnif (Green) = 3(0.02%)

GeneNonUnif (Green) = 3 (0.022 %)

BG NonUniform | BG Population | Green FeaturePopulation | Green Feature NonUniform

Negative Control Stats

	Green
Average Net Signals	15.63

Net Signal Statistics

Agilent SpikeIns:

	Green
# Saturated Features	0
99% of Sig. Distrib.	211862
50% of Sig. Distrib.	524
1% of Sig. Distrib.	14

Non-Control probes:

	Green
# Saturated Features	0
99% of Sig. Distrib.	42103
50% of Sig. Distrib.	35
1% of Sig. Distrib.	13

Histogram of Signals Plot

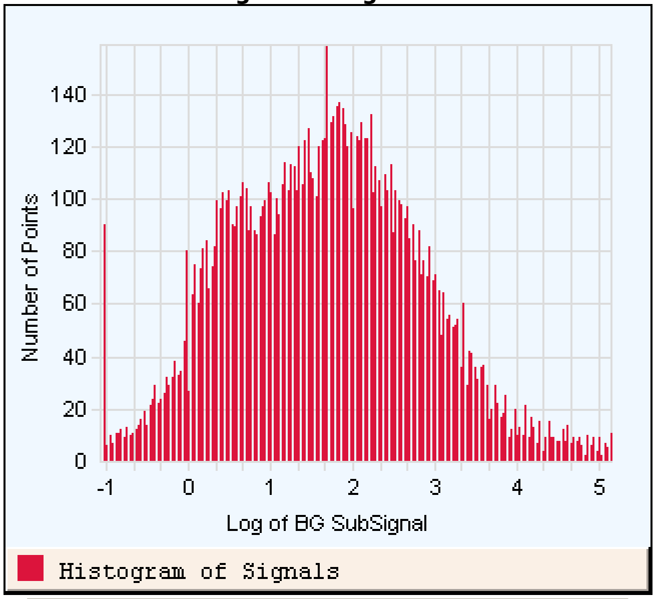

Features (NonCtrl) with BGSubSignal < 0: 2684 (Green)

StdDev Net Signals	1.99
Average BG Sub Signal	-1.70
StdDev BG Sub Signal	2.22

Local Bkg (inliers)

	Green
Number	13934
Avg	44.95
SD	1.95

Foreground Surface Fit

	Green
RMS_Fit	1.53
RMS_Resid	2.60
Avg_Fit	46.71

Multiplicative Surface Fit

	Green
RMS_Fit	0.12

Reproducibility: %CV for Replicated Probes

	Median %CV Signal (inliers)	
	Non-Control probes	Agilent SpikeIns
	Green	Green
BGSubSignal	-1.00	13.51
ProcessedSignal	-1.00	4.24

Agilent SpikeIns Signal Statistics

Probe Name	Log (Relative Conc.)	Median (Log Proc. Sig.)	% CV	StdDev
(+)E1A_r60_3	0.30	0.44	120.00	0.22
(+)E1A_r60_a104	1.30	0.58	39.15	0.14
(+)E1A_r60_a107	2.30	1.31	22.41	0.11
(+)E1A_r60_a135	3.30	2.16	6.94	0.03
(+)E1A_r60_a20	3.83	2.58	4.10	0.02
(+)E1A_r60_a22	4.30	3.09	3.15	0.01
(+)E1A_r60_a97	4.82	3.80	4.87	0.02
(+)E1A_r60_n11	5.30	4.35	4.24	0.02
(+)E1A_r60_n9	5.82	4.72	5.78	0.03
(+)E1A_r60_1	6.30	5.27	4.24	0.02

Agilent SpikeIns: %CV of Avg. Processed Signal Plot

Spatial Distribution of Median Signals for each Row

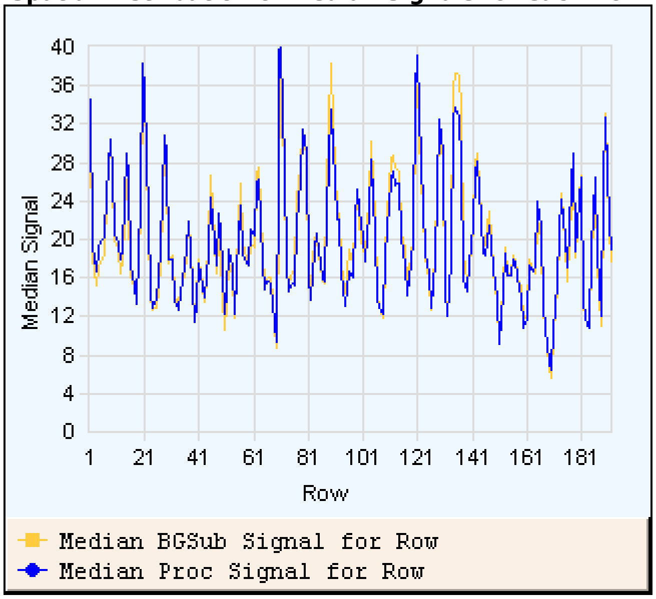

Spatial Distribution of Median Signals for each Column

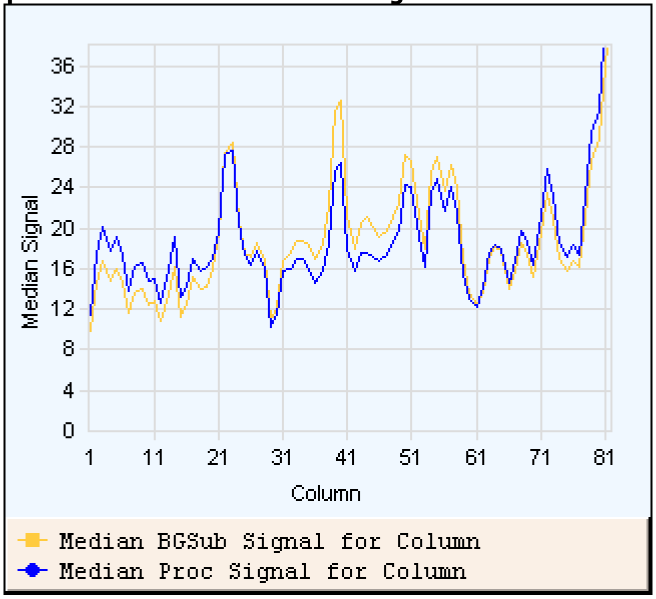

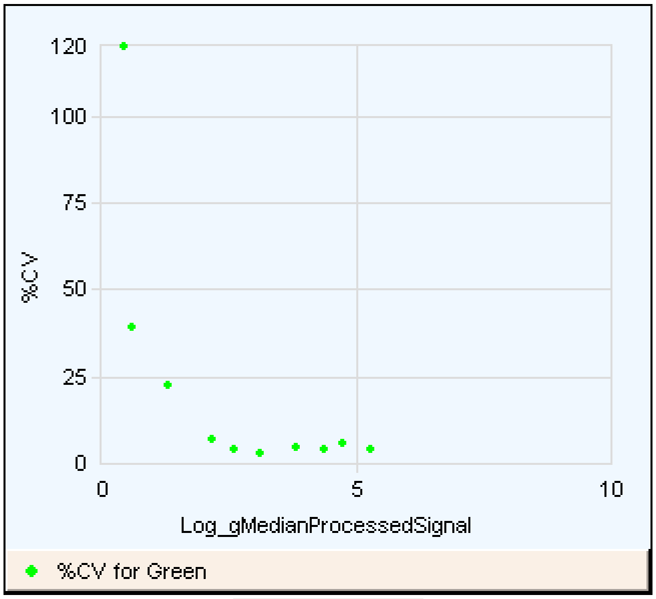

Median %CV:4.24

Agilent SpikeIns: Log(Signal) vs. Log(Relative concentration) Plot

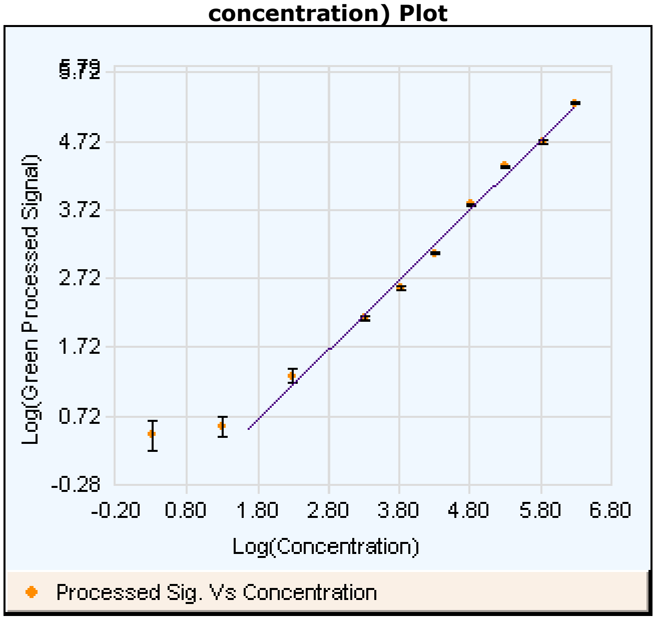

Agilent Spike-In Concentration-Response Statistics

Linear Range Statistics:

Low Signal	0.54
High Signal	5.67
Low Relative Concentration	1.67
High Relative Concentration	6.72
Slope	1.02
R^2 Value	0.99

Signal Detection Limit Statistics

Saturation Point	5.77
Low Threshold	0.28
Low Threshold Error	0.28
Spike-In Detection Limit	0.79

QC Report - Agilent Technologies : 1 Color Gene Expression

Date	Tuesday, September 20, 2011 - 11:05	Grid	035923_D_F_20110809
Image	UniversityPadova_253592310003_S01_H [2_2]	BG Method	No Background
Protocol	GE1-v5_95_Feb07 (Read Only)	Background Detrend	On(FeatNCRange, LoPass)
User Name	Administrator	Multiplicative Detrend	True
FE Version	9.5.1.1	Additive Error	2(Green)
		Saturation Value	588722 (g)

Spot Finding of the Four Corners of the Array

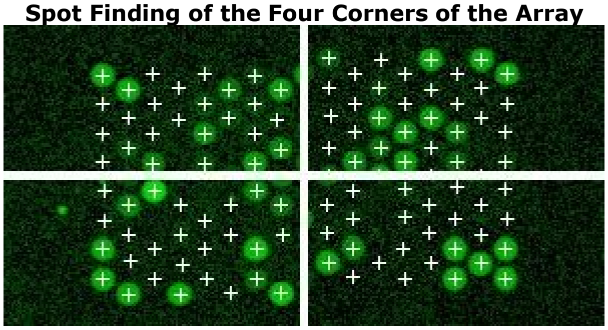

Grid Normal

	Feature	Local Background
	Green	Green
Non Uniform	4	39
Population	17	216

Spatial Distribution of All Outliers on the Array

192 rows x 82 columns

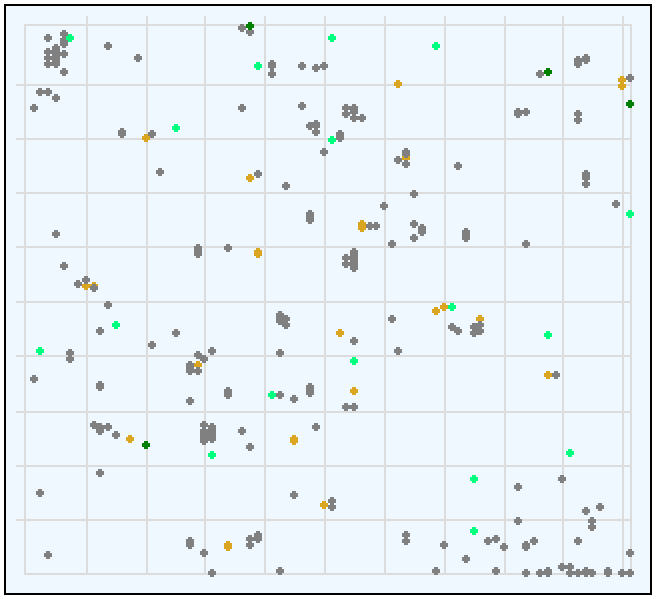

FeatureNonUnif (Green) = 4(0.03%)

GeneNonUnif (Green) = 4 (0.029 %)

BG NonUniform · BG Population · Green FeaturePopulation · Green Feature NonUniform

Negative Control Stats

	Green
Average Net Signals	15.57

Net Signal Statistics

Agilent SpikeIns:

	Green
# Saturated Features	0
99% of Sig. Distrib.	294421
50% of Sig. Distrib.	746
1% of Sig. Distrib.	15

Non-Control probes:

	Green
# Saturated Features	0
99% of Sig. Distrib.	38059
50% of Sig. Distrib.	30
1% of Sig. Distrib.	12

Histogram of Signals Plot

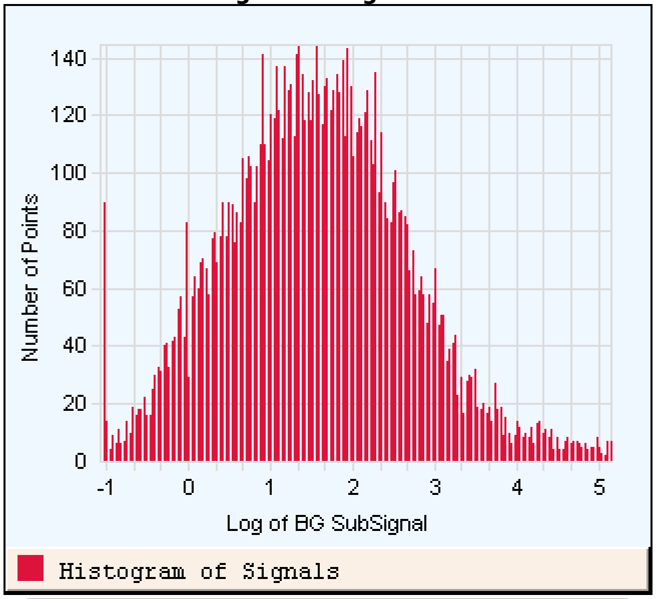

Features (NonCtrl) with BGSubSignal < 0: 2949 (Green)

StdDev Net Signals	2.27
Average BG Sub Signal	-1.86
StdDev BG Sub Signal	1.67

Local Bkg (inliers)

	Green
Number	13964
Avg	44.58
SD	2.06

Foreground Surface Fit

	Green
RMS_Fit	1.61
RMS_Resid	2.22
Avg_Fit	46.71

Multiplicative Surface Fit

	Green
RMS_Fit	0.11

Reproducibility: %CV for Replicated Probes

	Median %CV Signal (inliers)	
	Non-Control probes	Agilent SpikeIns
	Green	Green
BGSubSignal	-1.00	11.78
ProcessedSignal	-1.00	3.43

Agilent SpikeIns Signal Statistics

Probe Name	Log (Relative Conc.)	Median (Log Proc. Sig.)	% CV	StdDev
(+)E1A_r60_3	0.30	0.36	92.70	0.19
(+)E1A_r60_a104	1.30	0.52	43.88	0.18
(+)E1A_r60_a107	2.30	1.44	13.31	0.06
(+)E1A_r60_a135	3.30	2.37	4.89	0.02
(+)E1A_r60_a20	3.83	2.79	3.78	0.02
(+)E1A_r60_a22	4.30	3.20	3.25	0.01
(+)E1A_r60_a97	4.82	3.99	2.97	0.01
(+)E1A_r60_n11	5.30	4.53	3.43	0.01
(+)E1A_r60_n9	5.82	4.90	3.25	0.01
(+)E1A_r60_1	6.30	5.43	5.02	0.02

Agilent SpikeIns: %CV of Avg. Processed Signal Plot

Spatial Distribution of Median Signals for each Row

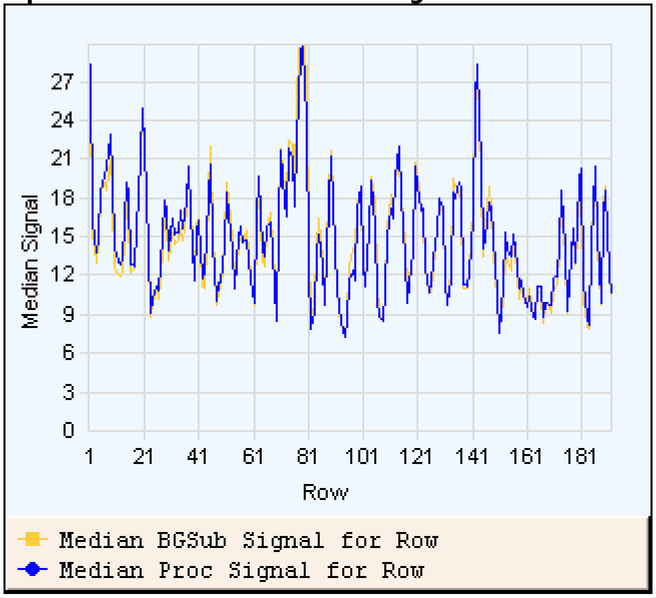

Spatial Distribution of Median Signals for each Column

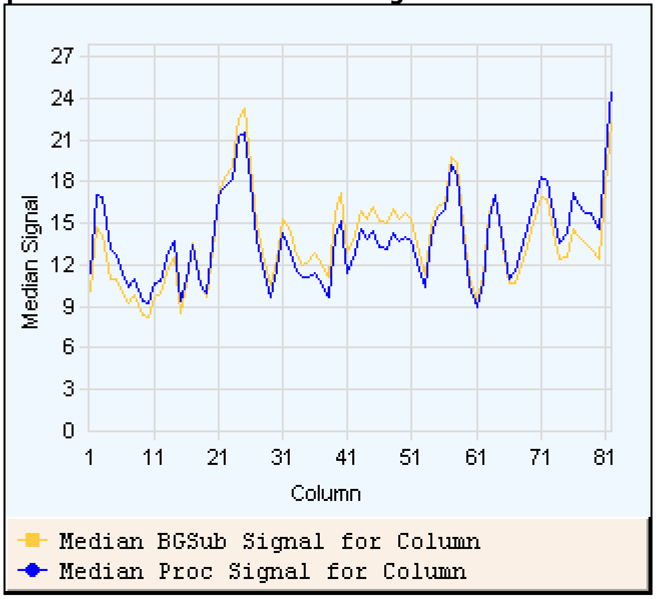

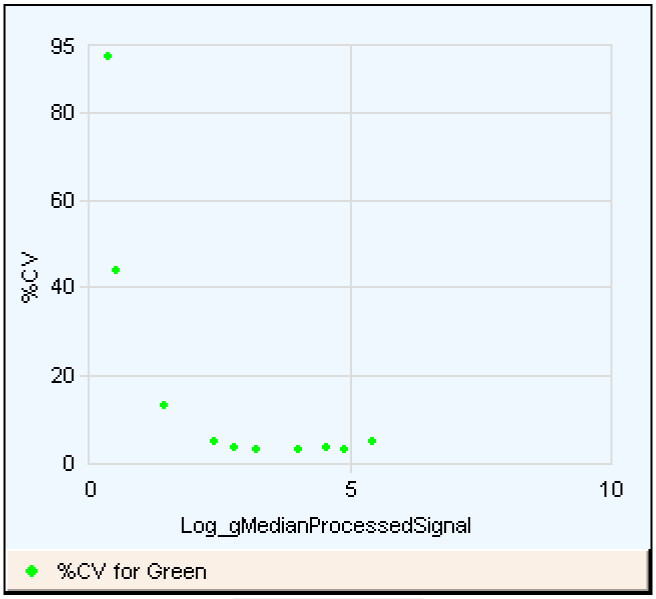

Median %CV:3.43

Agilent SpikeIns: Log(Signal) vs. Log(Relative concentration) Plot

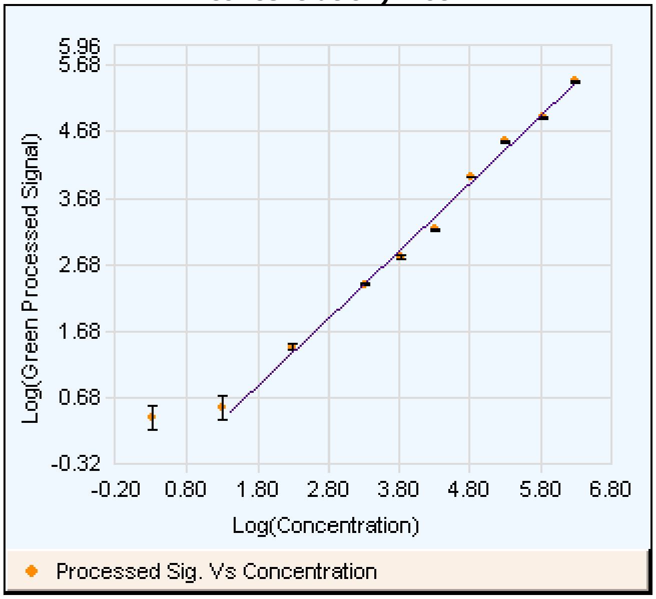

Agilent Spike-In Concentration-Response Statistics

Linear Range Statistics:

Low Signal	0.46
High Signal	5.63
Low Relative Concentration	1.43
High Relative Concentration	6.51
Slope	1.02
R^2 Value	0.99

Signal Detection Limit Statistics

Saturation Point	5.77
Low Threshold	0.16
Low Threshold Error	0.26
Spike-In Detection Limit	0.73
